# Complete Comparison Display (CCD) evaluation of ethanol extracts of *Centella asiatica* and *Withania somnifera* shows that they can non-synergistically ameliorate biochemical and behavioural damages in MPTP induced Parkinson's model of mice

**DOI:** 10.1371/journal.pone.0177254

**Published:** 2017-05-16

**Authors:** Maheep Bhatnagar, Ishan Goel, Tathagato Roy, Sunil Dutt Shukla, Sukant Khurana

**Affiliations:** 1 Department of Zoology, Mohanlal Sukhadia University, Udaipur, Rajasthan, India; 2 Pharmacology Department, Central Drug Research Institute - Lucknow, Uttar Pradesh, India; 3 Department of Biological Sciences, Indian Institute of Science Education and Research - Kolkata, Haringhata Farm, West Bengal, India; 4 Government Meera Girl's College, Udaipur, Rajasthan, India; National Institutes of Health, UNITED STATES

## Abstract

Parkinson’s disease remains as one of the most common debilitating neurodegenerative disorders. With the hopes of finding agents that can cure or reduce the pace of progression of the disease, we studied two traditional medicinal plants: *Centella asiatica* and *Withania somnifera* that have been explored in some recent studies. In agreement with the previous work on ethanol extracts of these two plants in mice model, we saw an improvement in oxidative stress profile as well as behavioral performance in 1-methyl-4-phenyl-1,2,3,6-tetrahydropyridine (MPTP) induced Parkinson-like symptoms in Balb/c mice. Given the known potential of both the herbal extracts in improving Parkinson-like symptoms, we expected the combination of the two to show better results than either of the two but surprisingly there was no additivity in either oxidative stress or behavioural recovery. In fact, in some assays, the combination performed worse than either of the two individual constituents. This effect of mixtures highlights the need of testing mixtures in supplements market using enthomedicine. The necessity of comparing multiple groups in this study to get most information from the experiments motivated us to design a ladder-like visualization to show comparison with different groups that we call complete comparison display (CCD). In summary, we show the potential of *Centella asiatica* and *Withania somnifera* to ameliorate Parkinson’s disorder.

## Introduction

The depletion of dopaminergic neurons in Substantia Nigra Pars Compacta (Snc) leads to the development of Parkinson’s disease (PD). It is a progressive age-dependent neurodegenerative syndrome. Patients exhibit symptoms of tremor, rigidity, bradykinesia, and impairment of balance [1] due to underlying neurodegeneration that involves multiple pathways, such as oxidative stress, mitochondrial damage, protein aggregation, and neuroinflammation [2, 3]. Given the various manifestations of neurodegeneration, therapeutics that could simultaneously target multiple pathways, without significant side effects, are greatly needed to slow the progression of the disease. Traditional herbal neuroactive plant extracts that have admixture of various antioxidant and neuroactive compounds, could be a good source of such therapeutics [4]. Characterization of extracts from plants that show anti-PD effects can potentially lead to discovery of new medicinal molecules. With the motivation of finding role of traditional plants, we explored neuroprotective role of extracts of two well-known Ayurvedic plants: *Centella asiatica* and *Withania somnifera* to test on MPTP treated Parkinsonian model in mice. *Centella asiatica* and *Withania somnifera* have individually shown promise for preventing PD progression. Individual treatment with madecassoside and asiaticoside, compounds isolated from *Centella asiatica*, have been reported to possess a neuroprotective role when tested in the rat model of Parkinsonism induced with MPTP [5, 6]. *Withania somnifera* extract has been shown to alleviate the symptoms of PD in maneb and paraquat induced model of PD [7]. Its neuroprotective role has also been reported in 1-methyl-4-phenyl-1, 2, 3, 6 tetrahydropyridine (MPTP) and 6-Hydroxydopamine mice (6OHD) PD models [8, 9].

Results from two studies on *Centella asiatica*, which utilized different single gene drosophilia models of Parkinsonism proved to be contradictory [10, 11]. One study utilizing single gene drosophilia model did not show a neuroprotective role of *Withania somnifera* [11]. This might be due to inherent limitations in the models themselves.

We study for the first time if the combination of the two herbs *Centella asiatica* and *Withania somnifera*, previously demonstrated to have potential in PD treatment individually would be of use in development of treatment. We expose animals to these plant extracts after the MPTP disease induction to study recovery separate from interference in induction, which can confound results. Previous studies in ethnomedicine and several branches of biology compare multiple groups, but the comparison of different groups is in contrast to only one ‘control group’. This undermines researchers’ ability to extract maximum information from experiments. We present a novel visualization tool that shows comparison of each group in an intuitive manner. We expect this tool to become a regular feature in several areas of science.

## Materials and methods

### Extract preparation and characterization

Roots of *Withania Somnifera* were purchased from a local Ayurvedic products distributor and *Centella asiatica* was collected from the forest department nursery at Banki, Udaipur, Rajasthan, India. Both plant materials were authenticated by the Department of Botany, MLS University, Udaipur, Rajasthan, India. Dried roots of Withania and leaves of Centella were ground separately into a fine powder using an electrical mixer. Fine powdered material (20g) was extracted with 800 ml 50% ethanol using soxhlet extractor for 6 hours at 80°C temperature. The ethanol extract was concentrated under reduced pressure in the Rotavapor yielding a gum-like residue. Both Withania and Centella were extracted separately. Crude extract were collected, weighed and stored in refrigerator at 4°C for future use. We did not characterize the chemical constituents of the mixture.

### Animals

Ten to 14 weeks old BALB/c mice, weighing about the 25 to 30 grams were used for this study. Animals were housed at 26 ± 1°C with a 12-hour light/dark cycle with an *ad libitum* supply of water and standard rodent chow. The Institutional Animal Ethics Committee of the Mohan Lal Sukhadia University, Udaipur was consulted and the protocol was approved by them.

### MPTP treatment

To serve as the PD model, the animals were given 2 doses of MPTP-HCl of 20 mg/kg of body weight at an interval of 2 hours. No animals died in our study. Animals were monitored at least twice every day to observe any symptoms of suffering. No analgesics were given to animals in the duration of the study.

### Perfusion

After 21 days of MPTP treatment, animals were anesthetised with 100 mg/Kg of Ketamine and 10 mg/Kg of Xylazine and perfused trans-cardially using a perfusion unit. One container of the perfusion unit was filled with chilled phosphate buffer saline (PBS) and other container with fixative. Both the containers were kept at 102.5 cm height to maintain a constant 78.5 mm Hg pressure. Opening the thoracic cavity exposed the heart and about 250 ml of phosphate buffered saline was run through left ventricle using a 21-gauge needle and simultaneously blood was allowed to flush out by puncturing the right auricle. When the whole blood was replaced with buffered saline, then the animal was perfused with 250 ml of fixative. Fixative used was different in immunohistochemistry and electron microscopy methods and are described at suitable places.

### Light microscopic validation

After perfusion, brains were fixed overnight in 4% PFA and cryopreserved in 30% sucrose solution at 4°C. We then sectioned the Substantia Nigra region (*Bregma*: − 2.70–3.70) to in 30 μm slices. To remove intrinsic peroxidase activity, we incubated Substantia Nigra slices for 5 minutes in 3% (v/v) H_2_O_2_ in water, followed by 3 rinses with water for 2 to 3 minute each. Sections were blocked using goat serum, followed by incubation with anti-Tyrosine Hydroxylase (TH-rabbit) primary antibody (Catalogue number: T8700 Sigma Aldrich) in PBS for 48 hours at 4°C. We then treated slices with secondary antibody (sheep anti-rabbit) and incubated for at least 1 hour at room temperature with ABC mix. After that slices were treated with 3, 3’-diaminobenzidine (DAB) until staining was achieved and the reaction was stopped by 1x PBS.

### EM preparation

After perfusion brains were overnight fixed in 2.5% gluteraldehyde. We separated the Substantia Nigra under stereomicroscope and fixed the brain region in 2.5% gluteraldehyde at 4°C for 16 hours. After fixation we washed the tissues in 0.2 M phosphate buffer of pH 7.2 at a temperature of 4°C. After washing tissues were taken to the E.M. facility at the Department of Anatomy, All India Institute of Medical Sciences (AIIMS), New Delhi. Tissues were rinsed again in 0.2 M phosphate buffer of pH 7.2 at a temperature of 4°C and were then post-fixed in 1% osmium tetraoxide for 100 to 120 min at 4°C. The tissues were then dehydrated through acetone. The embedding media consisted of Araldite CY212, DDSA (dodecenyl succinic anhydride*)*, DMP*-*30 (Tris(dimethylaminomethyl)phenol, and dibutyl phthalate in ratio of 10:10:0.4:1. The blocks were sectioned using glass knife on a LKBIII ultra- microtome. Lead Citrate and Uranyl acetate were used to stain ultra-thin sections of <90 nm. A Philips CM—100 electron microscope was used at an accelerating voltage of 80 KV to examine and photograph these sections.

### Experimental groups

Animals were given 21-day treatment of herbal extracts or 0.9% NaCl depending on the group and then assayed for biochemical and behavioral assays. They were divided into the following groups:

1. Animals were treated with 2 ml of 0.9% NaCl. The data from these animals has been labelled under the category of No Herbal Ext.
2. Animals treated with 40 mg/kg of body weight ethanolic extract of *Centella asiatica*. The data from these animals has been labelled under the category of Centella Ext.
3. Animals treated with 40 mg/kg of body weight ethanolic extract of *Withania somnifera*. The data from these animals has been labelled under the category of Withania Ext.
4. Animals treated with 40 mg/kg of body weight ethanolic extract of *Centella asiatica* and 40 mg/kg of body weight ethanolic extract of *Withania somnifera*. The data from these animals has been labelled under the category of Withania & Centella Ext.
5. Animals which received only the MPTP treatment were labelled under the category of MPTP NoHerbal Ext.
6. Animals were treated with MPTP followed by 40 mg/kg of body weight ethanolic extract of *Centella asiatica* for 21 days. The data from these animals has been labelled under the category of MPTP Centella Ext.
7. Animals were treated with MPTP followed by 40 mg/kg of body weight ethanolic extract of *Withania somnifera* for 21 consecutive days. The data from these animals has been labeled under the category of MPTP Withania Ext.
8. Animals were treated with MPTP followed by 40 mg/kg of body weight ethanolic extract of *Withania somnifera* and 40 mg/kg of *Centella asiatica* for 21 consecutive days. The data from these animals has been labeled under the category of MPTP Withania & Centella Ext.

### Biochemical analysis

For biochemical assays, in the morning all mice were sacrificed by cervical decapitation to avoid diurnal variations of the enzymes, endogenous amines, and other antioxidant molecules. We separated the Substantia Nigra portion and ice-cold PBS (pH 8.0; concentration: 15% w/v) was used to homogenize it separately. Homogenized samples were centrifuged briefly to collect supernatant. Aliquot was taken from supernatants for biochemical estimations and remaining was stored at -20°C. We used homogenized Substantia Nigra for conducting assays on Superoxide dismutase (SOD), Catalyse (CAT), Glutathione peroxidase (Gpx), Reduced glutathione (GSH), and Lipid peroxidation (LPO).

#### Superoxide dismutase (SOD)

Superoxide dismutase (SOD) was determined using assay mixture containing 0.052M sodium pyrophosphate buffer, 186 μM phenazine methosulphate, 300 μM nitroblue tetrazolium(NBT), and 780 μM NADH. 50% inhibition of NBT corresponded to 1 single unit of enzyme. Enzymes specific activity was expressed in mu/mg. [12].

#### Catalase (CAT)

We chose to use colorimetric evaluation of Catalase activity. 5% potassium dichromate and glacial acetic acid were mixed in 1:3 ratio to give color reaction. The utilization of hydrogen peroxide was assessed by measuring intensity of the color reaction at 620 nm and expressed in form of uMoles/min/mg of protein. [13].

#### Glutathione peroxidase (GPx)

Glutathione peroxidase (GPx) activity was measured using the reaction mixture of 0.25 mM hydrogen peroxide, 1 eu/ml Glutathione Reductase, 1 mM sodium azide, 1 mM glutathione, 1 mM EDTA, 0.2 mM of NADPH, 0.05 M PBS (pH 7.0), and 10% w/v post-microsomal supernatants (PMS). At room temperature, NADPH disappearance at 340 nm was measured. Enzyme activity was expressed in nM NADPH oxidized/min per mg protein [14].

#### Reduced glutathione (GSH)

Reduced Glutathione (GSH) was determined by aliquoting 1 ml of supernatant from the mixture of 0.5 ml of tissue homogenate and 2 ml of TCA (Trichloroacetic acid). To this 1 ml aliquote 0.5 ml of 0.0198% Ellman’s reagent in 1% sodium citrate and 3 ml of phosphate buffer (pH 8.0) were added. The colour developed was read at 412 nm. Reduced Glutathine was expressed as mmoles/g tissue [15].

#### Lipid peroxidation

Lipid peroxidation in substantia nigra pars compacta was measured by colorimetric evaluation of hydroperoxides and thiobarbituric acid reactive substances (TBARS). In brief, homogenate was treated with 1:1:1 ratio of 0.37% thiobarbituric acid (TBA), 15% trichloroacetic acid (TCA), and 0.25 N hydrochloric acid (HCl). It was followed by placing the homogenate in a water bath for 15 minutes and then being cooled and centrifuged. Clear supernatant was recorded against reference at 535nm and enzyme activity was reported in nmoles/ml of TBARS in tissue [16–19].

### Behavioural analysis

#### Akinesia

To measure Akinesia, the latency for the animals to move all 4 limbs was recorded in seconds. If the latency exceeded 3 minutes, the test was terminated. Initially, every animal was acclimatized for 5 minutes on an elevated wooden platform. We then recorded the time taken by the animal to move all the 4 limbs [20–22]. This exercise was repeated 5 times for each animal with 5-minute wait periods in between each repeat.

#### Catalepsy

To record Catalepsy, the animals were first acclimatized on a wooden platform and then placed on a flat horizontal surface with both the hind limbs placed on a cubical wooden block (3 cm high). The latency to move both the hind limbs to the ground was recorded in seconds [23,24].

#### Swim-test

Swim-test was conducted using water tubs of dimensions 40 cm length x 25 cm width x 16 cm height. The temperature was maintained at 27 ± 2°C and the depth of water was kept at 12 cm. Observations were classified in 4 different levels and a score was allotted accordingly, hind part sinks with head floating (0); occasional swimming using hind limbs while floating on one side (1); occasional floating/swimming only (2); continuous swimming (3) [21,25]. The animals were wiped dry immediately after the experiment using a dry towel and returned to cages kept at 27 ± 2°C.

#### Statistical analysis

We calculated mean and standard deviation of the data and plotted bar graphs, with error bars representing the standard deviation. We plotted data points as horizontal lines to show the actual spread of the data. MPTP treatment is labelled with a light blue solid line. The dotted line below the bar graphs is used to represent herbal extract treatment. Numbers below the bar graphs represent sample sizes.. We thoroughly studied each dataset for normality using two graphical approaches and three normality tests, Shapiro-Wilk Test, Anderson-Darling Test and Kolmogorov-Smirnov Test. Normality testing has been presented as a report in (S1 Fig). We studied significance for all combinations of datasets. For all combinations, we used the Levene’s test to determine if the two datasets have similar variances or not. If both the datasets in the combination were normal, we used a student’s t-test if the variances were similar and a Welch’s t test otherwise. If even a single dataset in the combination was non-normal, we used a Mann Whitney U Test if the variances were similar. If the variances were not similar, we ranked the data and used a Welch’s t-test on the ranked data (also called Welch’s U Test). We show significance tests between all combinations of datasets (S2 Fig). The tests were conducted and the results were plotted in python. We know that using p values for hypothesis testing is not the most statistically rigorous approach and have used it only as a proxy for estimation. We provide the p scores for each method using a new visualization approach of Complete Comparison Display (CCD) that we have developed for this paper and describe in the results section.

## Results

### Complete Comparison Display (CCD)

Several studies involve multiple comparisons in which one compares the probability of groups being similar (or different), but most studies use the comparison of one ‘control’ group with the rest of the groups. We devised a staircase like display to be able to display comparisons of all groups one by one with the rest. Detailed comparison of all groups that we call Complete Comparison Display (CCD) is given on the right hand side of each bar graph.

### Plan of the study

**Fig 1:**
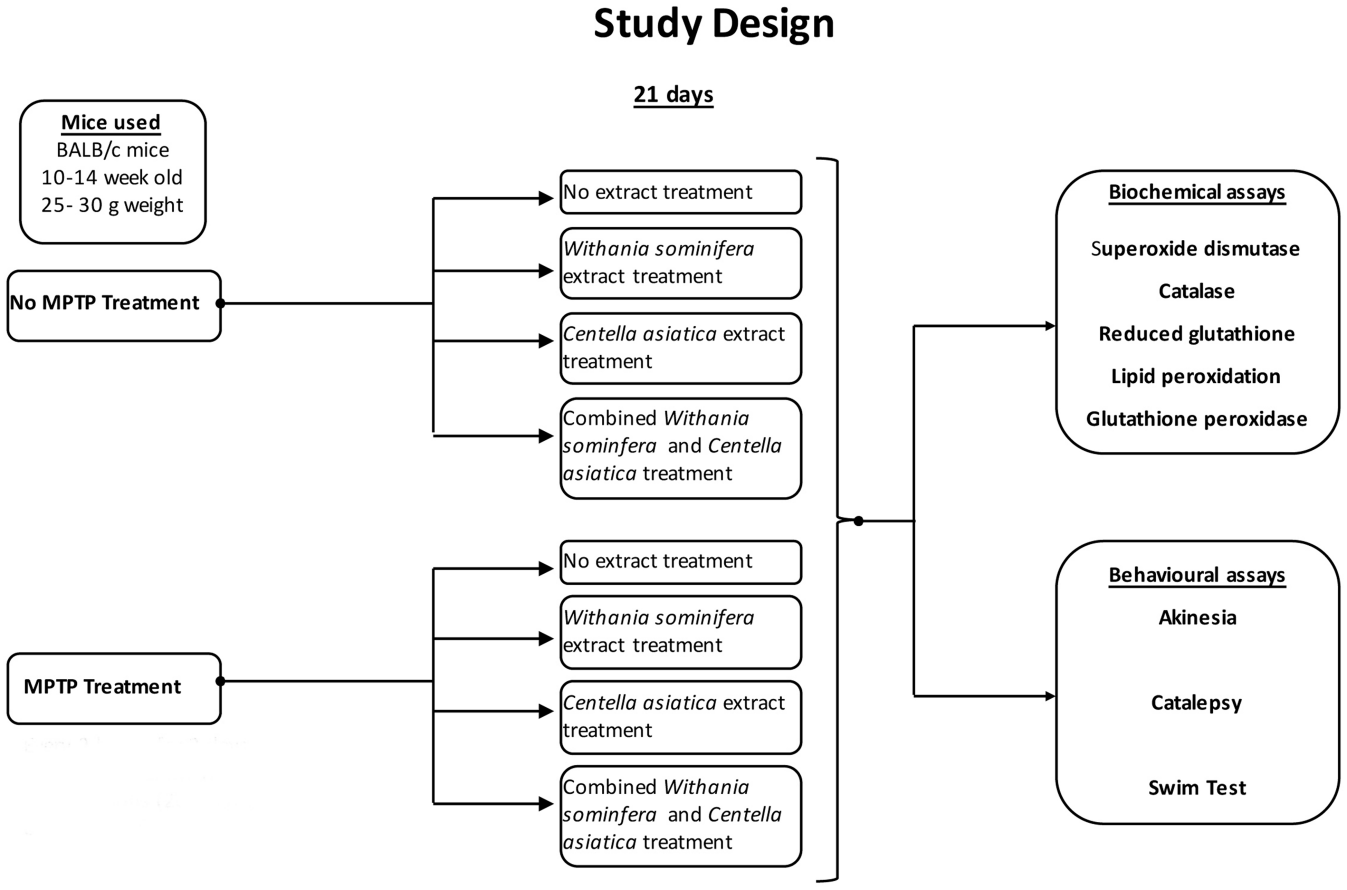
Study design. Study design showing treatments and assays.

We induced PD like symptoms in Balb/c mice using repeated intra-peritoneal MPTP injection for 2 times at an interval of 2 hours. An equal number of mice underwent saline injection. Following this treatment both groups were further split in four further groups for 21 days. One was not treated any further; two groups were treated with one herbal extract each, and one group with both the herbal extracts. Following this regimen, animals were tested for biochemical and behavioral response. The schematic (Fig 1) summarizes the scheme of the experiment. In addition to the above-mentioned scheme, some mice were also tested microscopically after 21 days to ensure that MPTP treatment had the desired effect.

### Microscopic evaluation of MPTP effect

**Fig 2:**
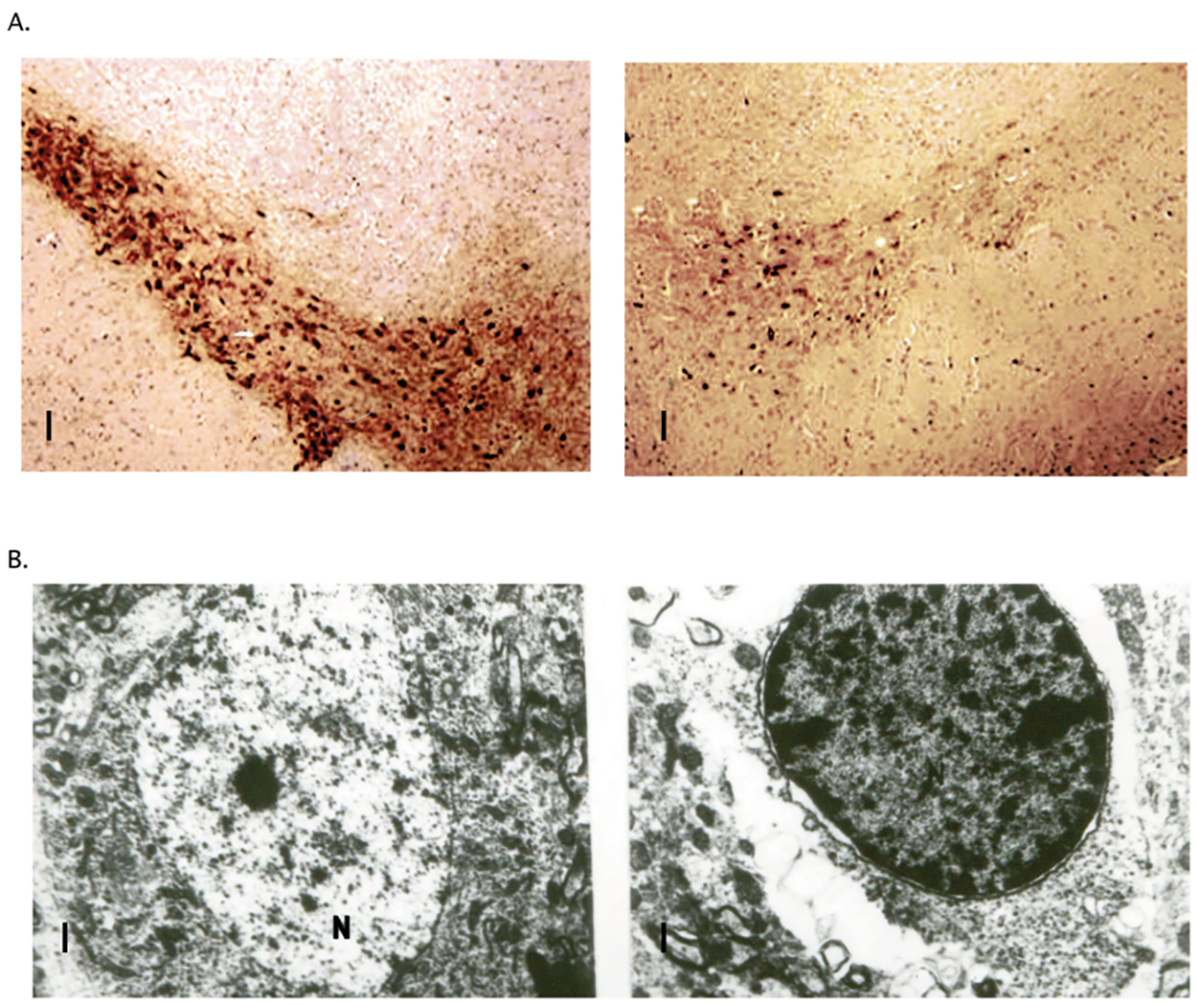
Microscopic confirmation of MPTP induced damage of Substantia Nigra. A. Light microscopic images show lower count of dopaminergic neurons in Substantia Nigra as shown by tyrosine hydroxylase staining. Left is brain slice from saline only treated animal and right is from MPTP treated animal. Scale is 100 μm. B. Electron microscopy shows damage induced in the nucleus of remaining SN dopaminergic neurons. Left is from saline treated animal and right is from MPTP treated animal. N label stands for nucleus. Scale is 1 μm.

Light microscopic examination confirmed loss of dopaminergic neurons in Substantia Nigra of MPTP treated mice (Fig 2). Electron microscopic examination showed that nuclear damage was induced due to MPTP treatment (Fig 2). We only used microscopic examination to confirm that MPTP treatment showed desired results.

### Biochemical analysis of oxidative stress

**Fig 3:**
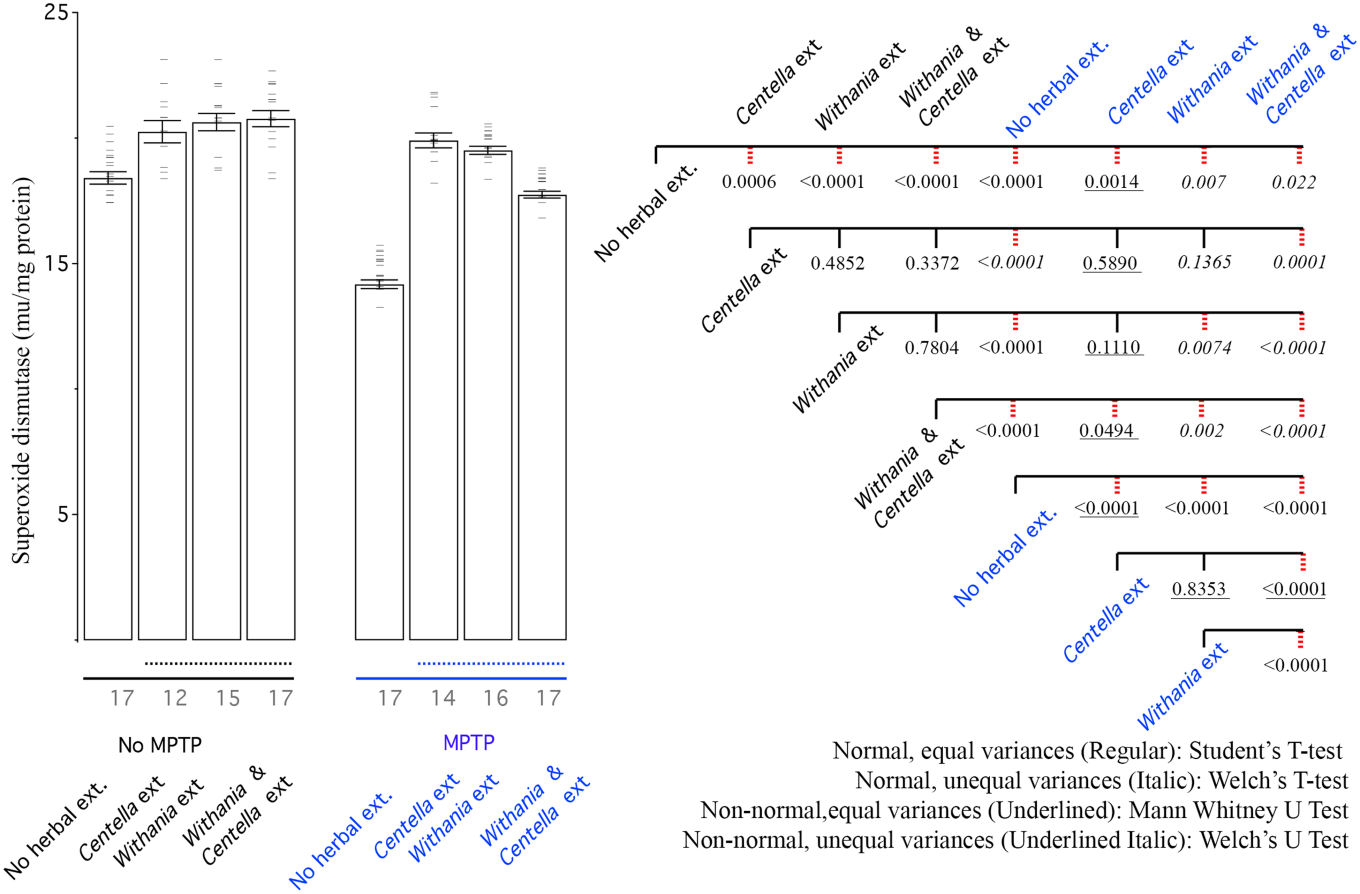
MPTP reduces SOD activity, while all herbal extracts improve it. Left: Bar graph shows mean SOD activity of all groups, where error bars depict standard deviation, horizontal bars data points. Black line show only saline treated groups and blue line shows MPTP treated groups. Dotted line shows groups that are treated with herbal extracts. Right: CCD of different groups. The three values under each branch, from top to bottom are student’s T test, Tukey’s honest significant difference and Dunnett’s two tailed T test scores. Dotted right lines reflect p values similar or lower to 0.05 in Tukey’s honest significant difference test.

**Fig 4:**
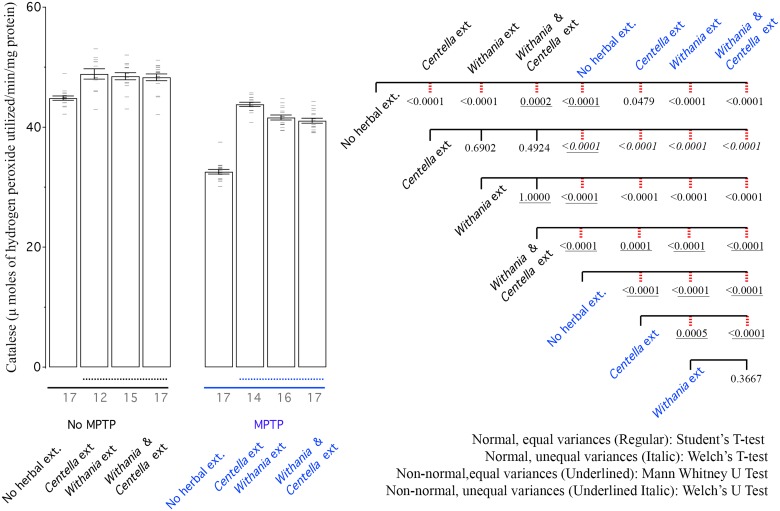
MPTP reduces catalase activity, while all herbal extracts improve it. Left: Bar graph shows mean catalase activity of all groups. Right: CCD of different groups. Labelling scheme is similar to other bar graphs.

**Fig 5:**
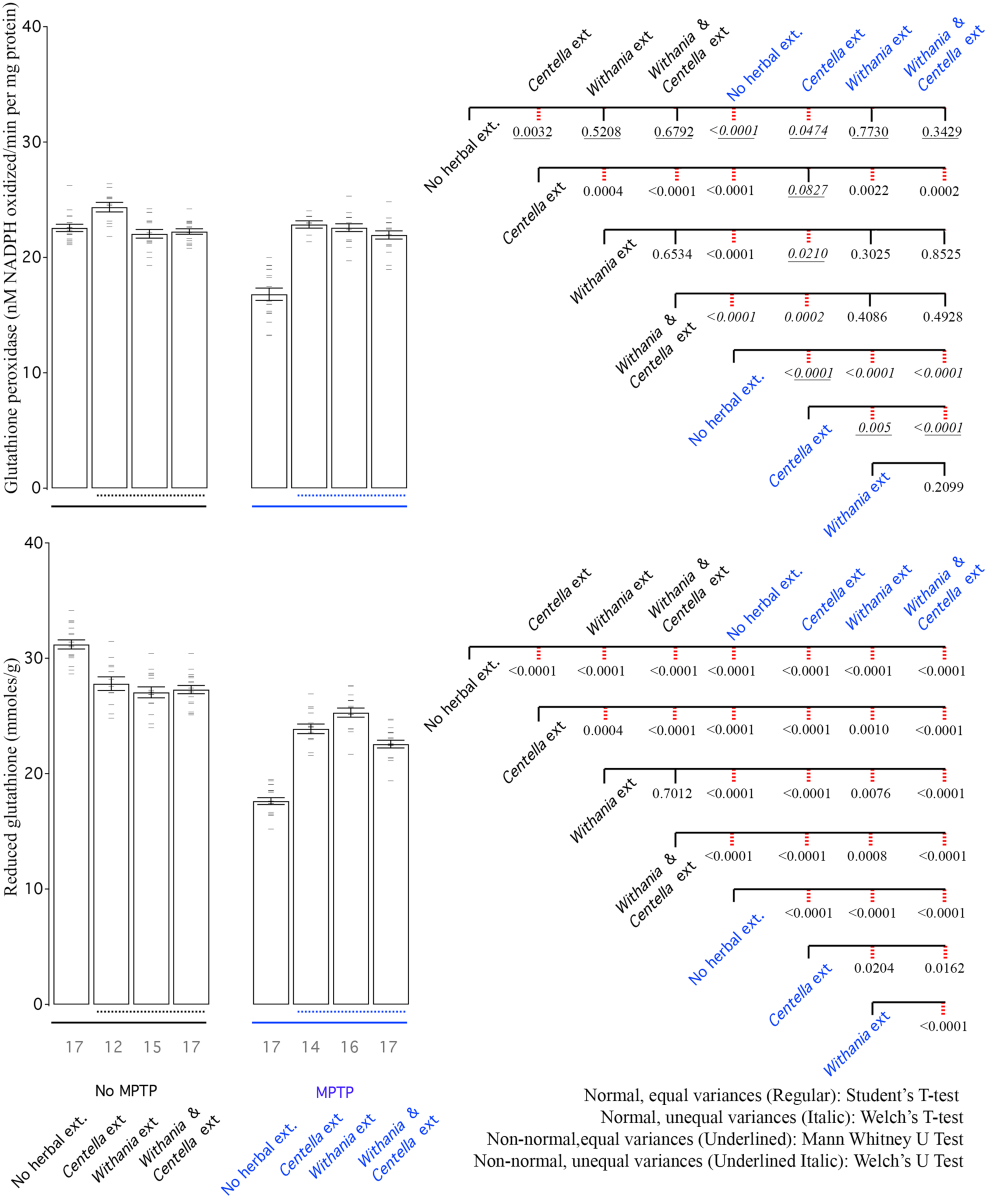
MPTP reduces both GPX and GSH levels, while all herbal extracts improve them. Herbal extracts also reduce GSH levels in saline only treated animals. Left: Bar graph shows mean GPX and GSH activity of all groups. Right: CCD of different groups. Labeling scheme is similar to other bar graphs.

**Fig 6:**
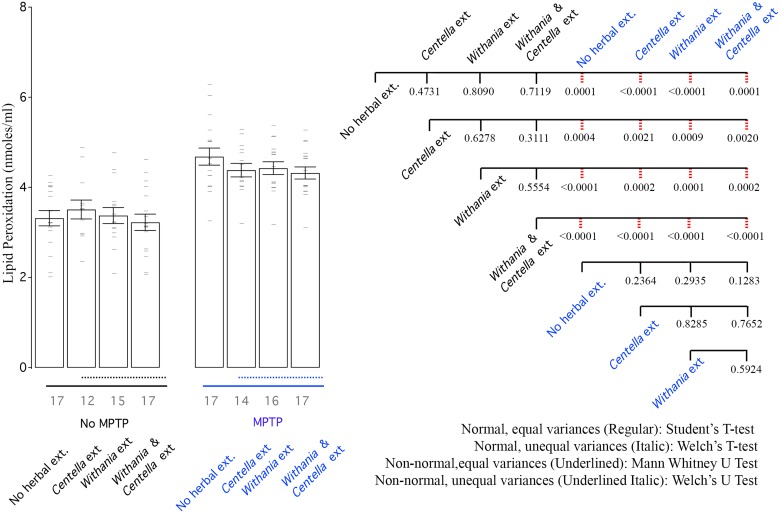
MPTP increases lipid peroxidation (LPO) and none of the herbal extracts are able to improve lipid peroxidation levels. Left: Bar graph shows mean LPO activity of all groups. Right: CCD of different groups. Labelling scheme is similar to other bar graphs.

Summarily, in some *Centella asiatica* and *Withania somnifera* treated groups, the antioxidant activity was strong enough to show improvement over saline treatment (Figs 3–6). The combination of the two was never better than that of the single extract, which showed superior activity (Figs 3–6). Oxidative stress always increased as a result of MPTP treatment (Figs 3–6). In the case of lipid peroxidation there was no strong improvement in oxidative stress profile due to herbal extracts (Fig 6). Herbal extracts could mediate recovery from oxidative stress almost back to normal in several cases (Figs 3–5).

### Superoxide dismutase (SOD)

Superoxide dismutase catalyzes the conversion of the superoxide radical into either hydrogen peroxide or ordinary molecular oxygen. It is vital to combat oxidative stress. On non-MPTP treated animals any of the herbs and their combination can increase the activity of SOD to above baseline levels, showing a promise for other medical application in addition to PD. MPTP induced large decrease in SOD activity in SN region. There was recovery from either of the two herbal extracts and the combination but surprisingly, the recovery from the combination was lower either of the two herbal extracts (Fig 3). The recovery from either of the two herbs alone was strong enough to reach herbal treatment in no-MPTP group. In fact SOD activity in mice given *Centella asiatica* in MPTP treated group was even better than the saline treated group (Fig 3).

### Catalase

Catalase is a common enzyme that is responsible for decomposition of hydrogen peroxide into hydrogen and water. It is interesting that both the herbs individually or in combination are able to increase breakdown of H_2_O_2_ (Fig 4), suggesting possible tonic uses in non-Parkinsonian patients and use in patients suffering from high oxidative stress, such as in the case of diabetes. The increase due to either of the herbs or combination was comparable (Fig 5). MPTP treatment reduced the breakdown of H_2_O_2_ but both herbs and the combination of two helped in recovery (Fig 4).

### Glutathione peroxidase (GPx)

The glutathione peroxidase enzymes are responsible for reducing lipid hydroperoxides to their corresponding alcohols and for converting free hydrogen peroxide to water. Our assay does not discriminate between the specific GPx but only measures overall GPx activity in SN region. *Centella asiatica* treatment increased GPx activity above saline only levels, while *Withania somnifera* and the combination did not increase the GPx activity in saline only group (Fig 5). MPTP treatment dropped GPx activity, which was largely recovered by any of the 3 herbal extract treatments (Fig 5).

### Reduced glutathione (GSH)

Quite surprisingly, treatment of either of the two herbal extracts and the combination reduced the amount of reduced glutathione levels below saline only levels (Fig 5). This could be simply due to up-regulation of GPx or homeostatic compensatory mechanism to counter increased anti-oxidant response. MPTP treatment decreased GSH to almost half the levels of saline only treatment (Fig 5). All the herbal extracts treatments improved GSH levels in MPTP treated mice but were not close to saline only levels and lower than saline treated mice receiving herbal extracts (Fig 5). Within MPTP treated animals, either of the 2 extracts worked better than the combination of the 2 herbal extracts (Fig 5).

### Lipid peroxidation (LPO)

Lipid peroxidation is highly damaging to cells and is a cyclic reaction. Several anti-oxidants mechanisms contribute to termination of the cyclic reaction and limit damage. None of the herbal extracts contributed to change of LPO levels within the saline treatment alone animals (Fig 6). MPTP exposure increased LPO levels in SN and the herbal treatments were incapable of making any reduction in the LPO levels (Fig 6).

### Behavior

**Fig 7:**
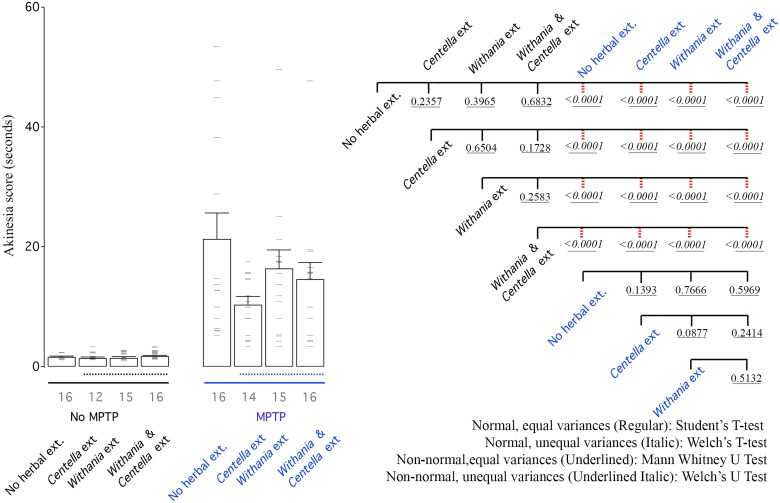
MPTP increases akinesia. Left: Bar graph shows mean akinesia activity of all groups. Right: CCD of different groups. Labelling scheme is similar to other bar graphs.

**Fig 8:**
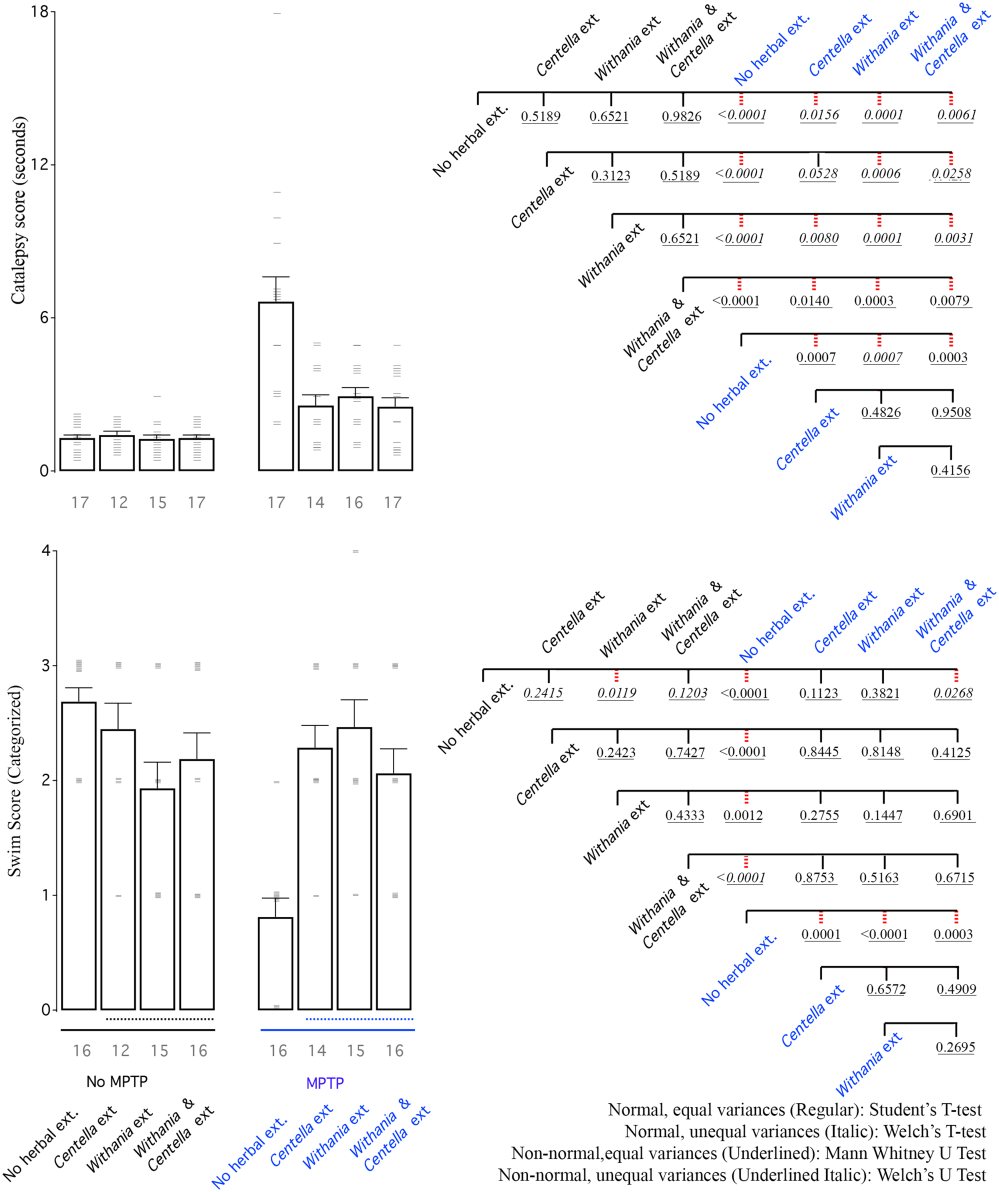
MPTP increases catalepsy scores and decreases swimming scores. All herbal extracts are able to show improvement in MPTP treated groups. Left: Bar graph shows mean akinesia activity of all groups. Right: CCD of different groups. Labelling scheme is similar to other bar graphs.

In summary, akinesia testing treatment with *Centella asiatica* extract showed the most improvement followed by other groups (Fig 7). All 3 treatments showed significant improvement in catalepsy and swim scores compared to mice treated only with MPTP (Fig 8). Here too the combination did not produce superior results when compared that of the single extract, which showed superior activity.

#### Akinesia

Impaired ability to initiate movements is termed as akinesia. Within saline treated animals, herbal extract treatment made no measurable difference in latency of animals to respond. Latency to move all limbs was increased after treatment with MPTP (Fig 7). Although a trend was observed in the direction of behavioural improvement but no herbal extract could rescue akinesia deficits significantly, for the sample size of our study (Fig 7).

#### Catalepsy

Catalepsy is inability to correct an externally induced posture of hind limbs. Within saline treated group the herbal extracts did not elicit any measurable improvement (Fig 8). MPTP administered animals exhibited significant increase in catalepsy symptoms. Treatment with herbal drug exhibited large reduction in symptoms (Fig 8).

#### Swim-test

Swim test within saline treated animals was largely the same except *Withania somnifera* extracts resulted in reduced swimming performance (Fig 8). There was a drastic reduction in swimming abilities of animals due to MPTP treatment and all three herbal extract treatments were able to show large recovery (Fig 8). Within the herbal groups, whether in saline only or MPTP treated animals there was no difference (Fig 8).

To summarize, both *Centella asiatica* and *Withania somnifera* were useful in behavioural and biochemical recovery from MPTP induced PD but the combination of the two never outperformed the better of the two herbal extracts (Figs 3–8). This analysis provides a nuanced comparison of different groups and a detailed analysis.

## Discussion

Levodopa is to date the most effective symptomatic treatment of PD. Its works by crossing the blood brain barrier and entering the central nervous system where it is converted into Dopamine by the action of L-amino acid decarboxylase also known as DOPA decarboxylase [26]. Levodopa use does however have side effects with links to depression, insomnia, agitation, and anxiety [27–30]. Also, while levodopa ameliorates many of the motor symptoms of PD, the non-motor symptoms are hardly affected [31]. It is also not able to stop the degeneration of dopaminergic nigral neurons. Long-term levodopa treatment is associated with adverse motor effects partly due to tolerance including the ‘on-off’ phenomena, wearing off, dose failure, akinesia [32] and dyskinesias [33]. Strategies to alleviate these symptoms include use of slow-release formulations, catechol-O-methyl transferase (COMT) inhibitors, anticholinergics, dopamine agonists or monoamine oxidase-B (MAO-B) inhibitors [34]. However all other auxiliary treatments also suffer from several side-effects. These range from confusion, drowsiness, agitation, and hallucinations for anticholinergics to psychiatric disturbances, and cardiovascular problems with dopamine agonist use [35]. Thus the use of herbal therapeutics for PD is the need of the hour. This is especially true since increasing life spans means that long-term PD treatment regimens are required that minimize side effects.

To explore alternative medicines that can supplement or replace existing ones, we tested herbal extracts in MPTP induced PD mice. Nigrostriatal DA pathway is damaged by 1-methyl-4-phenyl- 1,2,3,6-tetrahydropyridine (MPTP) and a significant number of DA neurons are lost in the striatum and Substantia Nigra [36, 37]. MPTP is a lipophylic precursor of 1-methyl-4-phenylpyridinium (MPP+). MPP+ is neurotoxic and has affinity for plasma membrane dopamine, norepinephrine, and serotonin transporters [38, 39]. It enters mitochondria utilizing mitochondrial transmembrane potential and blocks the electron transport chain by inhibiting Mitochondrial Complex I [40]. Even though, mice treated with MPTP do not exhibit typical PD behaviour, some alterations might be observed in the motor movement where dopaminergic neuron are lost significantly [39, 41].

Our study confirms the potential of *Withania somnifera* and *Centella asiatica* and finds that there is no additive effect of the two. This is likely due to presence of same active ingredients or ingredients that act on the same pathway or counteraction of synergistic action with antagonistic action of active ingredients of the two. *Withania somnifera* is used as anti-inflammatory and antitumor agent, immunity booster, and aphrodisiac in Ayurveda. Studies in animal models have shown that the herbal extract possesses antioxidant properties [42], anti-stress activity [16, 18]. Studies have also explored the neuroprotective role of the herbal extract [7, 9, 43]. Various parts of the *Centella asiatica* plant are considered useful in diseases related to skin, blood and nervous system in ayurveda. *Centella* possesses a potent antioxidant effect due to the presence of triterpenes, flavonoids, and selenium [44]. In animal studies *Centella* has shown use in improving learning scores [45, 46], neurodegeneration protection [47], oxidative stress reduction [48,49]. A *Centella* regimen reduced anxiety levels, mental fatigue rate, and improved immediate memory in patients with generalized anxiety disorder [50]. *Centella* attenuates d-galactose- induced behavioural, biochemical, and mitochondrial dysfunction in senescent mice [51]. *C*. *asiatica* extract has also been reported to have anti-inflammatory, anti-ulcer, sedative, cardioprotective, anti-diabetic, and anti-bacterial [52–57] activities. It is believed that triterpenes and saponins are the biologically active components of *Centella* plant [58]. The triterpenes in *Centella* are made of a number of compounds including asiatic acid, centic acid, madecassoside, brahmoside, madasiatic acid, madecassic acid, isothankunisode, asiaticosside, brahmic acid, brahminoside, thankiniside, centelloside, and cenellic acid [59]. *C*. *asiatica* contains triterpene glycosides such as saponin, asiaticoside, madecassoside, and sceffoleoside [60]. Location and diverse environmental conditions can affect the content of *Centella*’s triterpene components [61]. *Centella* also has high total phenolic content in the form of flavonoids like apigenin, quercetin, catechin, kaempherol, rutin, and naringin and volatile oils like farnesol, caryophyllene, and elemene [59]. To contribute to the neuroprotective effect of herbs and herbal extracts, several mechanisms have been proposed. Some of these include their function as inhibitors of monoamine oxidase B to decrease neurotoxicity, antioxidants to alleviate oxidative stress, chelators of harmful metals, scavengers of free radicals, modulators of cell survival genes and apoptotic signals. The combined antioxidant and anti-apoptotic effects and possible cross-talk between the two is responsible for at least part of its protective effects.

We observed a reduction in reduced glutathione levels in saline treated animals, when exposed to the herbal extracts (Fig 5). This could be due to increase in GPx utilizing GSH as substrate or due to homeostatic mechanisms. Several studies utilizing herbal extracts have measured glutathione levels [62–65]. However, these do not take in to account the impact of the herbal extract on glutathione levels in mice not treated to exhibit the disorder phenotype. Thus any glutathione modulatory roles the extract might have under normal conditions, is not reported. This needs to be investigated further. LPO levels (Fig 6), suggest that neither of the two tested herbal extracts has any influence on it.

To measure dopamine-induced motor deficits various behavioral tests are suggested rodents models of PD. If animals are less dependent on all four limbs balance its easier to characterize tremor and rigidity. Behavioral test performed in the present study are subtler and mimics tremor and rigidity as seen in seen in PD patients. Open Field test, which is suggested, is learning skill test. Innate motor skills/abilities of animals that are dopamine dependent are measured by various test methods like rotarod, grid test, inclined beam traversal, climbing down a pole, reaction-time test, staircase test, adhesive removal and nesting behavior. Most of these measures require the animal to first learn a complex task first. Failure in performing these complex tasks does not show it is because of motor deficit or from a learning deficit. One drawback with these measures is the inability of all animals to learn these complex tasks even before receiving the dopamine lesion. They are thus often excluded from the results.

Treatment with *Withania somnifera* can reverse the alterations in behavioural deficits in 6-hydroxy dopamine-induced parkinsonic rats via an unknown mechanism [9], Reports suggest ethanolic extract of *Withania somnifera* increases the striatum dopamine content and attenuates the effects of MPTP induced parkinsonism in rats [8]. Cantella significantly restores dopamine and attenuate changes in oxidative alterations, and also corrects behavioural deficits [5]. Similar observations were also made by us both the herbs have the capacity of resorting dopamine levels after MPTP treatment. Both *Withania* and *Centella* restored tyrosine hydroxylase activity (Data not presented).

In summary, our study devices novel data visualization approach for multiple comparisons and shows a lack of synergism of the two herbal extracts. We expect future work in the field to be on finding several other suitable extracts and combination of extracts, finding active molecules in the extracts and conducting clinical studies. In addition to the few behavioral tests we have used, researchers can also use several others to investigate PD symptoms. We were able to integrate light and electron microscopy, biochemical work and animal behavioral assays and in future expect to integrate various omics approaches, more biochemical assays and more behavioral work, taking these screens forward. We hope that **C**omplete **C**omparison **D**isplay (CCD) would help researchers across various branches of science dealing with multiple groups.

## Supporting information

S1 Fig Normality testing of each data sets. FiguEach dataset was tested for normality using two graphical approaches and three normality tests, Shapiro-Wilk Test, Anderson-Darling Test and Kolmogorov-Smirnov Test.
(PDF)

S2 Fig Significance tests between combinations of datasets. Levene’s test was used to determine if the two datasets have similar variances. When both datasets were normal, we used a student’s t-test if the variances were similar and a Welch’s t test otherwise to test significance. For one or both non-normal datasets being compared with similar variance, we used a Mann Whitney U Test. If the variances were not similar for non-normal datasets we used Welch’s U Test.
(PDF)

S1 Table Experimental values of each individual treatment. This pdf has tables that present value of each single biochemical and behavioural measurement.
(PDF)
