# Supplementary material for: Complete Comparison Display (CCD) evaluation of ethanol extracts of Centella asiatica and Withania somnifera shows that they can non-synergistically ameliorate biochemical and behavioural damages in MPTP induced Parkinson's model of mice
Source: PLoS One. 2017 May 16;12(5):e0177254. doi: 10.1371/journal.pone.0177254 (PMC5433711; doi:10.1371/journal.pone.0177254)
Supplement: S1 Fig — FiguEach dataset was tested for normality using two graphical approaches and three normality tests, Shapiro-Wilk Test, Anderson-Darling Test and Kolmogorov-Smirnov Test. (PDF) [file pone.0177254.s001.pdf]

Normality Tests of Datasets

All the data sets that were obtained from the study were tested for normality so that a suitable test could be chosen for significance testing. Three tests of normality were chosen and a data set had to pass all the three tests to be considered normal. Shapiro-Wilk test (SW) was the most powerful test among the three followed by Anderson-Darling test (AD) and the Kolmogorov-Smirnov Test (KS). A confidence of 95% was chosen when reporting a test to be non-normal. Critical Values are also reported along with the test statistics. Please note that for SW test the test statistic should be greater than the critical value for the dataset to be normal while for AD test and KS test the test statistic should be lesser than the critical value for the dataset to be normal. Visually, the normality of the dataset has been shown with a histogram of the data along with the estimated distribution line (Figure 1 to 64). All the datasets that were found to be normal are plotted in blue while those which were not found to be normal are plotted in red. A QQ-plot for every dataset has also been given. Further, a table has been presented that shows the skewness, the kurtosis and the tests results for every data set together.

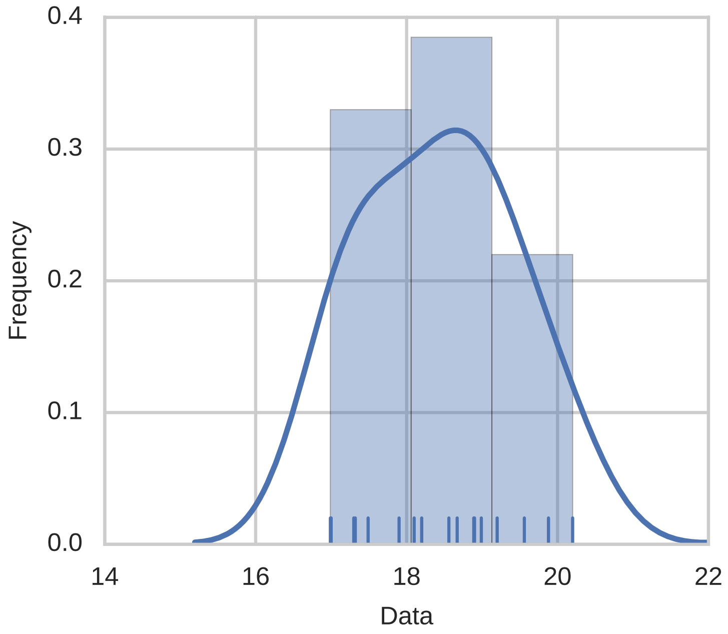

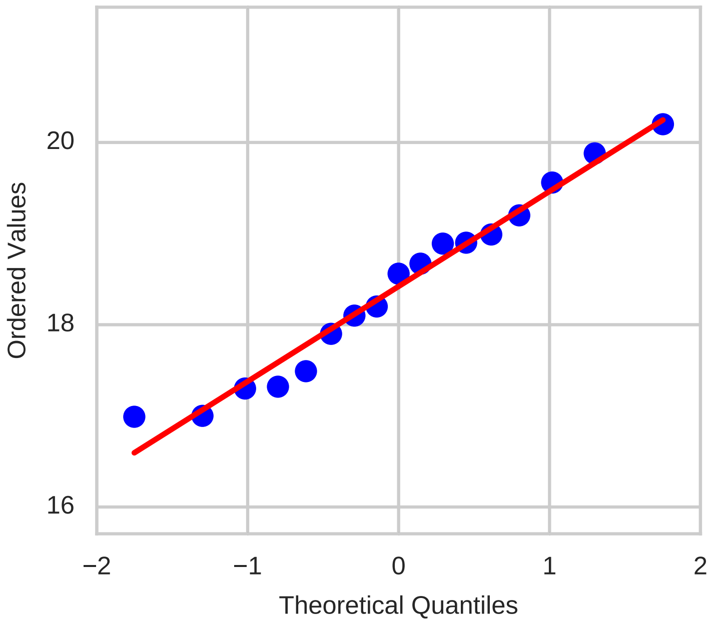

Figure 1: Superoxide dismutase levels for the untreated group (SOD_U): The data set was found to be normal. The Shapiro Wilk Test-Statistic was 0.957 (Critical Value: 0.892, passed). The Anderson-Darling Test Static was 0.237 (Critical Value: 0.685, passed). The Kolmogorov-Smirnov Test Statistic was 0.126 (Critical Value: 0.318, passed).

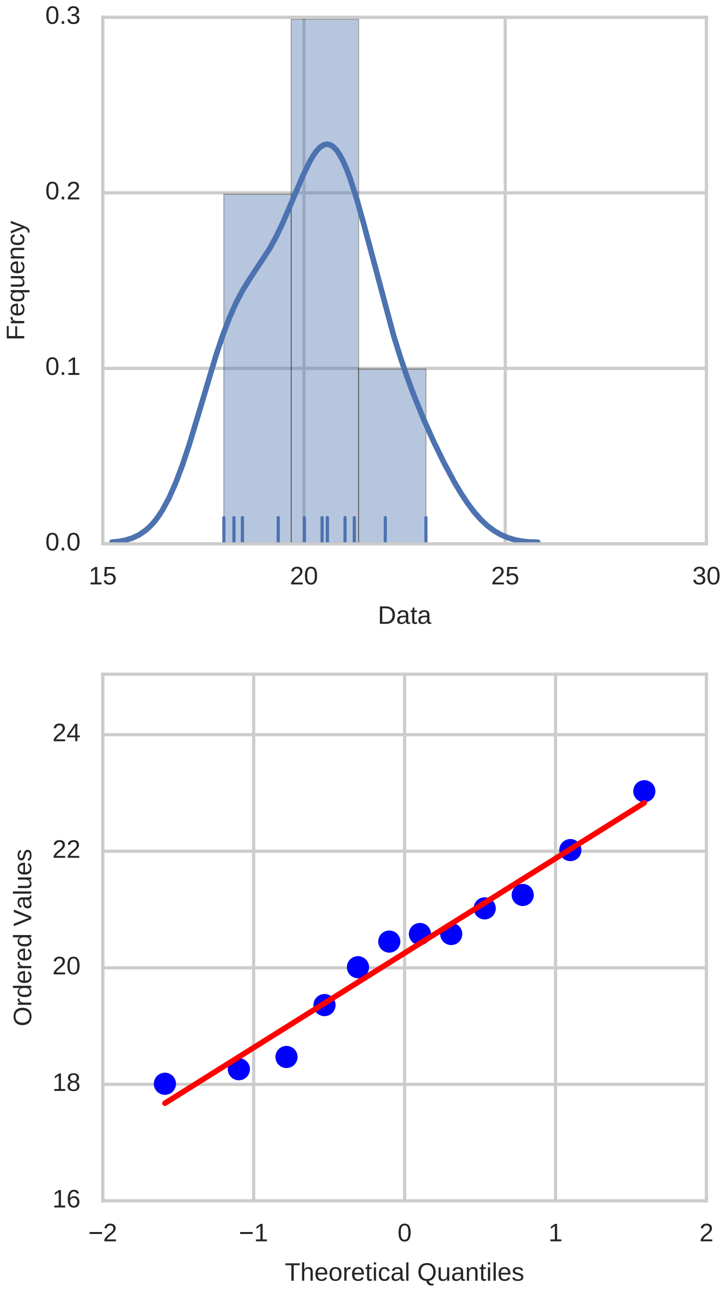

Figure 2: Superoxide dismutase levels for the group treated with *Centella asiatica* (SOD_C): The data set was found to be normal. The Shapiro Wilk Test-Statistic was 0.961 (Critical Value: 0.859, passed). The Anderson-Darling Test Static was 0.231 (Critical Value: 0.679, passed). The Kolmogorov-Smirnov Test Statistic was 0.138 (Critical Value: 0.375, passed).

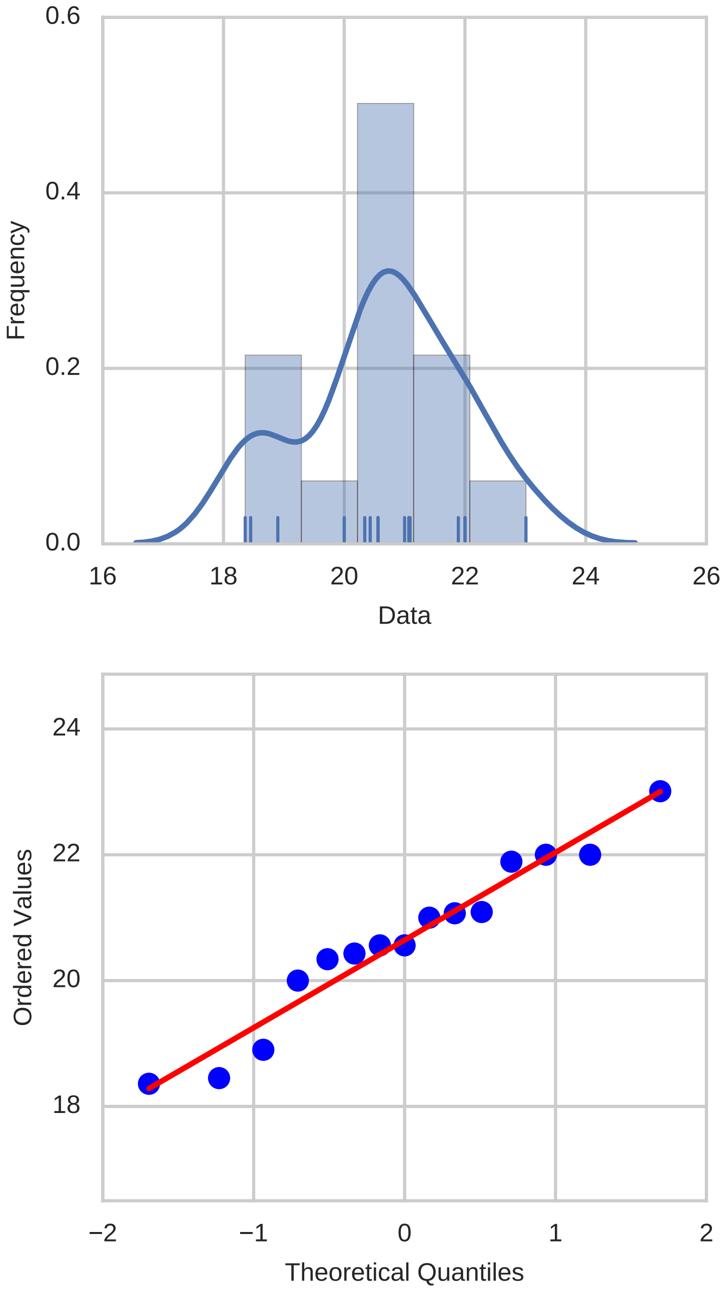

Figure 3: Superoxide dismutase levels for the group treated with *Withania somnifera* (SOD_W): The data set was found to be normal. The Shapiro Wilk Test-Statistic was 0.951 (Critical Value: 0.881, passed). The Anderson-Darling Test Static was 0.338 (Critical Value: 0.681, passed). The Kolmogorov-Smirnov Test Statistic was 0.140 (Critical Value: 0.338, passed).

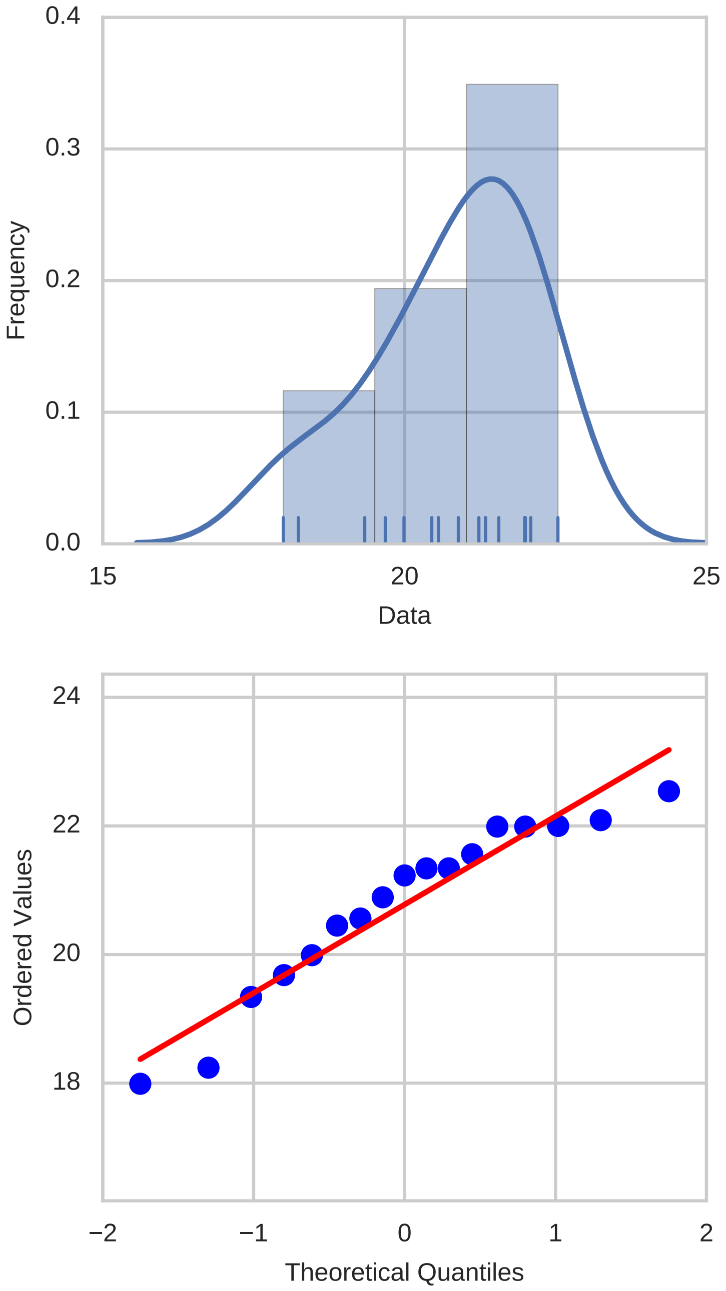

Figure 4: Superoxide dismutase levels for the group treated with both *Withania somnifera* and *Centella asiatica* (SOD_WC): The data set was found to be normal. The Shapiro Wilk Test-Statistic was 0.917 (Critical Value: 0.892, passed). The Anderson-Darling Test Static was 0.511 (Critical Value: 0.685, passed). The Kolmogorov-Smirnov Test Statistic was 0.165 (Critical Value: 0.318, passed).

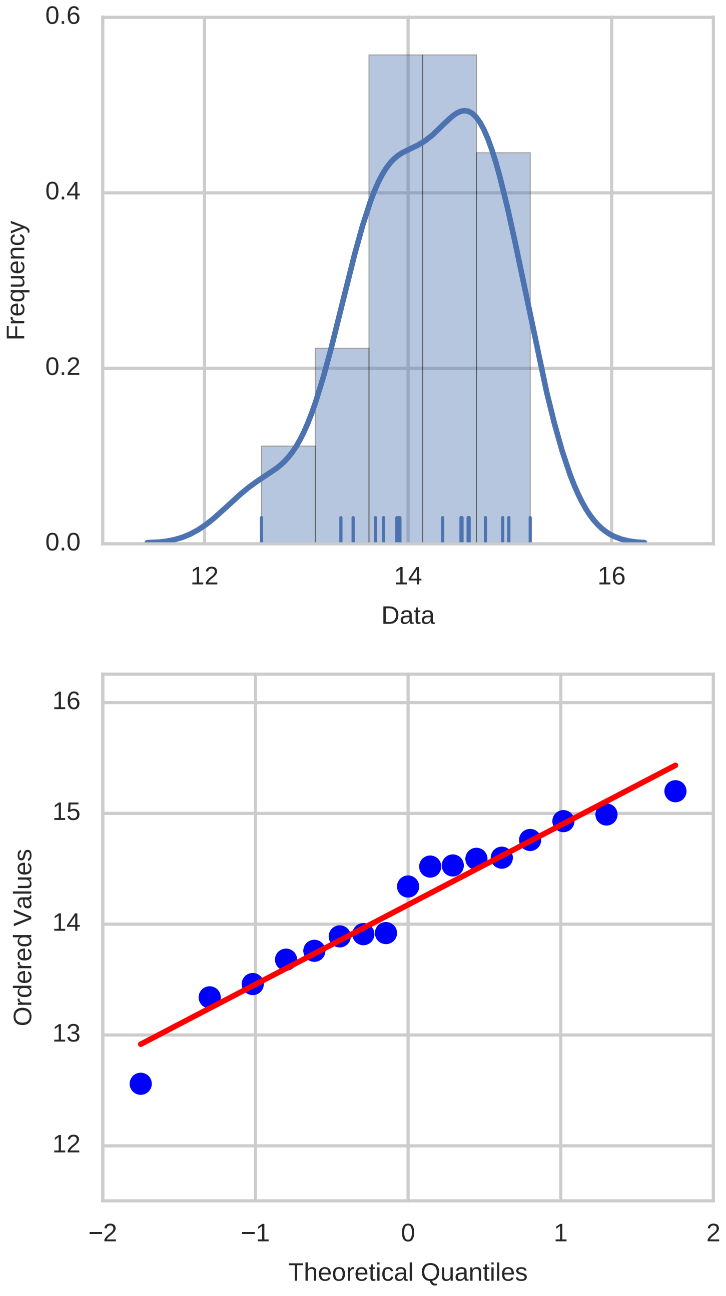

Figure 5: Superoxide dismutase levels for the MPTP disease induced and no treatment group (SOD_M): The data set was found to be normal. The Shapiro Wilk Test-Statistic was 0.955 (Critical Value: 0.892, passed). The Anderson-Darling Test Static was 0.319 (Critical Value: 0.685, passed). The Kolmogorov-Smirnov Test Statistic was 0.166 (Critical Value: 0.318, passed).

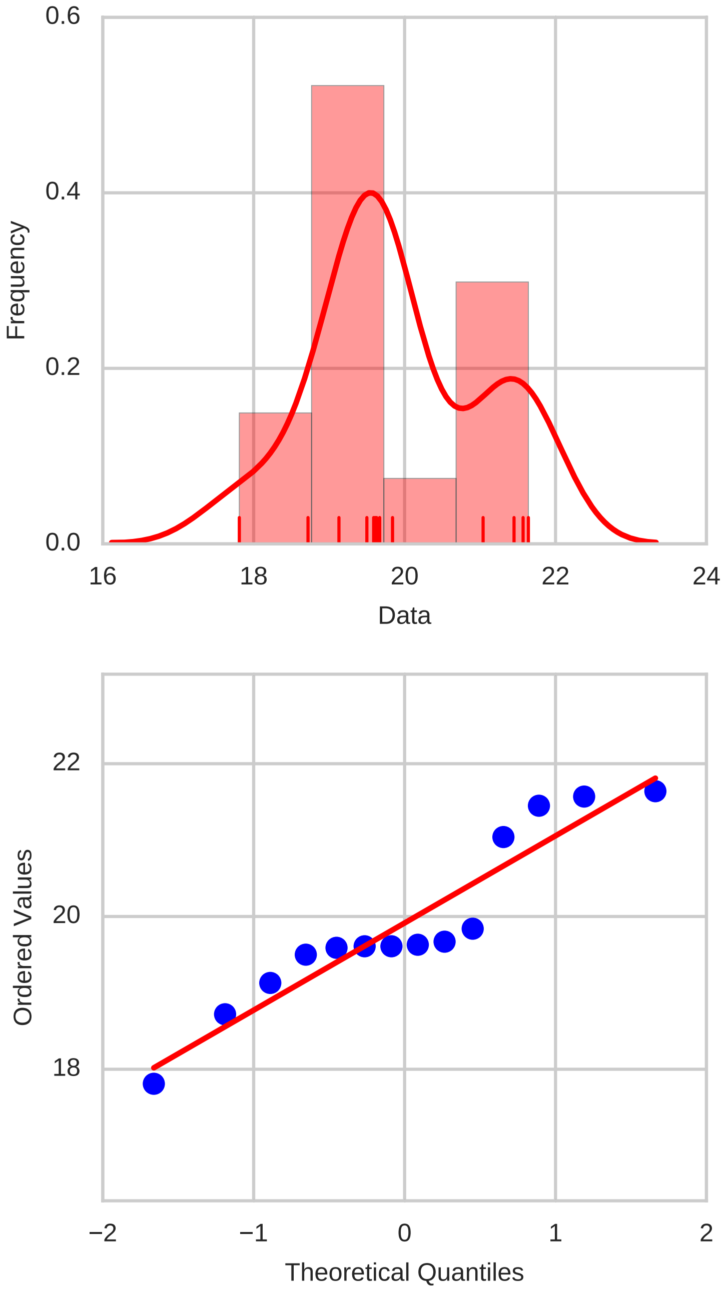

Figure 6: Superoxide dismutase levels for the MPTP disease induced and treated with *Centella asiatica* group (SOD_MC): The data set was found to be non-normal. The Shapiro Wilk Test-Statistic was 0.898 (Critical Value: 0.874, passed). The Anderson-Darling Test Static was 0.756 (Critical Value: 0.680, failed). The Kolmogorov-Smirnov Test Statistic was 0.242 (Critical Value: 0.349, passed).

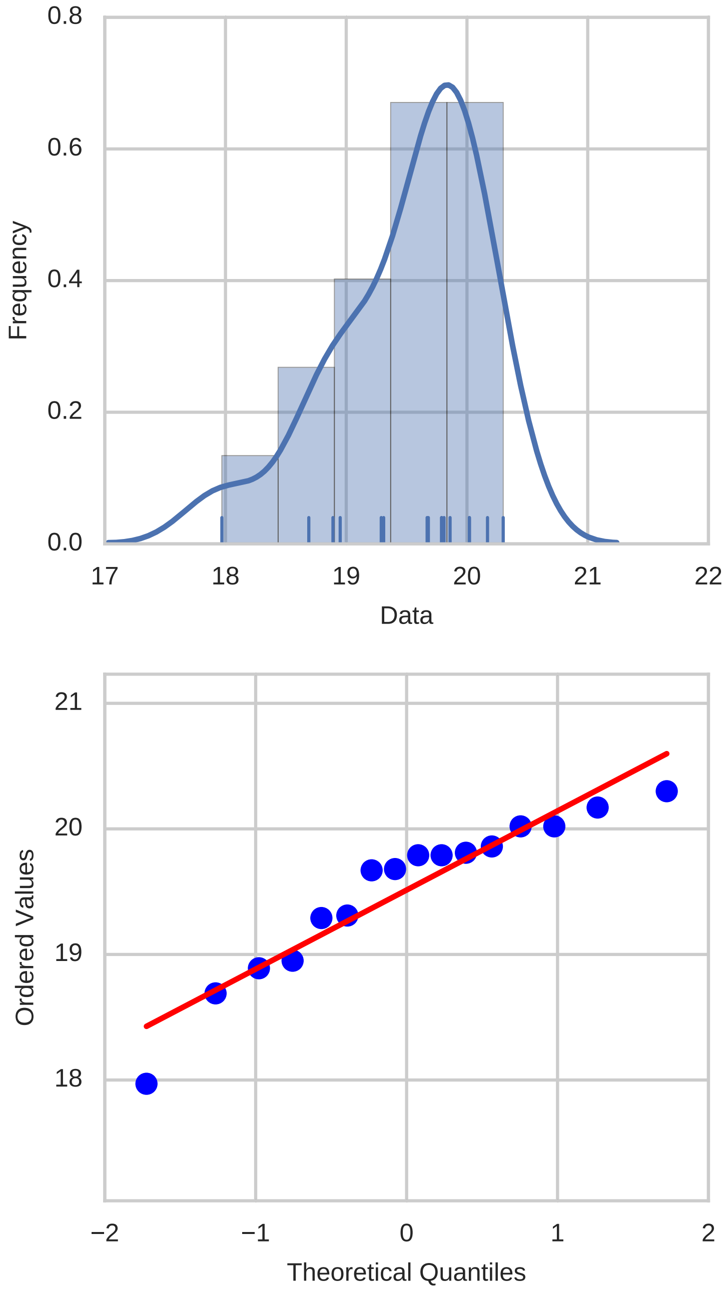

Figure 7: Superoxide dismutase levels for the MPTP disease induced and treated with *Withania somnifera* group (SOD_MW): The data set was found to be normal. The Shapiro Wilk Test-Statistic was 0.906 (Critical Value: 0.887, passed). The Anderson-Darling Test Static was 0.600 (Critical Value: 0.683, passed). The Kolmogorov-Smirnov Test Statistic was 0.227 (Critical Value: 0.327, passed).

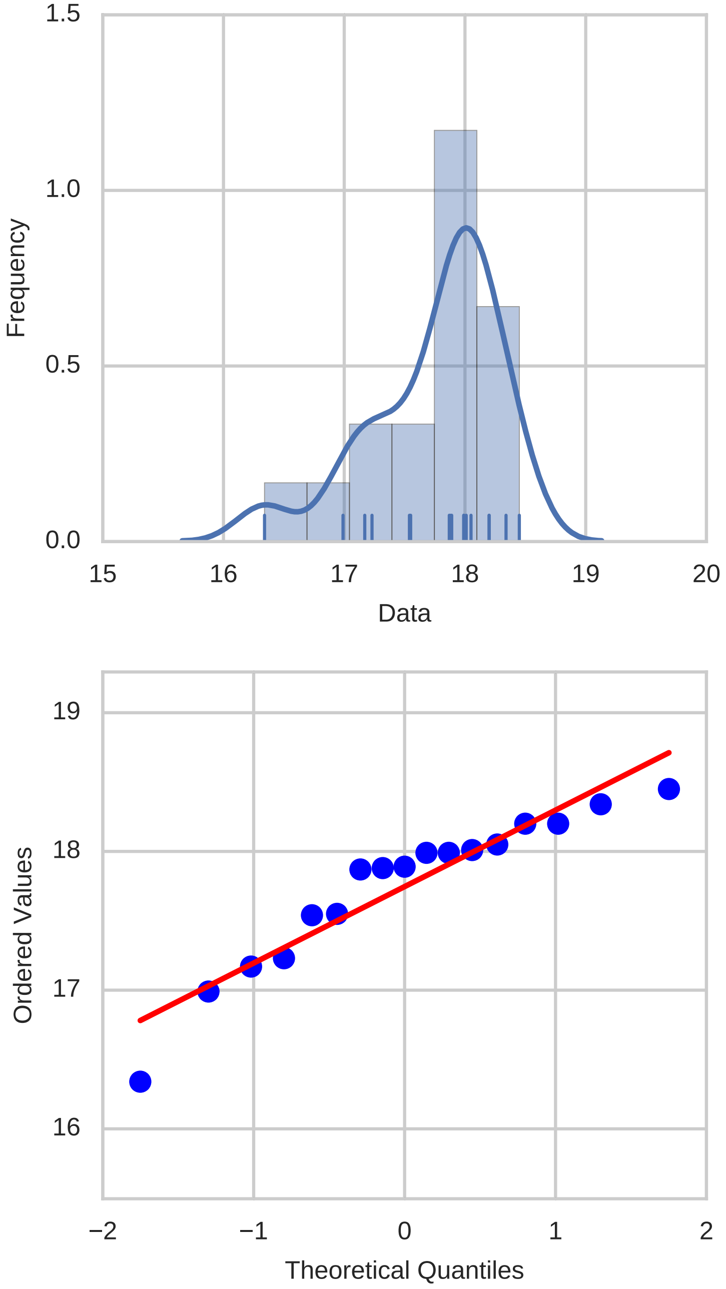

Figure 8: Superoxide dismutase levels for the MPTP disease induced and treated with both *Withania somnifera* and *Centella asiatica* group (SOD_MWC): The data set was found to be normal. The Shapiro Wilk Test-Statistic was 0.902 (Critical Value: 0.892, passed). The Anderson-Darling Test Static was 0.659 (Critical Value: 0.685, passed). The Kolmogorov-Smirnov Test Statistic was 0.239 (Critical Value: 0.318, passed).

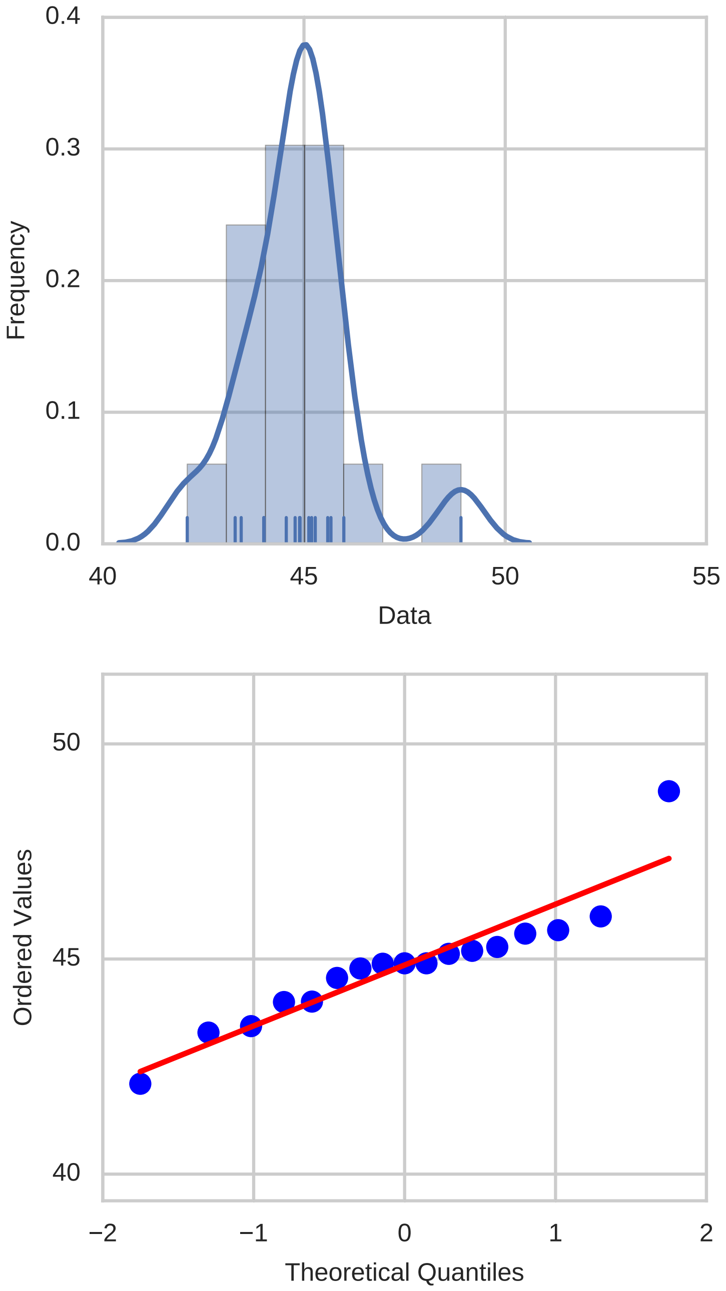

Figure 9: Catalase levels for the untreated group (CAT_U): The data set was found to be normal. The Shapiro Wilk Test-Statistic was 0.900 (Critical Value: 0.892, passed). The Anderson-Darling Test Static was 0.672 (Critical Value: 0.685, passed). The Kolmogorov-Smirnov Test Statistic was 0.162 (Critical Value: 0.318, passed).

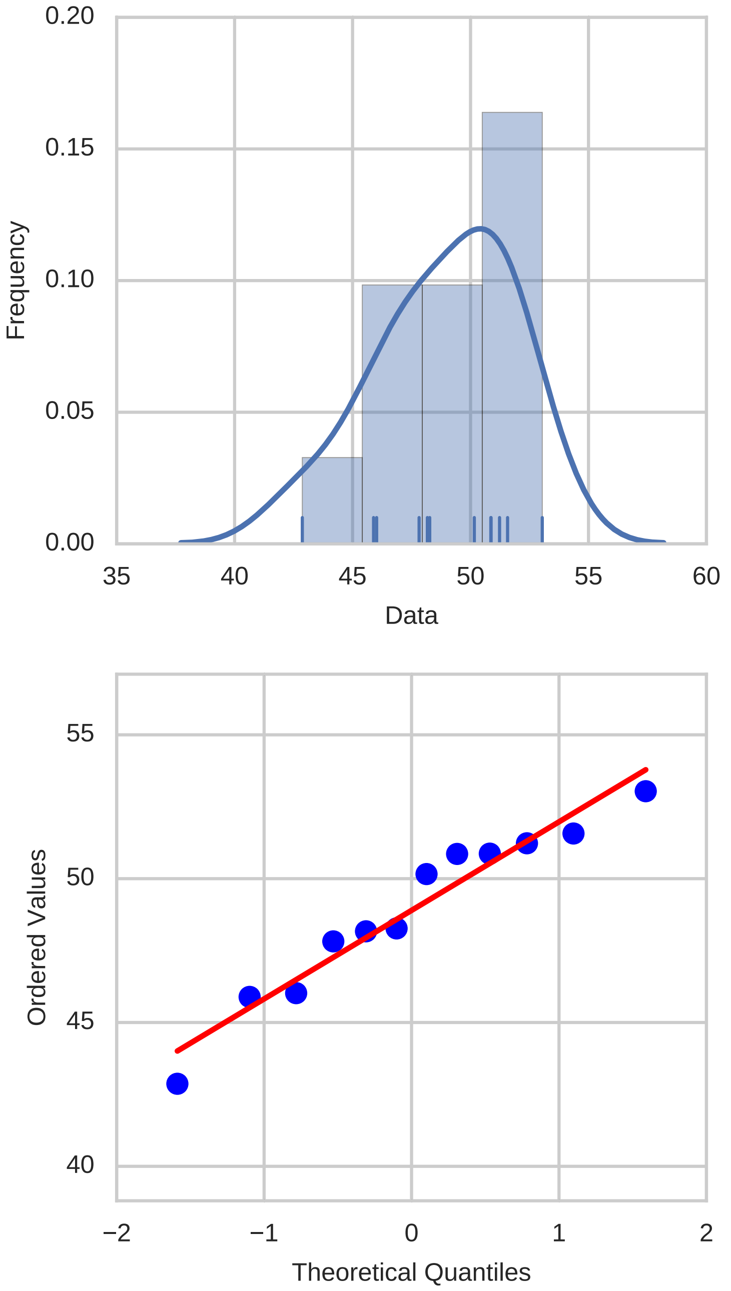

Figure 10: Catalase levels for the group treated with *Centella asiatica* (CAT_C): The data set was found to be normal. The Shapiro Wilk Test-Statistic was 0.946 (Critical Value: 0.859, passed). The Anderson-Darling Test Static was 0.330 (Critical Value: 0.679, passed). The Kolmogorov-Smirnov Test Statistic was 0.174 (Critical Value: 0.375, passed).

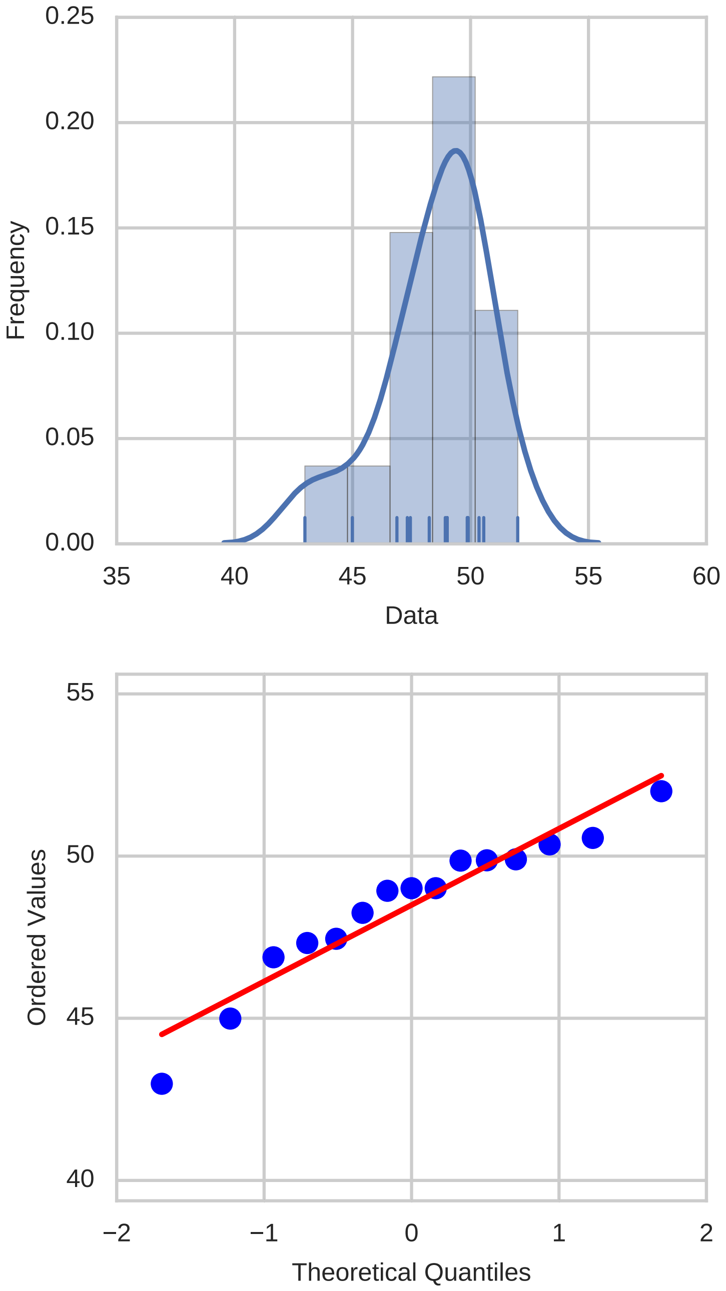

Figure 11: Catalase levels for the group treated with *Withania somnifera* (CAT_W): The data set was found to be normal. The Shapiro Wilk Test-Statistic was 0.930 (Critical Value: 0.881, passed). The Anderson-Darling Test Static was 0.464 (Critical Value: 0.681, passed). The Kolmogorov-Smirnov Test Statistic was 0.178 (Critical Value: 0.338, passed).

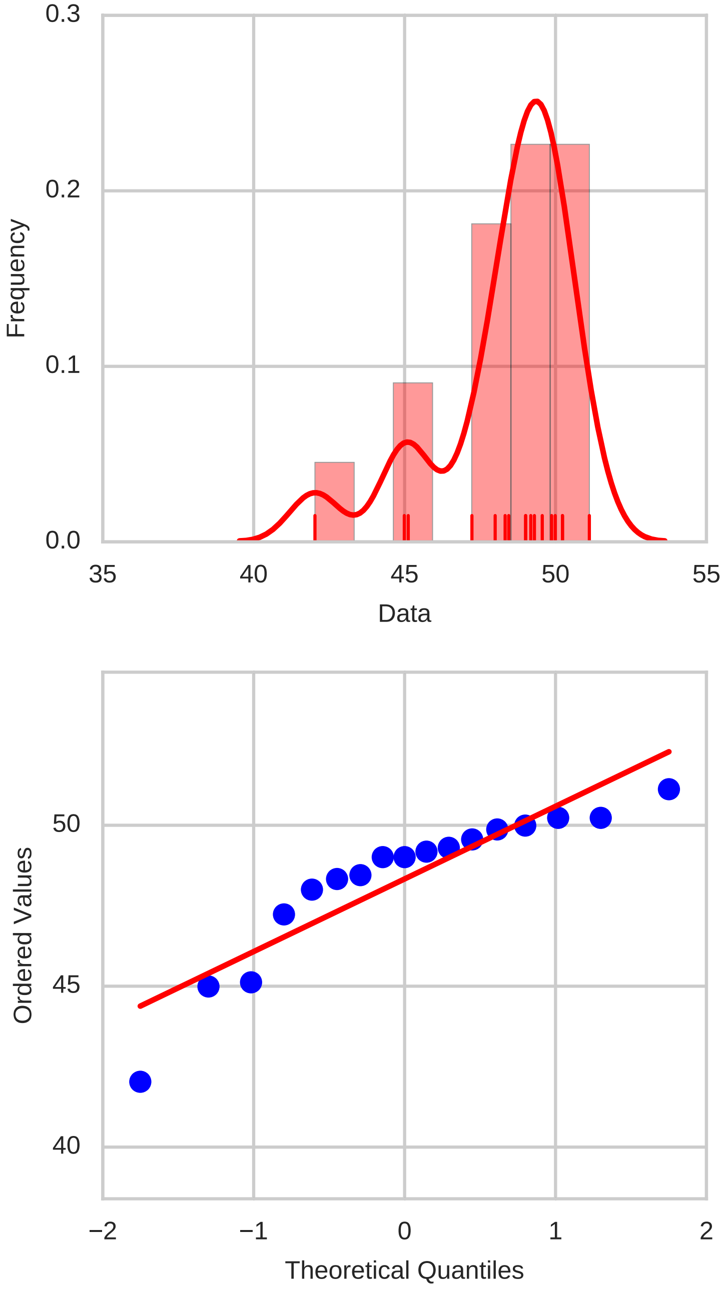

Figure 12: Catalase levels for the group treated with both *Withania somnifera* and *Centella asiatica* (CAT_WC): The data set was found to be non-normal. The Shapiro Wilk Test-Statistic was 0.846 (Critical Value: 0.892, failed). The Anderson-Darling Test Static was 1.026 (Critical Value: 0.685, failed). The Kolmogorov-Smirnov Test Statistic was 0.206 (Critical Value: 0.318, passed).

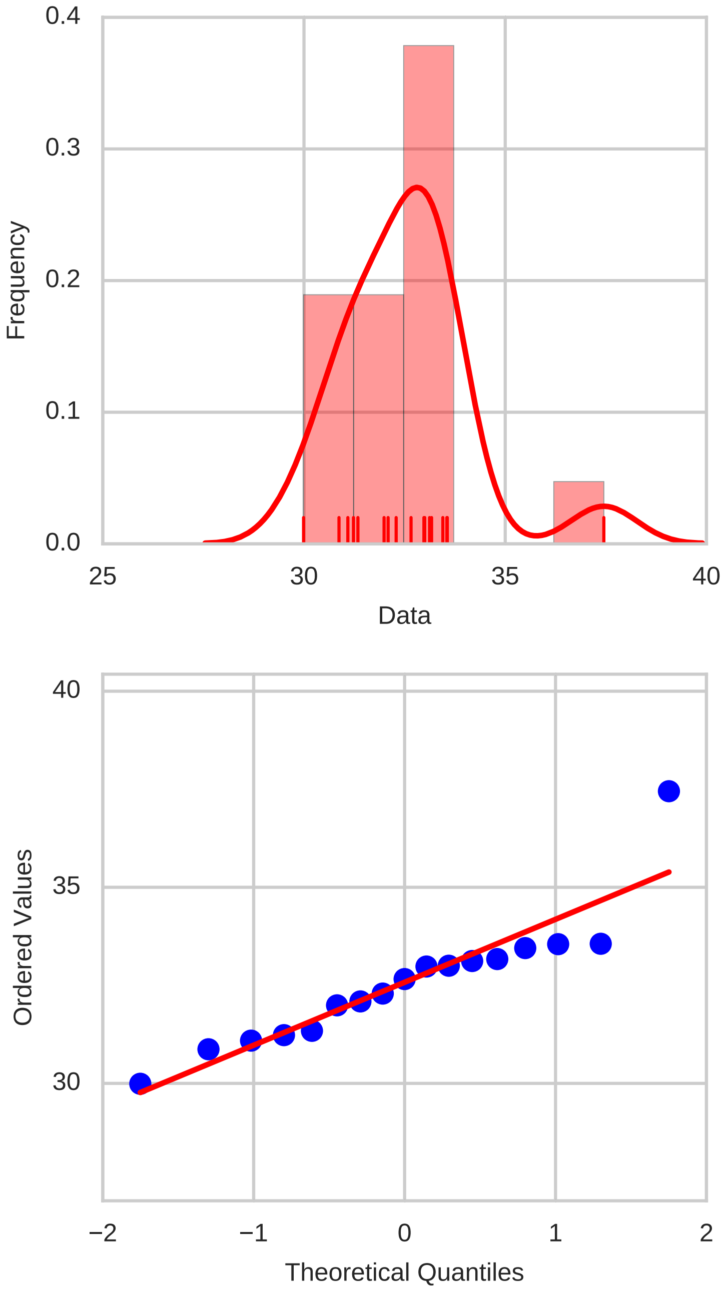

Figure 13: Catalase levels for the MPTP disease induced and no treatment group (CAT_M): The data set was found to be non-normal. The Shapiro Wilk Test-Statistic was 0.874 (Critical Value: 0.892, failed). The Anderson-Darling Test Static was 0.661 (Critical Value: 0.685, passed). The Kolmogorov-Smirnov Test Statistic was 0.210 (Critical Value: 0.318, passed).

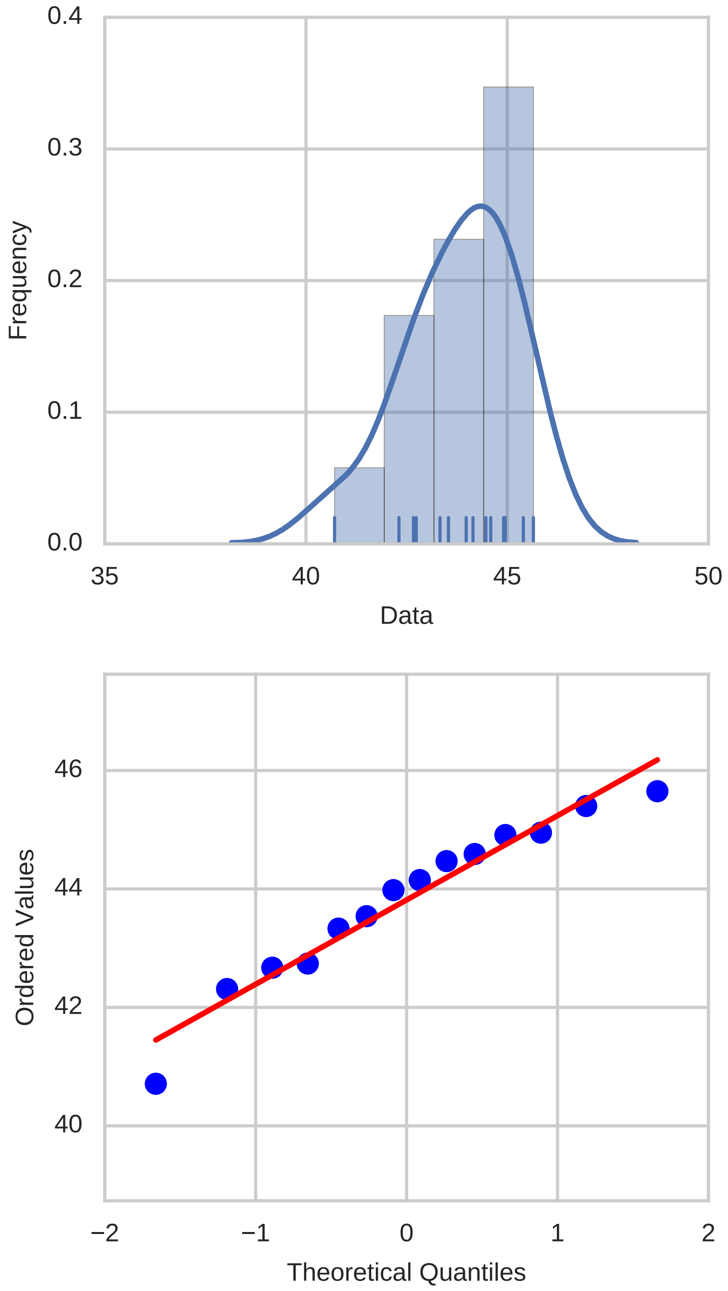

Figure 14: Catalase levels for the MPTP disease induced and treated with *Centella asiatica* group (CAT_MC): The data set was found to be normal. The Shapiro Wilk Test-Statistic was 0.951 (Critical Value: 0.874, passed). The Anderson-Darling Test Static was 0.256 (Critical Value: 0.680, passed). The Kolmogorov-Smirnov Test Statistic was 0.122 (Critical Value: 0.349, passed).

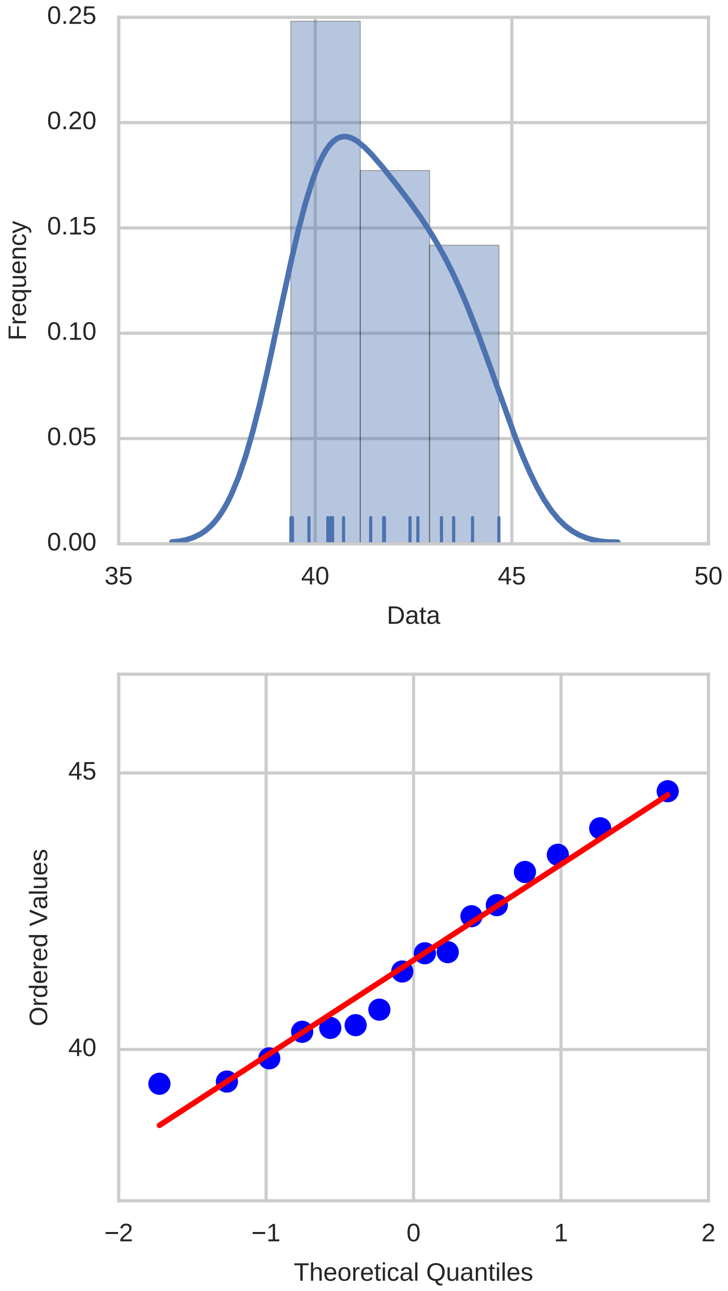

Figure 15: Catalase levels for the MPTP disease induced and treated with *Withania somnifera* group (CAT_MW): The data set was found to be normal. The Shapiro Wilk Test-Statistic was 0.950 (Critical Value: 0.887, passed). The Anderson-Darling Test Static was 0.283 (Critical Value: 0.683, passed). The Kolmogorov-Smirnov Test Statistic was 0.149 (Critical Value: 0.327, passed).

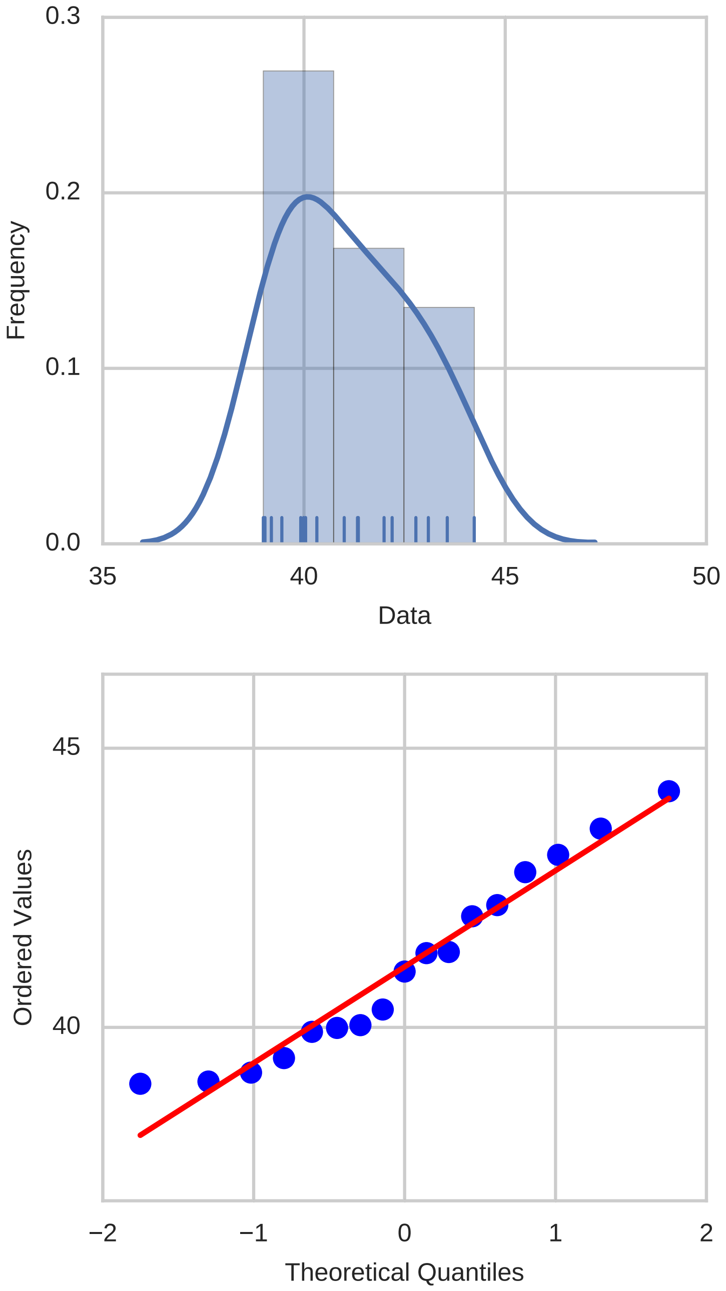

Figure 16: Catalase levels for the MPTP disease induced and treated with both *Withania somnifera* and *Centella asiatica* group (CAT_MWC): The data set was found to be normal. The Shapiro Wilk Test-Statistic was 0.938 (Critical Value: 0.892, passed). The Anderson-Darling Test Static was 0.359 (Critical Value: 0.685, passed). The Kolmogorov-Smirnov Test Statistic was 0.153 (Critical Value: 0.318, passed).

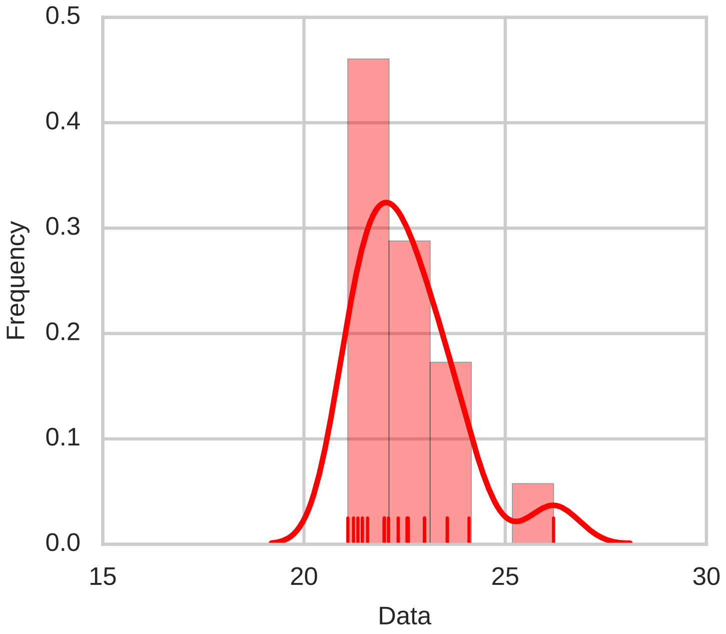

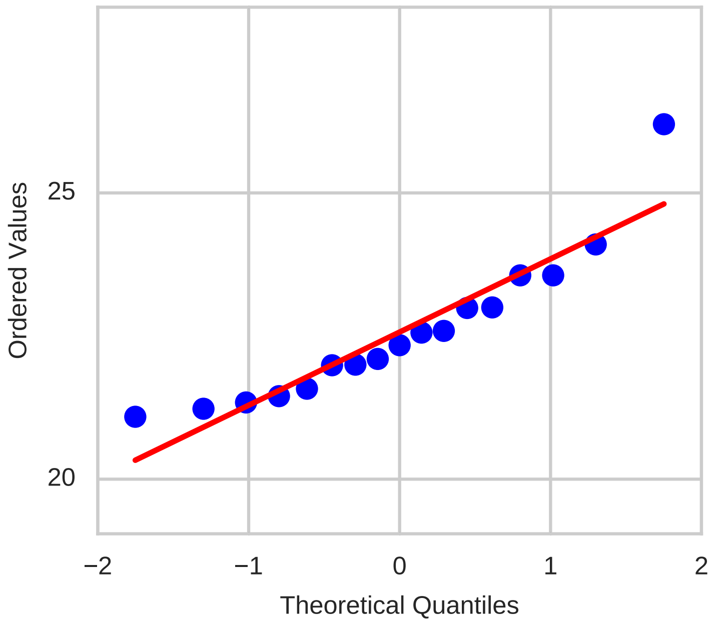

Figure 17: Glutathione peroxidase levels for the untreated group (GPx_U): The data set was found to be non-normal. The Shapiro Wilk Test-Statistic was 0.887 (Critical Value: 0.892, failed). The Anderson-Darling Test Static was 0.542 (Critical Value: 0.685, passed). The Kolmogorov-Smirnov Test Statistic was 0.140 (Critical Value: 0.318, passed).

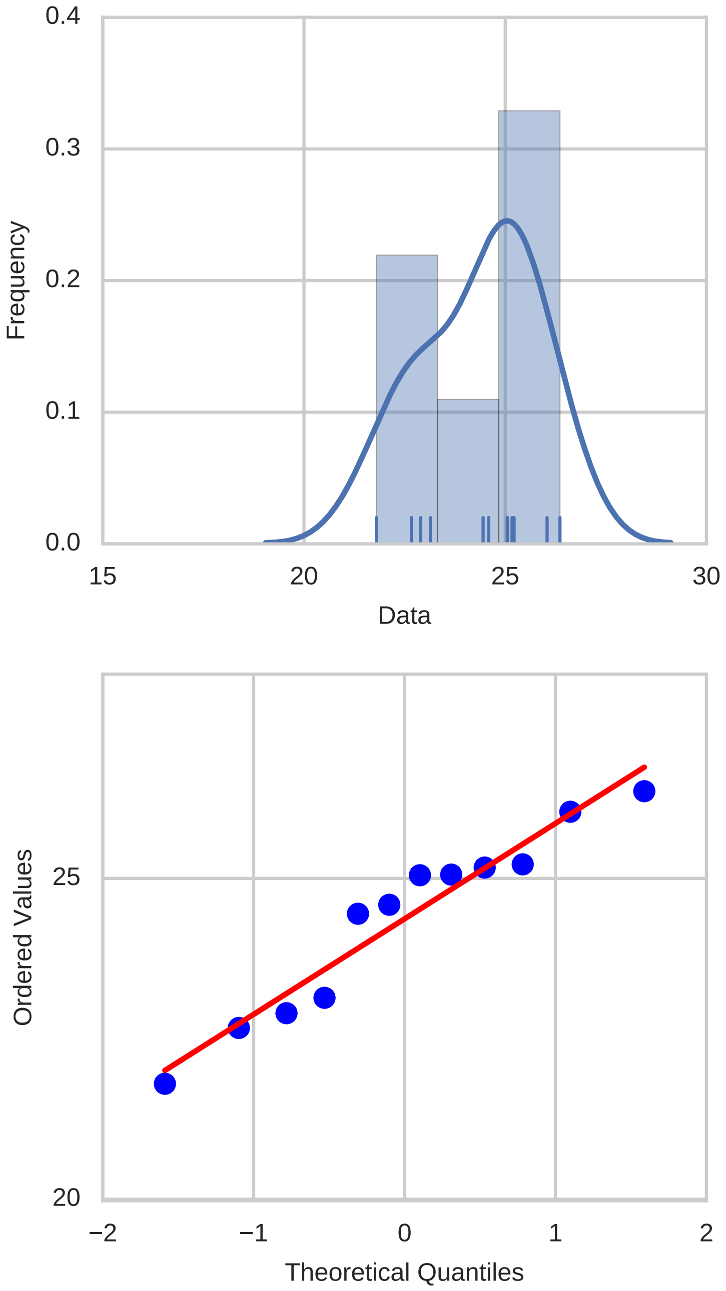

Figure 18: Glutathione peroxidase levels for the group treated with *Centella asiatica* (GPx_C): The data set was found to be normal. The Shapiro Wilk Test-Statistic was 0.931 (Critical Value: 0.859, passed). The Anderson-Darling Test Static was 0.430 (Critical Value: 0.679, passed). The Kolmogorov-Smirnov Test Statistic was 0.191 (Critical Value: 0.375, passed).

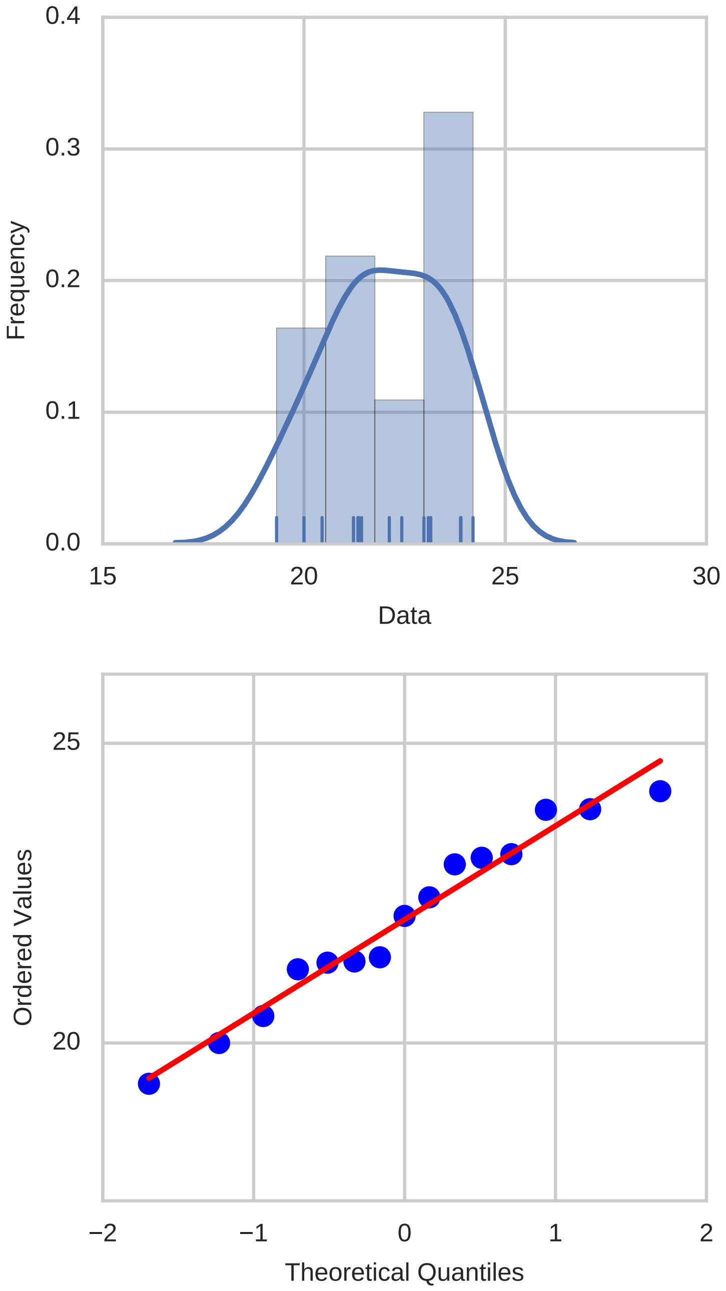

Figure 19: Glutathione peroxidase levels for the group treated with *Withania somnifera* (GPx_W): The data set was found to be normal. The Shapiro Wilk Test-Statistic was 0.956 (Critical Value: 0.881, passed). The Anderson-Darling Test Static was 0.262 (Critical Value: 0.681, passed). The Kolmogorov-Smirnov Test Statistic was 0.139 (Critical Value: 0.338, passed).

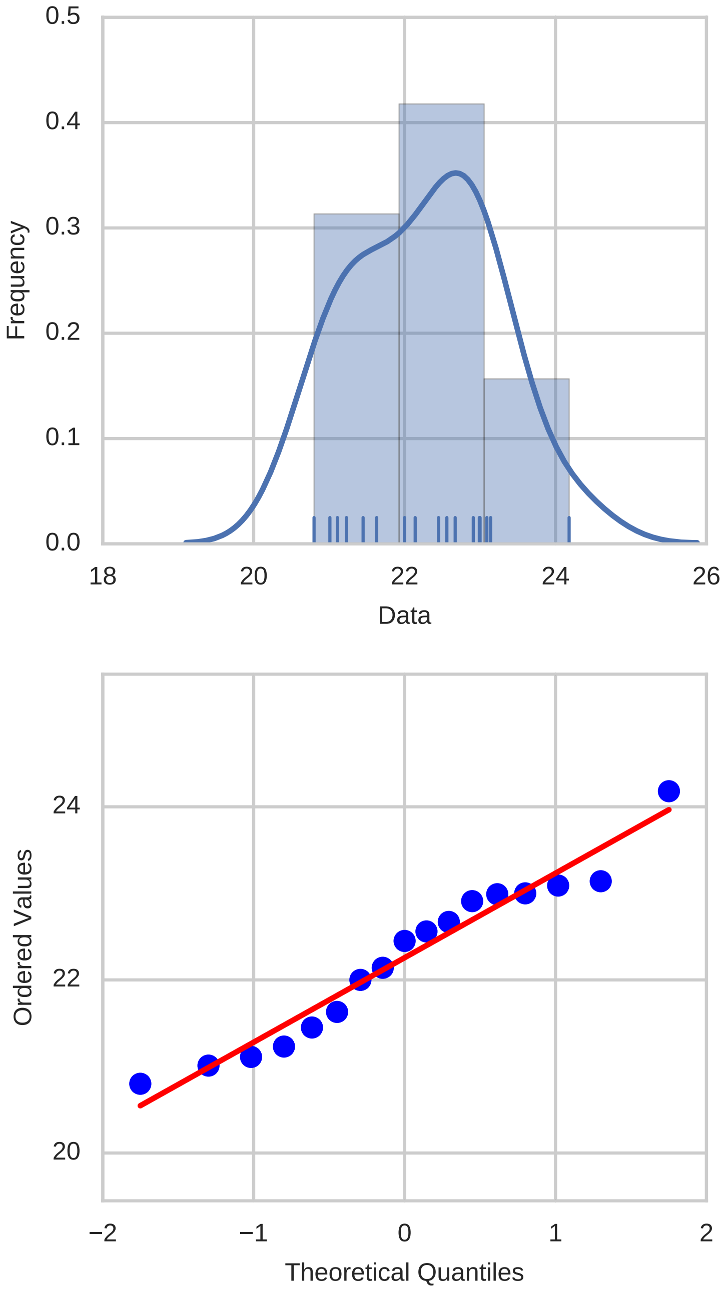

Figure 20: Glutathione peroxidase levels for the group treated with both *Withania somnifera* and *Centella asiatica* (GPx_WC): The data set was found to be normal. The Shapiro Wilk Test-Statistic was 0.952 (Critical Value: 0.892, passed). The Anderson-Darling Test Static was 0.351 (Critical Value: 0.685, passed). The Kolmogorov-Smirnov Test Statistic was 0.116 (Critical Value: 0.318, passed).

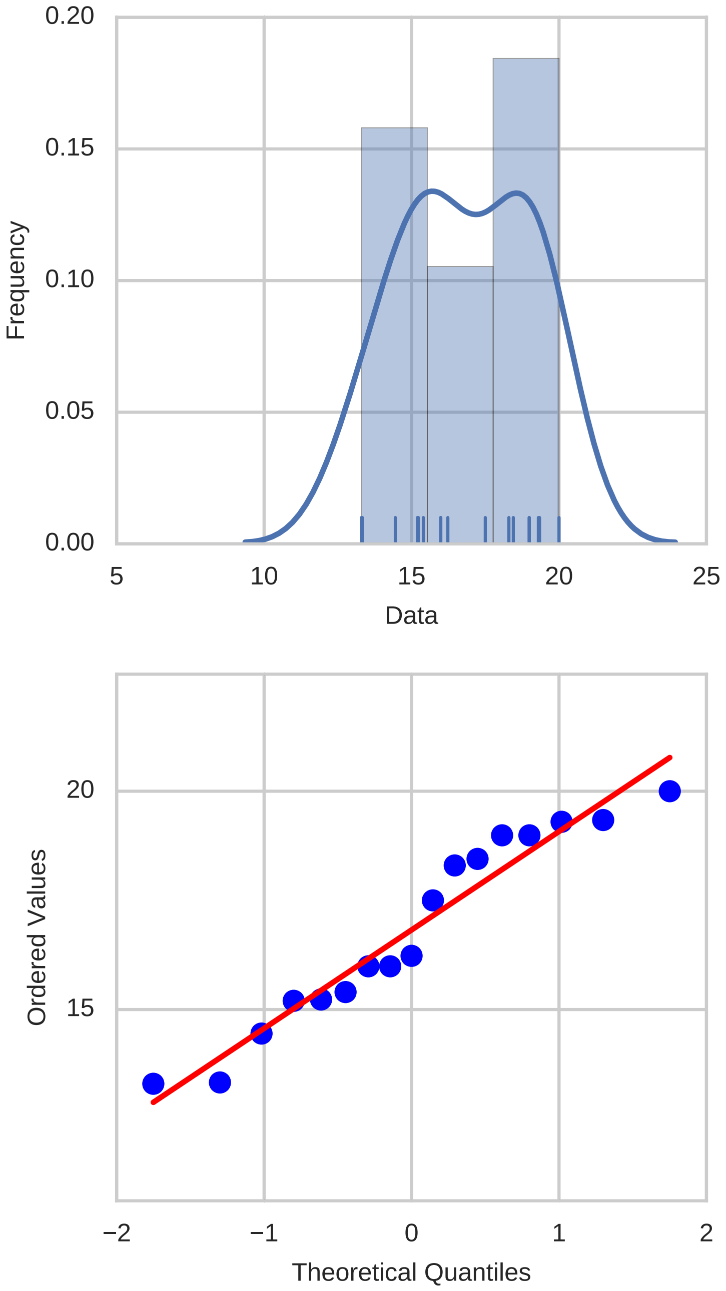

Figure 21: Glutathione peroxidase levels for the MPTP disease induced and no treatment group (GPx_M): The data set was found to be normal. The Shapiro Wilk Test-Statistic was 0.926 (Critical Value: 0.892, passed). The Anderson-Darling Test Static was 0.483 (Critical Value: 0.685, passed). The Kolmogorov-Smirnov Test Statistic was 0.169 (Critical Value: 0.318, passed).

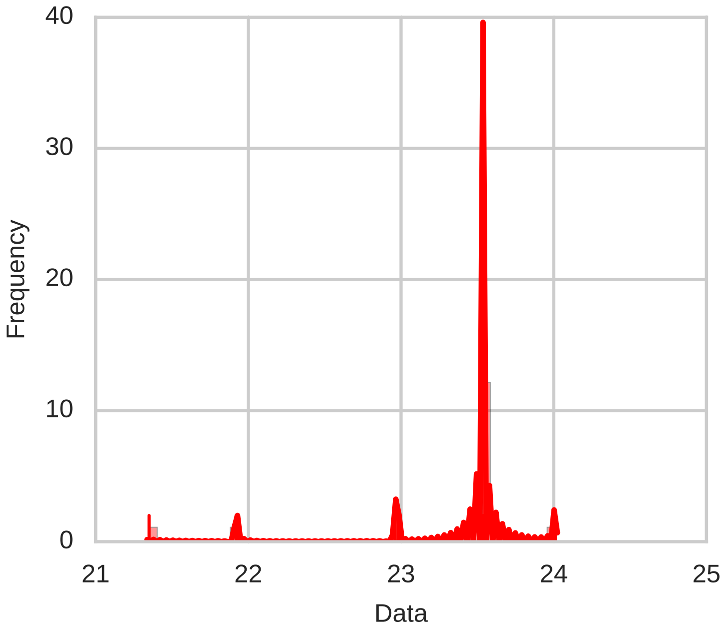

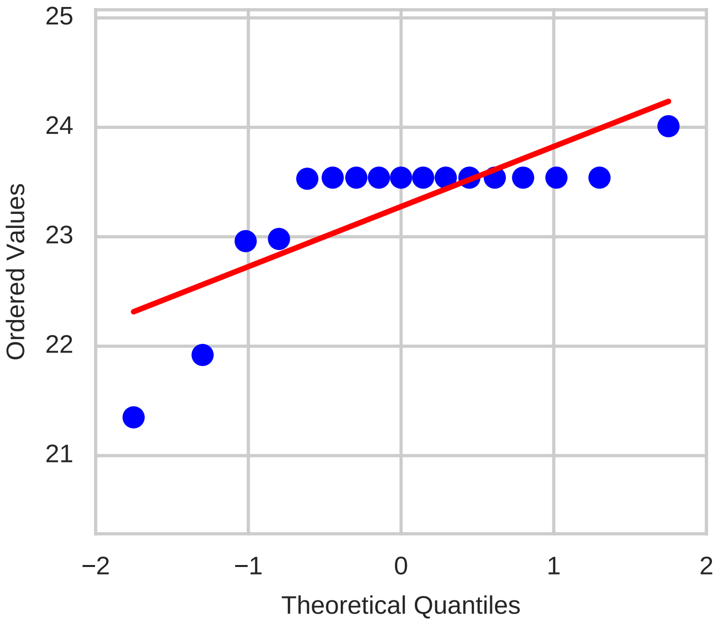

Figure 22: Glutathione peroxidase levels for the MPTP disease induced and treated with *Centella asiatica* group (GPx_MC): The data set was found to be non-normal. The Shapiro Wilk Test-Statistic was 0.628 (Critical Value: 0.892, failed). The Anderson-Darling Test Static was 3.037 (Critical Value: 0.685, failed). The Kolmogorov-Smirnov Test Statistic was 0.418 (Critical Value: 0.318, failed).

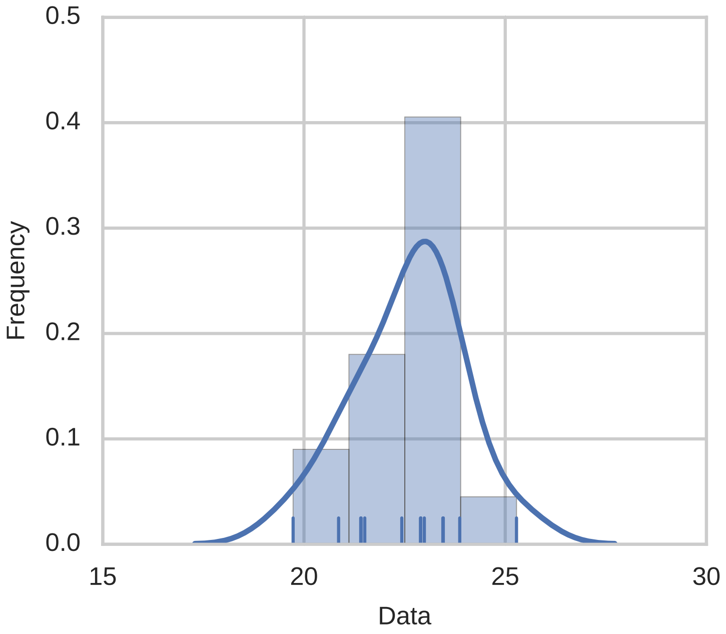

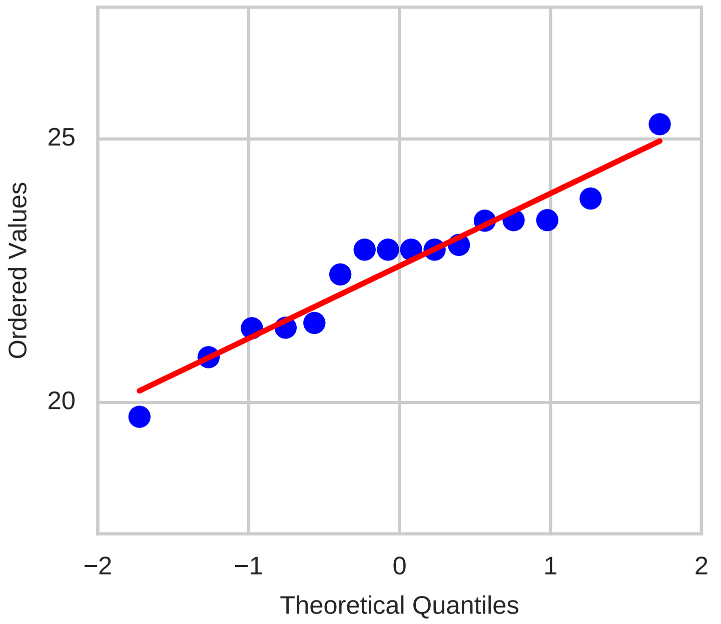

Figure 23: Glutathione peroxidase levels for the MPTP disease induced and treated with *Withania somnifera* group (GPx_MW): The data set was found to be normal. The Shapiro Wilk Test-Statistic was 0.950 (Critical Value: 0.887, passed). The Anderson-Darling Test Static was 0.495 (Critical Value: 0.683, passed). The Kolmogorov-Smirnov Test Statistic was 0.219 (Critical Value: 0.327, passed).

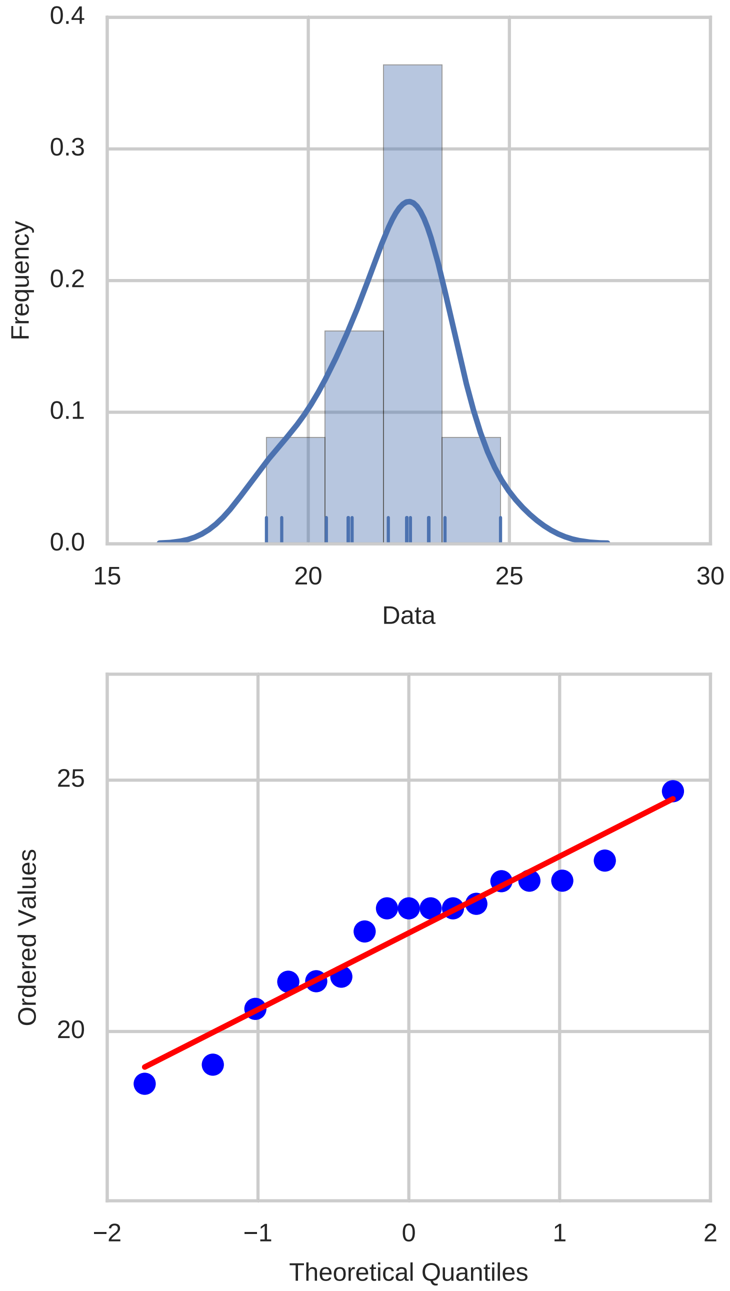

Figure 24: Glutathione peroxidase levels for the MPTP disease induced and treated with both *Withania somnifera* and *Centella asiatica* group (GPx_MWC): The data set was found to be normal. The Shapiro Wilk Test-Statistic was 0.941 (Critical Value: 0.892, passed). The Anderson-Darling Test Static was 0.543 (Critical Value: 0.685, passed). The Kolmogorov-Smirnov Test Statistic was 0.221 (Critical Value: 0.318, passed).

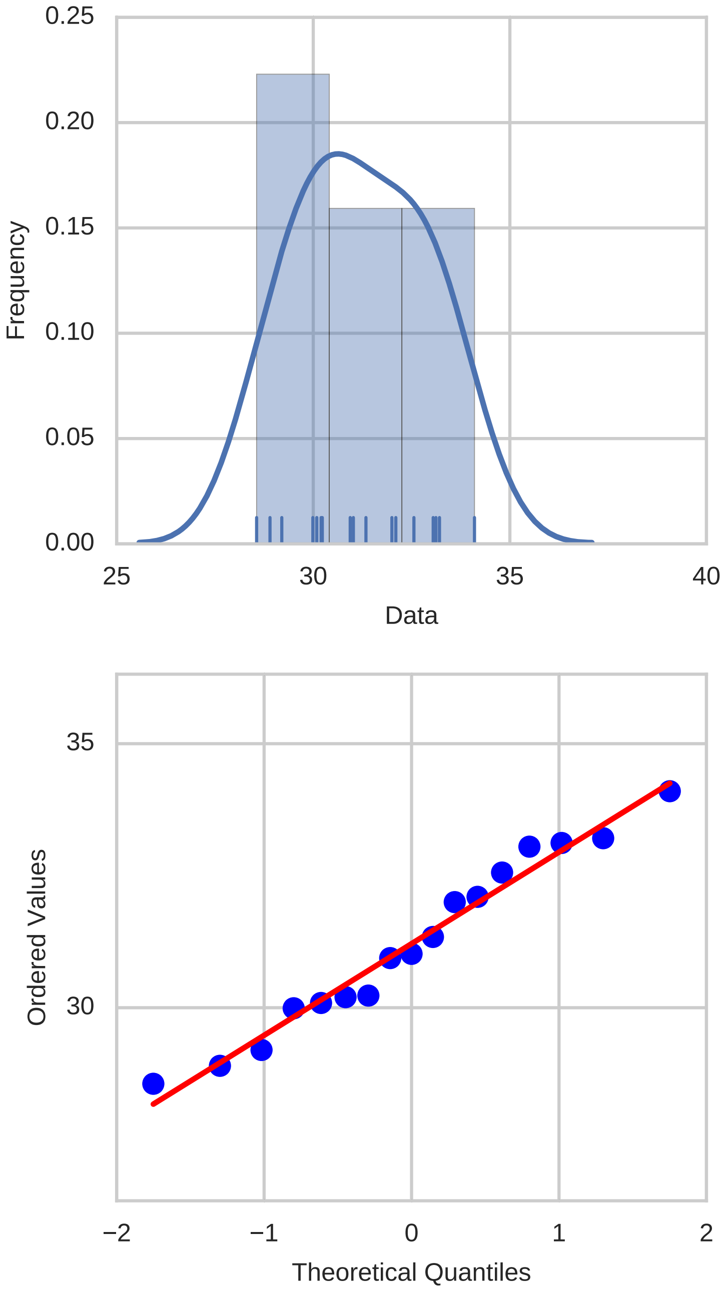

Figure 25: Reduced glutathione levels for the untreated group (GSH_U): The data set was found to be normal. The Shapiro Wilk Test-Statistic was 0.962 (Critical Value: 0.892, passed). The Anderson-Darling Test Static was 0.255 (Critical Value: 0.685, passed). The Kolmogorov-Smirnov Test Statistic was 0.141 (Critical Value: 0.318, passed).

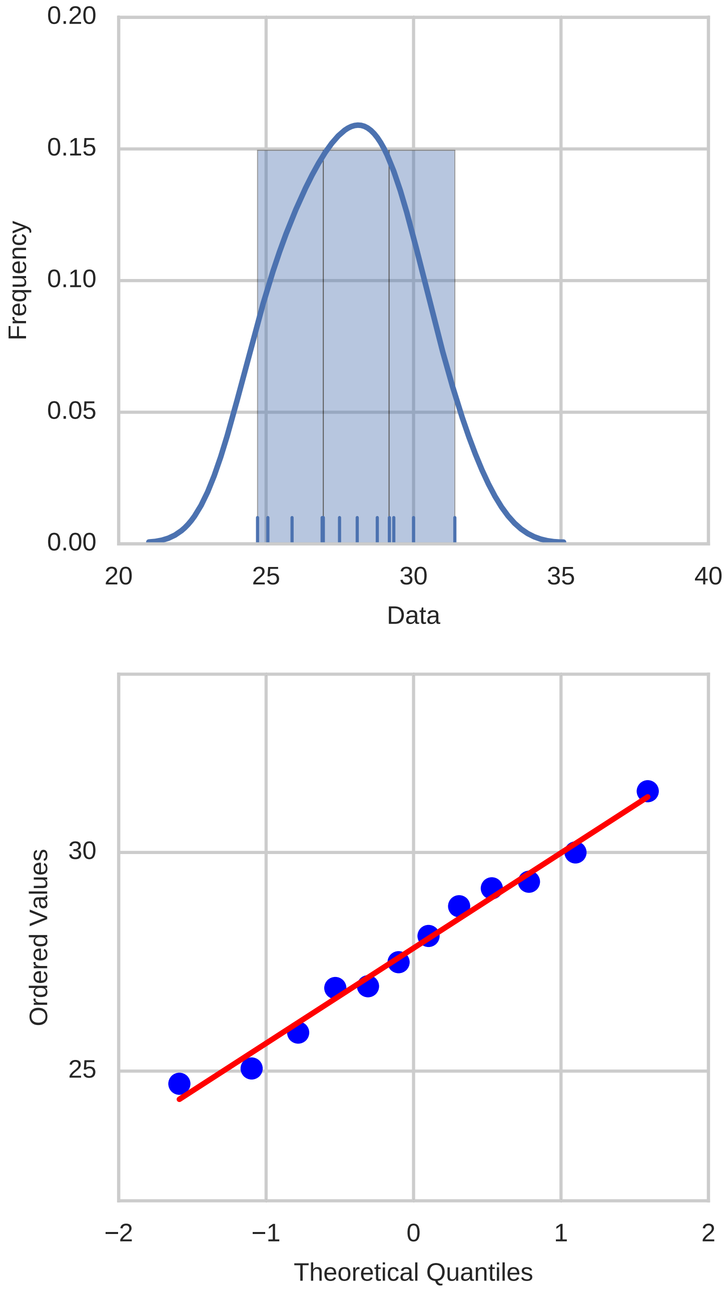

Figure 26: Reduced glutathione levels for the group treated with *Centella asiatica* (GSH_C): The data set was found to be normal. The Shapiro Wilk Test-Statistic was 0.977 (Critical Value: 0.859, passed). The Anderson-Darling Test Static was 0.143 (Critical Value: 0.679, passed). The Kolmogorov-Smirnov Test Statistic was 0.105 (Critical Value: 0.375, passed).

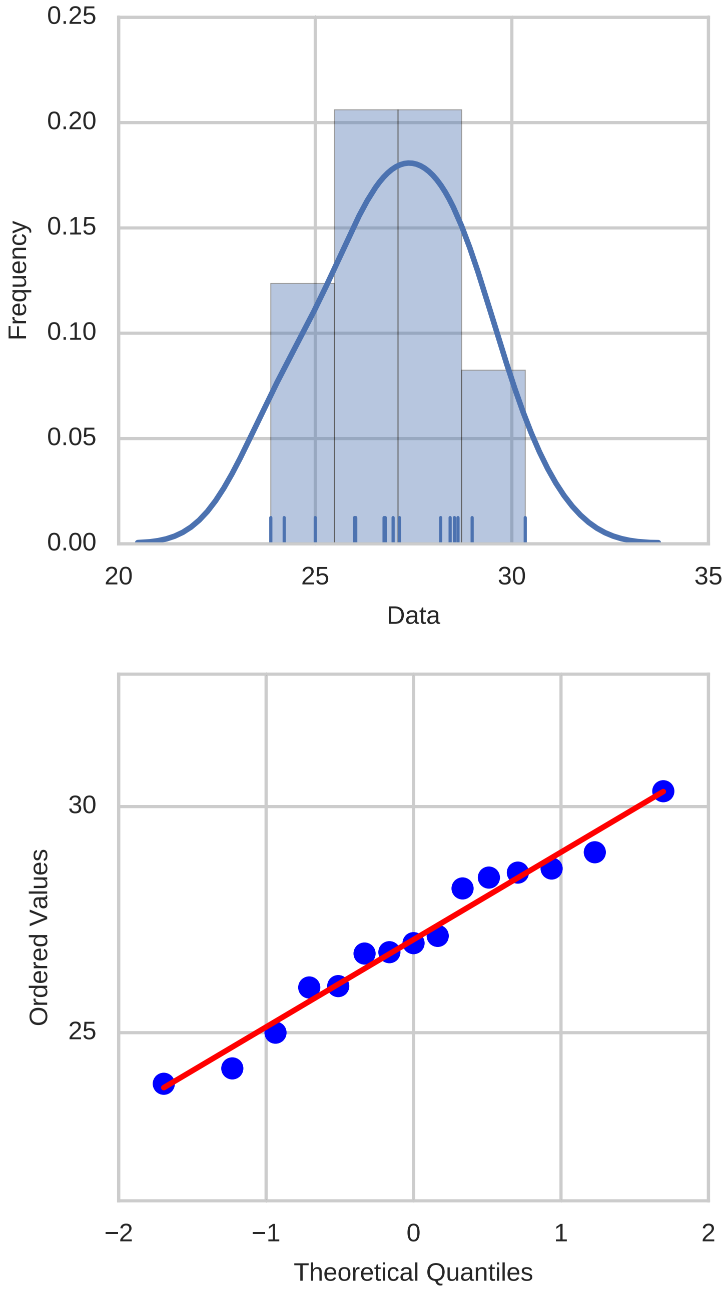

Figure 27: Reduced glutathione levels for the group treated with *Withania somnifera* (GSH_W): The data set was found to be normal. The Shapiro Wilk Test-Statistic was 0.969 (Critical Value: 0.881, passed). The Anderson-Darling Test Static was 0.235 (Critical Value: 0.681, passed). The Kolmogorov-Smirnov Test Statistic was 0.138 (Critical Value: 0.338, passed).

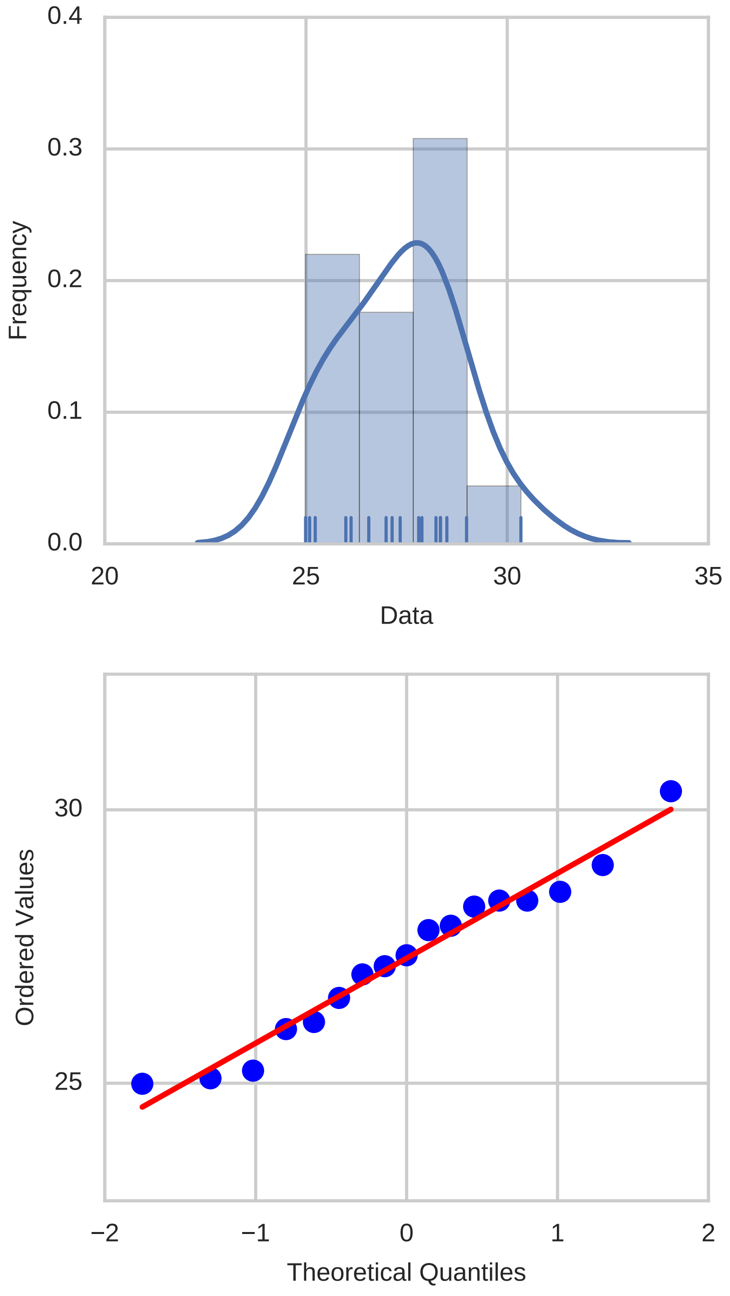

Figure 28: Reduced glutathione levels for the group treated with both *Withania somnifera* and *Centella asiatica* (GSH_WC): The data set was found to be normal. The Shapiro Wilk Test-Statistic was 0.964 (Critical Value: 0.892, passed). The Anderson-Darling Test Static was 0.245 (Critical Value: 0.685, passed). The Kolmogorov-Smirnov Test Statistic was 0.110 (Critical Value: 0.318, passed).

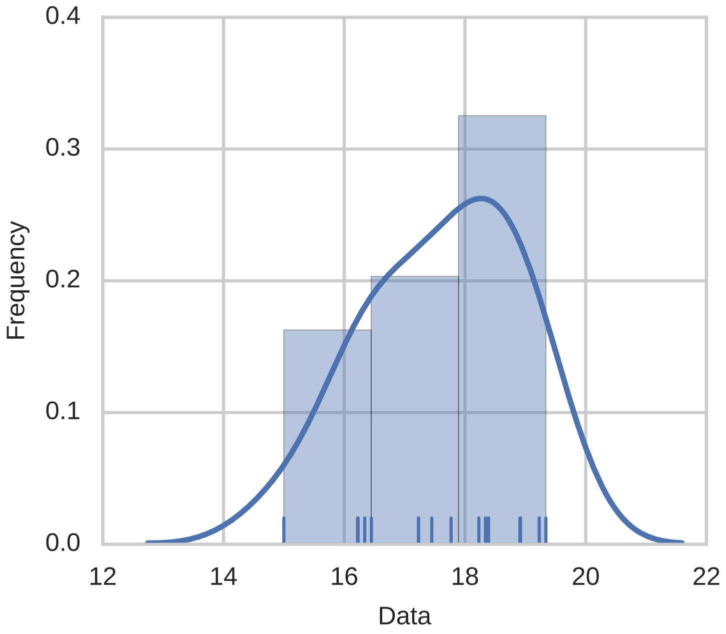

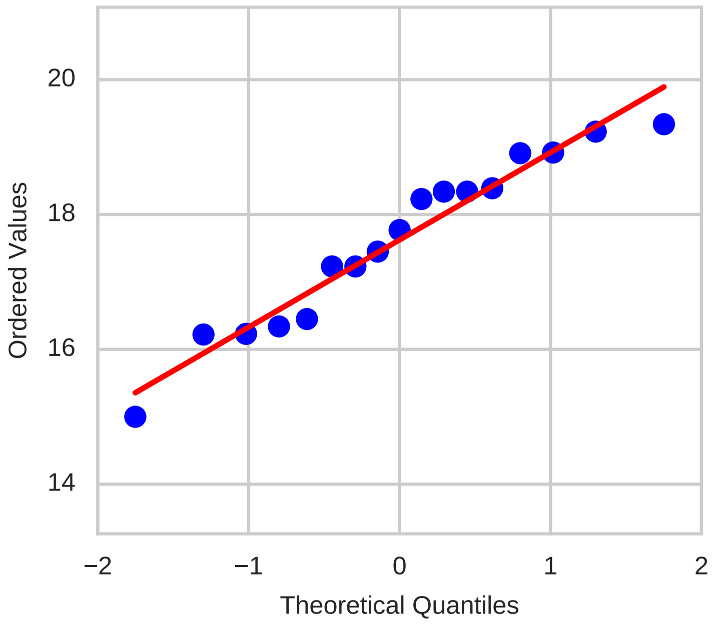

Figure 29: Reduced glutathione levels for the MPTP disease induced and no treatment group (GSH_M): The data set was found to be normal. The Shapiro Wilk Test-Statistic was 0.945 (Critical Value: 0.892, passed). The Anderson-Darling Test Static was 0.367 (Critical Value: 0.685, passed). The Kolmogorov-Smirnov Test Statistic was 0.162 (Critical Value: 0.318, passed).

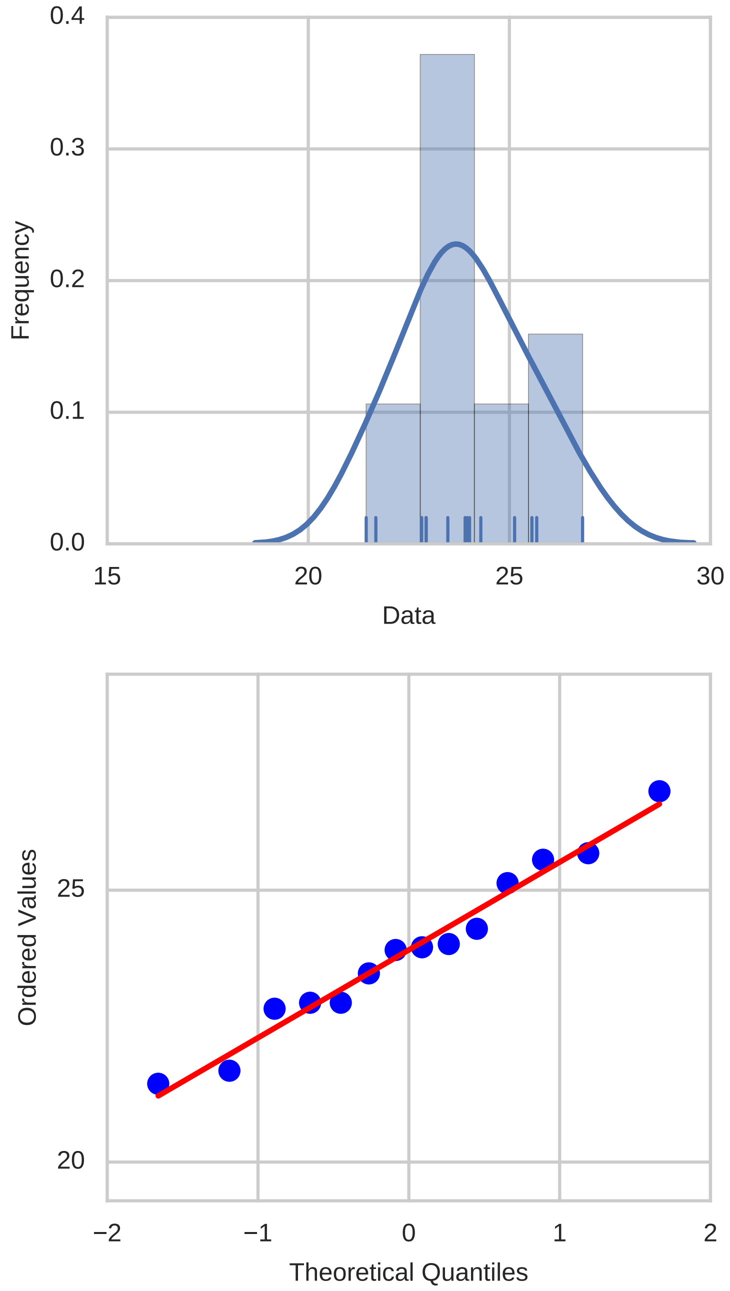

Figure 30: Reduced glutathione levels for the MPTP disease induced and treated with *Centella asiatica* group (GSH_MC): The data set was found to be normal. The Shapiro Wilk Test-Statistic was 0.972 (Critical Value: 0.874, passed). The Anderson-Darling Test Static was 0.205 (Critical Value: 0.680, passed). The Kolmogorov-Smirnov Test Statistic was 0.113 (Critical Value: 0.349, passed).

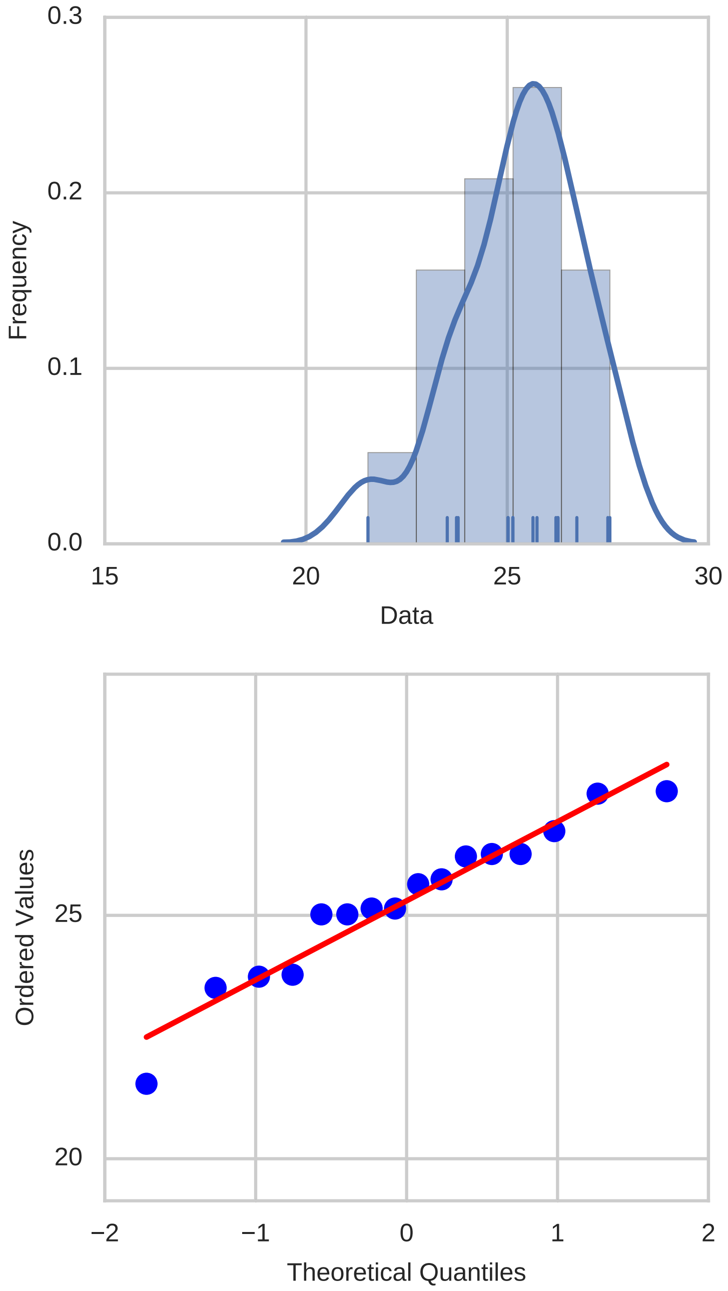

Figure 31: Reduced glutathione levels for the MPTP disease induced and treated with *Withania somnifera* group (GSH_MW): The data set was found to be normal. The Shapiro Wilk Test-Statistic was 0.945 (Critical Value: 0.887, passed). The Anderson-Darling Test Static was 0.345 (Critical Value: 0.683, passed). The Kolmogorov-Smirnov Test Statistic was 0.177 (Critical Value: 0.327, passed).

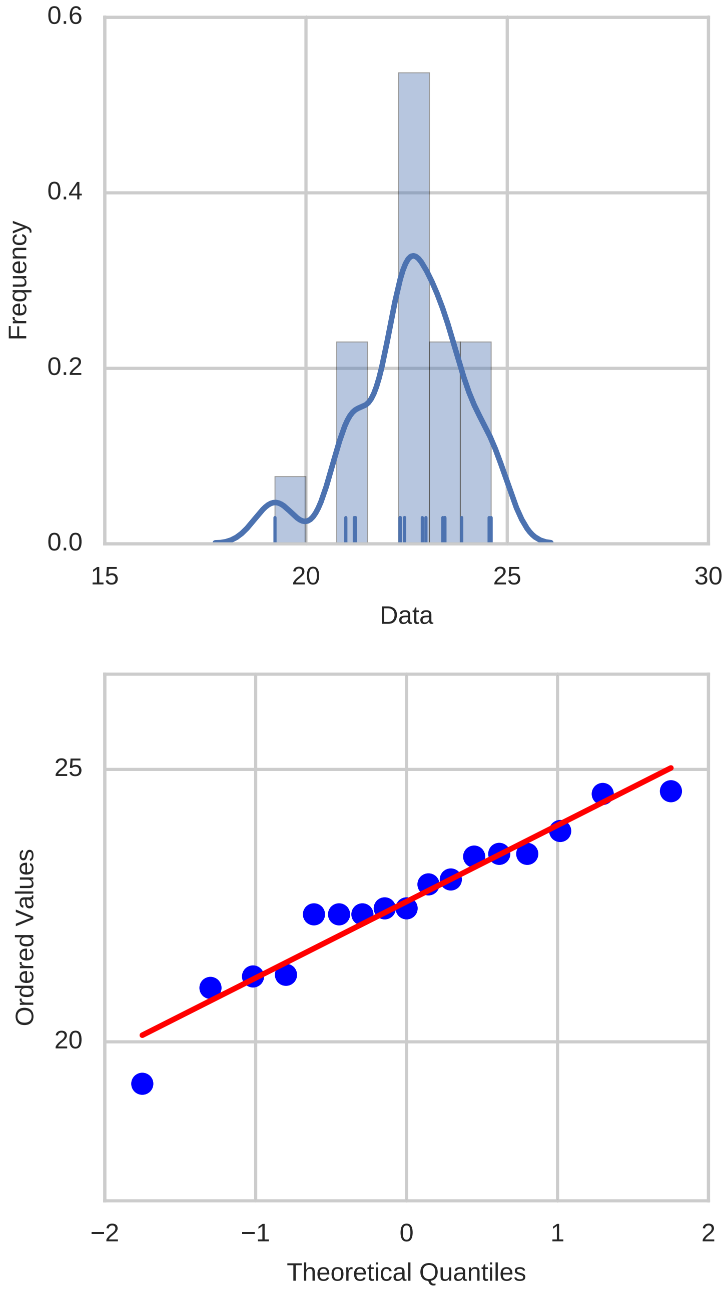

Figure 32: Reduced glutathione levels for the MPTP disease induced and treated with both *Withania somnifera* and *Centella asiatica* group (GSH_MWC): The data set was found to be normal. The Shapiro Wilk Test-Statistic was 0.945 (Critical Value: 0.892, passed). The Anderson-Darling Test Static was 0.373 (Critical Value: 0.685, passed). The Kolmogorov-Smirnov Test Statistic was 0.194 (Critical Value: 0.318, passed).

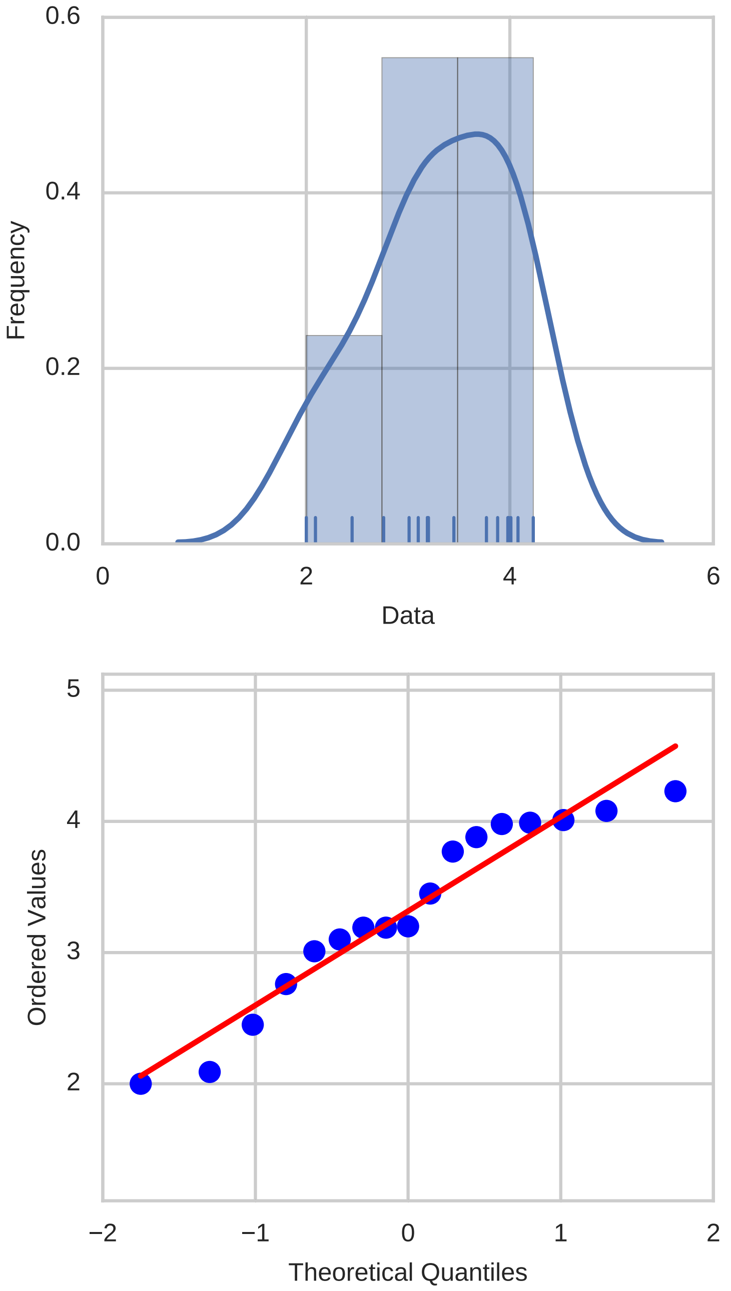

Figure 33: Lipid peroxidation levels for the untreated group (LPO_U): The data set was found to be normal. The Shapiro Wilk Test-Statistic was 0.923 (Critical Value: 0.892, passed). The Anderson-Darling Test Static was 0.474 (Critical Value: 0.685, passed). The Kolmogorov-Smirnov Test Statistic was 0.160 (Critical Value: 0.318, passed).

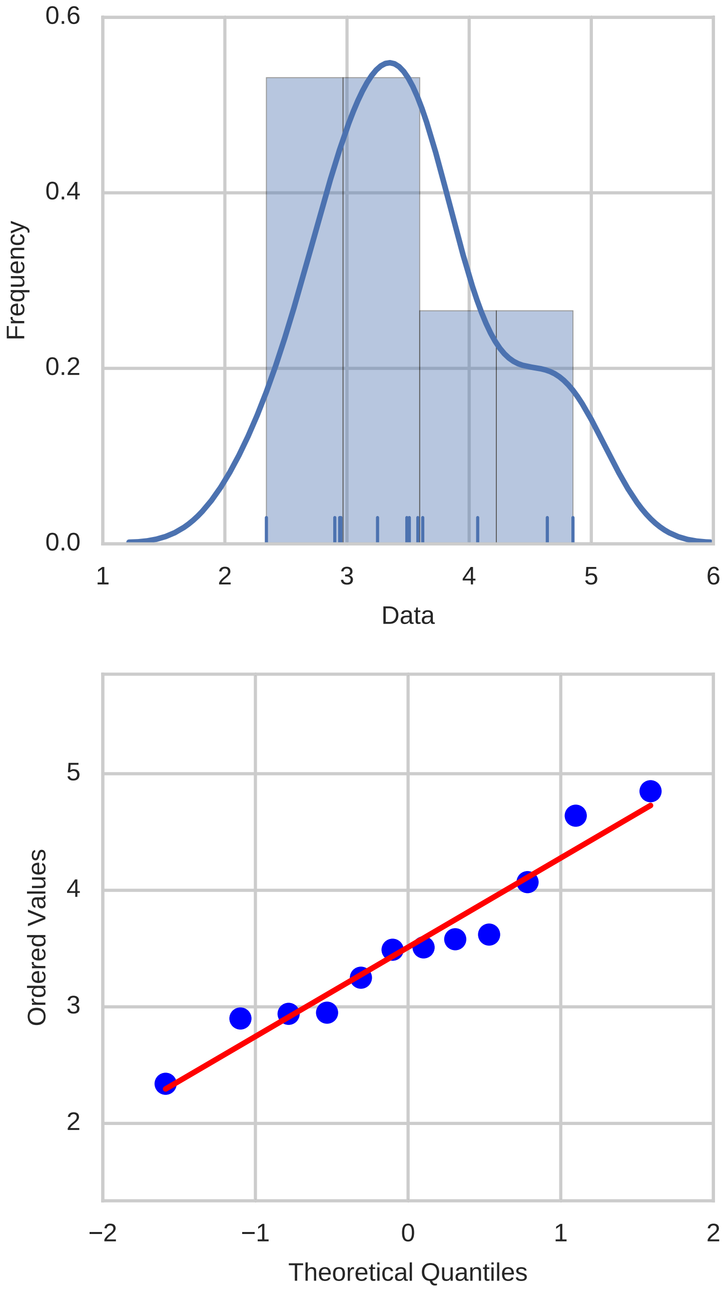

Figure 34: Lipid peroxidation levels for the group treated with *Centella asiatica* (LPO_C): The data set was found to be normal. The Shapiro Wilk Test-Statistic was 0.948 (Critical Value: 0.859, passed). The Anderson-Darling Test Static was 0.334 (Critical Value: 0.679, passed). The Kolmogorov-Smirnov Test Statistic was 0.188 (Critical Value: 0.375, passed).

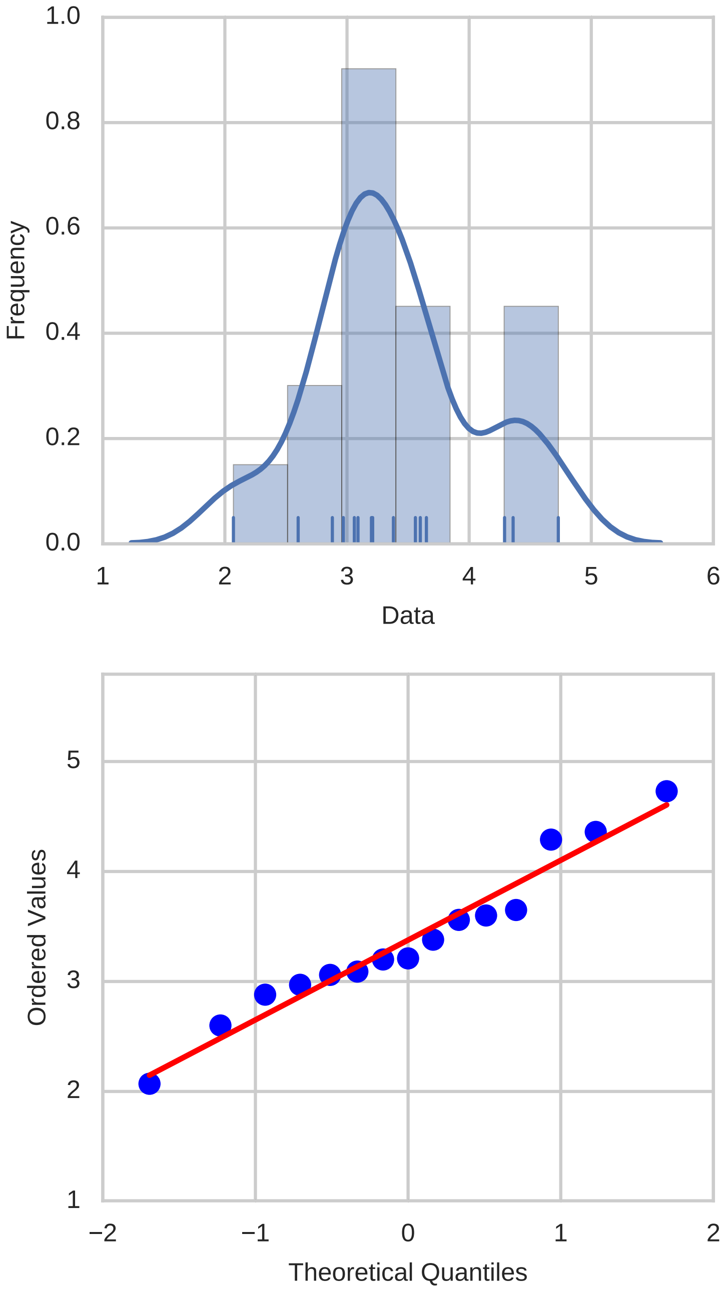

Figure 35: Lipid peroxidation levels for the group treated with *Withania somnifera* (LPO_W): The data set was found to be normal. The Shapiro Wilk Test-Statistic was 0.965 (Critical Value: 0.881, passed). The Anderson-Darling Test Static was 0.318 (Critical Value: 0.681, passed). The Kolmogorov-Smirnov Test Statistic was 0.142 (Critical Value: 0.338, passed).

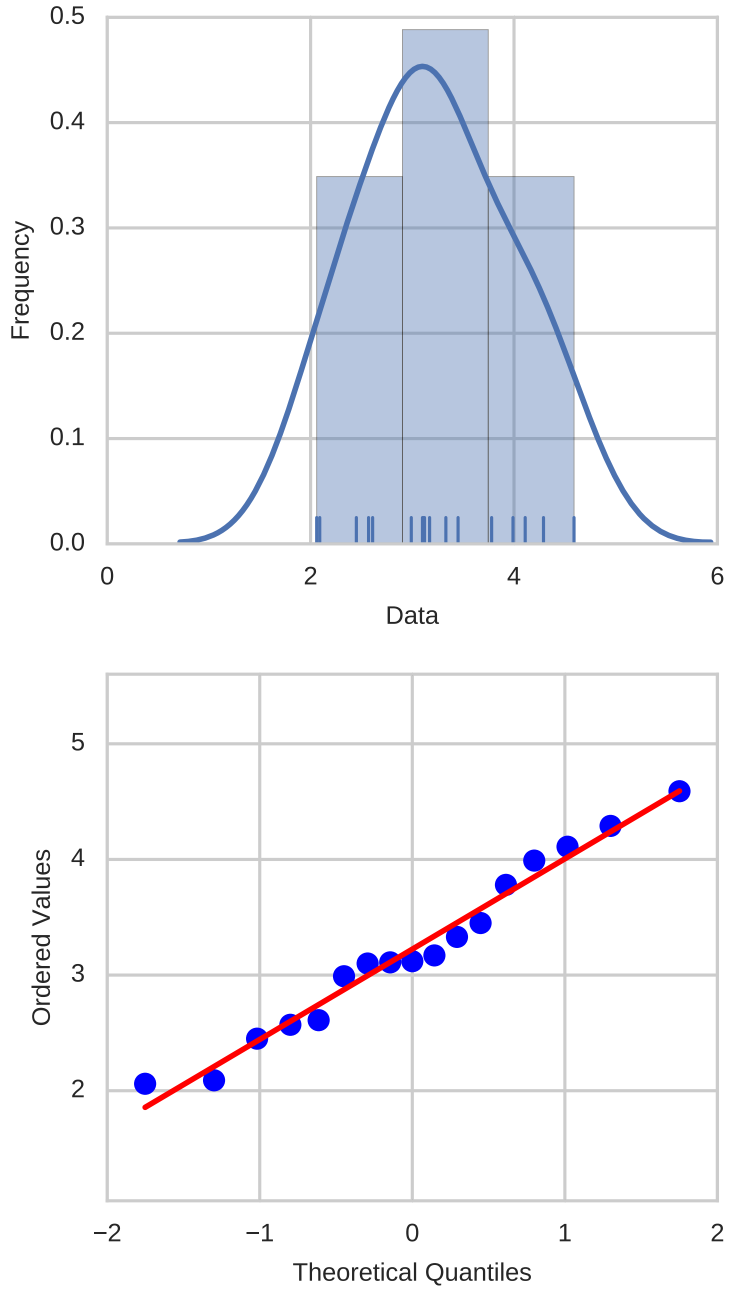

Figure 36: Lipid peroxidation levels for the group treated with both *Withania somnifera* and *Centella asiatica* (LPO_WC): The data set was found to be normal. The Shapiro Wilk Test-Statistic was 0.965 (Critical Value: 0.892, passed). The Anderson-Darling Test Static was 0.235 (Critical Value: 0.685, passed). The Kolmogorov-Smirnov Test Statistic was 0.118 (Critical Value: 0.318, passed).

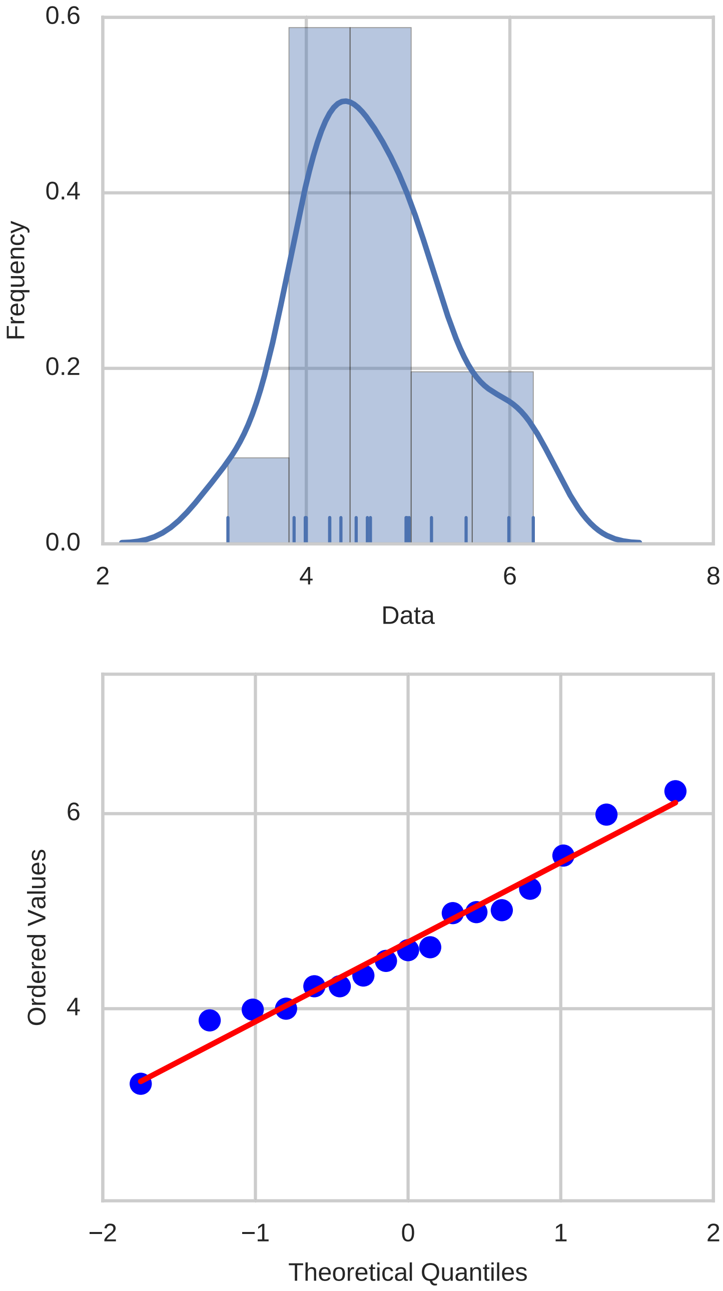

Figure 37: Lipid peroxidation levels for the MPTP disease induced and no treatment group (LPO_M): The data set was found to be normal. The Shapiro Wilk Test-Statistic was 0.973 (Critical Value: 0.892, passed). The Anderson-Darling Test Static was 0.238 (Critical Value: 0.685, passed). The Kolmogorov-Smirnov Test Statistic was 0.116 (Critical Value: 0.318, passed).

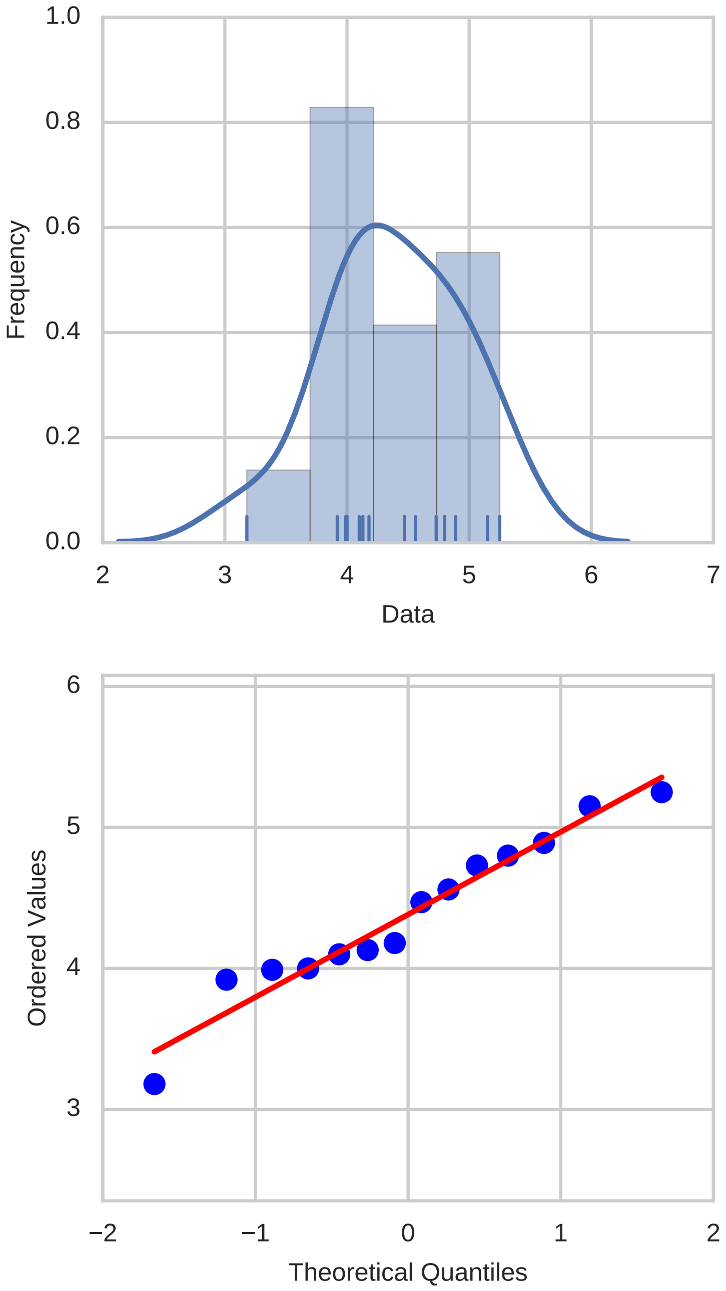

Figure 38: Lipid peroxidation levels for the MPTP disease induced and treated with *Centella asiatica* group (LPO_MC): The data set was found to be normal. The Shapiro Wilk Test-Statistic was 0.958 (Critical Value: 0.874, passed). The Anderson-Darling Test Static was 0.275 (Critical Value: 0.680, passed). The Kolmogorov-Smirnov Test Statistic was 0.146 (Critical Value: 0.349, passed).

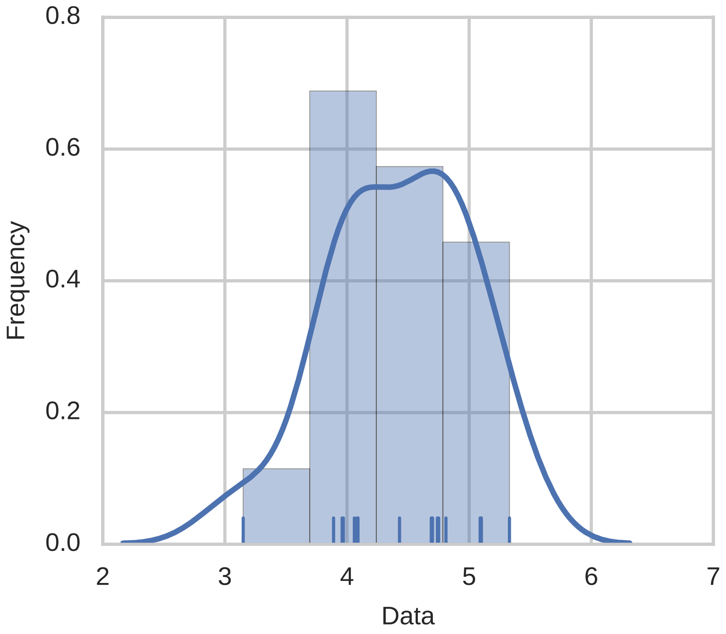

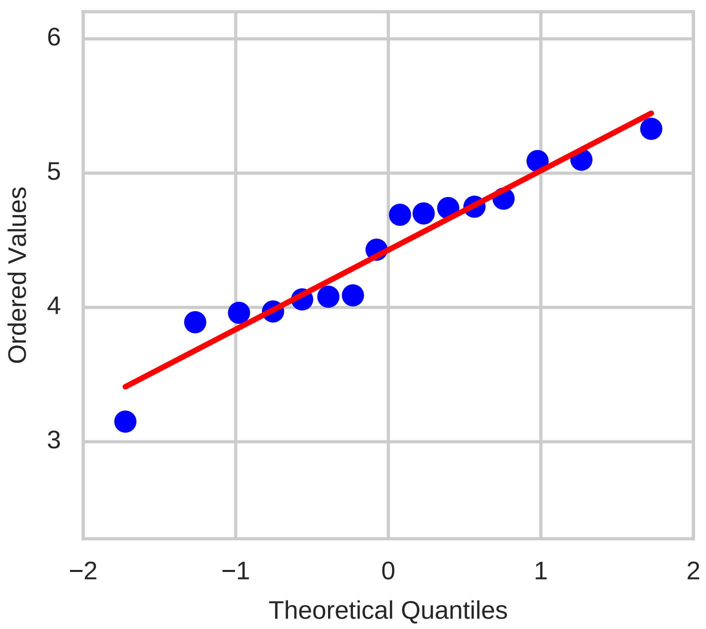

Figure 39: Lipid peroxidation levels for the MPTP disease induced and treated with *Withania somnifera* group (LPO_MW): The data set was found to be normal. The Shapiro Wilk Test-Statistic was 0.945 (Critical Value: 0.887, passed). The Anderson-Darling Test Static was 0.425 (Critical Value: 0.683, passed). The Kolmogorov-Smirnov Test Statistic was 0.182 (Critical Value: 0.327, passed).

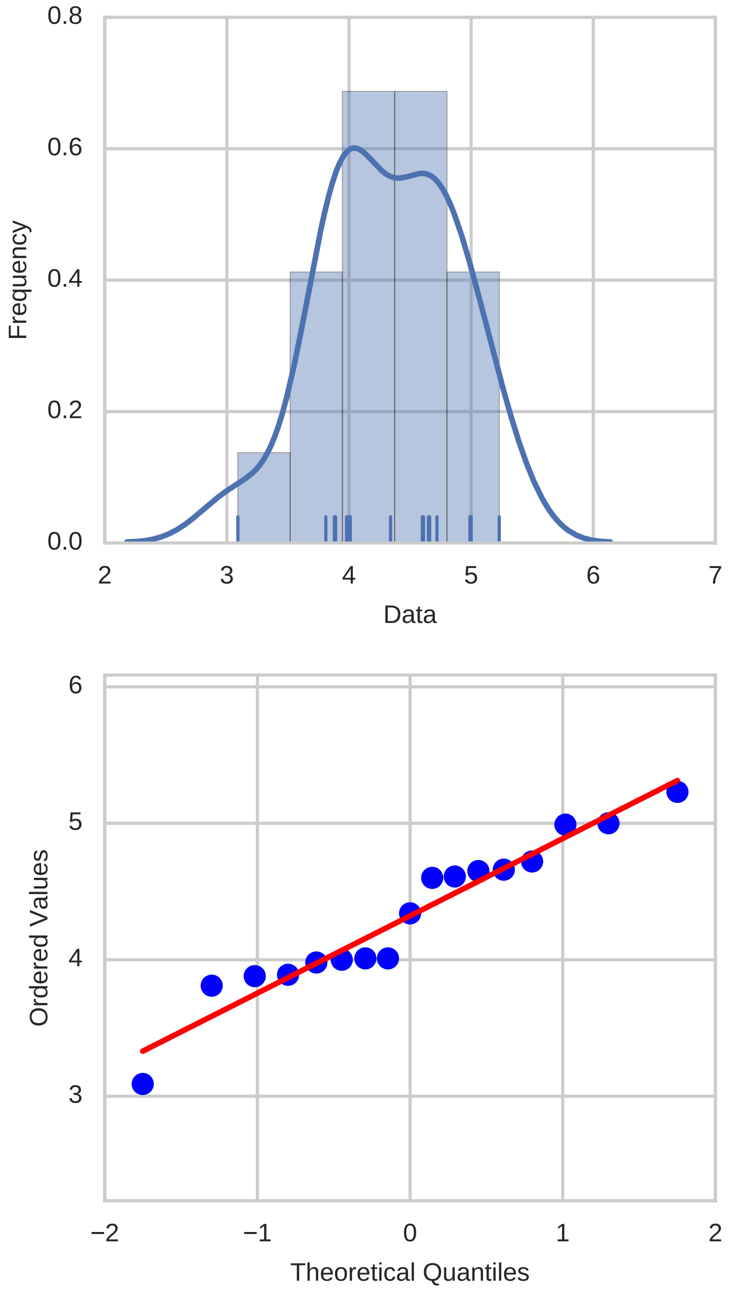

Figure 40: Lipid peroxidation levels for the MPTP disease induced and treated with both *Withania somnifera* and *Centella asiatica* group (LPO_MWC): The data set was found to be normal. The Shapiro Wilk Test-Statistic was 0.944 (Critical Value: 0.892, passed). The Anderson-Darling Test Static was 0.472 (Critical Value: 0.685, passed). The Kolmogorov-Smirnov Test Statistic was 0.191 (Critical Value: 0.318, passed).

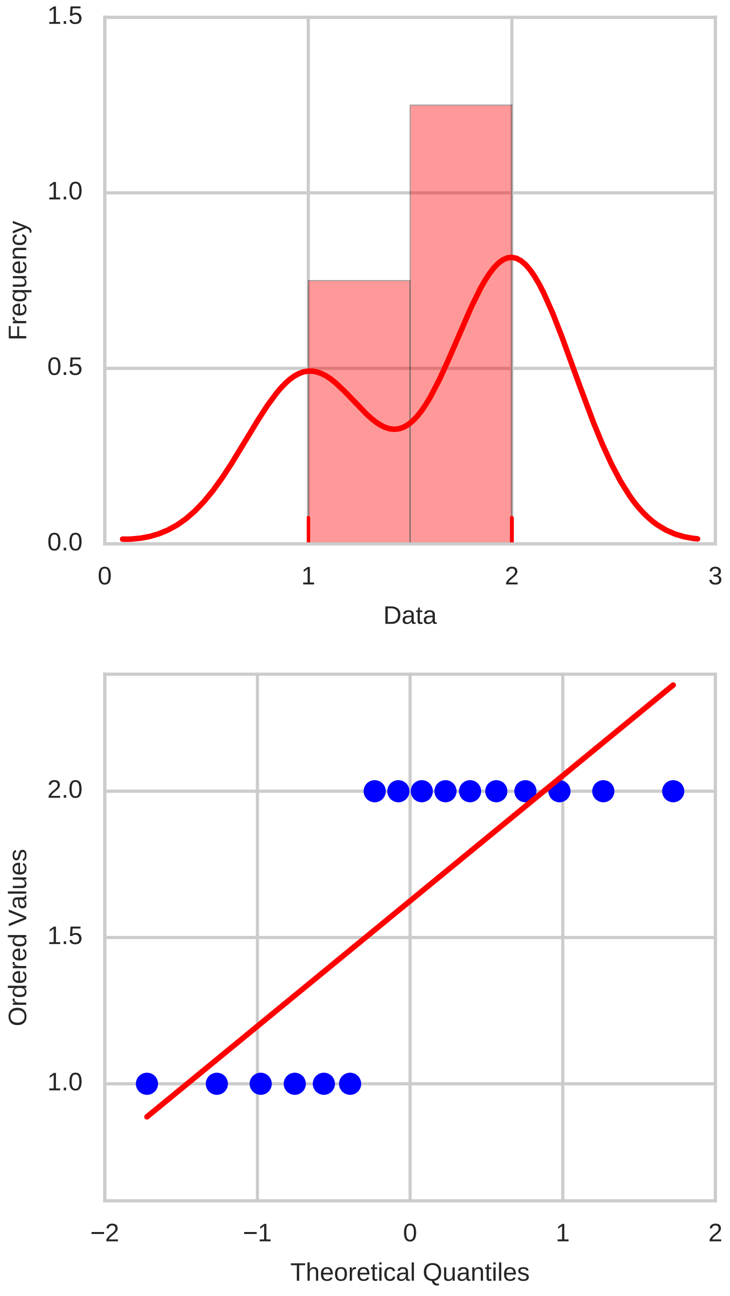

Figure 41: Akinesia results for the untreated group (AU): The data set was found to be non-normal. The Shapiro Wilk Test-Statistic was 0.621 (Critical Value: 0.887, failed). The Anderson-Darling Test Static was 2.957 (Critical Value: 0.683, failed). The Kolmogorov-Smirnov Test Statistic was 0.406 (Critical Value: 0.327, failed).

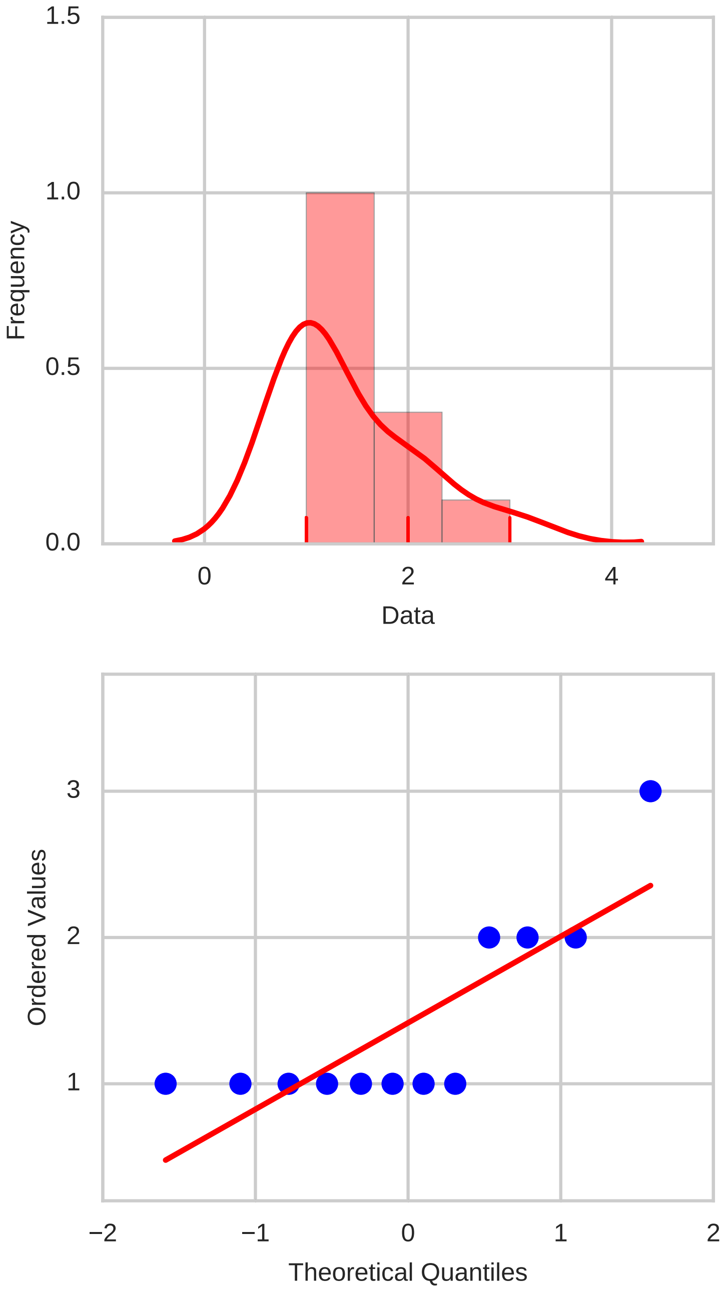

Figure 42: Akinesia results for the group treated with *Centella asiatica* (AC): The data set was found to be non-normal. The Shapiro Wilk Test-Statistic was 0.674 (Critical Value: 0.859, failed). The Anderson-Darling Test Static was 1.844 (Critical Value: 0.679, failed). The Kolmogorov-Smirnov Test Statistic was 0.409 (Critical Value: 0.375, failed).

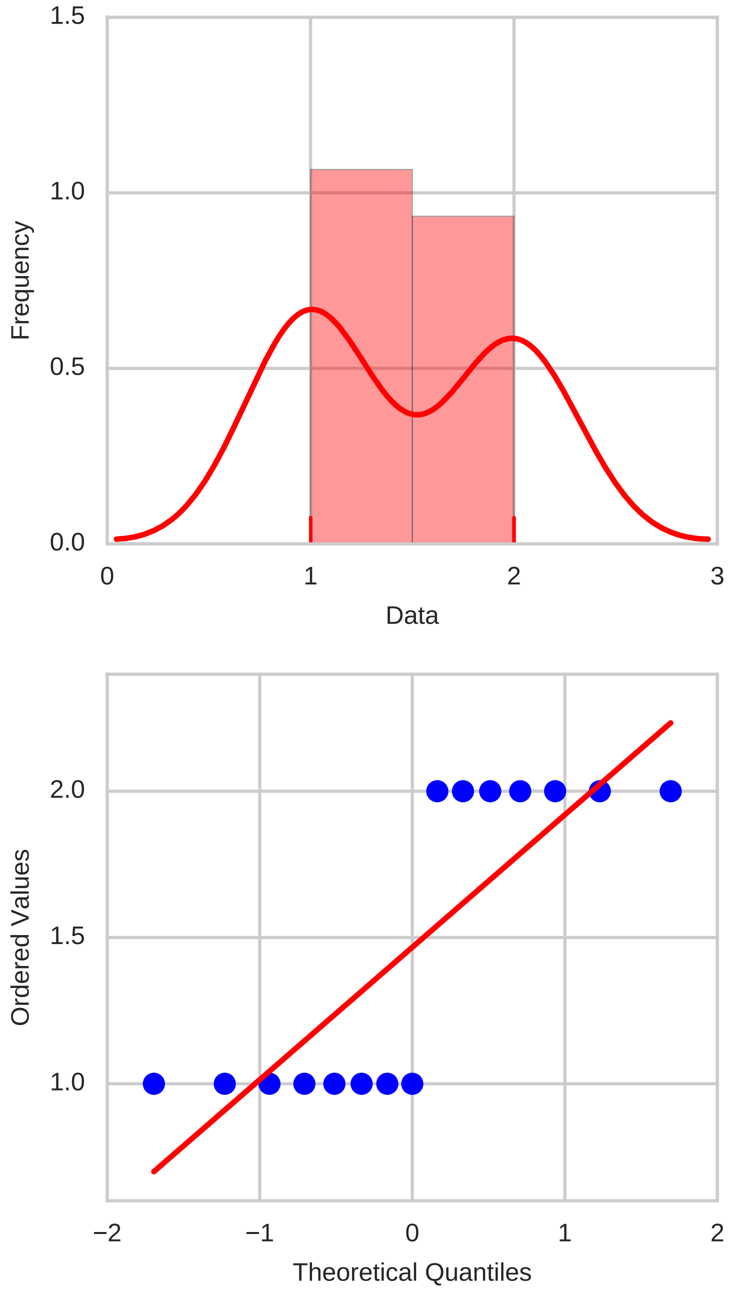

Figure 43: Akinesia results for the group treated with *Withania somnifera* (AW): The data set was found to be non-normal. The Shapiro Wilk Test-Statistic was 0.643 (Critical Value: 0.881, failed). The Anderson-Darling Test Static was 2.551 (Critical Value: 0.681, failed). The Kolmogorov-Smirnov Test Statistic was 0.359 (Critical Value: 0.338, failed).

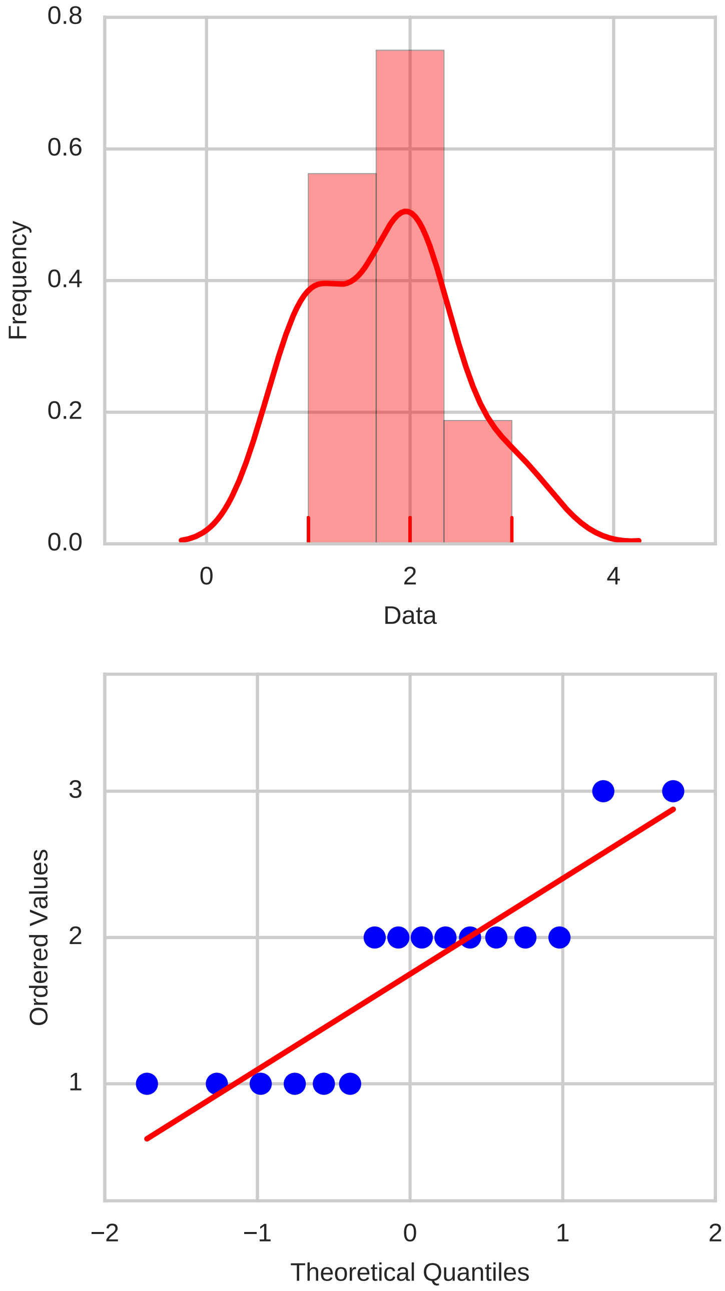

Figure 44: Akinesia results for the group treated with both *Withania somnifera* and *Centella asiatica* (AWC): The data set was found to be non-normal. The Shapiro Wilk Test-Statistic was 0.796 (Critical Value: 0.887, failed). The Anderson-Darling Test Static was 1.486 (Critical Value: 0.683, failed). The Kolmogorov-Smirnov Test Statistic was 0.272 (Critical Value: 0.327, passed).

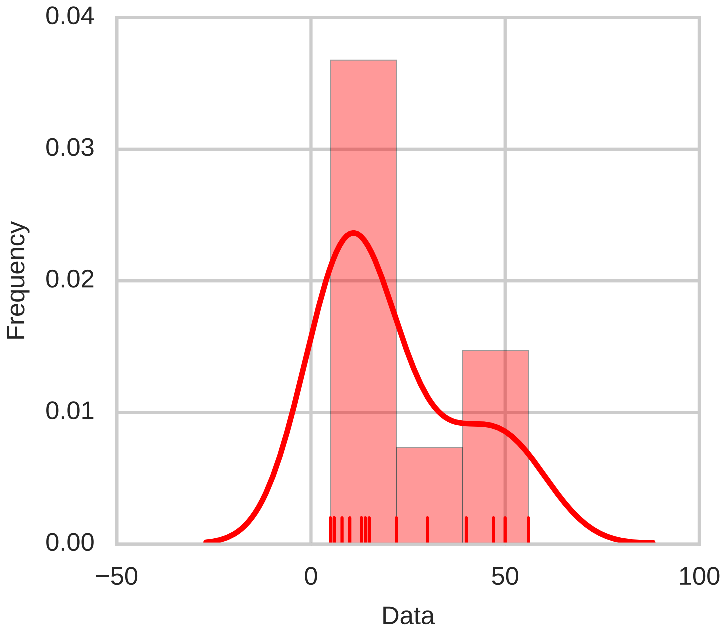

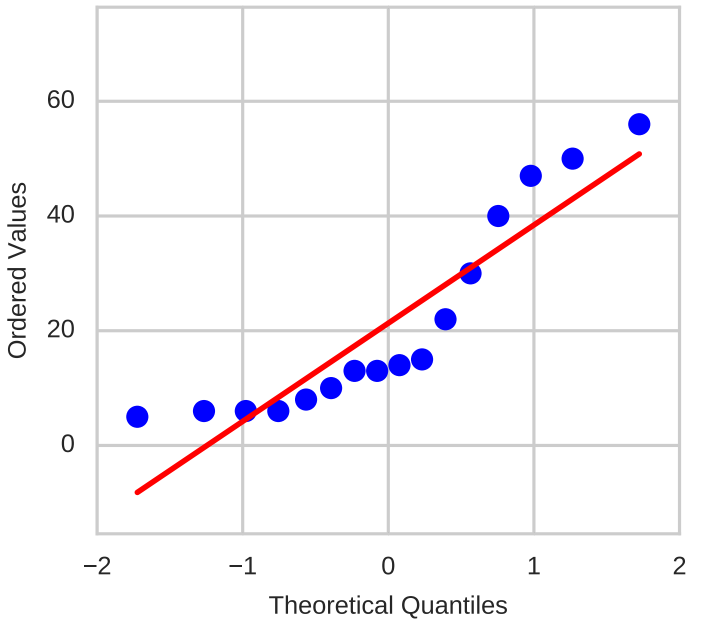

Figure 45: Akinesia results for the MPTP disease induced and no treatment group (AM): The data set was found to be non-normal. The Shapiro Wilk Test-Statistic was 0.825 (Critical Value: 0.887, failed). The Anderson-Darling Test Static was 1.153 (Critical Value: 0.683, failed). The Kolmogorov-Smirnov Test Statistic was 0.270 (Critical Value: 0.327, passed).

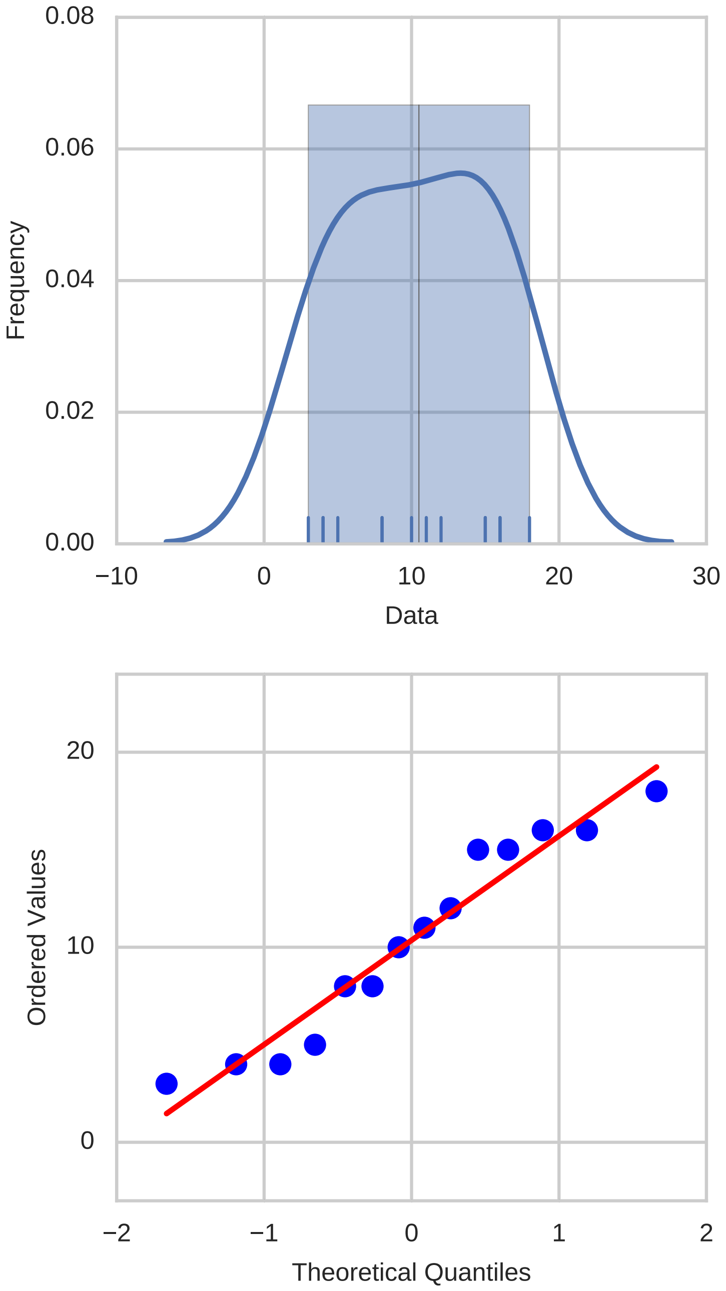

Figure 46: Akinesia results for the MPTP disease induced and treated with *Centella asiatica* group (AMC): The data set was found to be normal. The Shapiro Wilk Test-Statistic was 0.923 (Critical Value: 0.874, passed). The Anderson-Darling Test Static was 0.405 (Critical Value: 0.680, passed). The Kolmogorov-Smirnov Test Statistic was 0.183 (Critical Value: 0.349, passed).

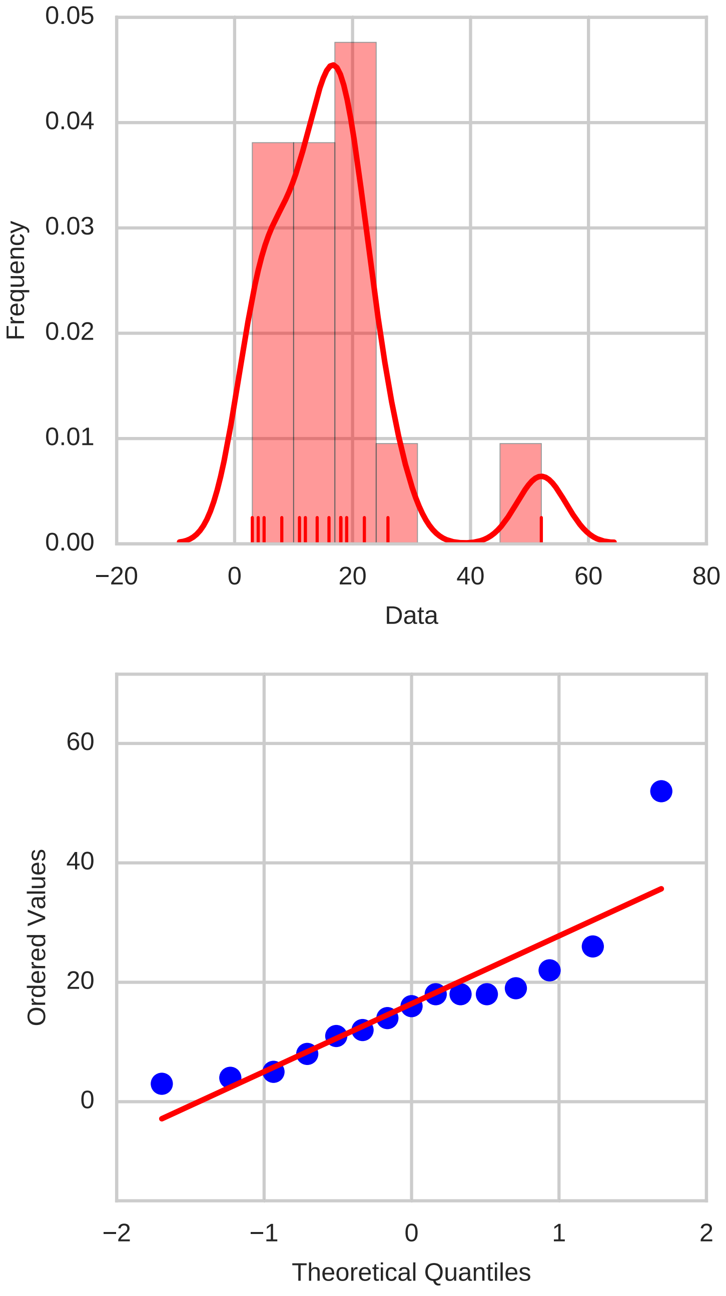

Figure 47: Akinesia results for the MPTP disease induced and treated with *Withania somnifera* group (AMW): The data set was found to be non-normal. The Shapiro Wilk Test-Statistic was 0.821 (Critical Value: 0.881, failed). The Anderson-Darling Test Static was 0.813 (Critical Value: 0.681, failed). The Kolmogorov-Smirnov Test Statistic was 0.211 (Critical Value: 0.338, passed).

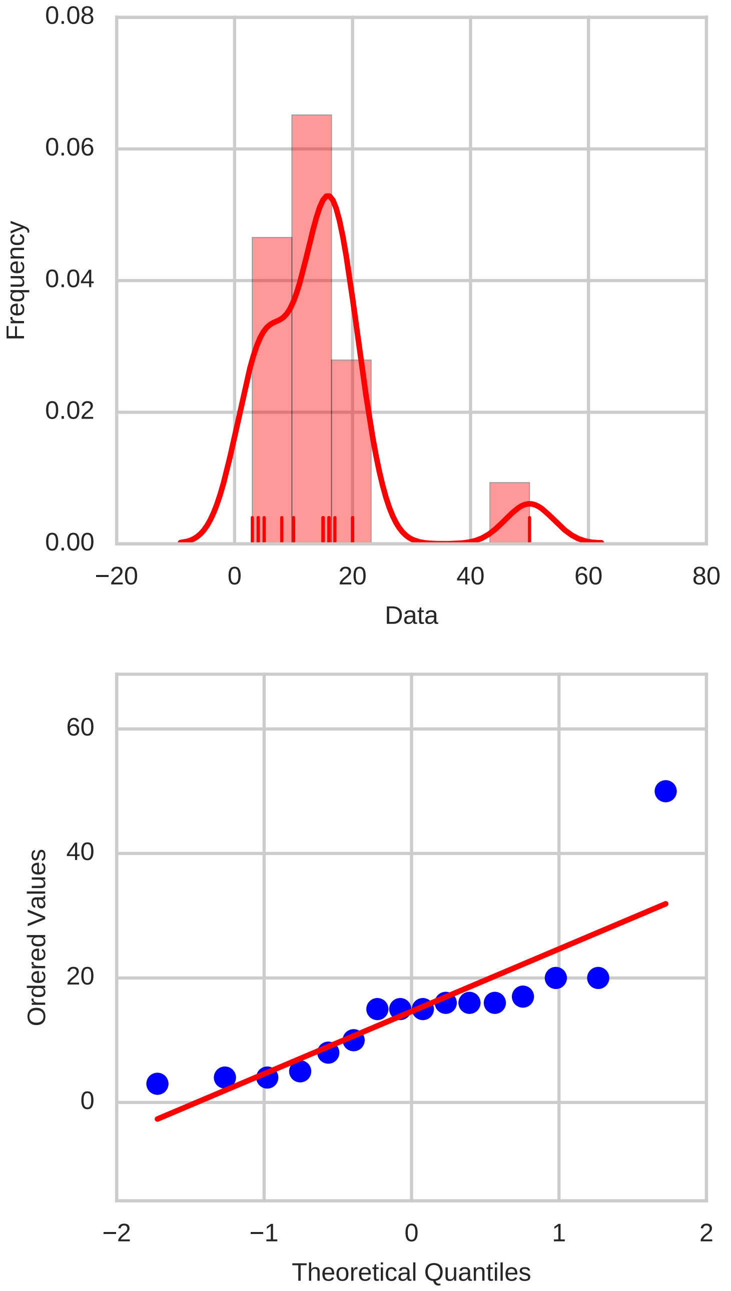

Figure 48: Akinesia results for the MPTP disease induced and treated with both *Withania somnifera* and *Centella asiatica* group (AMWC): The data set was found to be non-normal. The Shapiro Wilk Test-Statistic was 0.752 (Critical Value: 0.887, failed). The Anderson-Darling Test Static was 1.244 (Critical Value: 0.683, failed). The Kolmogorov-Smirnov Test Statistic was 0.246 (Critical Value: 0.327, passed).

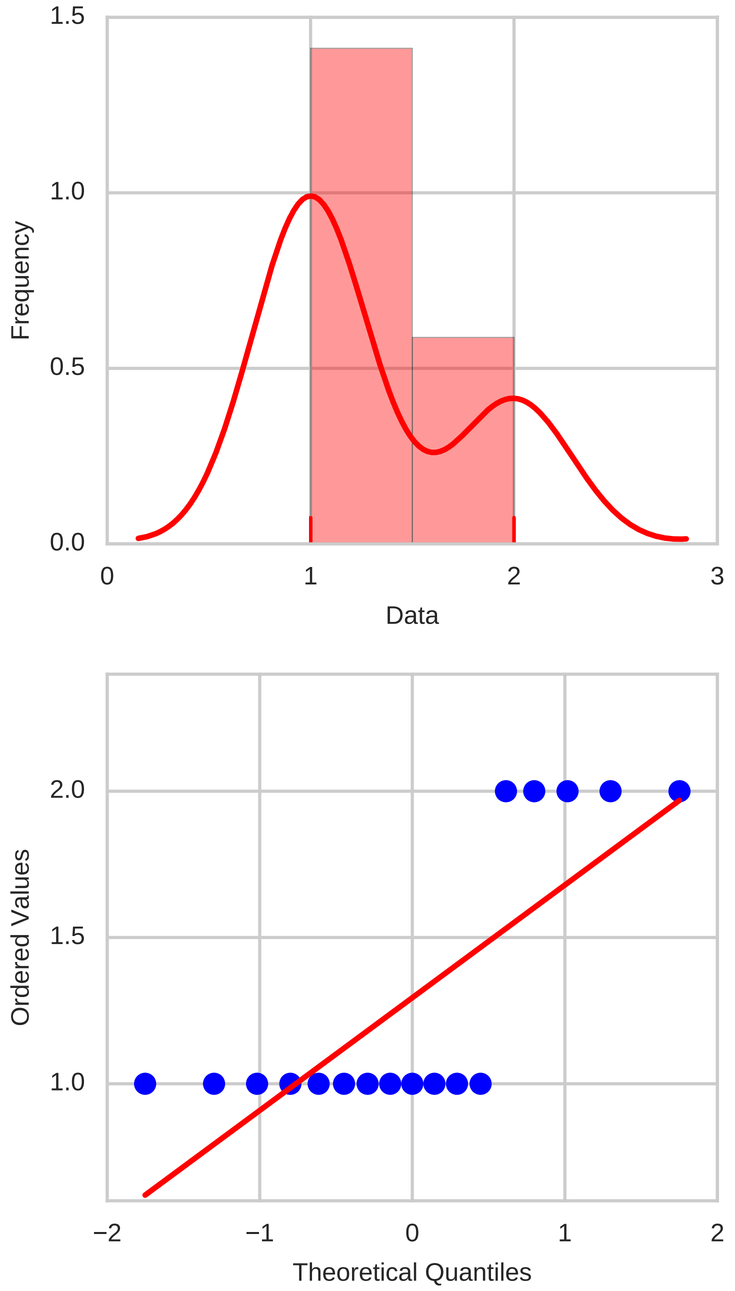

Figure 49: Catalepsy results for the untreated group (CU): The data set was found to be non-normal. The Shapiro Wilk Test-Statistic was 0.579 (Critical Value: 0.892, failed). The Anderson-Darling Test Static was 3.598 (Critical Value: 0.685, failed). The Kolmogorov-Smirnov Test Statistic was 0.447 (Critical Value: 0.318, failed).

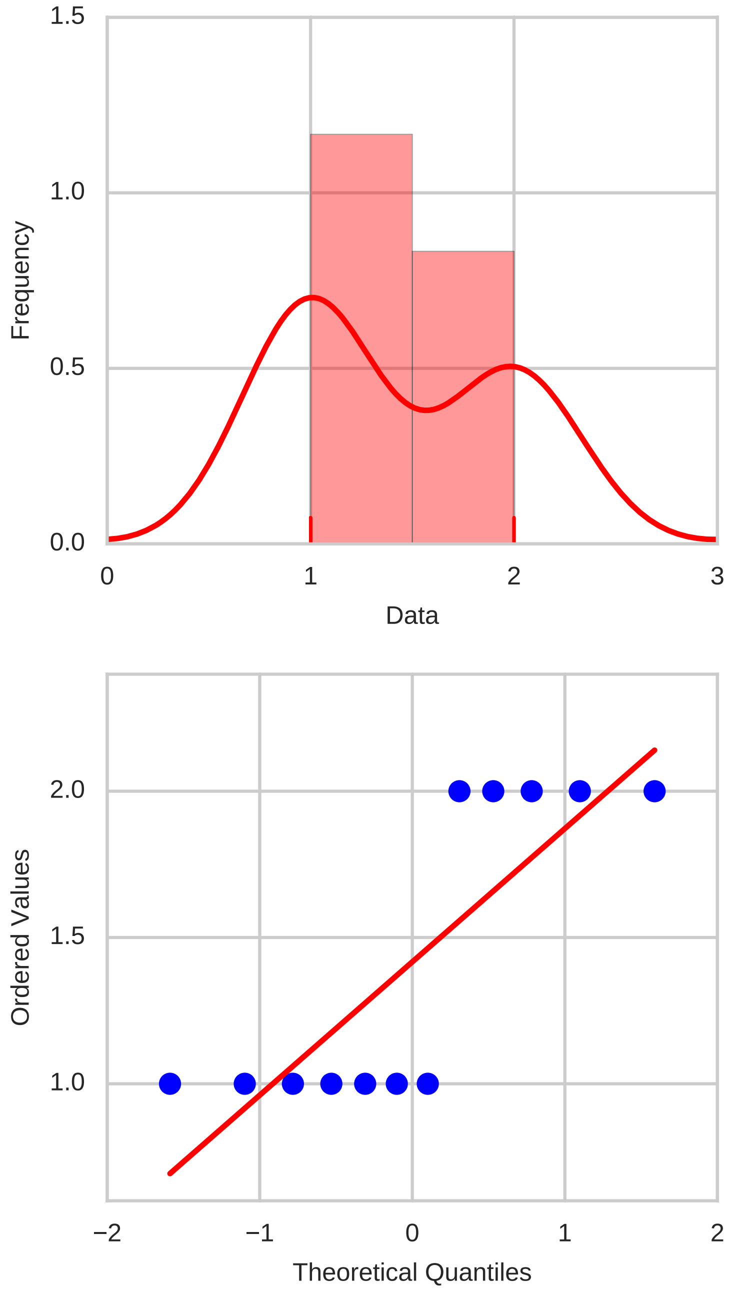

Figure 50: Catalepsy results for the group treated with *Centella asiatica* (CC): The data set was found to be non-normal. The Shapiro Wilk Test-Statistic was 0.640 (Critical Value: 0.859, failed). The Anderson-Darling Test Static was 2.078 (Critical Value: 0.679, failed). The Kolmogorov-Smirnov Test Statistic was 0.384 (Critical Value: 0.375, failed).

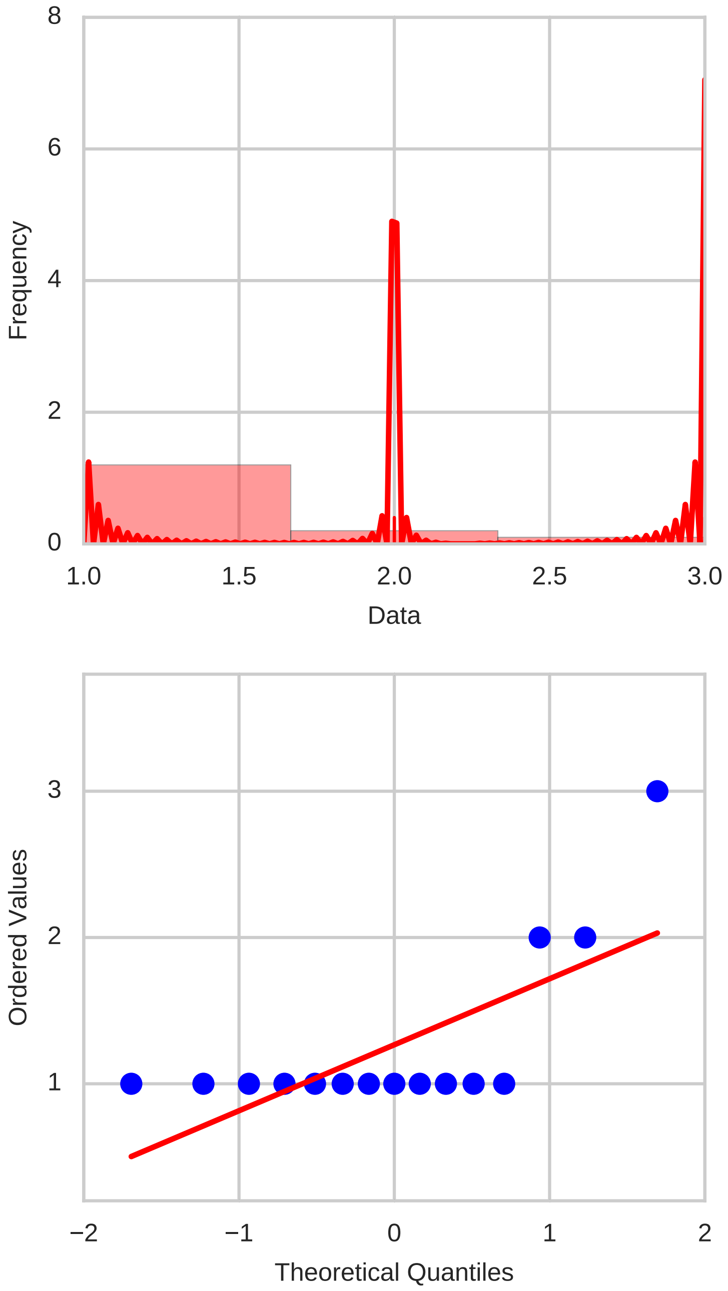

Figure 51: Catalepsy results for the group treated with *Withania somnifera* (CW): The data set was found to be non-normal. The Shapiro Wilk Test-Statistic was 0.525 (Critical Value: 0.881, failed). The Anderson-Darling Test Static was 3.446 (Critical Value: 0.681, failed). The Kolmogorov-Smirnov Test Statistic was 0.479 (Critical Value: 0.338, failed).

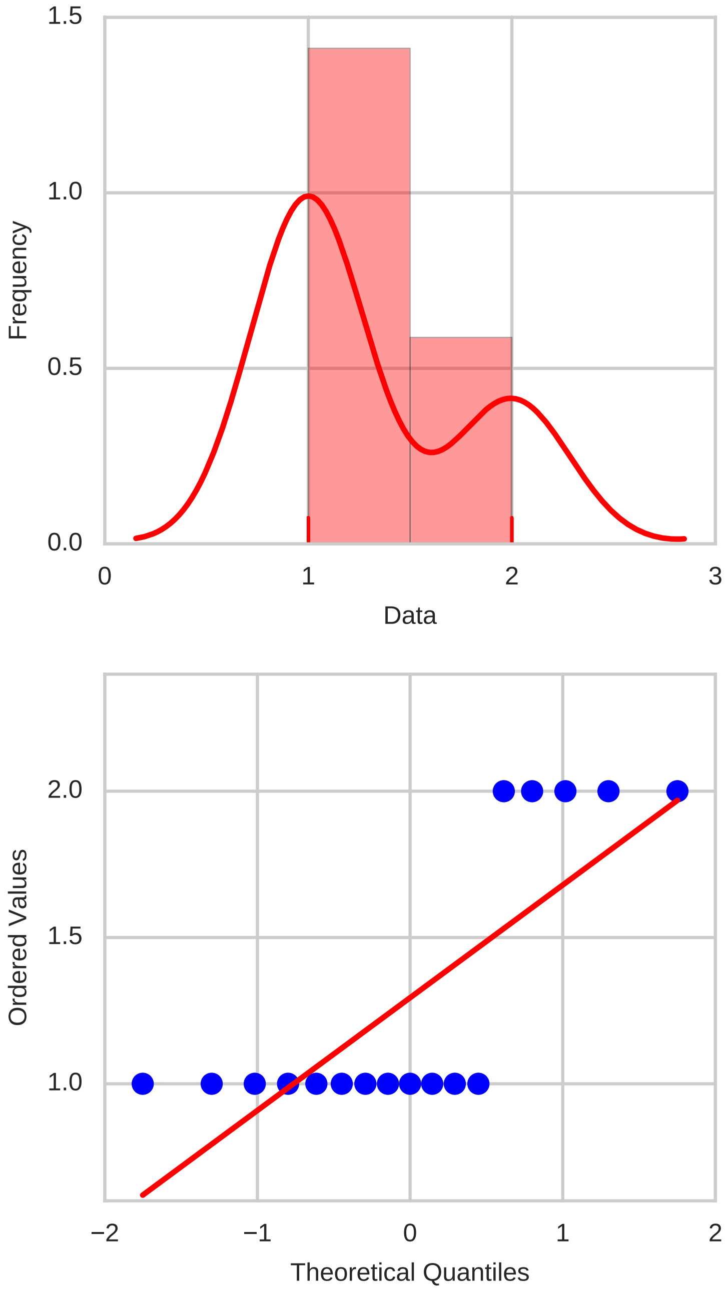

Figure 52: Catalepsy results for the group treated with both *Withania somnifera* and *Centella asiatica* (CWC): The data set was found to be non-normal. The Shapiro Wilk Test-Statistic was 0.579 (Critical Value: 0.892, failed). The Anderson-Darling Test Static was 3.598 (Critical Value: 0.685, failed). The Kolmogorov-Smirnov Test Statistic was 0.447 (Critical Value: 0.318, failed).

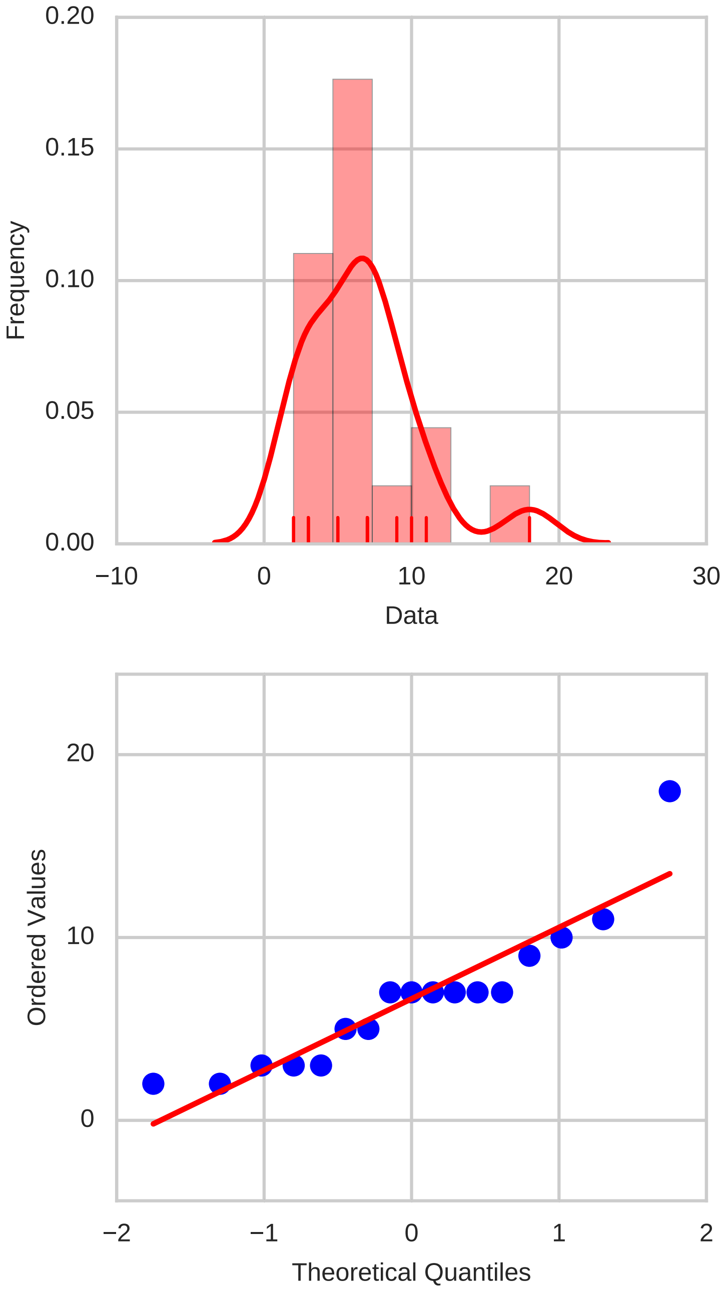

Figure 53: Catalepsy results for the MPTP disease induced and no treatment group (CM): The data set was found to be non-normal. The Shapiro Wilk Test-Statistic was 0.868 (Critical Value: 0.892, failed). The Anderson-Darling Test Static was 0.727 (Critical Value: 0.685, failed). The Kolmogorov-Smirnov Test Statistic was 0.228 (Critical Value: 0.318, passed).

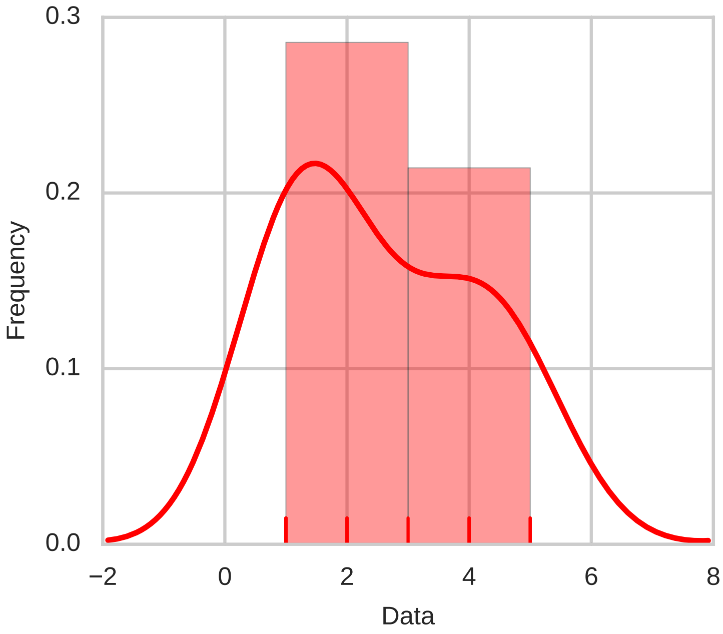

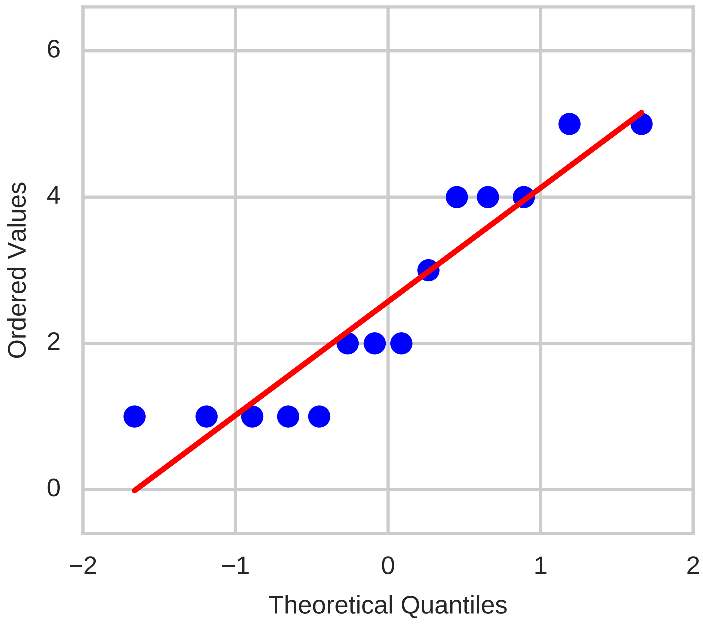

Figure 54: Catalepsy results for the MPTP disease induced and treated with *Centella asiatica* group (CMC): The data set was found to be non-normal. The Shapiro Wilk Test-Statistic was 0.844 (Critical Value: 0.874, failed). The Anderson-Darling Test Static was 0.843 (Critical Value: 0.680, failed). The Kolmogorov-Smirnov Test Statistic was 0.220 (Critical Value: 0.349, passed).

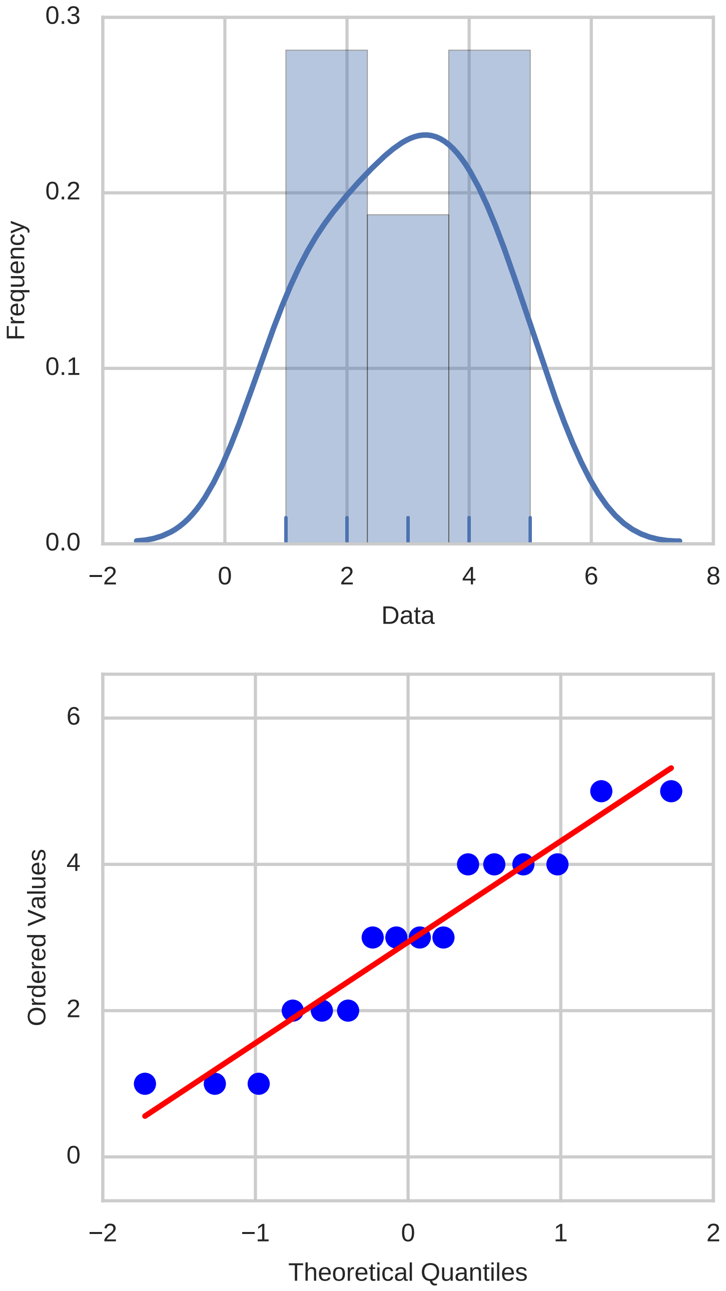

Figure 55: Catalepsy results for the MPTP disease induced and treated with *Withania somnifera* group (CMW): The data set was found to be normal. The Shapiro Wilk Test-Statistic was 0.918 (Critical Value: 0.887, passed). The Anderson-Darling Test Static was 0.486 (Critical Value: 0.683, passed). The Kolmogorov-Smirnov Test Statistic was 0.169 (Critical Value: 0.327, passed).

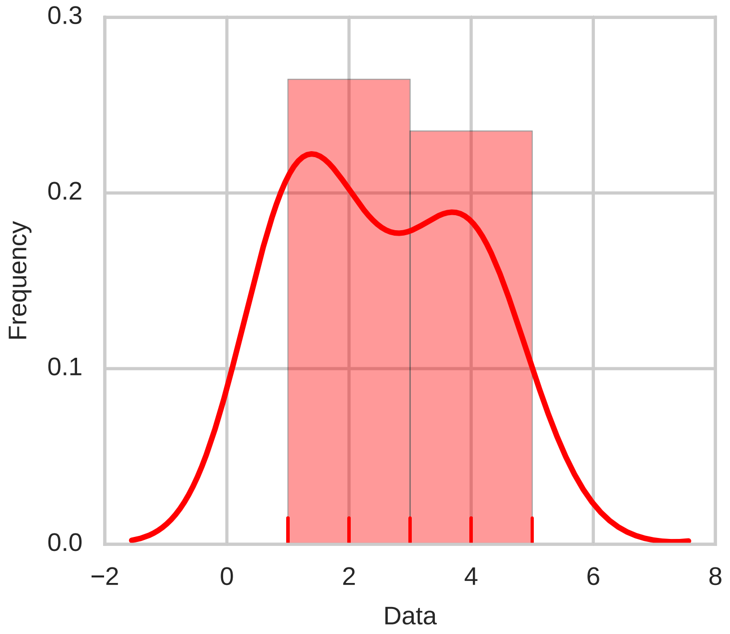

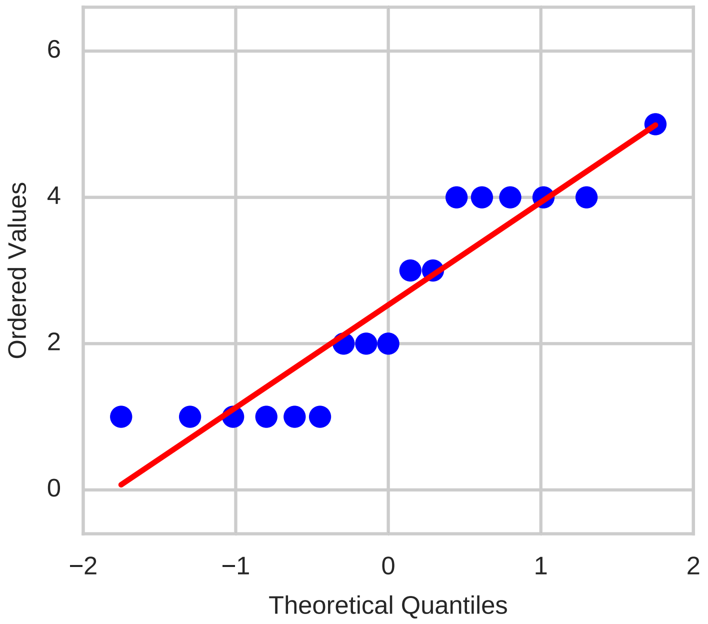

Figure 56: Catalepsy results for the MPTP disease induced and treated with both *Withania somnifera* and *Centella asiatica* group (CMWC): The data set was found to be non-normal. The Shapiro Wilk Test-Statistic was 0.847 (Critical Value: 0.892, failed). The Anderson-Darling Test Static was 1.054 (Critical Value: 0.685, failed). The Kolmogorov-Smirnov Test Statistic was 0.220 (Critical Value: 0.318, passed).

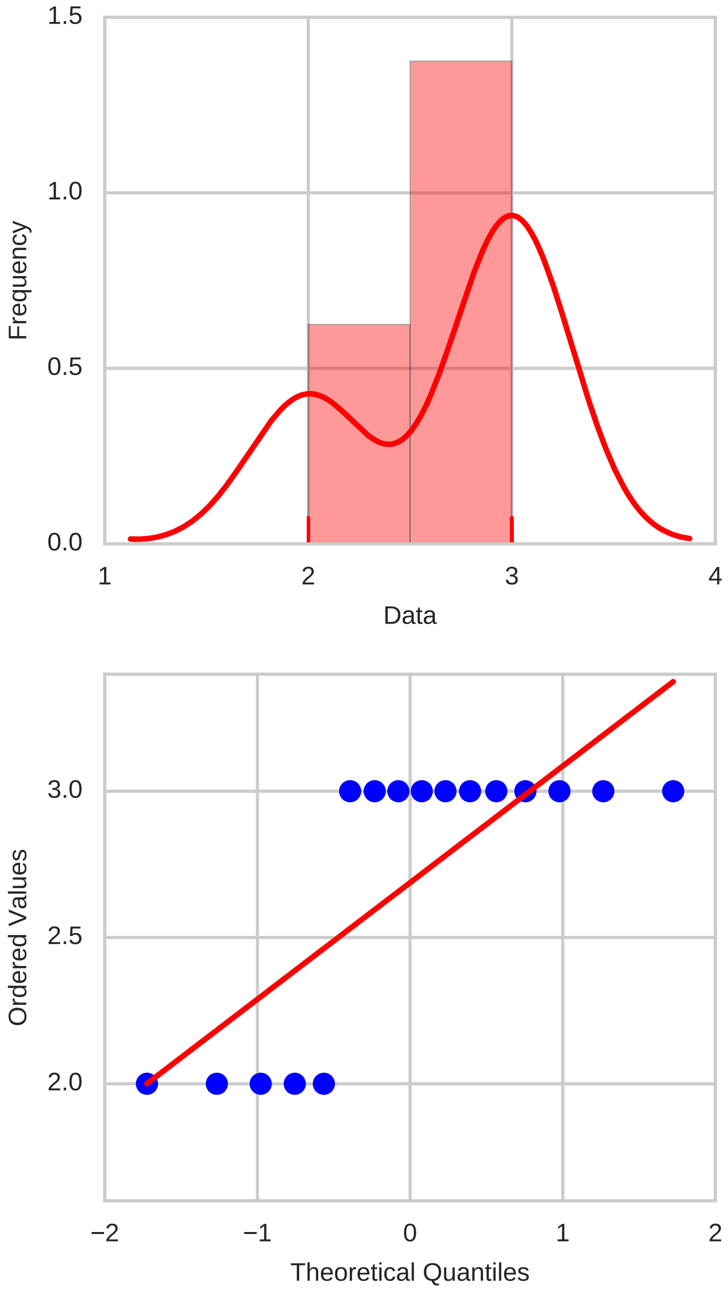

Figure 57: Swim-test results for the untreated group (SU): The data set was found to be non-normal. The Shapiro Wilk Test-Statistic was 0.591 (Critical Value: 0.887, failed). The Anderson-Darling Test Static was 3.264 (Critical Value: 0.683, failed). The Kolmogorov-Smirnov Test Statistic was 0.437 (Critical Value: 0.327, failed).

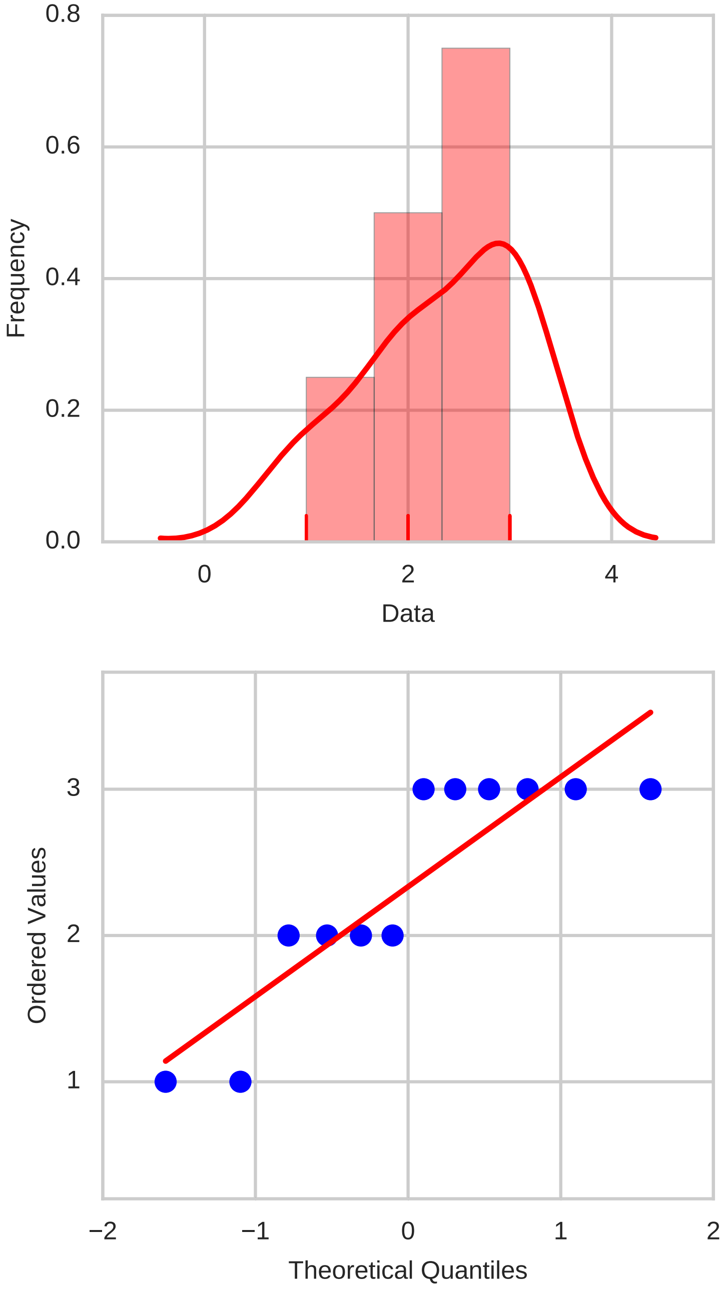

Figure 58: Swim-test results for the group treated with *Centella asiatica* (SC): The data set was found to be non-normal. The Shapiro Wilk Test-Statistic was 0.777 (Critical Value: 0.859, failed). The Anderson-Darling Test Static was 1.153 (Critical Value: 0.679, failed). The Kolmogorov-Smirnov Test Statistic was 0.314 (Critical Value: 0.375, passed).

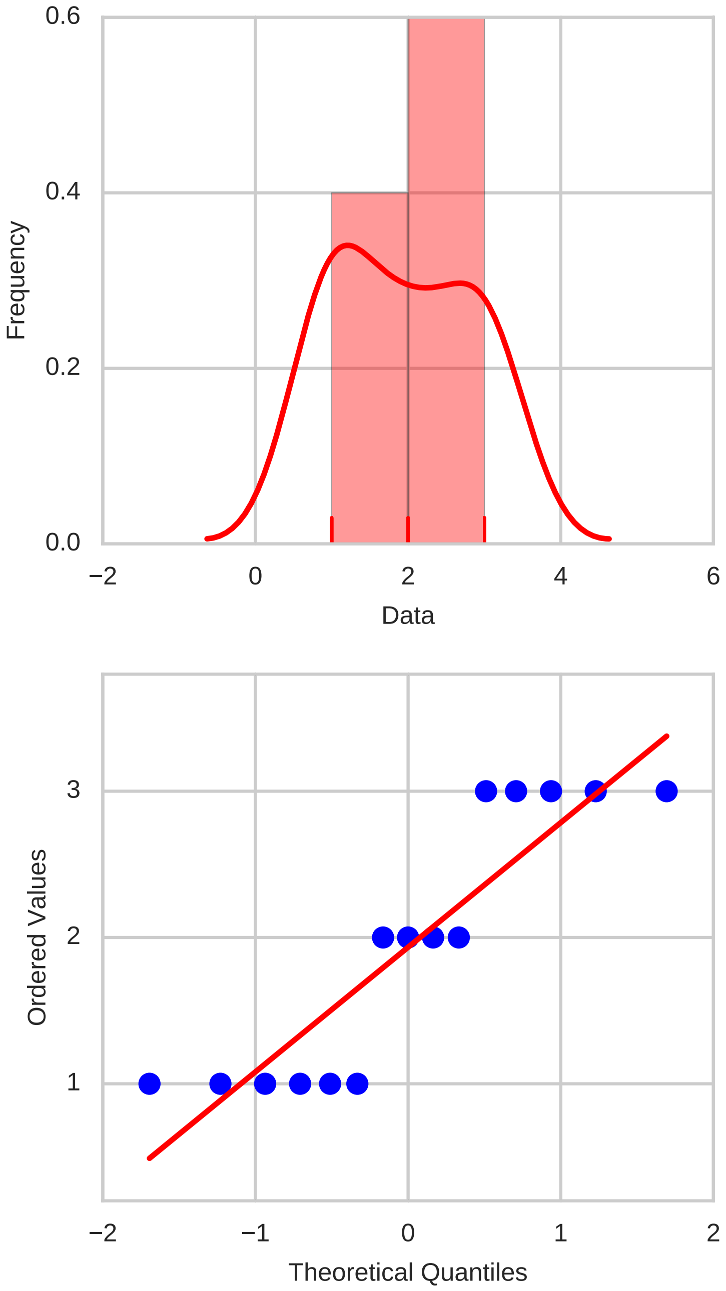

Figure 59: Swim-test results for the group treated with *Withania somnifera* (SW): The data set was found to be non-normal. The Shapiro Wilk Test-Statistic was 0.782 (Critical Value: 0.881, failed). The Anderson-Darling Test Static was 1.293 (Critical Value: 0.681, failed). The Kolmogorov-Smirnov Test Statistic was 0.263 (Critical Value: 0.338, passed).

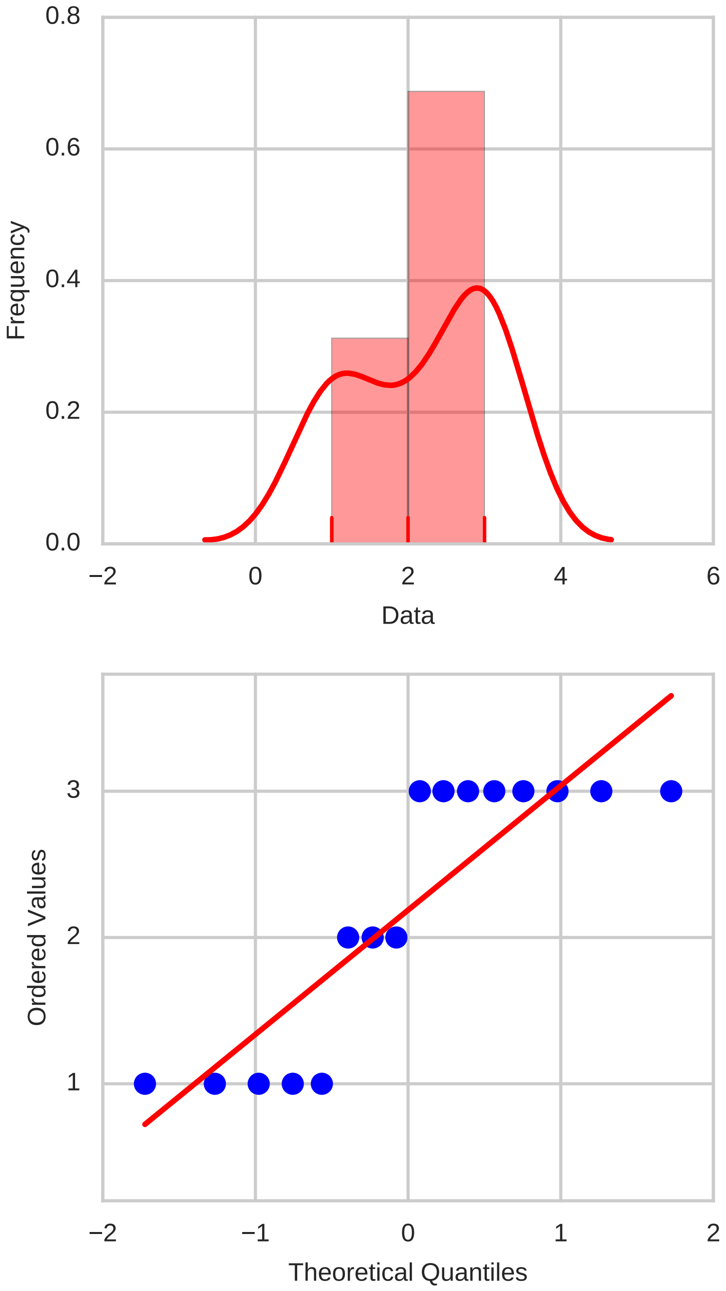

Figure 60: Swim-test results for the group treated with both *Withania somnifera* and *Centella asiatica* (SWC): The data set was found to be non-normal. The Shapiro Wilk Test-Statistic was 0.738 (Critical Value: 0.887, failed). The Anderson-Darling Test Static was 1.764 (Critical Value: 0.683, failed). The Kolmogorov-Smirnov Test Statistic was 0.322 (Critical Value: 0.327, passed).

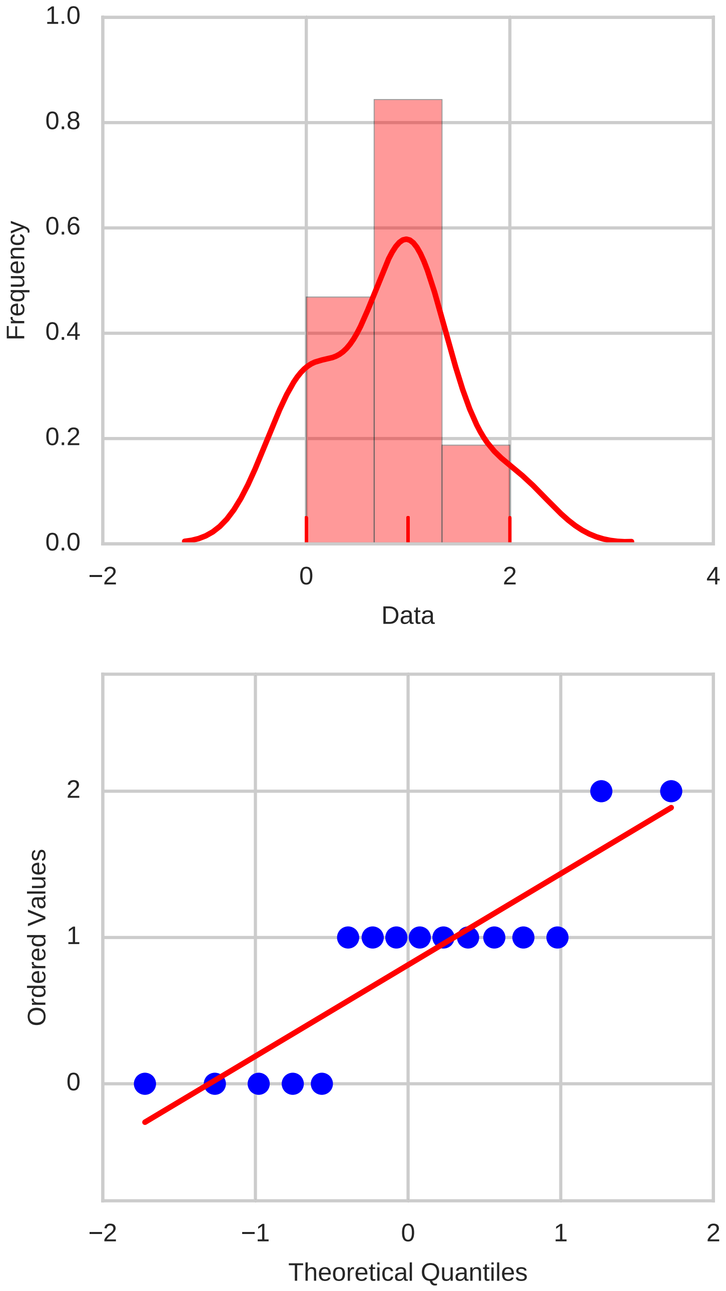

Figure 61: Swim-test results for the MPTP disease induced and no treatment group (SM): The data set was found to be non-normal. The Shapiro Wilk Test-Statistic was 0.794 (Critical Value: 0.887, failed). The Anderson-Darling Test Static was 1.588 (Critical Value: 0.683, failed). The Kolmogorov-Smirnov Test Statistic was 0.304 (Critical Value: 0.327, passed).

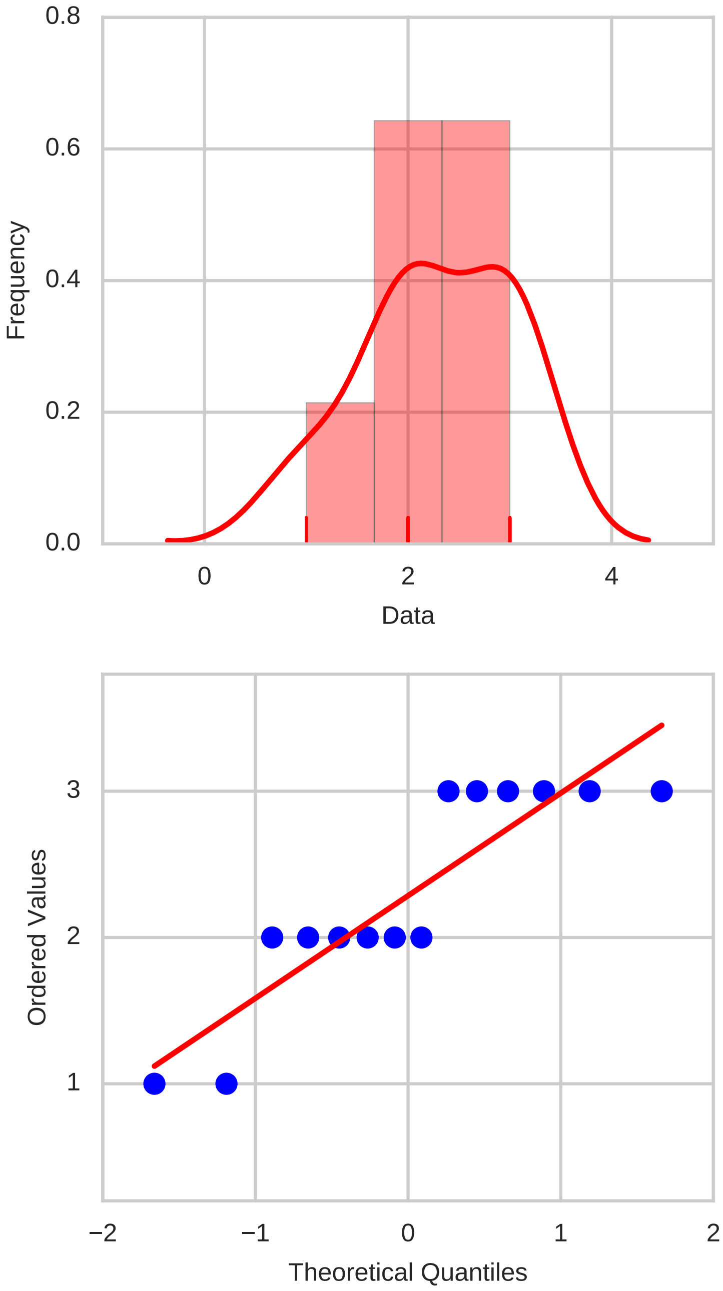

Figure 62: Swim-test results for the MPTP disease induced and treated with *Centella asiatica* group (SMC): The data set was found to be non-normal. The Shapiro Wilk Test-Statistic was 0.796 (Critical Value: 0.874, failed). The Anderson-Darling Test Static was 1.244 (Critical Value: 0.680, failed). The Kolmogorov-Smirnov Test Statistic was 0.275 (Critical Value: 0.349, passed).

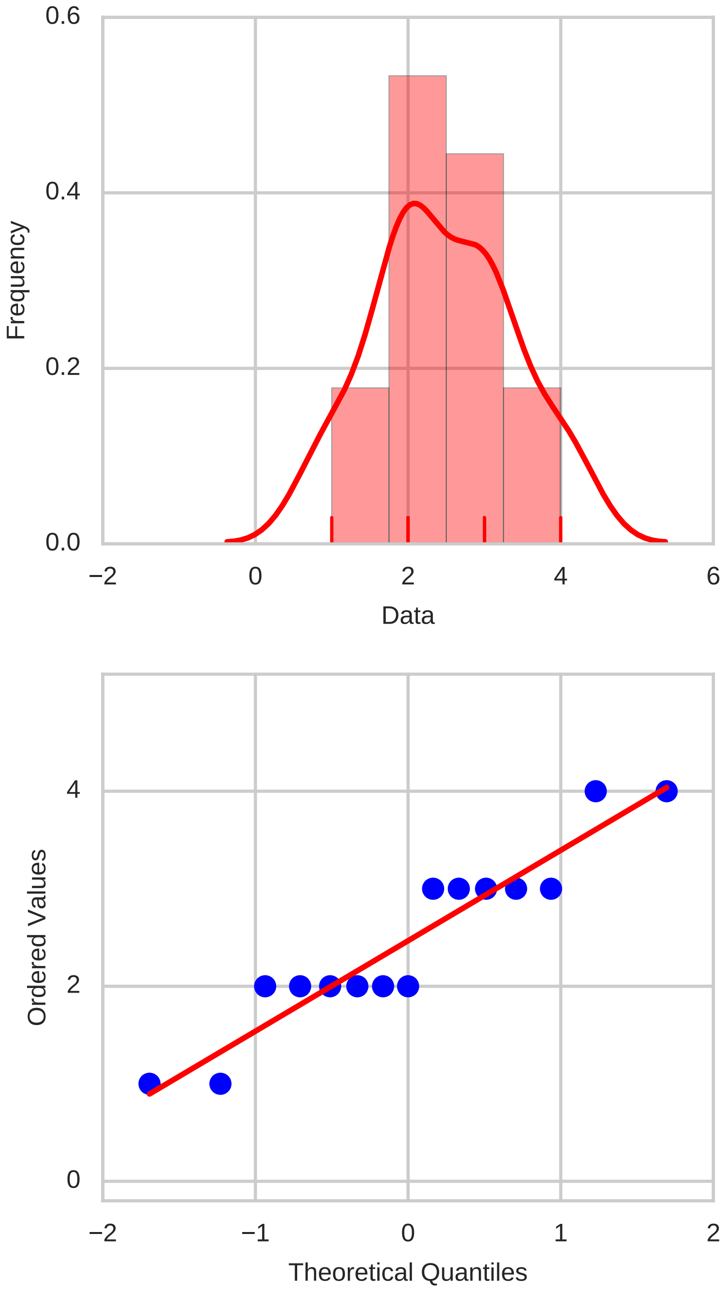

Figure 63: Swim-test results for the MPTP disease induced and treated with *Withania somnifera* group (SMW): The data set was found to be non-normal. The Shapiro Wilk Test-Statistic was 0.896 (Critical Value: 0.881, passed). The Anderson-Darling Test Static was 0.759 (Critical Value: 0.681, failed). The Kolmogorov-Smirnov Test Statistic was 0.234 (Critical Value: 0.338, passed).

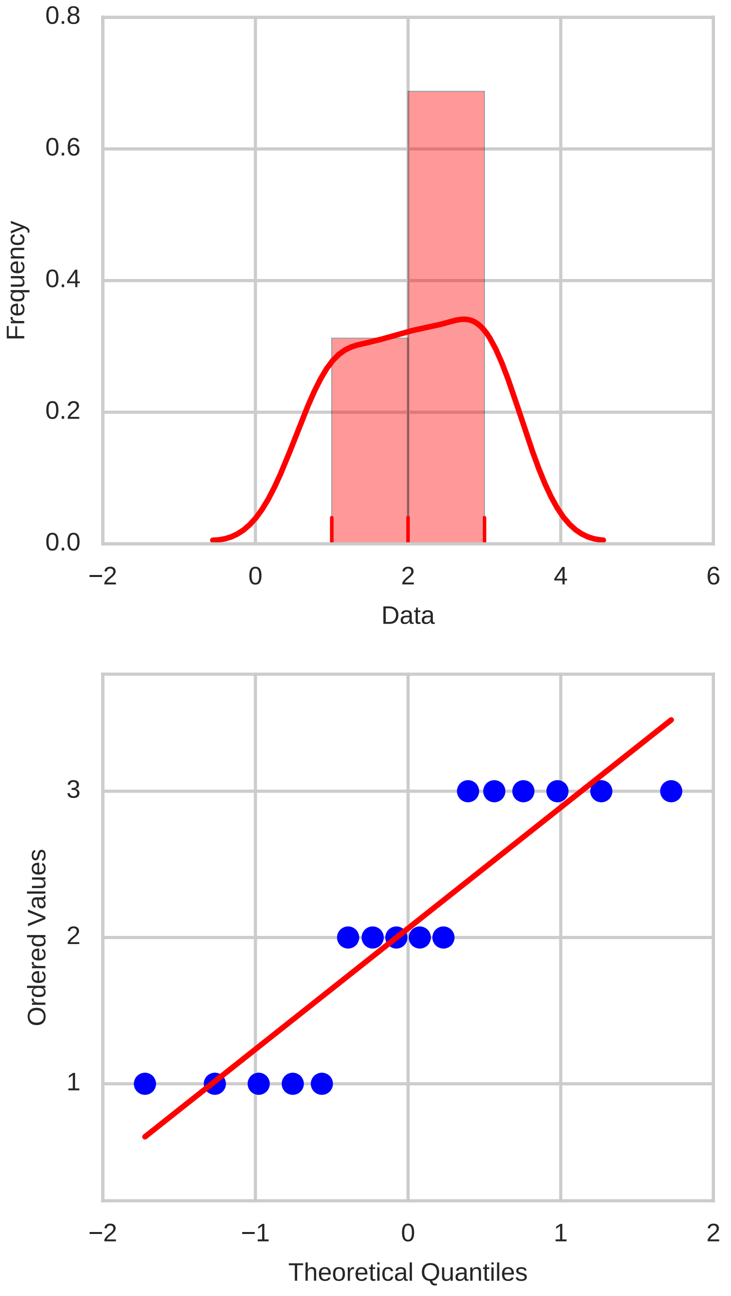

Figure 64: Swim-test results for the MPTP disease induced and treated with both *Withania somnifera* and *Centella asiatica* group (SMWC): The data set was found to be non-normal. The Shapiro Wilk Test-Statistic was 0.796 (Critical Value: 0.887, failed). The Anderson-Darling Test Static was 1.274 (Critical Value: 0.683, failed). The Kolmogorov-Smirnov Test Statistic was 0.247 (Critical Value: 0.327, passed).

DataSet	Size	Skewness	Kurtosis	SW_TS	SW_CV	AD_TS	AD_CV	KS_TS	KS_CV
SOD_U	17	0.103	-1.047	0.957	0.892	0.237	0.685	0.126	0.318
SOD_C	12	0.075	-0.777	0.961	0.859	0.231	0.679	0.138	0.375
SOD_W	15	-0.239	-0.585	0.951	0.881	0.338	0.681	0.140	0.338
SOD_WC	17	-0.761	-0.414	0.917	0.892	0.511	0.685	0.165	0.318
SOD_M	17	-0.565	-0.209	0.955	0.892	0.319	0.685	0.166	0.318
SOD_MC	14	0.168	-0.631	0.898	0.874	0.756	0.680	0.242	0.349
SOD_MW	16	-1.000	0.348	0.906	0.887	0.600	0.683	0.227	0.327
SOD_MWC	17	-1.064	0.581	0.902	0.892	0.659	0.685	0.239	0.318
CAT_U	17	0.866	2.342	0.900	0.892	0.672	0.685	0.162	0.318
CAT_C	12	-0.569	-0.491	0.946	0.859	0.330	0.679	0.174	0.375
CAT_W	15	-0.909	0.466	0.930	0.881	0.464	0.681	0.178	0.338
CAT_WC	17	-1.403	1.327	0.846	0.892	1.026	0.685	0.206	0.318
CAT_M	17	1.281	2.729	0.874	0.892	0.661	0.685	0.210	0.318
CAT_MC	14	-0.705	-0.078	0.951	0.874	0.256	0.680	0.122	0.349
CAT_MW	16	0.315	-1.058	0.950	0.887	0.283	0.683	0.149	0.327
CAT_MWC	17	0.389	-1.052	0.938	0.892	0.359	0.685	0.153	0.318
GPx_U	17	1.304	1.701	0.887	0.892	0.542	0.685	0.140	0.318
GPx_C	12	-0.417	-0.973	0.931	0.859	0.430	0.679	0.191	0.375
GPx_W	15	-0.215	-0.983	0.956	0.881	0.262	0.681	0.139	0.338
GPx_WC	17	0.091	-0.801	0.952	0.892	0.351	0.685	0.116	0.318
GPx_M	17	-0.129	-1.286	0.926	0.892	0.483	0.685	0.169	0.318
GPx_MC	17	-1.976	2.893	0.628	0.892	3.037	0.685	0.418	0.318
GPx_MW	16	-0.275	0.073	0.950	0.887	0.495	0.683	0.219	0.327
GPx_MWC	17	-0.423	-0.177	0.941	0.892	0.543	0.685	0.221	0.318
GSH_U	17	0.063	-1.104	0.962	0.892	0.255	0.685	0.141	0.318
GSH_C	12	0.047	-0.889	0.977	0.859	0.143	0.679	0.105	0.375
GSH_W	15	-0.154	-0.742	0.969	0.881	0.235	0.681	0.138	0.338
GSH_WC	17	0.061	-0.632	0.964	0.892	0.245	0.685	0.110	0.318
GSH_M	17	-0.413	-0.783	0.945	0.892	0.367	0.685	0.162	0.318
GSH_MC	14	0.179	-0.617	0.972	0.874	0.205	0.680	0.113	0.349
GSH_MW	16	-0.678	0.198	0.945	0.887	0.345	0.683	0.177	0.327
GSH_MWC	17	-0.666	0.357	0.945	0.892	0.373	0.685	0.194	0.318
LPO_U	17	-0.487	-0.845	0.923	0.892	0.474	0.685	0.160	0.318
LPO_C	12	0.438	-0.489	0.948	0.859	0.334	0.679	0.188	0.375
LPO_W	15	0.281	-0.245	0.965	0.881	0.318	0.681	0.142	0.338
LPO_WC	17	0.165	-0.846	0.965	0.892	0.235	0.685	0.118	0.318
LPO_M	17	0.325	-0.356	0.973	0.892	0.238	0.685	0.116	0.318
LPO_MC	14	-0.291	-0.291	0.958	0.874	0.275	0.680	0.146	0.349
LPO_MW	16	-0.396	-0.353	0.945	0.887	0.425	0.683	0.182	0.327
LPO_MWC	17	-0.294	-0.367	0.944	0.892	0.472	0.685	0.191	0.318
AU	16	-0.516	-1.733	0.621	0.887	2.957	0.683	0.406	0.327
AC	12	1.267	0.412	0.674	0.859	1.844	0.679	0.409	0.375
AW	15	0.134	-1.982	0.643	0.881	2.551	0.681	0.359	0.338
AWC	16	0.324	-0.776	0.796	0.887	1.486	0.683	0.272	0.327
AM	16	0.877	-0.759	0.825	0.887	1.153	0.683	0.270	0.327
AMC	14	-0.048	-1.400	0.923	0.874	0.405	0.680	0.183	0.349
AMW	15	1.721	3.396	0.821	0.881	0.813	0.681	0.211	0.338
AMWC	16	1.995	4.669	0.752	0.887	1.244	0.683	0.246	0.327
CU	17	0.904	-1.183	0.579	0.892	3.598	0.685	0.447	0.318
CC	12	0.338	-1.886	0.640	0.859	2.078	0.679	0.384	0.375
CW	15	2.039	2.957	0.525	0.881	3.446	0.681	0.479	0.338
CWC	17	0.904	-1.183	0.579	0.892	3.598	0.685	0.447	0.318

CM	17	1.265	1.907	0.868	0.892	0.727	0.685	0.228	0.318
CMC	14	0.372	-1.400	0.844	0.874	0.843	0.680	0.220	0.349
CMW	16	-0.056	-1.106	0.918	0.887	0.486	0.683	0.169	0.327
CMWC	17	0.209	-1.465	0.847	0.892	1.054	0.685	0.220	0.318
SU	16	-0.809	-1.345	0.591	0.887	3.264	0.683	0.437	0.327
SC	12	-0.626	-0.960	0.777	0.859	1.153	0.679	0.314	0.375
SW	15	0.128	-1.616	0.782	0.881	1.293	0.681	0.263	0.338
SWC	16	-0.374	-1.611	0.738	0.887	1.764	0.683	0.322	0.327
SM	16	0.178	-0.619	0.794	0.887	1.588	0.683	0.304	0.327
SMC	14	-0.459	-0.896	0.796	0.874	1.244	0.680	0.275	0.349
SMW	15	0.101	-0.712	0.896	0.881	0.759	0.681	0.234	0.338
SMWC	16	-0.117	-1.528	0.796	0.887	1.274	0.683	0.247	0.327

*The cells in red show the tests which were failed.

TS: Test Statistic

CV: Critical Value

SW: Shapiro-Wilk Test

AD: Anderson Darling Test

KS: Kolmogorov-Smirnov Test
