# Supplementary material for: Complete Comparison Display (CCD) evaluation of ethanol extracts of Centella asiatica and Withania somnifera shows that they can non-synergistically ameliorate biochemical and behavioural damages in MPTP induced Parkinson's model of mice
Source: PLoS One. 2017 May 16;12(5):e0177254. doi: 10.1371/journal.pone.0177254 (PMC5433711; doi:10.1371/journal.pone.0177254)
Supplement: S2 Fig — Levene’s test was used to determine if the two datasets have similar variances. When both datasets were normal, we used a student’s t-test if the variances were similar and a Welch’s t test otherwise to test significance. For one or both non-normal datasets being compared with similar variance, we used a Mann Whitney U Test. If the variances were not similar for non-normal datasets we used Welch’s U Test. (PDF) [file pone.0177254.s002.pdf]

Significance Tests of Datasets

For every property that was studied, all the groups were compared against each other to understand the effect of the treatment. We studied if two datasets can be assumed to be coming from ditributions with different means. The null hypothesis taken was that both the datasets come from distributions with the same mean. We used a Levene's test to test for all combinations if the variance of the datasets is the same. For normal data sets, we took a student's t-test to test for significance if the variance was same, and used a Welch's t-test otherwise. For non-normal datasets, we used a Mann-Whitney U test if the variance was same. If the variance was not same for non-normal datasets, we ranked the data and used a welch's t-test on ranked data. A confidence interval of 95% was chosen. The histograms and the estimated distribution line of two datasets that were being compared together are presented in the same plot (Figure 1 to 224). All the datasets that were found to be normal are shown in red or dark red while those that were found to be non-normal are shown in blue or dark blue. The tests in which the null hypothesis has been rejected are shown with a gray background while those in which the null hypothesis could not be rejected are shown with a white background. Further, two tables have been presented in the end. The first table shows the results of the levene test while the second table shows the relevant significance tests and their results.

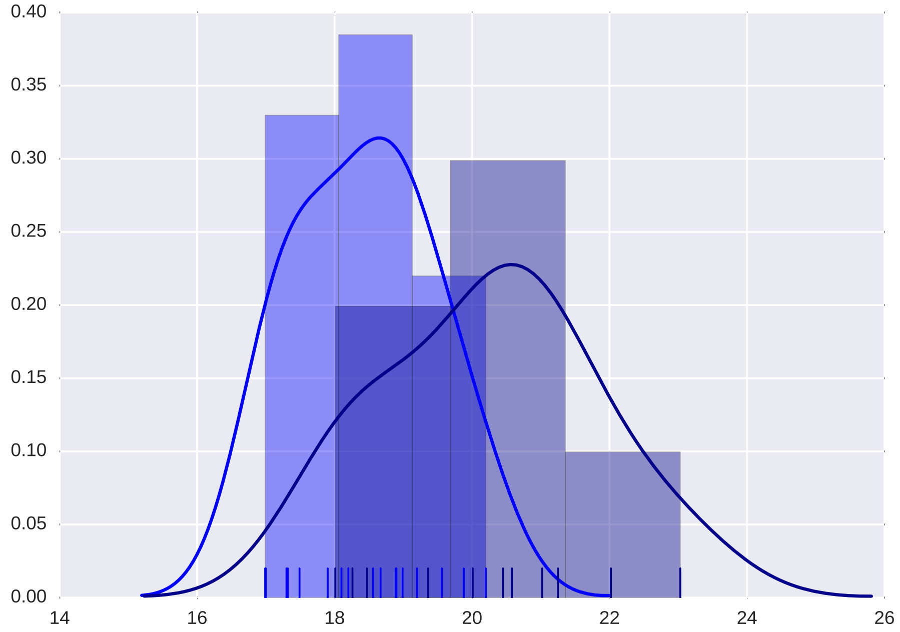

Figure 1: Significance Test Results for Superoxide dismutase levels between the untreated group(SOD_U) and the group treated with *Centella asiatica*(SOD_C). As both the sets were normal, a Student's T-test was used. The p-value obtained for the test was 0.0006 and hence the difference between the data sets was considered statistically significant.

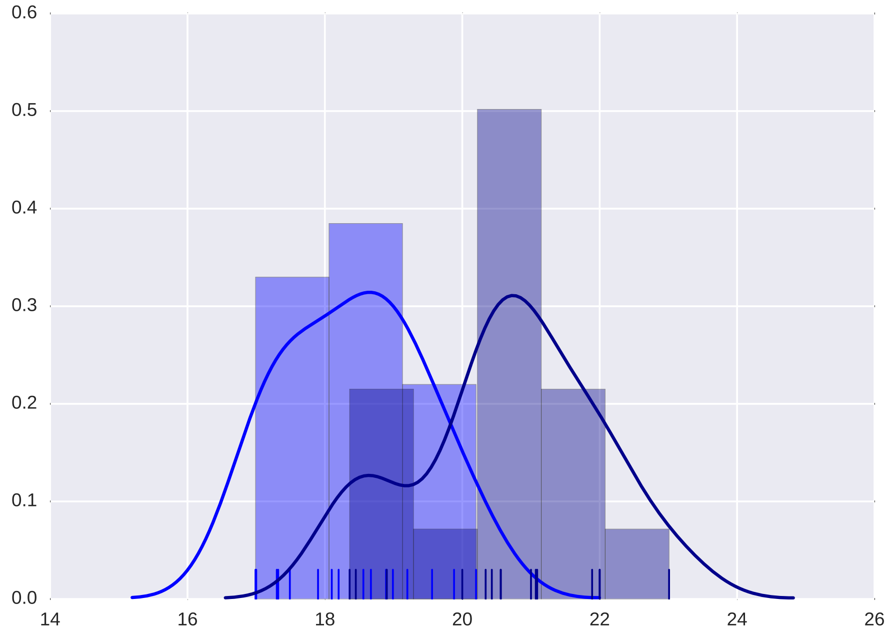

Figure 2: Significance Test Results for Superoxide dismutase levels between the untreated group(SOD_U) and the group treated with *Withania somnifera*(SOD_W). As both the sets were normal, a Student's T-test was used. The p-value obtained for the test was 0.0000 and hence the difference between the data sets was considered statistically significant.

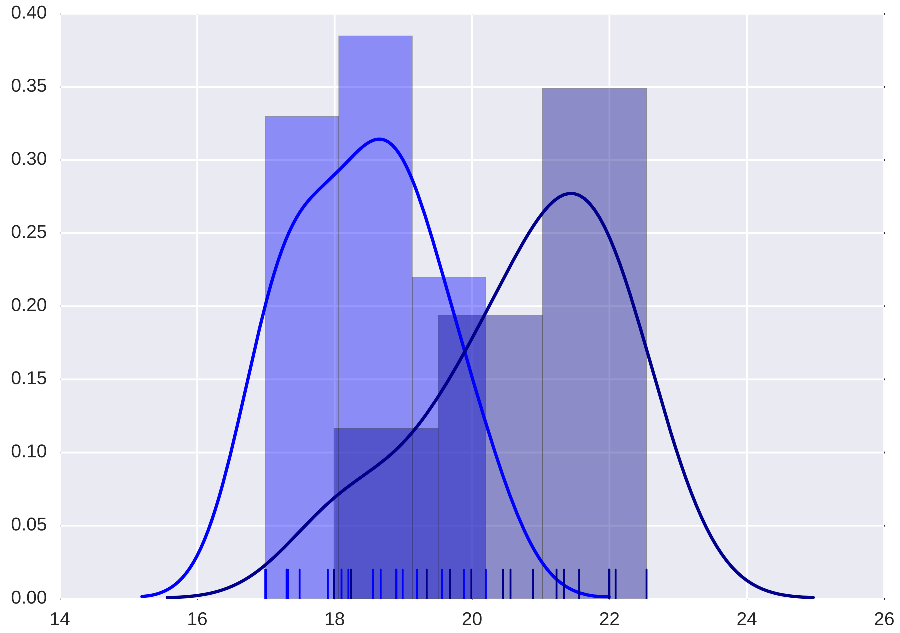

Figure 3: Significance Test Results for Superoxide dismutase levels between the untreated group(SOD_U) and the group treated with both *Withania somnifera* and *Centella asiatica*(SOD_WC). As both the sets were normal, a Student's T-test was used. The p-value obtained for the test was 0.0000 and hence the difference between the data sets was considered statistically significant.

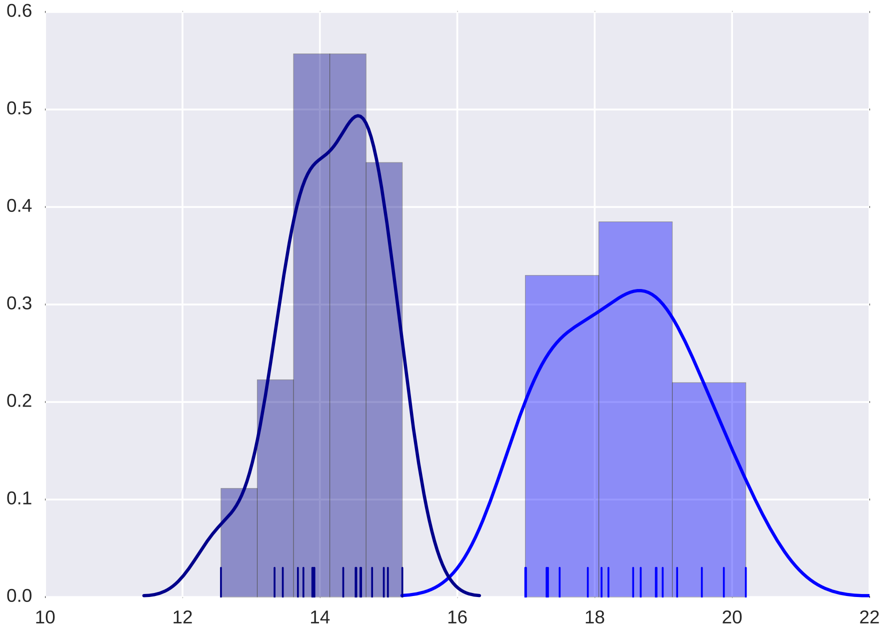

Figure 4: Significance Test Results for Superoxide dismutase levels between the untreated group(SOD_U) and the MPTP disease induced and no treatment group(SOD_M). As both the sets were normal, a Student's T-test was used. The p-value obtained for the test was 0.0000 and hence the difference between the data sets was considered statistically significant.

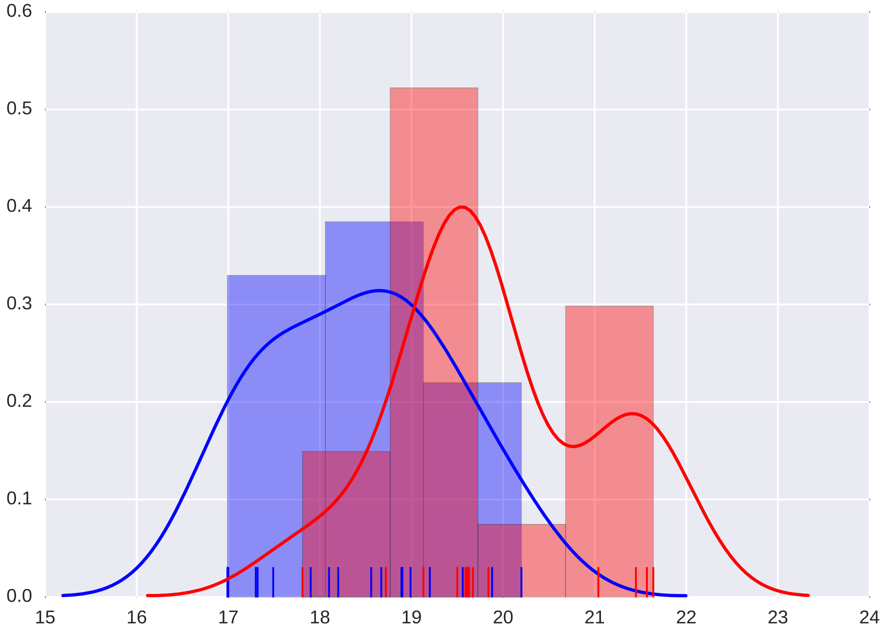

Figure 5: Significance Test Results for Superoxide dismutase levels between the untreated group(SOD_U) and the MPTP disease induced and treated with *Centella asiatica* group(SOD_MC). As one of the sets was non-normal, we used a Mann-Whitney U Test. The p-value obtained for the test was 0.0014 and hence the difference between the data sets was considered statistically significant.

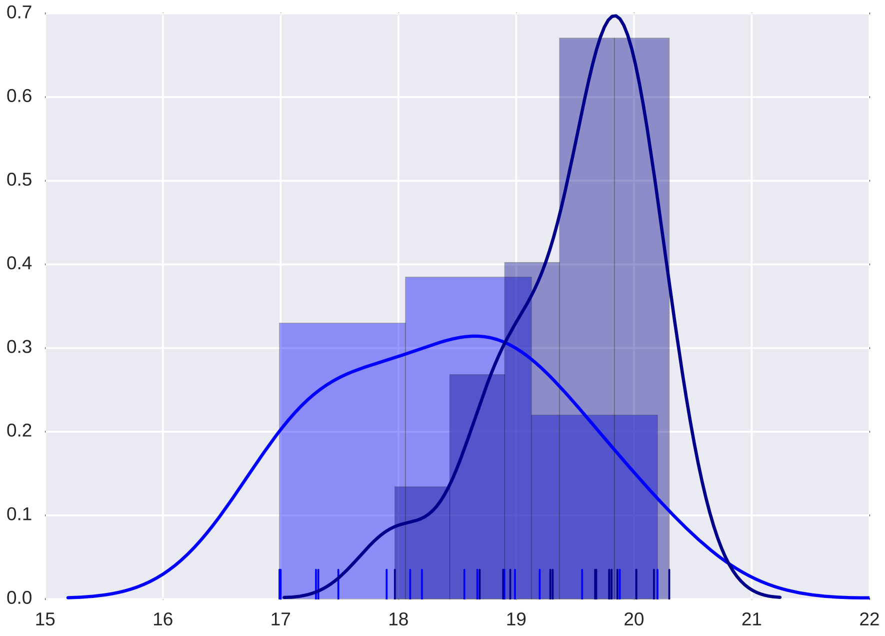

Figure 6: Significance Test Results for Superoxide dismutase levels between the untreated group(SOD_U) and the MPTP disease induced and treated with *Withania somnifera* group(SOD_MW). As both the sets were normal but with unequal variances, a Welch's T-test was used. The p-value obtained for the test was 0.0007 and hence the difference between the data sets was considered statistically significant.

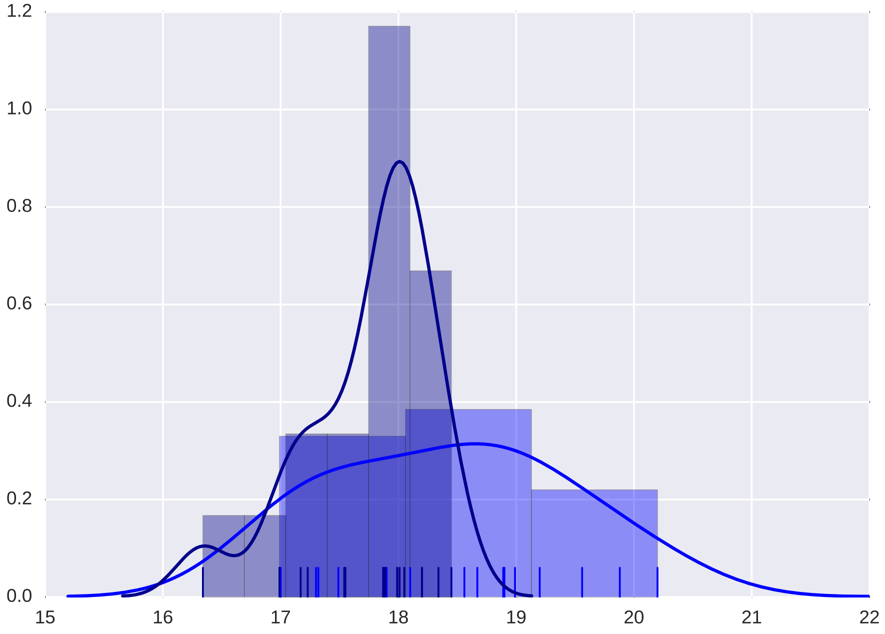

Figure 7: Significance Test Results for Superoxide dismutase levels between the untreated group(SOD_U) and the MPTP disease induced and treated with both *Withania somnifera* and *Centella asiatica* group(SOD_MWC). As both the sets were normal but with unequal variances, a Welch's T-test was used. The p-value obtained for the test was 0.0220 and hence the difference between the data sets was considered statistically significant.

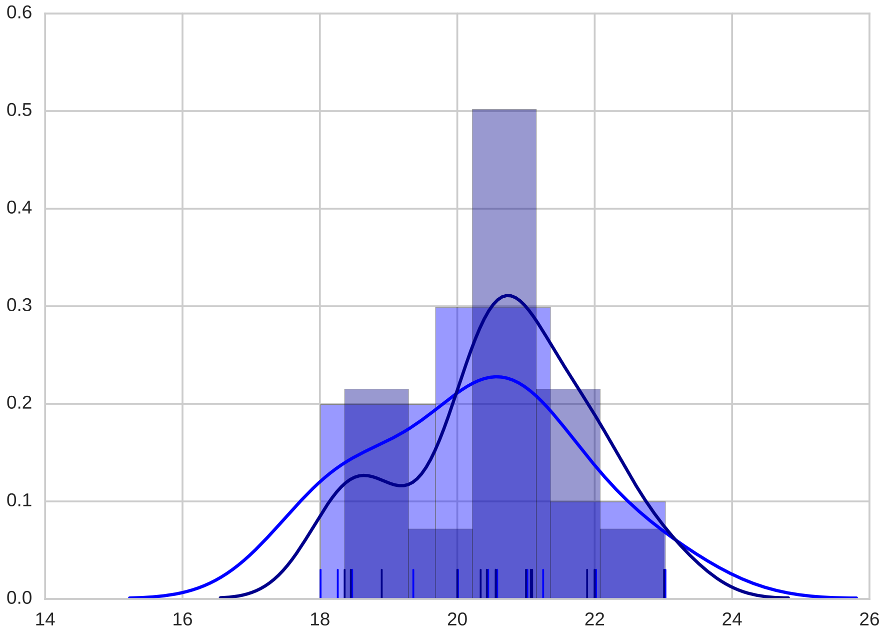

Figure 8: Significance Test Results for Superoxide dismutase levels between the group treated with *Centella asiatica*(SOD_C) and the group treated with *Withania somnifera*(SOD_W). As both the sets were normal, a Student's T-test was used. The p-value obtained for the test was 0.4852 and hence the difference between the data sets was considered statistically non-significant.

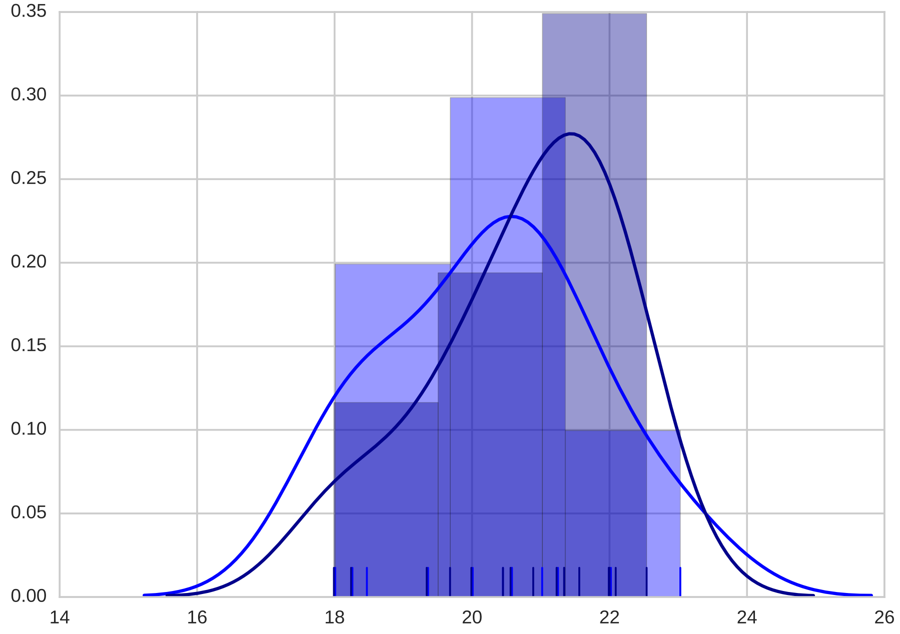

Figure 9: Significance Test Results for Superoxide dismutase levels between the group treated with *Centella asiatica*(SOD_C) and the group treated with both *Withania somnifera* and *Centella asiatica*(SOD_WC). As both the sets were normal, a Student's T-test was used. The p-value obtained for the test was 0.3372 and hence the difference between the data sets was considered statistically non-significant.

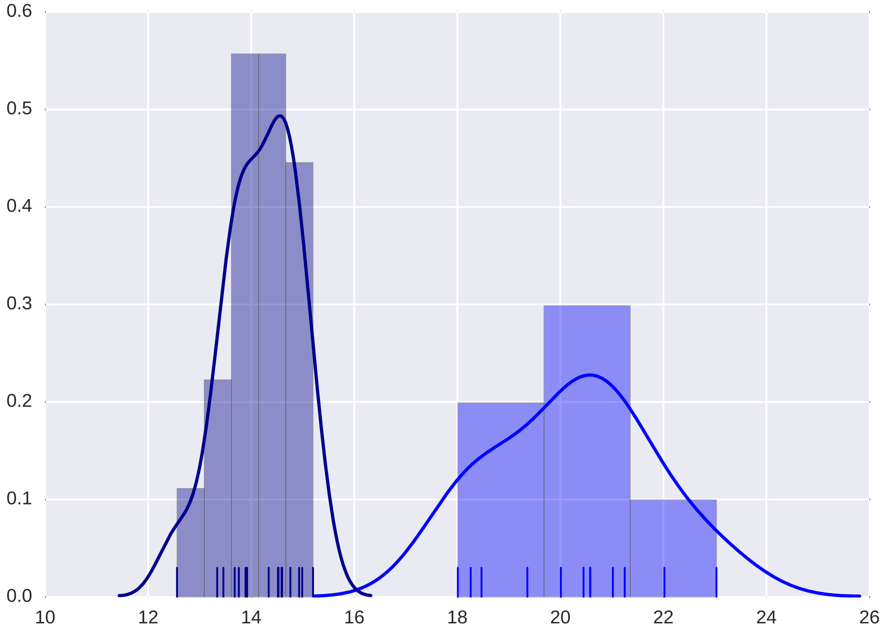

Figure 10: Significance Test Results for Superoxide dismutase levels between the group treated with *Centella asiatica*(SOD_C) and the MPTP disease induced and no treatment group(SOD_M). As both the sets were normal but with unequal variances, a Welch's T-test was used. The p-value obtained for the test was 0.0000 and hence the difference between the data sets was considered statistically significant.

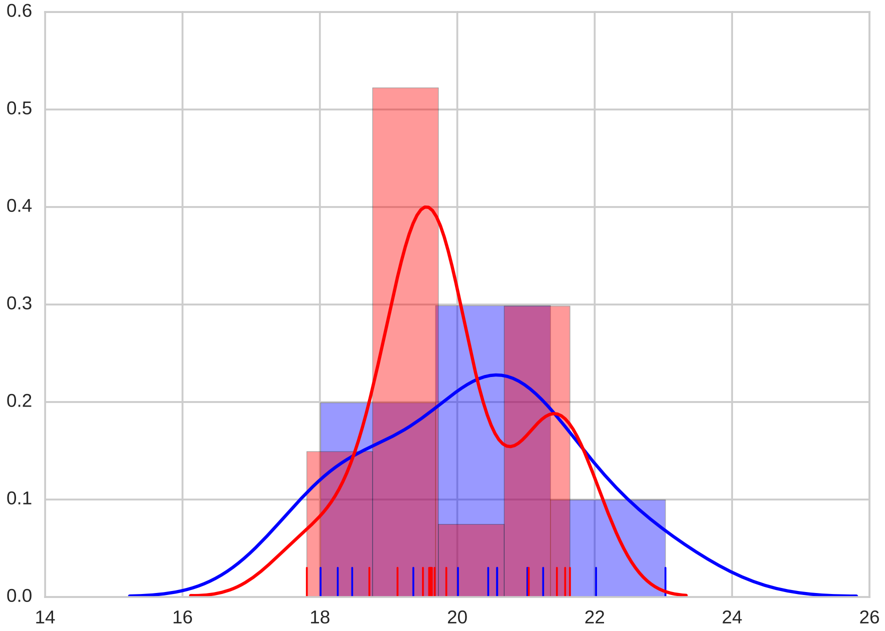

Figure 11: Significance Test Results for Superoxide dismutase levels between the group treated with *Centella asiatica*(SOD_C) and the MPTP disease induced and treated with *Centella asiatica* group(SOD_MC). As one of the sets was non-normal, we used a Mann-Whitney U Test. The p-value obtained for the test was 0.5890 and hence the difference between the data sets was considered statistically non-significant.

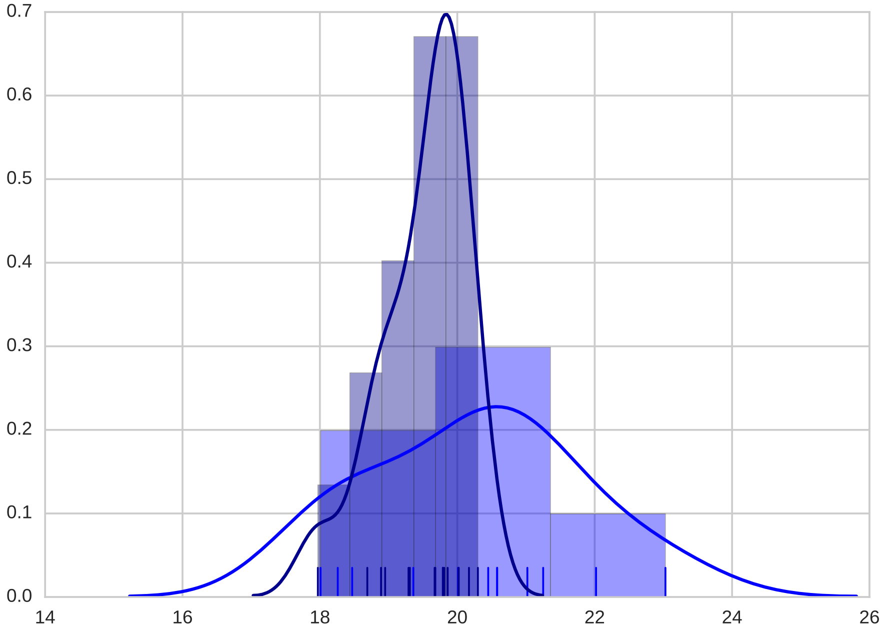

Figure 12: Significance Test Results for Superoxide dismutase levels between the group treated with *Centella asiatica*(SOD_C) and the MPTP disease induced and treated with *Withania somnifera* group(SOD_MW). As both the sets were normal but with unequal variances, a Welch's T-test was used. The p-value obtained for the test was 0.1365 and hence the difference between the data sets was considered statistically non-significant.

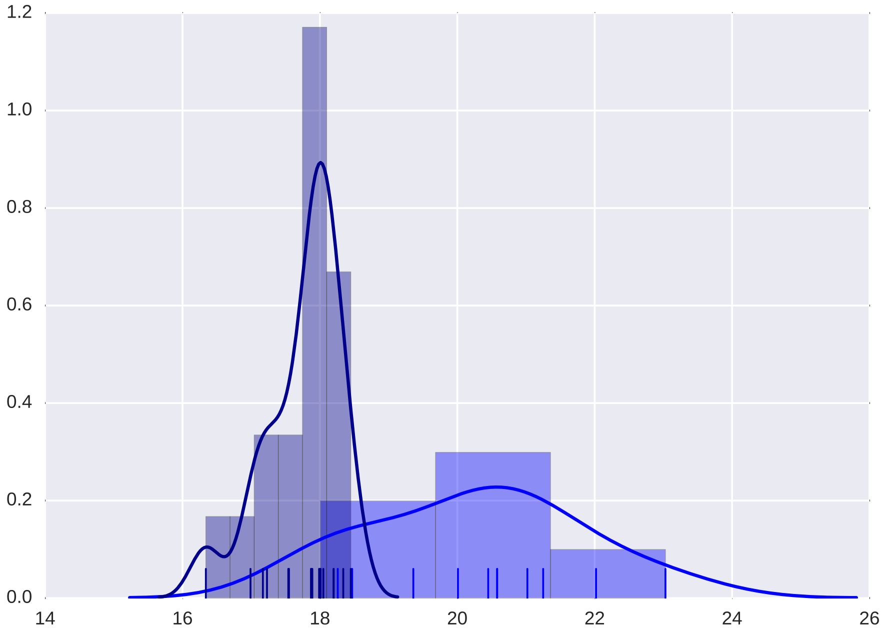

Figure 13: Significance Test Results for Superoxide dismutase levels between the group treated with *Centella asiatica*(SOD_C) and the MPTP disease induced and treated with both *Withania somnifera* and *Centella asiatica* group(SOD_MWC). As both the sets were normal but with unequal variances, a Welch's T-test was used. The p-value obtained for the test was 0.0001 and hence the difference between the data sets was considered statistically significant.

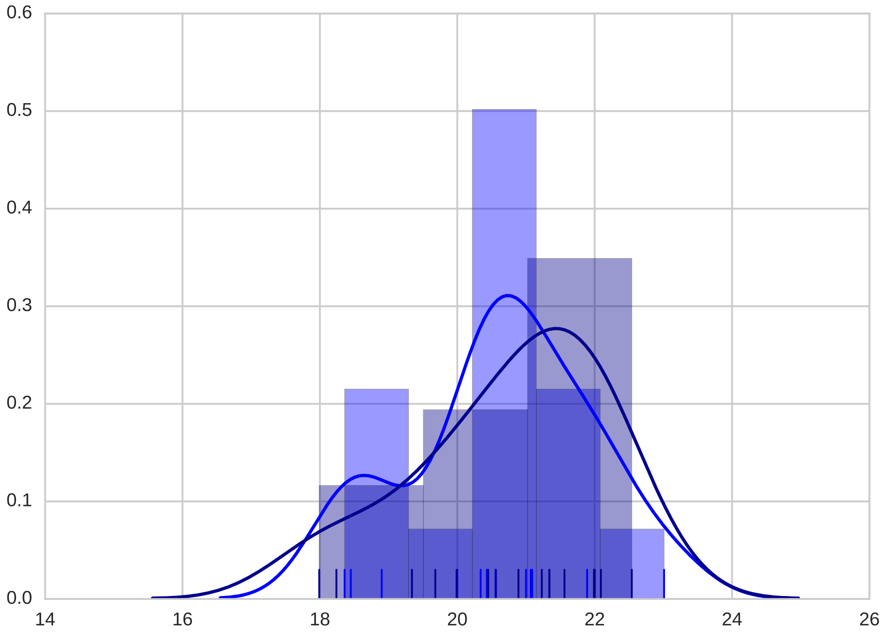

Figure 14: Significance Test Results for Superoxide dismutase levels between the group treated with *Withania somnifera*(SOD_W) and the group treated with both *Withania somnifera* and *Centella asiatica*(SOD_WC). As both the sets were normal, a Student's T-test was used. The p-value obtained for the test was 0.7804 and hence the difference between the data sets was considered statistically non-significant.

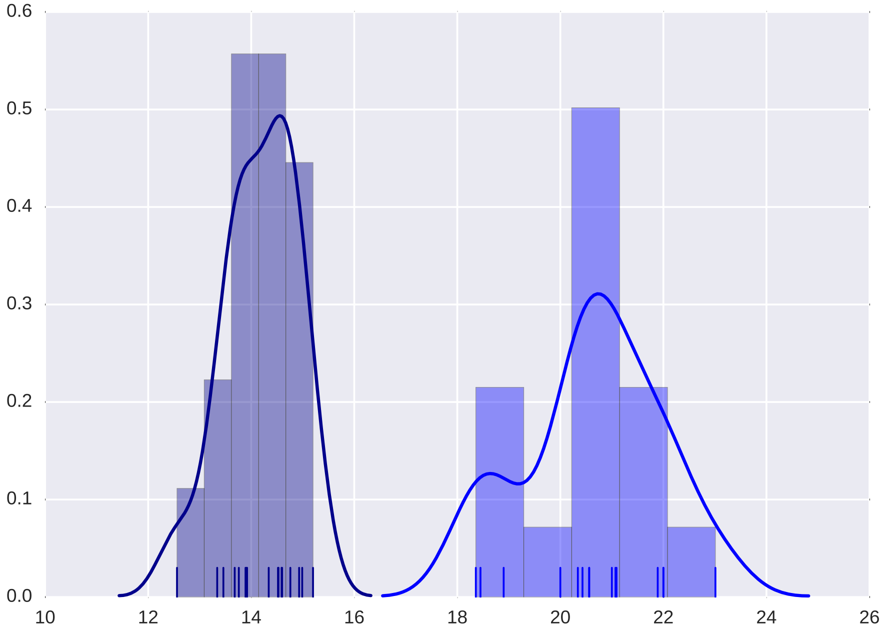

Figure 15: Significance Test Results for Superoxide dismutase levels between the group treated with *Withania somnifera*(SOD_W) and the MPTP disease induced and no treatment group(SOD_M). As both the sets were normal, a Student's T-test was used. The p-value obtained for the test was 0.0000 and hence the difference between the data sets was considered statistically significant.

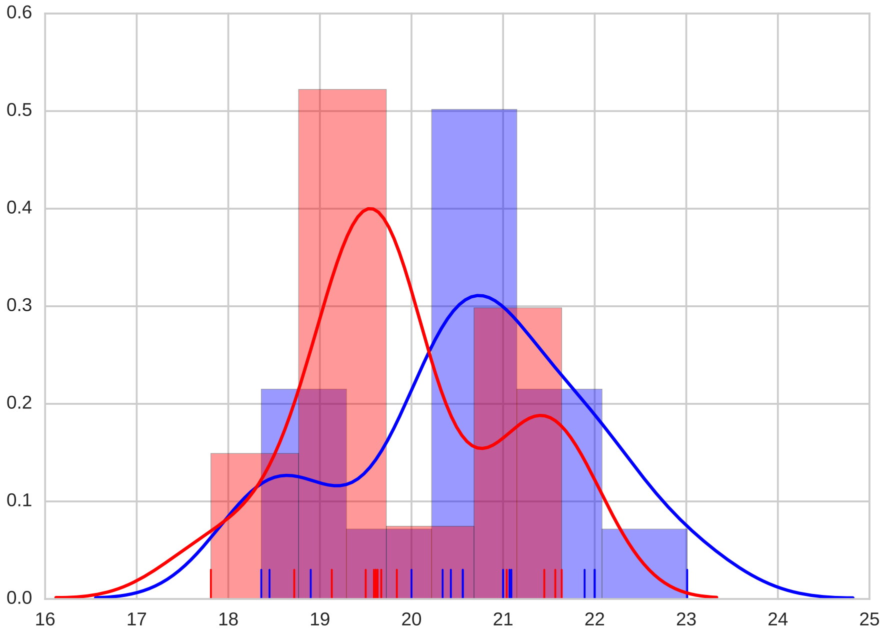

Figure 16: Significance Test Results for Superoxide dismutase levels between the group treated with *Withania somnifera*(SOD_W) and the MPTP disease induced and treated with *Centella asiatica* group(SOD_MC). As one of the sets was non-normal, we used a Mann-Whitney U Test. The p-value obtained for the test was 0.1110 and hence the difference between the data sets was considered statistically non-significant.

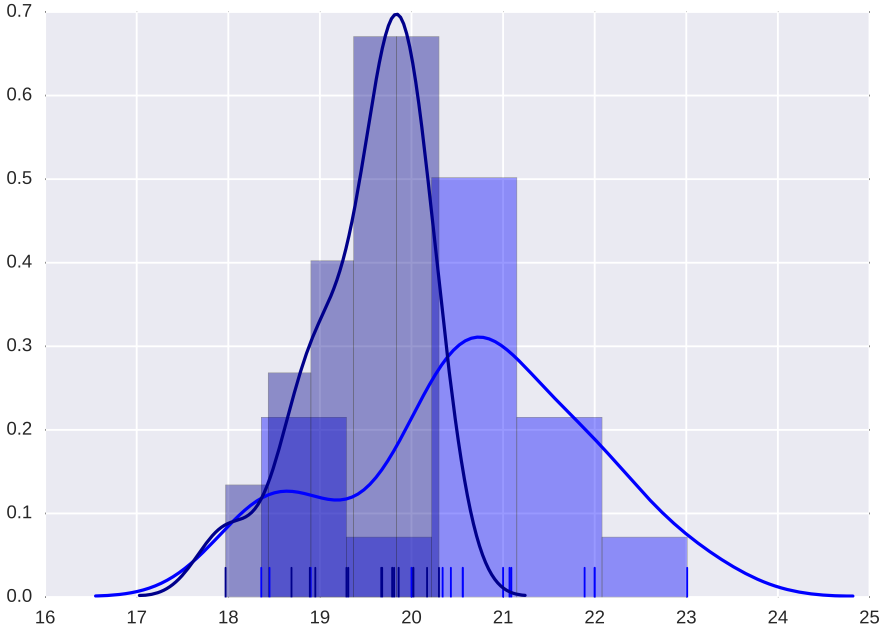

Figure 17: Significance Test Results for Superoxide dismutase levels between the group treated with *Withania somnifera*(SOD_W) and the MPTP disease induced and treated with *Withania somnifera* group(SOD_MW). As both the sets were normal but with unequal variances, a Welch's T-test was used. The p-value obtained for the test was 0.0074 and hence the difference between the data sets was considered statistically significant.

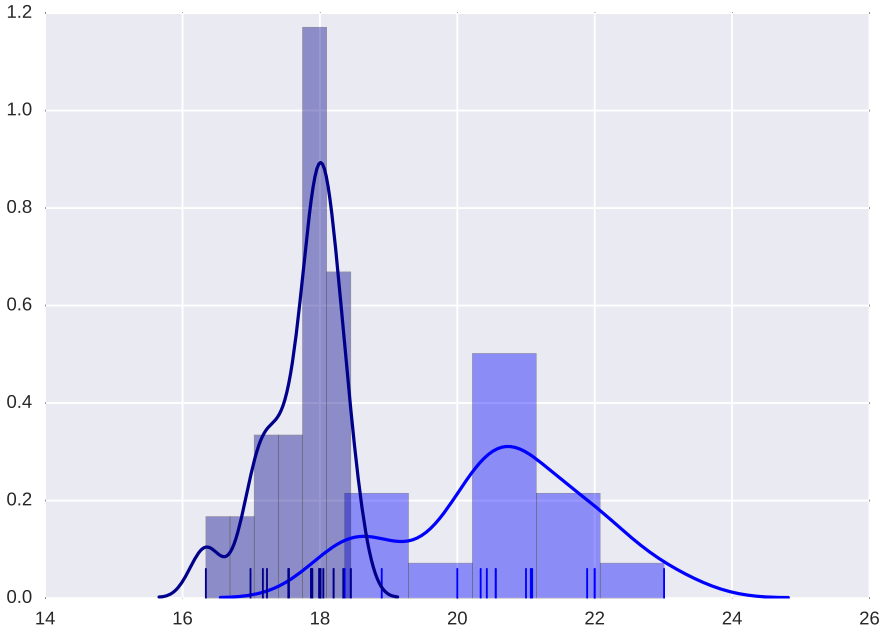

Figure 18: Significance Test Results for Superoxide dismutase levels between the group treated with *Withania somnifera*(SOD_W) and the MPTP disease induced and treated with both *Withania somnifera* and *Centella asiatica* group(SOD_MWC). As both the sets were normal but with unequal variances, a Welch's T-test was used. The p-value obtained for the test was 0.0000 and hence the difference between the data sets was considered statistically significant.

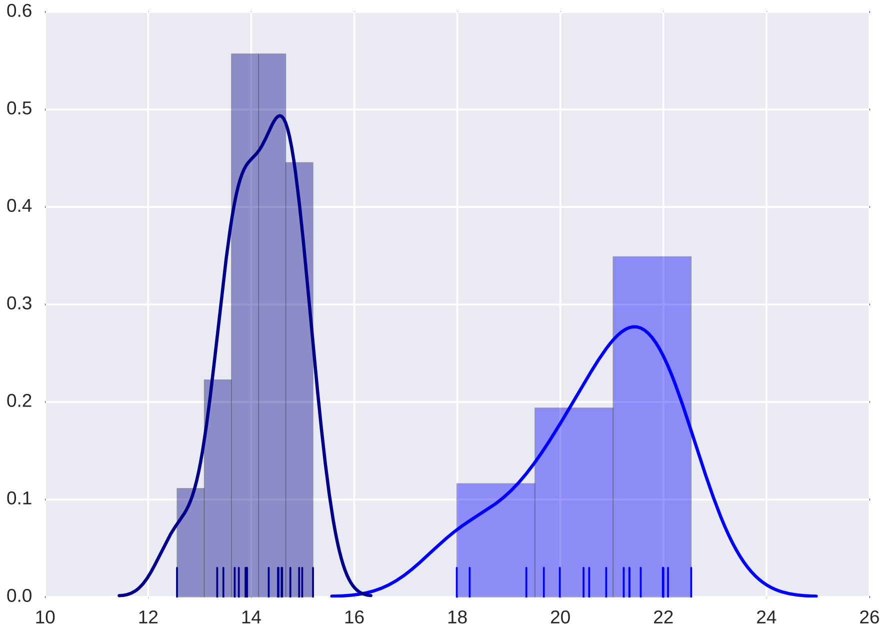

Figure 19: Significance Test Results for Superoxide dismutase levels between the group treated with both *Withania somnifera* and *Centella asiatica*(SOD_WC) and the MPTP disease induced and no treatment group(SOD_M). As both the sets were normal, a Student's T-test was used. The p-value obtained for the test was 0.0000 and hence the difference between the data sets was considered statistically significant.

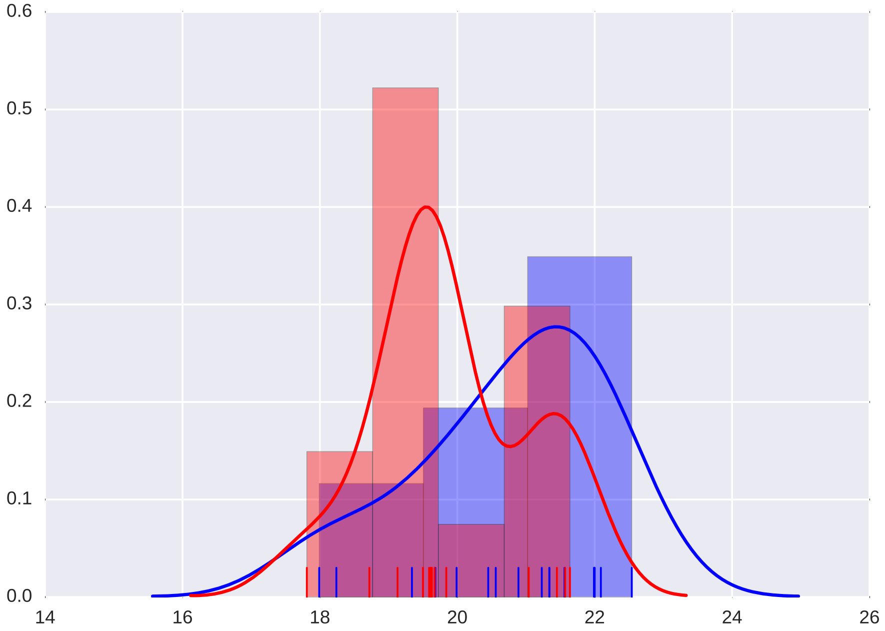

Figure 20: Significance Test Results for Superoxide dismutase levels between the group treated with both *Withania somnifera* and *Centella asiatica*(SOD_WC) and the MPTP disease induced and treated with *Centella asiatica* group(SOD_MC). As one of the sets was non-normal, we used a Mann-Whitney U Test. The p-value obtained for the test was 0.0494 and hence the difference between the data sets was considered statistically significant.

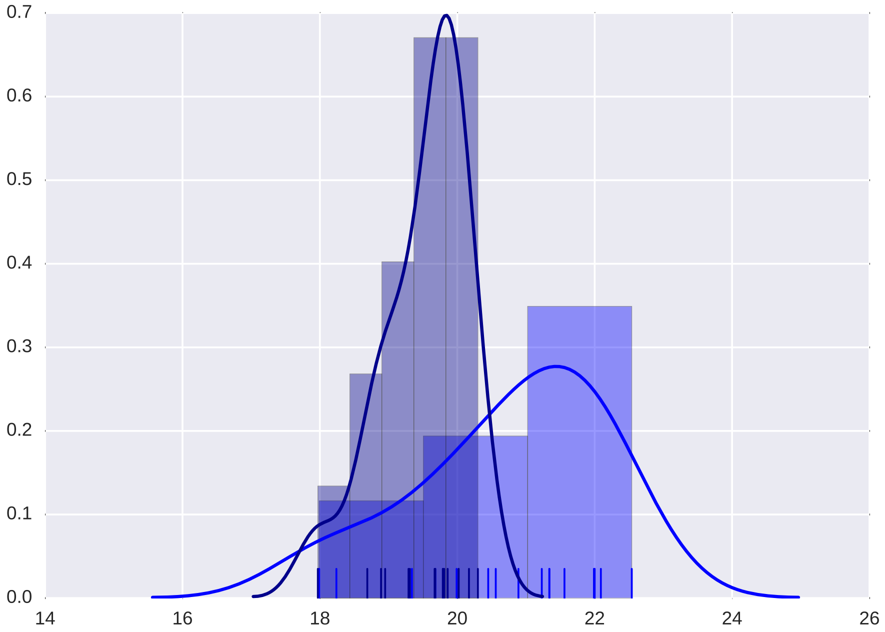

Figure 21: Significance Test Results for Superoxide dismutase levels between the group treated with both *Withania somnifera* and *Centella asiatica*(SOD_WC) and the MPTP disease induced and treated with *Withania somnifera* group(SOD_MW). As both the sets were normal but with unequal variances, a Welch's T-test was used. The p-value obtained for the test was 0.0020 and hence the difference between the data sets was considered statistically significant.

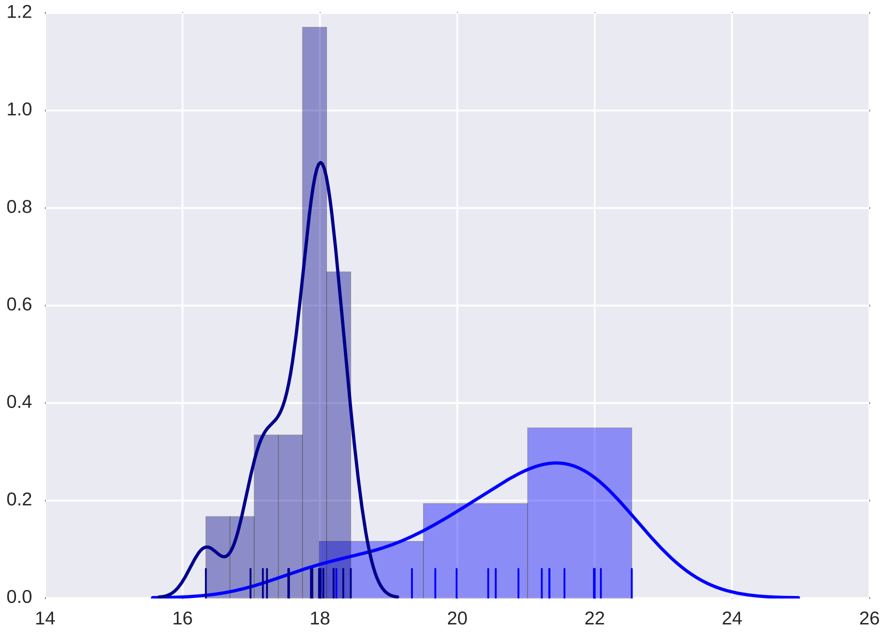

Figure 22: Significance Test Results for Superoxide dismutase levels between the group treated with both *Withania somnifera* and *Centella asiatica*(SOD_WC) and the MPTP disease induced and treated with both *Withania somnifera* and *Centella asiatica* group(SOD_MWC). As both the sets were normal but with unequal variances, a Welch's T-test was used. The p-value obtained for the test was 0.0000 and hence the difference between the data sets was considered statistically significant.

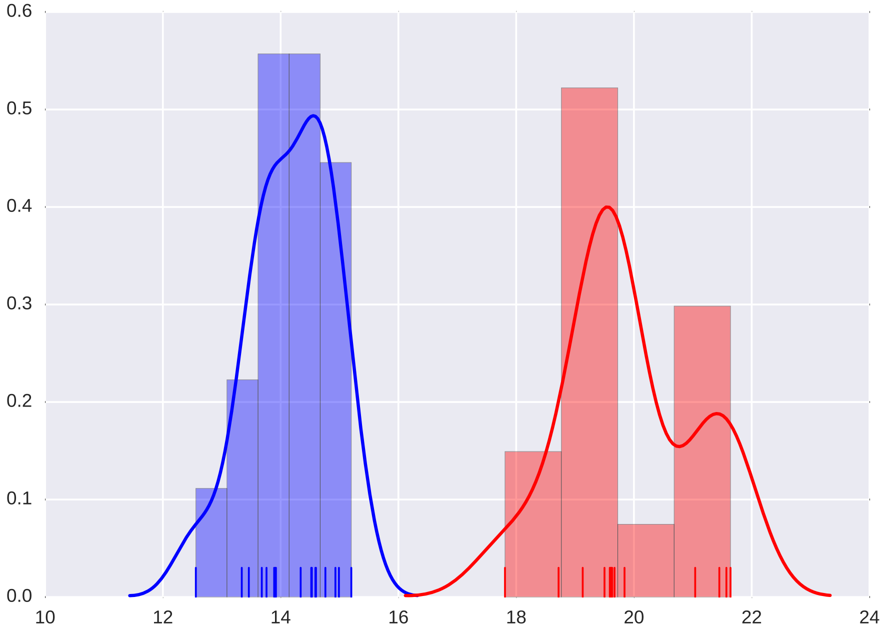

Figure 23: Significance Test Results for Superoxide dismutase levels between the MPTP disease induced and no treatment group(SOD_M) and the MPTP disease induced and treated with *Centella asiatica* group(SOD_MC). As one of the sets was non-normal, we used a Mann-Whitney U Test. The p-value obtained for the test was 0.0000 and hence the difference between the data sets was considered statistically significant.

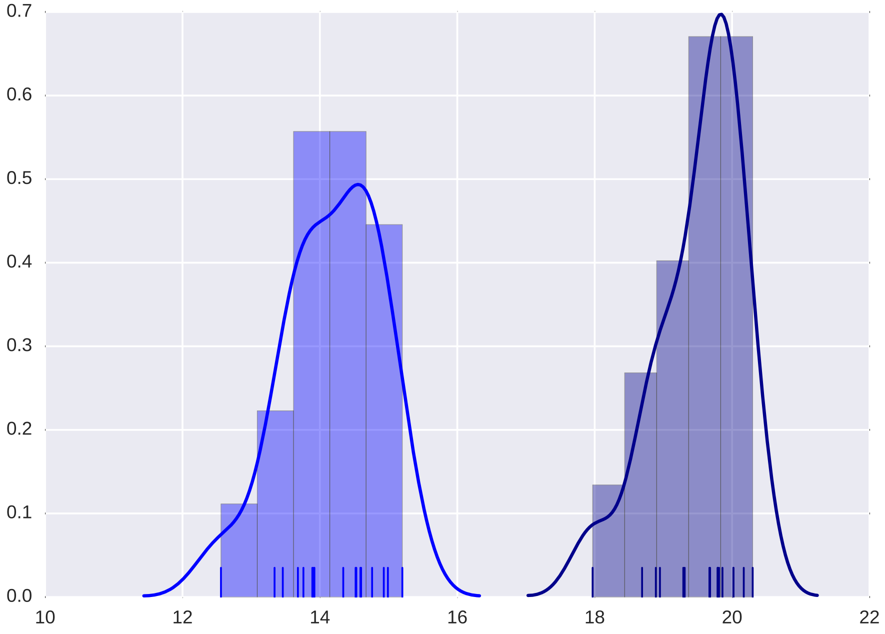

Figure 24: Significance Test Results for Superoxide dismutase levels between the MPTP disease induced and no treatment group(SOD_M) and the MPTP disease induced and treated with *Withania somnifera* group(SOD_MW). As both the sets were normal, a Student's T-test was used. The p-value obtained for the test was 0.0000 and hence the difference between the data sets was considered statistically significant.

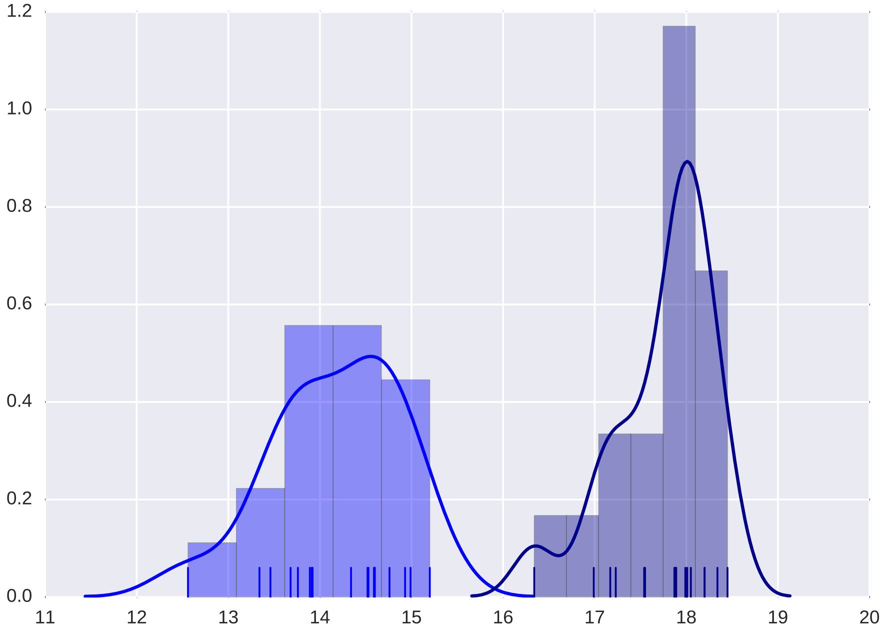

Figure 25: Significance Test Results for Superoxide dismutase levels between the MPTP disease induced and no treatment group(SOD_M) and the MPTP disease induced and treated with both *Withania somnifera* and *Centella asiatica* group(SOD_MWC). As both the sets were normal, a Student's T-test was used. The p-value obtained for the test was 0.0000 and hence the difference between the data sets was considered statistically significant.

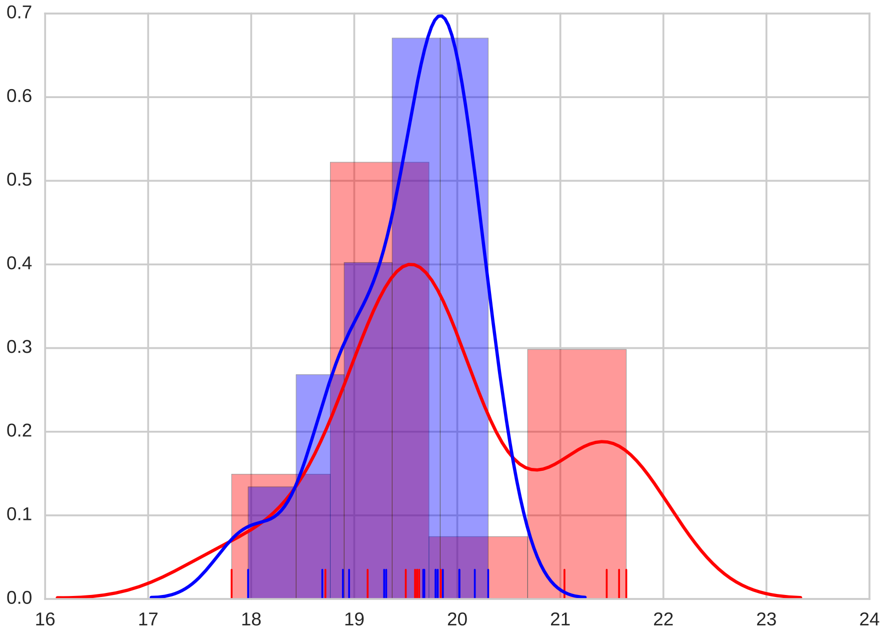

Figure 26: Significance Test Results for Superoxide dismutase levels between the MPTP disease induced and treated with *Centella asiatica* group(SOD_MC) and the MPTP disease induced and treated with *Withania somnifera* group(SOD_MW). As one of the sets was non-normal, we used a Mann-Whitney U Test. The p-value obtained for the test was 0.8353 and hence the difference between the data sets was considered statistically non-significant.

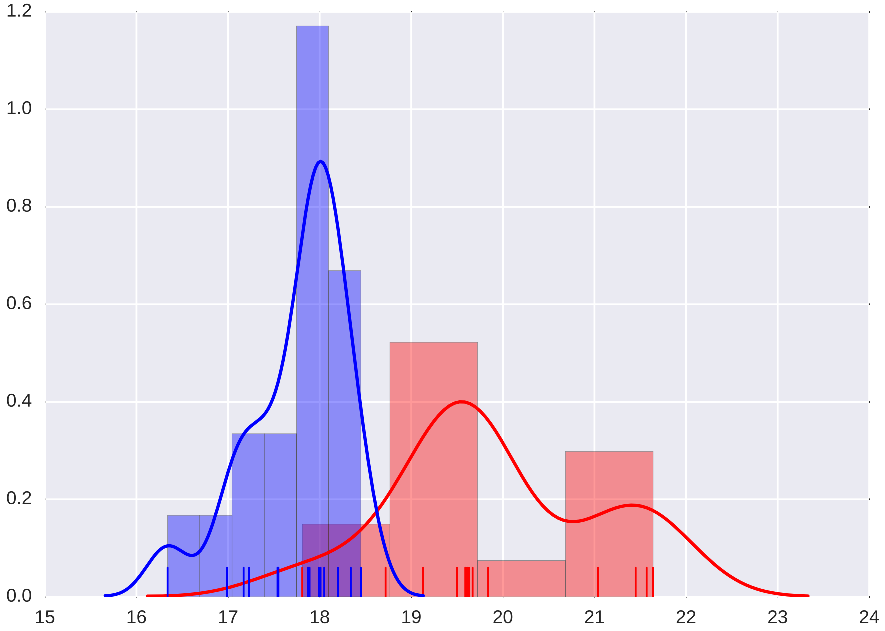

Figure 27: Significance Test Results for Superoxide dismutase levels between the MPTP disease induced and treated with *Centella asiatica* group(SOD_MC) and the MPTP disease induced and treated with both *Withania somnifera* and *Centella asiatica* group(SOD_MWC). As one of the sets was non-normal, we used a Mann-Whitney U Test. The p-value obtained for the test was 0.0000 and hence the difference between the data sets was considered statistically significant.

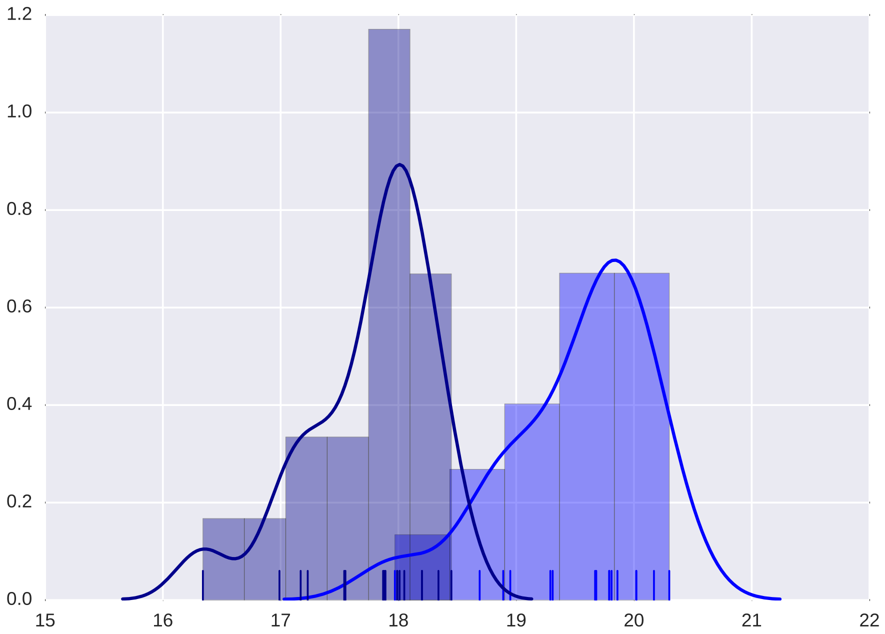

Figure 28: Significance Test Results for Superoxide dismutase levels between the MPTP disease induced and treated with *Withania somnifera* group(SOD_MW) and the MPTP disease induced and treated with both *Withania somnifera* and *Centella asiatica* group(SOD_MWC). As both the sets were normal, a Student's T-test was used. The p-value obtained for the test was 0.0000 and hence the difference between the data sets was considered statistically significant.

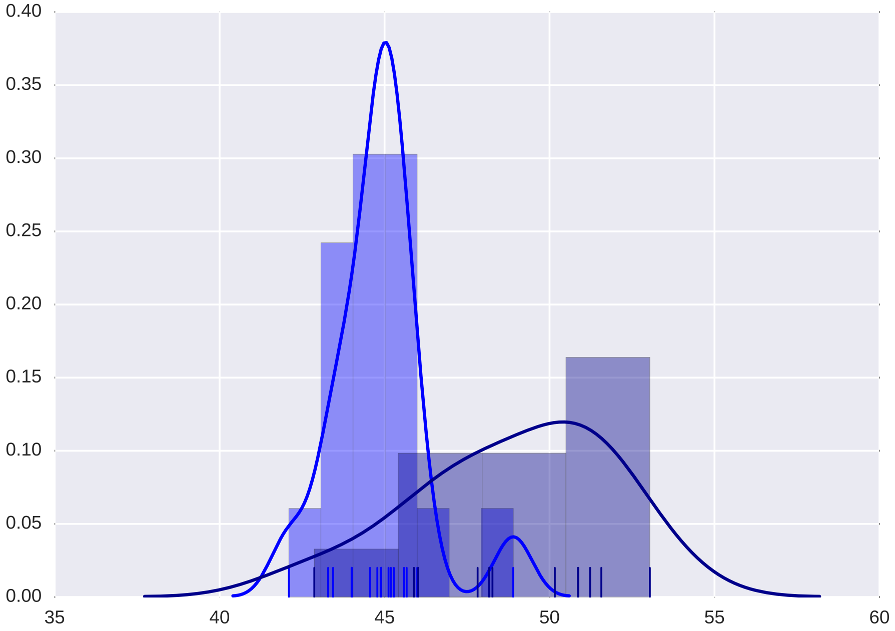

Figure 29: Significance Test Results for Catalase levels between the untreated group(CAT_U) and the group treated with *Centella asiatica*(CAT_C). As both the sets were normal but with unequal variances, a Welch's T-test was used. The p-value obtained for the test was 0.0005 and hence the difference between the data sets was considered statistically significant.

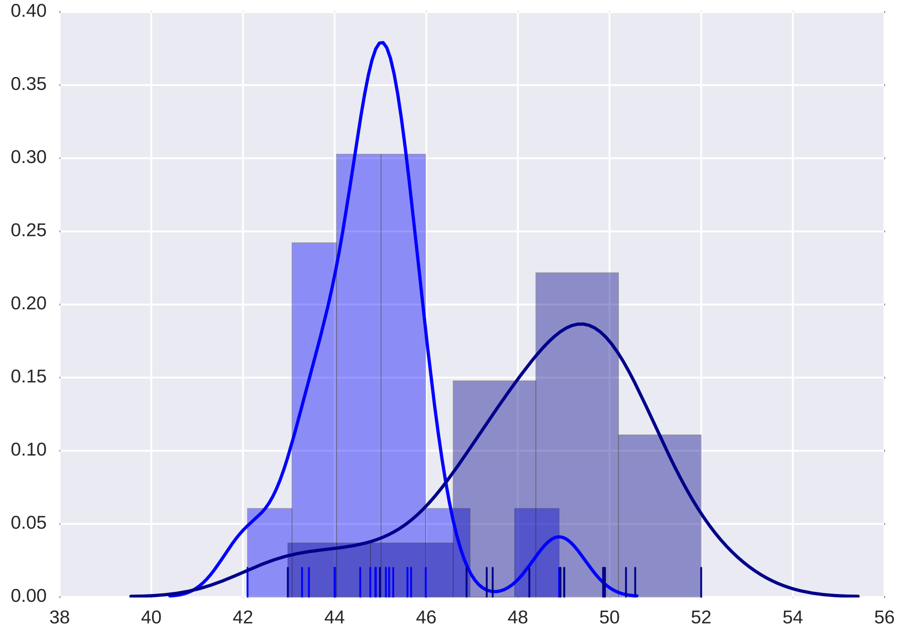

Figure 30: Significance Test Results for Catalase levels between the untreated group(CAT_U) and the group treated with *Withania somnifera*(CAT_W). As both the sets were normal, a Student's T-test was used. The p-value obtained for the test was 0.0000 and hence the difference between the data sets was considered statistically significant.

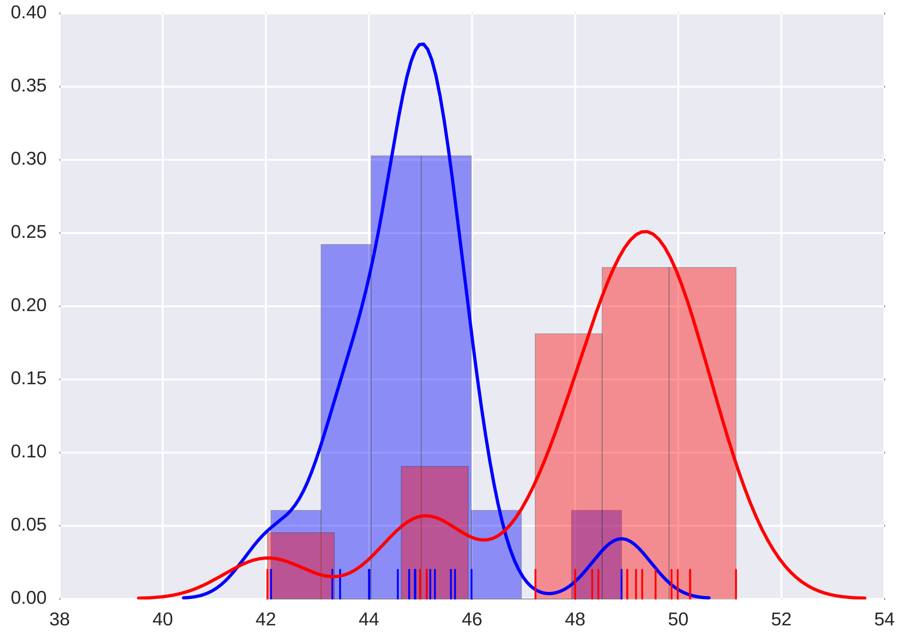

Figure 31: Significance Test Results for Catalase levels between the untreated group(CAT_U) and the group treated with both *Withania somnifera* and *Centella asiatica*(CAT_WC). As one of the sets was non-normal, we used a Mann-Whitney U Test. The p-value obtained for the test was 0.0002 and hence the difference between the data sets was considered statistically significant.

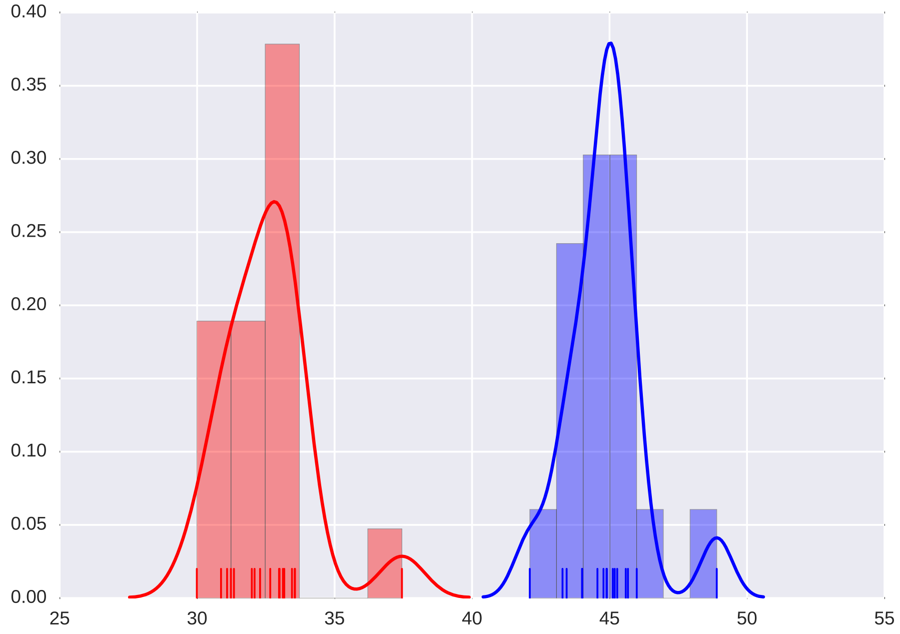

Figure 32: Significance Test Results for Catalase levels between the untreated group(CAT_U) and the MPTP disease induced and no treatment group(CAT_M). As one of the sets was non-normal, we used a Mann-Whitney U Test. The p-value obtained for the test was 0.0000 and hence the difference between the data sets was considered statistically significant.

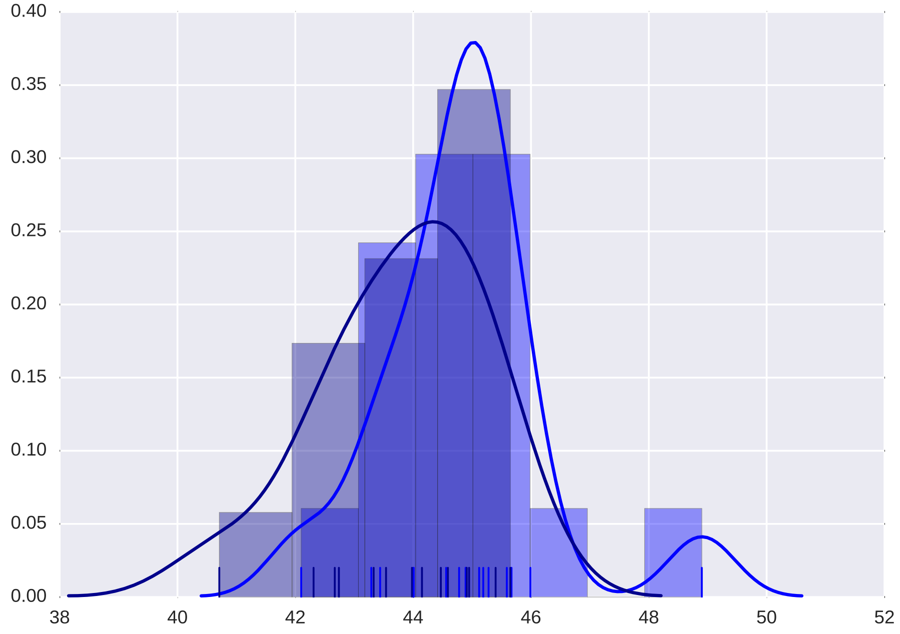

Figure 33: Significance Test Results for Catalase levels between the untreated group(CAT_U) and the MPTP disease induced and treated with *Centella asiatica* group(CAT_MC). As both the sets were normal, a Student's T-test was used. The p-value obtained for the test was 0.0479 and hence the difference between the data sets was considered statistically significant.

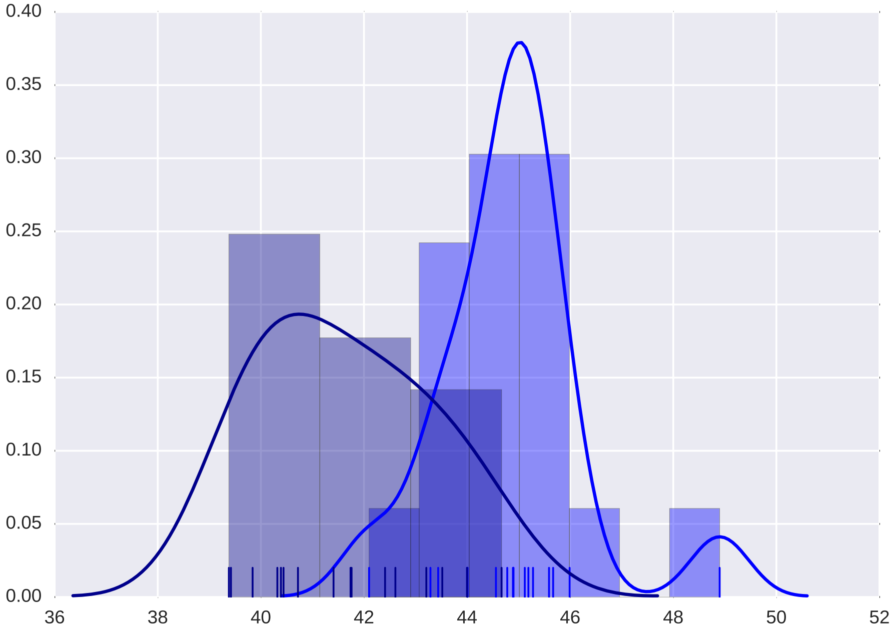

Figure 34: Significance Test Results for Catalase levels between the untreated group(CAT_U) and the MPTP disease induced and treated with *Withania somnifera* group(CAT_MW). As both the sets were normal, a Student's T-test was used. The p-value obtained for the test was 0.0000 and hence the difference between the data sets was considered statistically significant.

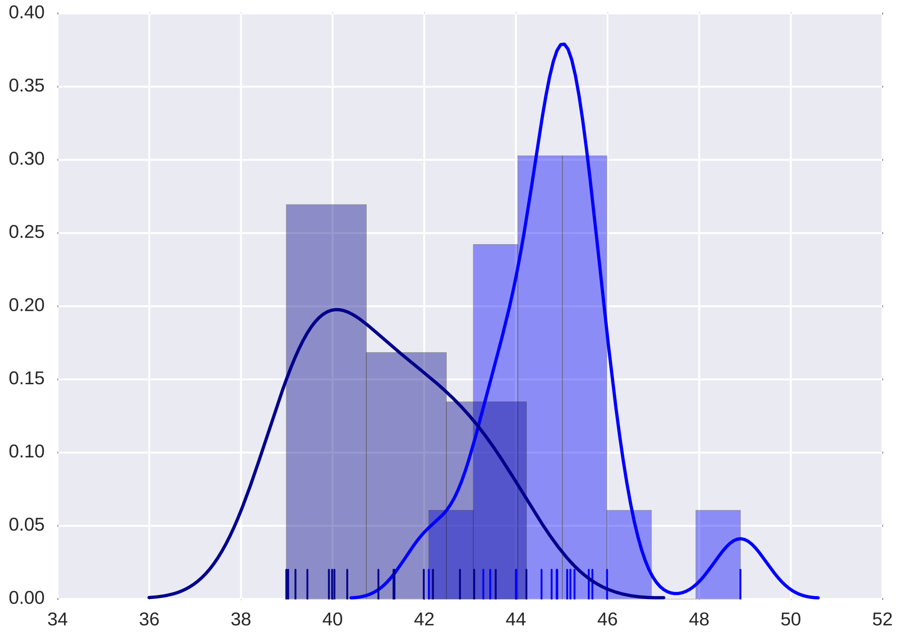

Figure 35: Significance Test Results for Catalase levels between the untreated group(CAT_U) and the MPTP disease induced and treated with both *Withania somnifera* and *Centella asiatica* group(CAT_MWC). As both the sets were normal, a Student's T-test was used. The p-value obtained for the test was 0.0000 and hence the difference between the data sets was considered statistically significant.

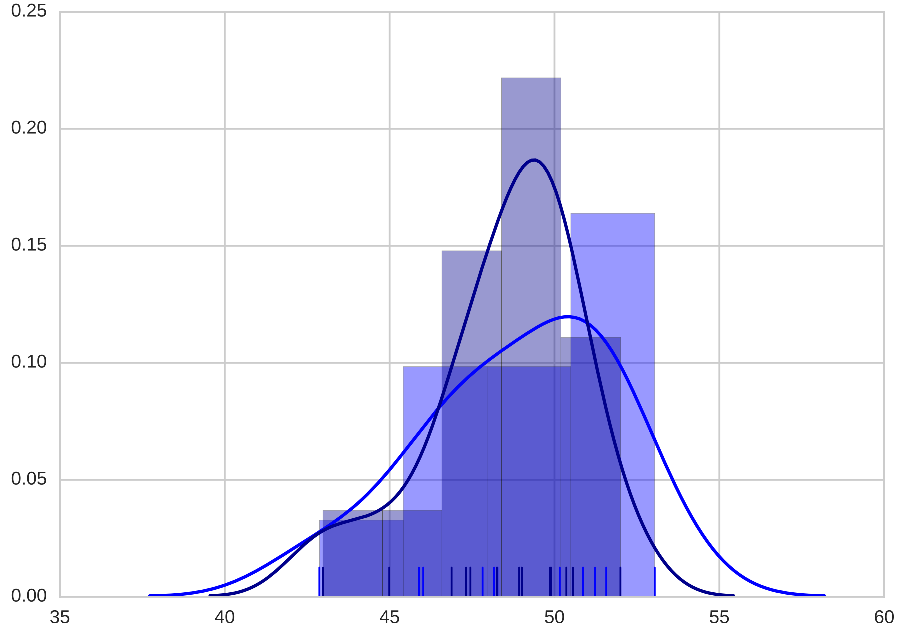

Figure 36: Significance Test Results for Catalase levels between the group treated with *Centella asiatica*(CAT_C) and the group treated with *Withania somnifera*(CAT_W). As both the sets were normal, a Student's T-test was used. The p-value obtained for the test was 0.6902 and hence the difference between the data sets was considered statistically non-significant.

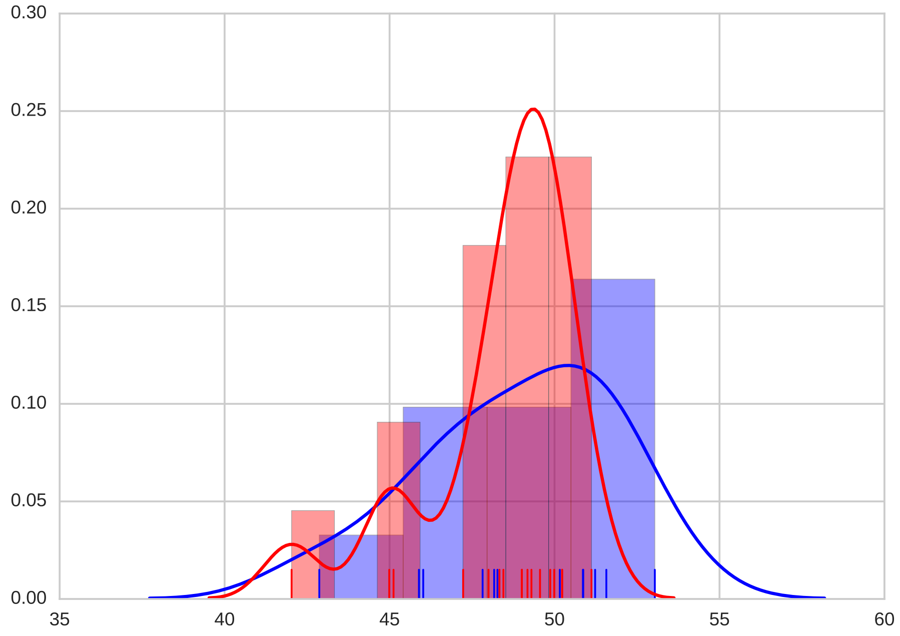

Figure 37: Significance Test Results for Catalase levels between the group treated with *Centella asiatica*(CAT_C) and the group treated with both *Withania somnifera* and *Centella asiatica*(CAT_WC). As one of the sets was non-normal, we used a Mann-Whitney U Test. The p-value obtained for the test was 0.4924 and hence the difference between the data sets was considered statistically non-significant.

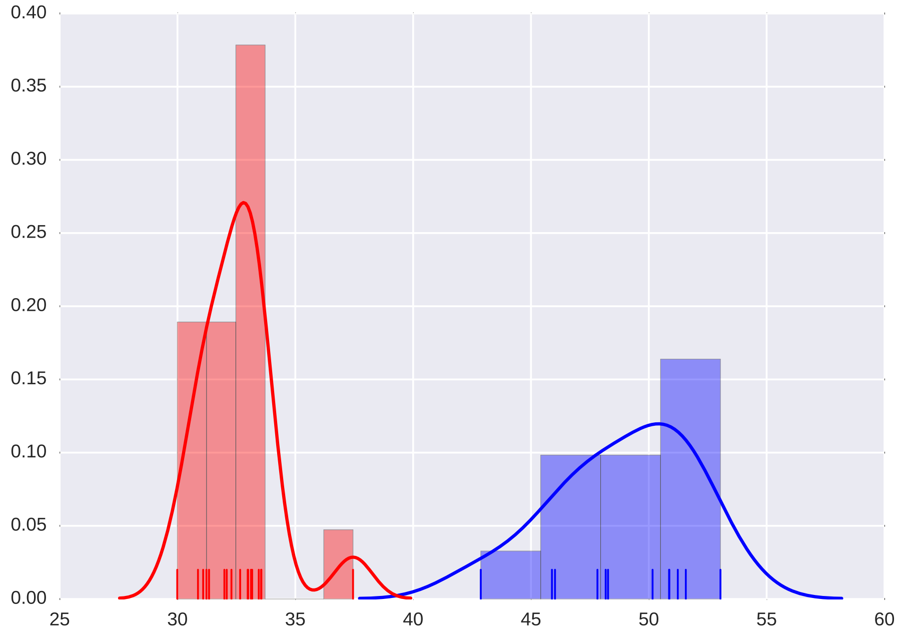

Figure 38: Significance Test Results for Catalase levels between the group treated with *Centella asiatica*(CAT_C) and the MPTP disease induced and no treatment group(CAT_M). As one of the sets was non-normal but the variances were unequal, we used a Welch's T Test with ranked data. The p-value obtained for the test was 0.0000 and hence the difference between the data sets was considered statistically significant.

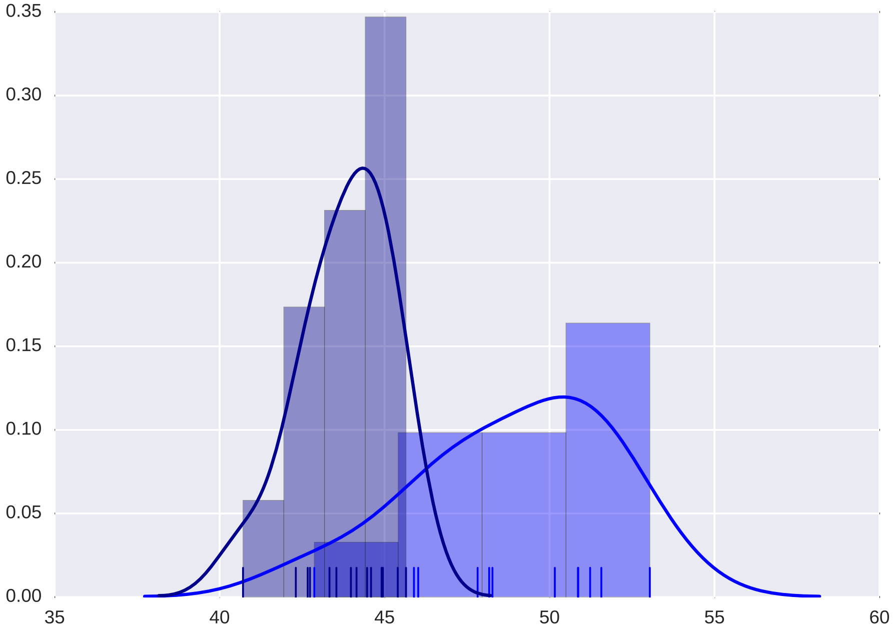

Figure 39: Significance Test Results for Catalase levels between the group treated with *Centella asiatica*(CAT_C) and the MPTP disease induced and treated with *Centella asiatica* group(CAT_MC). As both the sets were normal but with unequal variances, a Welch's T-test was used. The p-value obtained for the test was 0.0001 and hence the difference between the data sets was considered statistically significant.

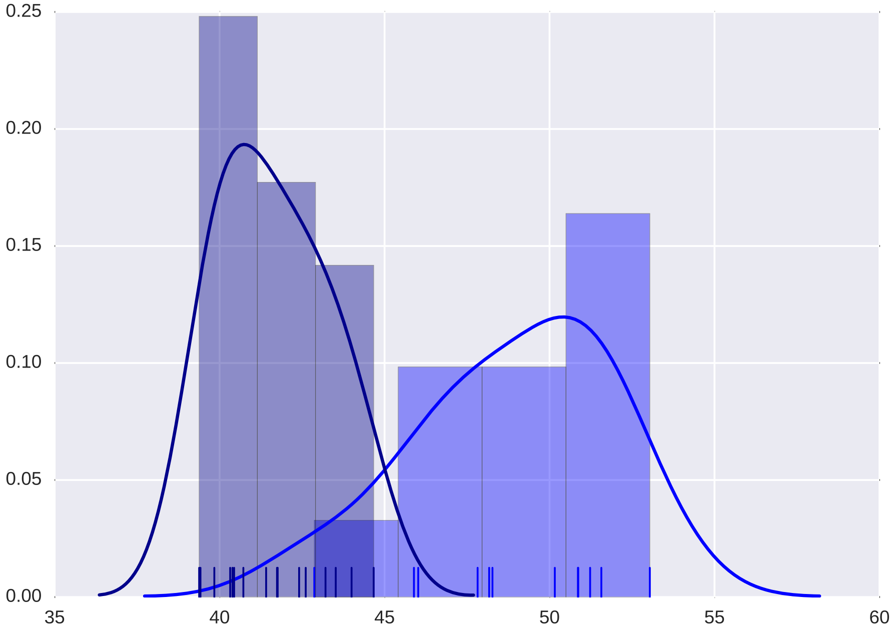

Figure 40: Significance Test Results for Catalase levels between the group treated with *Centella asiatica*(CAT_C) and the MPTP disease induced and treated with *Withania somnifera* group(CAT_MW). As both the sets were normal but with unequal variances, a Welch's T-test was used. The p-value obtained for the test was 0.0000 and hence the difference between the data sets was considered statistically significant.

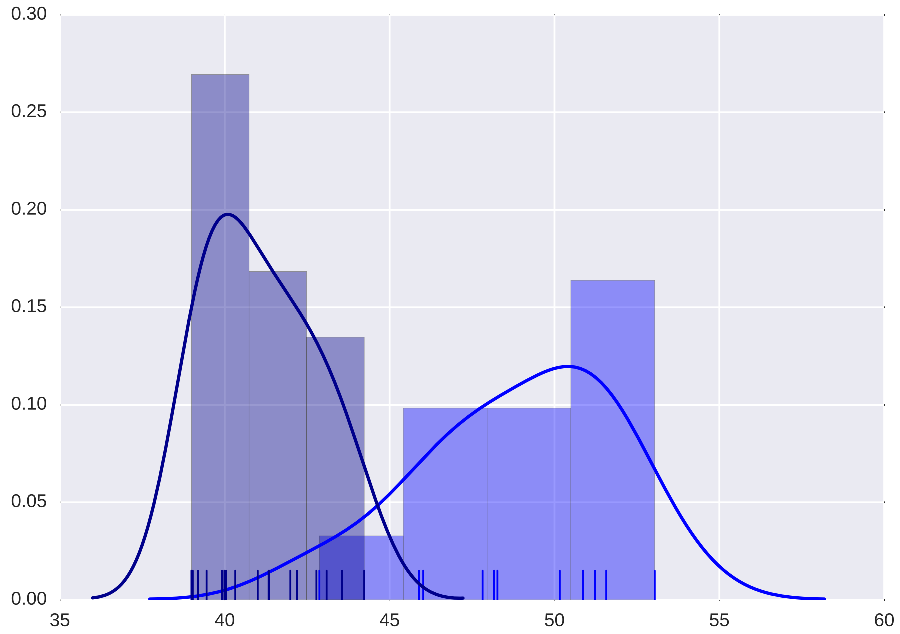

Figure 41: Significance Test Results for Catalase levels between the group treated with *Centella asiatica*(CAT_C) and the MPTP disease induced and treated with both *Withania somnifera* and *Centella asiatica* group(CAT_MWC). As both the sets were normal but with unequal variances, a Welch's T-test was used. The p-value obtained for the test was 0.0000 and hence the difference between the data sets was considered statistically significant.

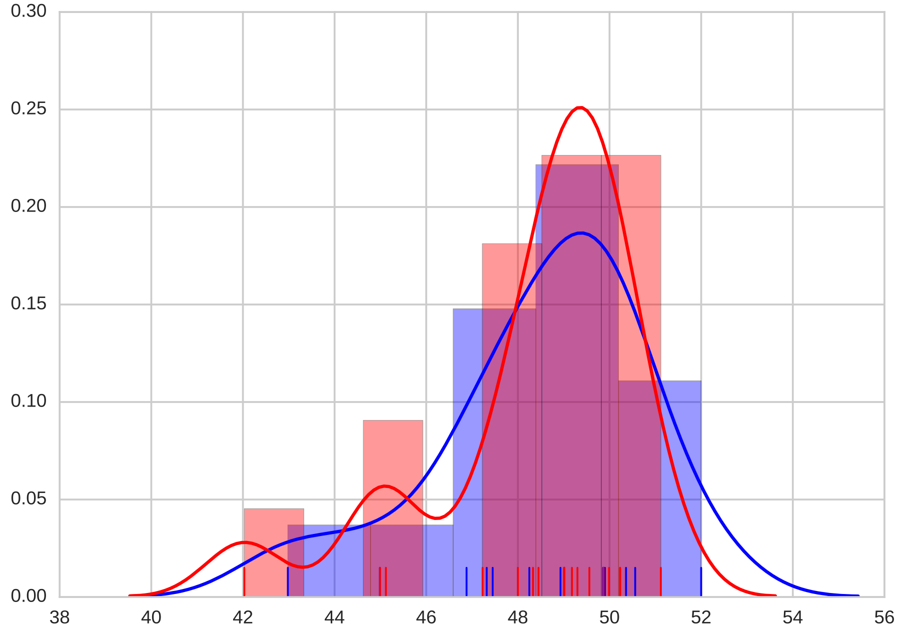

Figure 42: Significance Test Results for Catalase levels between the group treated with *Withania somnifera*(CAT_W) and the group treated with both *Withania somnifera* and *Centella asiatica*(CAT_WC). As one of the sets was non-normal, we used a Mann-Whitney U Test. The p-value obtained for the test was 1.0000 and hence the difference between the data sets was considered statistically non-significant.

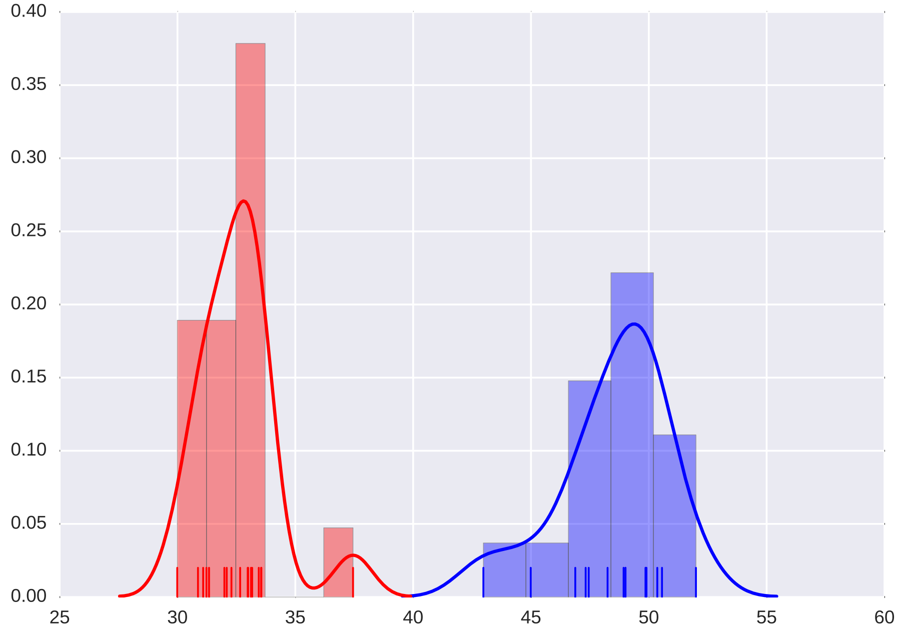

Figure 43: Significance Test Results for Catalase levels between the group treated with *Withania somnifera*(CAT_W) and the MPTP disease induced and no treatment group(CAT_M). As one of the sets was non-normal, we used a Mann-Whitney U Test. The p-value obtained for the test was 0.0000 and hence the difference between the data sets was considered statistically significant.

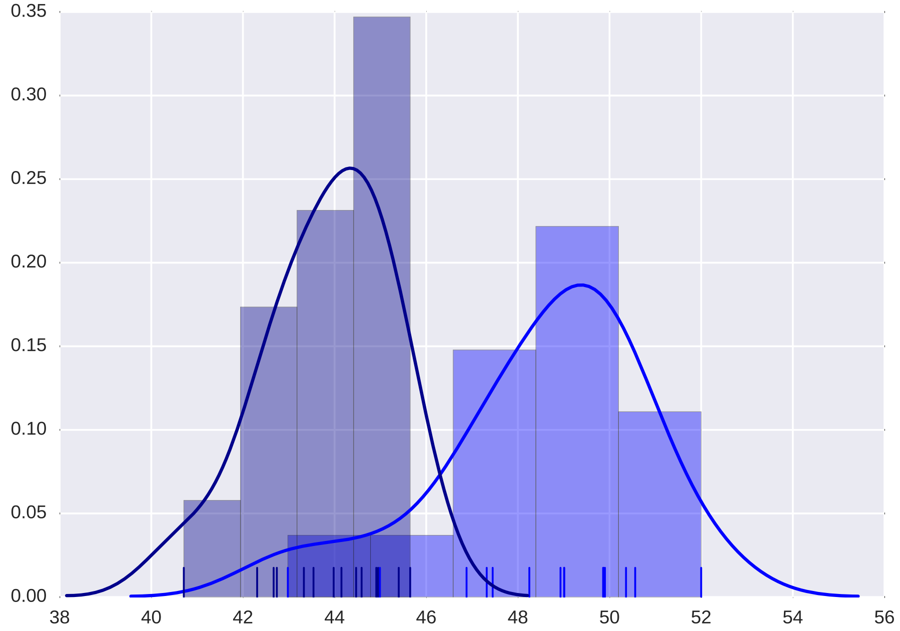

Figure 44: Significance Test Results for Catalase levels between the group treated with *Withania somnifera*(CAT_W) and the MPTP disease induced and treated with *Centella asiatica* group(CAT_MC). As both the sets were normal, a Student's T-test was used. The p-value obtained for the test was 0.0000 and hence the difference between the data sets was considered statistically significant.

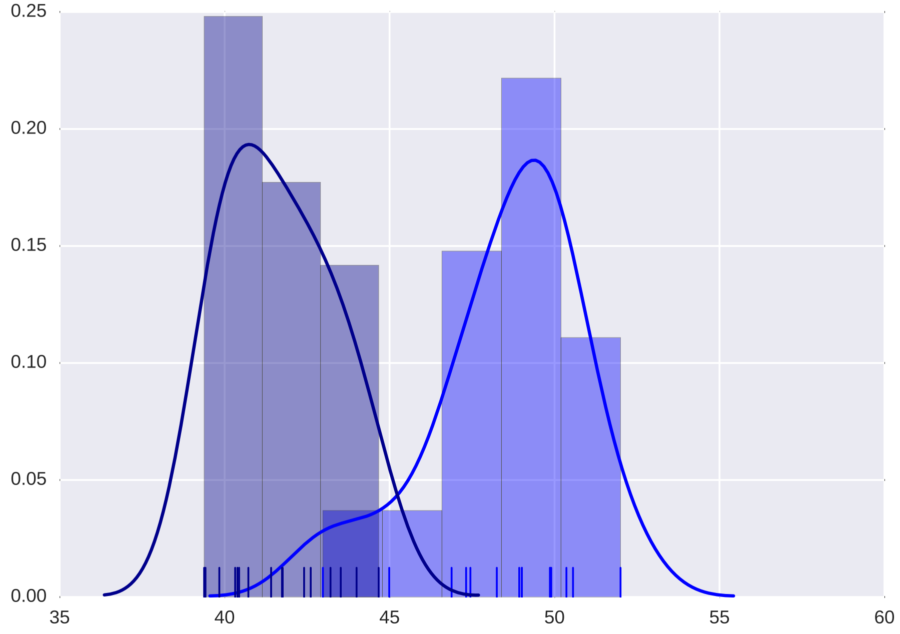

Figure 45: Significance Test Results for Catalase levels between the group treated with *Withania somnifera*(CAT_W) and the MPTP disease induced and treated with *Withania somnifera* group(CAT_MW). As both the sets were normal, a Student's T-test was used. The p-value obtained for the test was 0.0000 and hence the difference between the data sets was considered statistically significant.

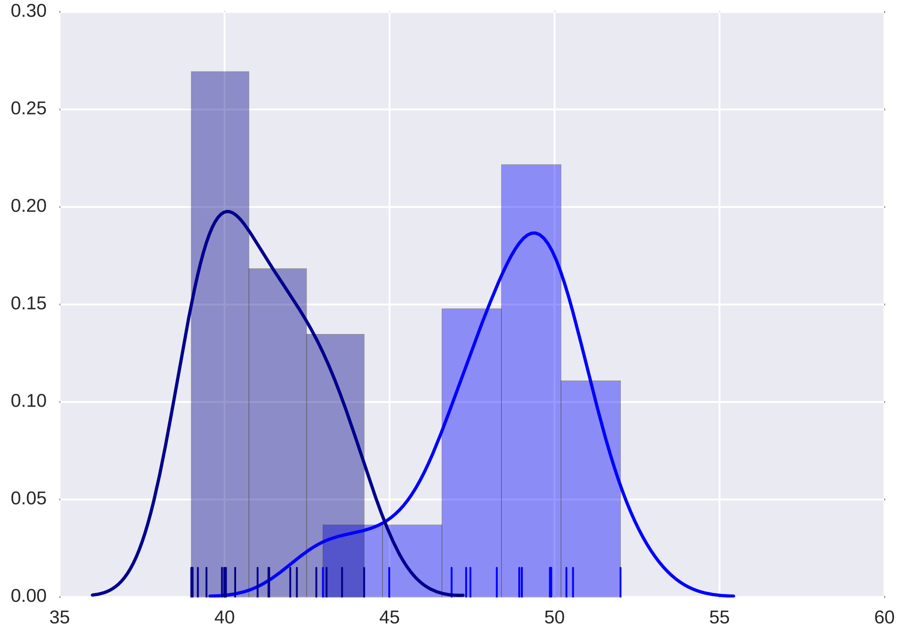

Figure 46: Significance Test Results for Catalase levels between the group treated with *Withania somnifera*(CAT_W) and the MPTP disease induced and treated with both *Withania somnifera* and *Centella asiatica* group(CAT_MWC). As both the sets were normal, a Student's T-test was used. The p-value obtained for the test was 0.0000 and hence the difference between the data sets was considered statistically significant.

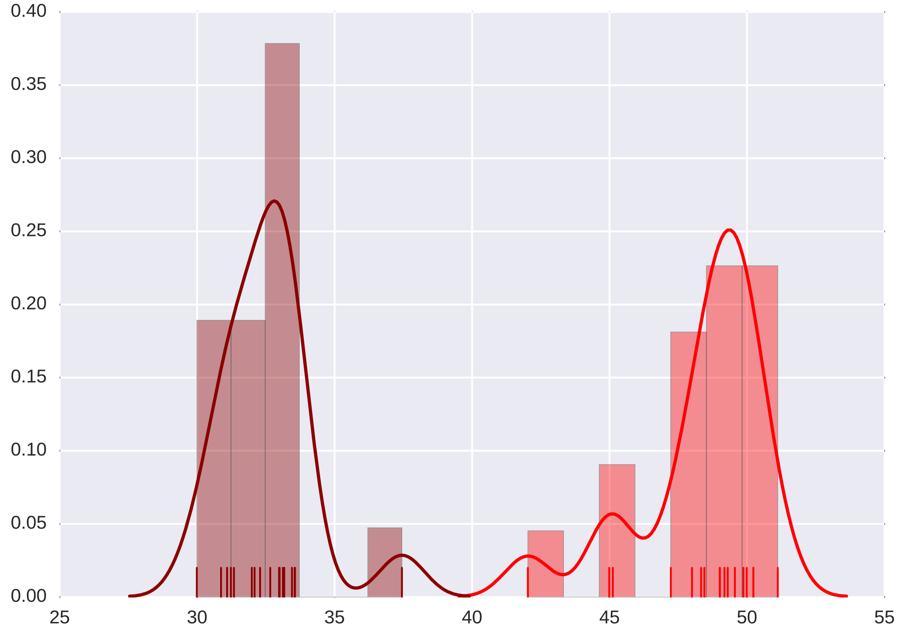

Figure 47: Significance Test Results for Catalase levels between the group treated with both *Withania somnifera* and *Centella asiatica*(CAT_WC) and the MPTP disease induced and no treatment group(CAT_M). As both the sets were non-normal, we used a Mann-Whitney U Test. The p-value obtained for the test was 0.0000 and hence the difference between the data sets was considered statistically significant.

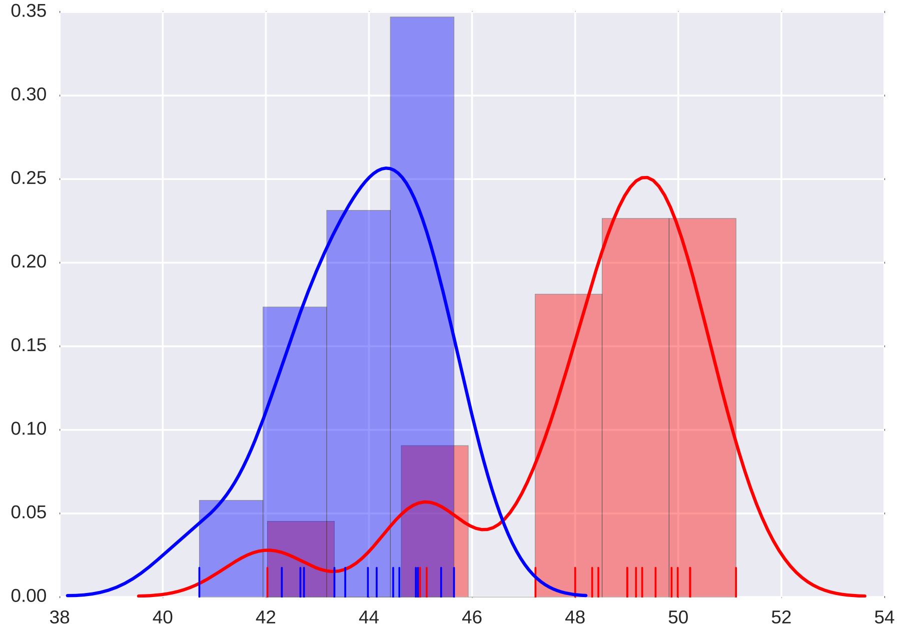

Figure 48: Significance Test Results for Catalase levels between the group treated with both *Withania somnifera* and *Centella asiatica*(CAT_WC) and the MPTP disease induced and treated with *Centella asiatica* group(CAT_MC). As one of the sets was non-normal, we used a Mann-Whitney U Test. The p-value obtained for the test was 0.0001 and hence the difference between the data sets was considered statistically significant.

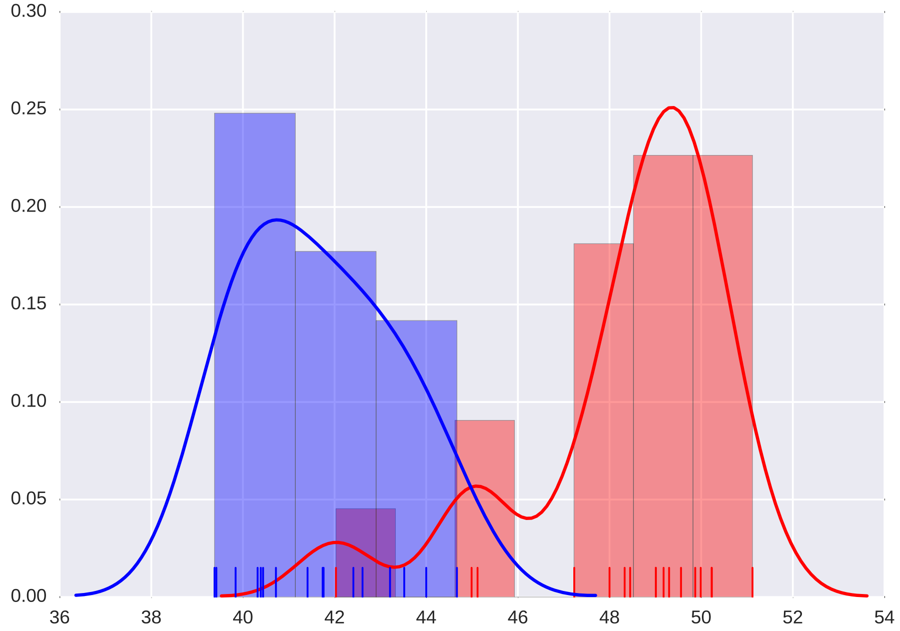

Figure 49: Significance Test Results for Catalase levels between the group treated with both *Withania somnifera* and *Centella asiatica*(CAT_WC) and the MPTP disease induced and treated with *Withania somnifera* group(CAT_MW). As one of the sets was non-normal, we used a Mann-Whitney U Test. The p-value obtained for the test was 0.0000 and hence the difference between the data sets was considered statistically significant.

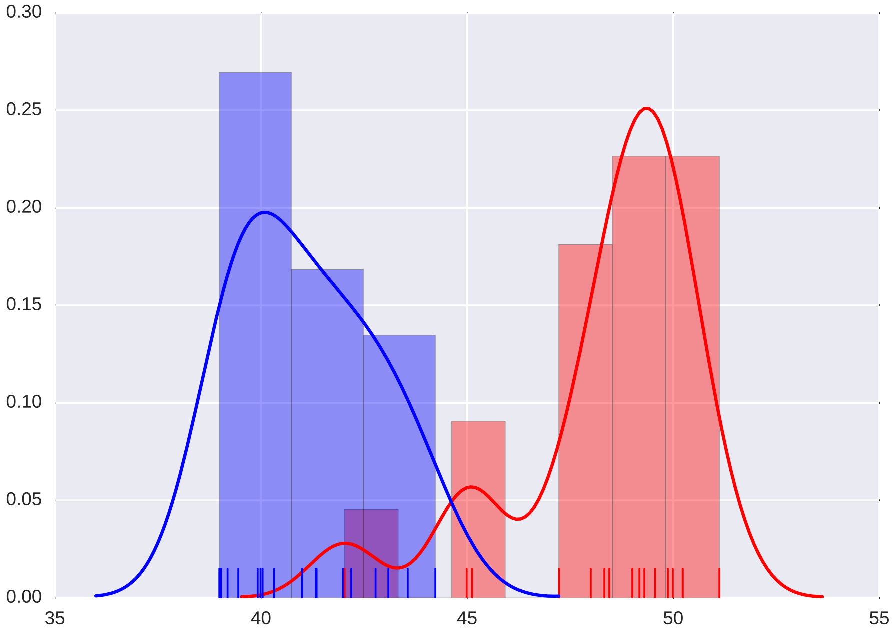

Figure 50: Significance Test Results for Catalase levels between the group treated with both *Withania somnifera* and *Centella asiatica*(CAT_WC) and the MPTP disease induced and treated with both *Withania somnifera* and *Centella asiatica* group(CAT_MWC). As one of the sets was non-normal, we used a Mann-Whitney U Test. The p-value obtained for the test was 0.0000 and hence the difference between the data sets was considered statistically significant.

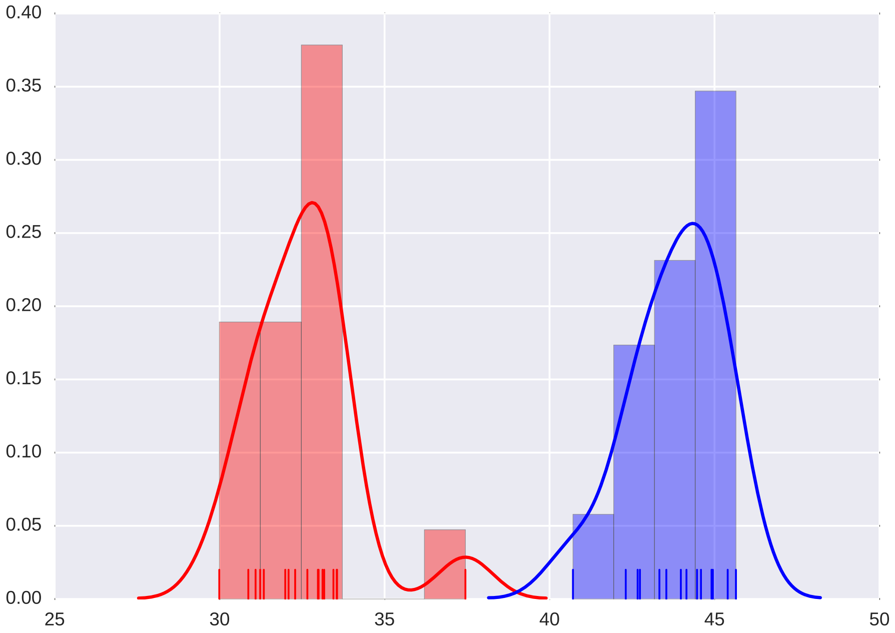

Figure 51: Significance Test Results for Catalase levels between the MPTP disease induced and no treatment group(CAT_M) and the MPTP disease induced and treated with *Centella asiatica* group(CAT_MC). As one of the sets was non-normal, we used a Mann-Whitney U Test. The p-value obtained for the test was 0.0000 and hence the difference between the data sets was considered statistically significant.

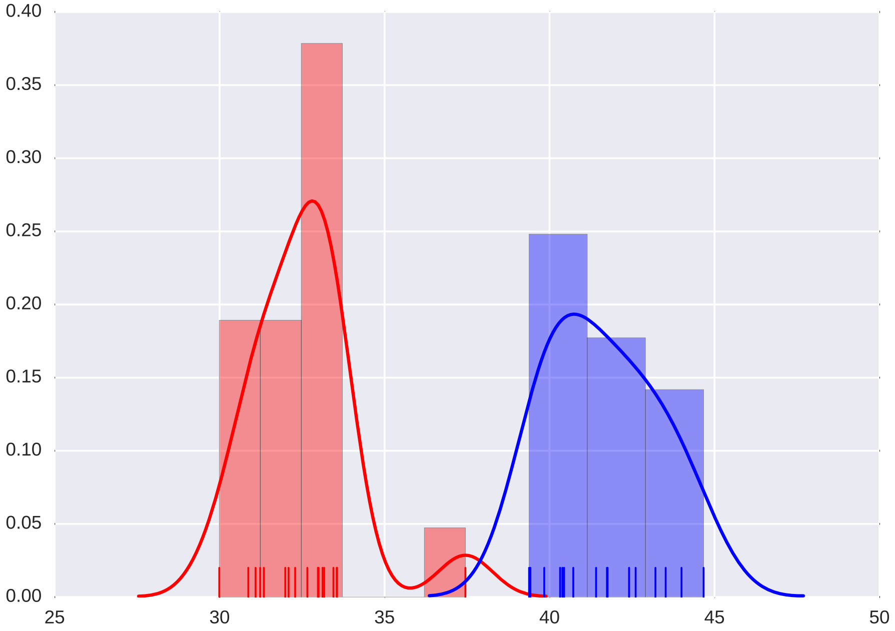

Figure 52: Significance Test Results for Catalase levels between the MPTP disease induced and no treatment group(CAT_M) and the MPTP disease induced and treated with *Withania somnifera* group(CAT_MW). As one of the sets was non-normal, we used a Mann-Whitney U Test. The p-value obtained for the test was 0.0000 and hence the difference between the data sets was considered statistically significant.

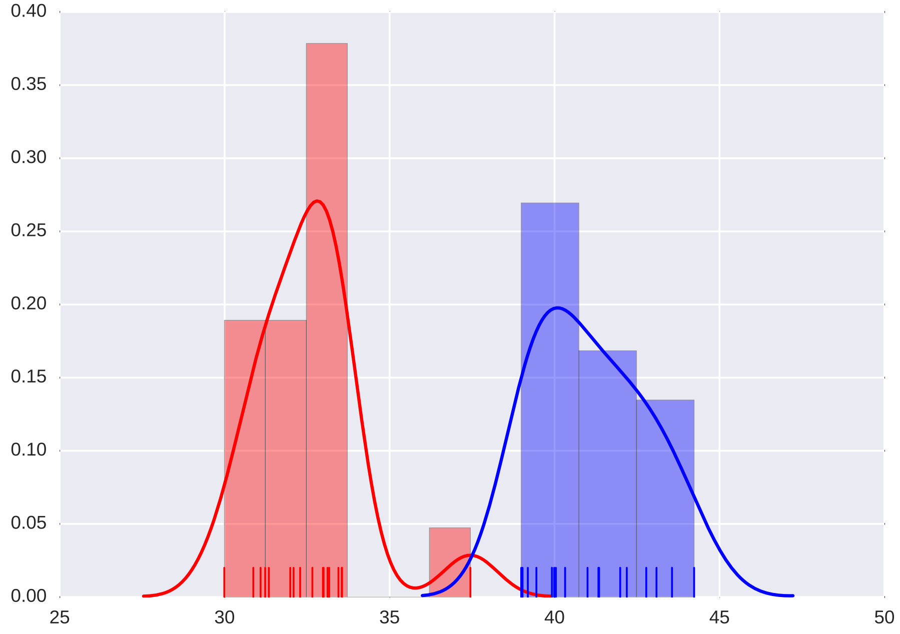

Figure 53: Significance Test Results for Catalase levels between the MPTP disease induced and no treatment group(CAT_M) and the MPTP disease induced and treated with both *Withania somnifera* and *Centella asiatica* group(CAT_MWC). As one of the sets was non-normal, we used a Mann-Whitney U Test. The p-value obtained for the test was 0.0000 and hence the difference between the data sets was considered statistically significant.

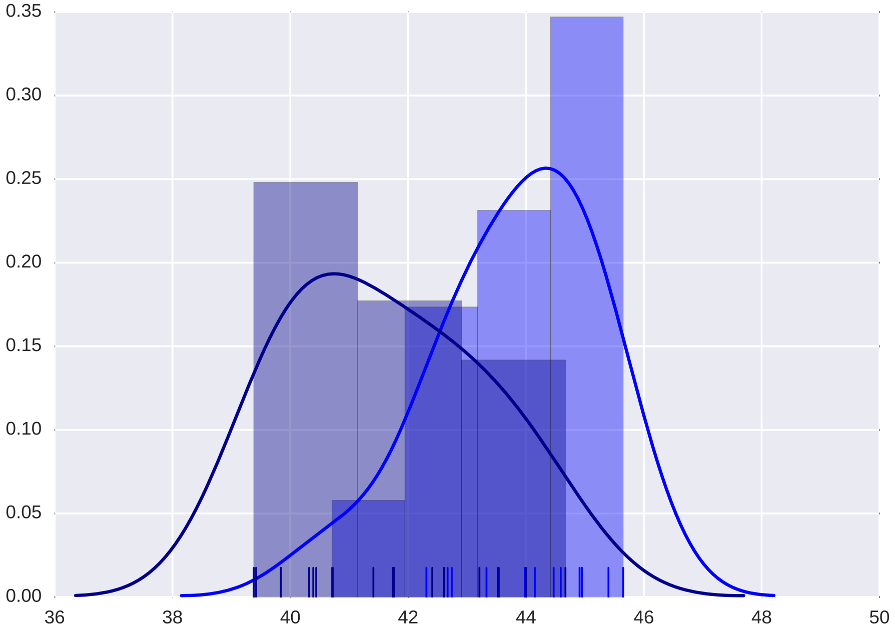

Figure 54: Significance Test Results for Catalase levels between the MPTP disease induced and treated with *Centella asiatica* group(CAT_MC) and the MPTP disease induced and treated with *Withania somnifera* group(CAT_MW). As both the sets were normal, a Student's T-test was used. The p-value obtained for the test was 0.0005 and hence the difference between the data sets was considered statistically significant.

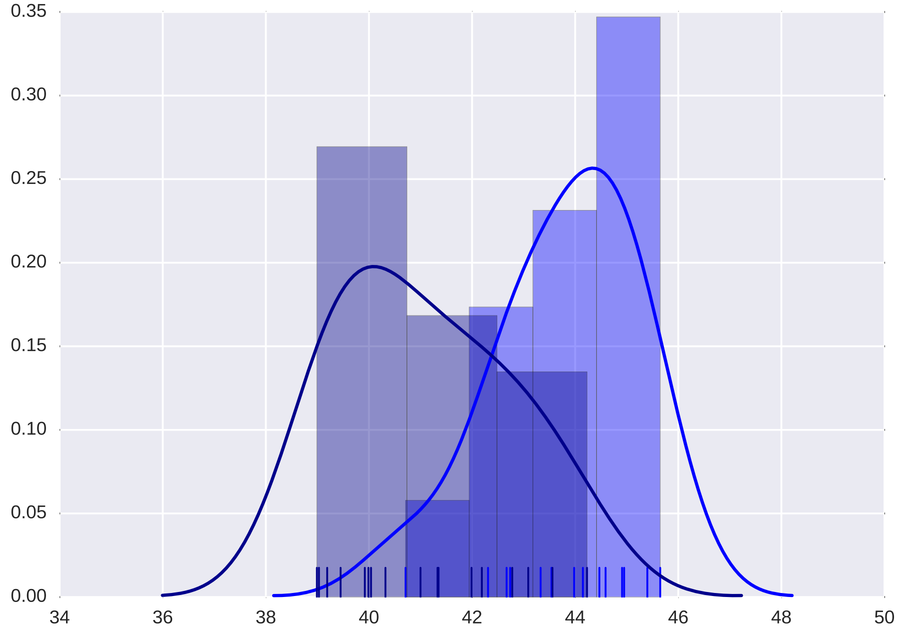

Figure 55: Significance Test Results for Catalase levels between the MPTP disease induced and treated with *Centella asiatica* group(CAT_MC) and the MPTP disease induced and treated with both *Withania somnifera* and *Centella asiatica* group(CAT_MWC). As both the sets were normal, a Student's T-test was used. The p-value obtained for the test was 0.0000 and hence the difference between the data sets was considered statistically significant.

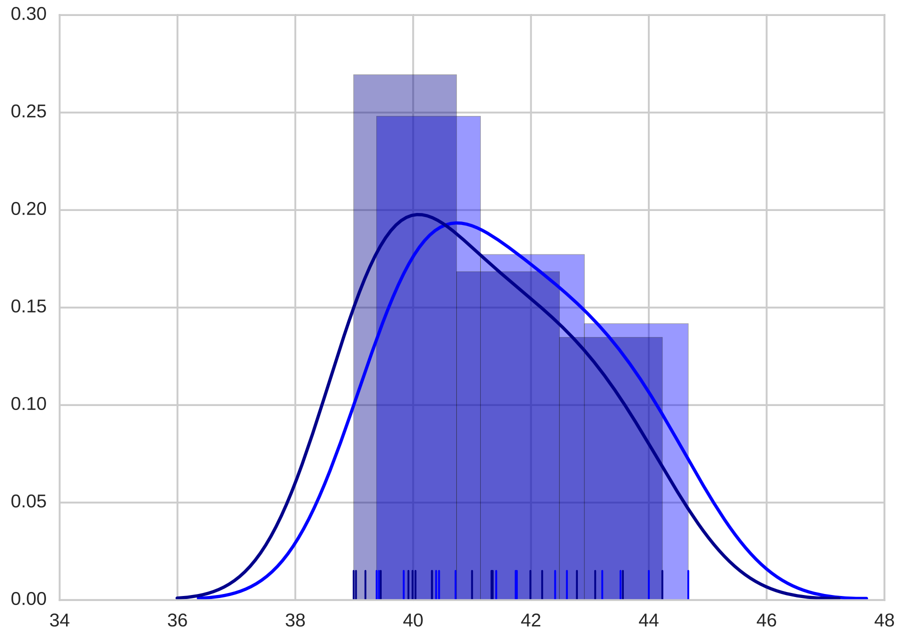

Figure 56: Significance Test Results for Catalase levels between the MPTP disease induced and treated with *Withania somnifera* group(CAT_MW) and the MPTP disease induced and treated with both *Withania somnifera* and *Centella asiatica* group(CAT_MWC). As both the sets were normal, a Student's T-test was used. The p-value obtained for the test was 0.3667 and hence the difference between the data sets was considered statistically non-significant.

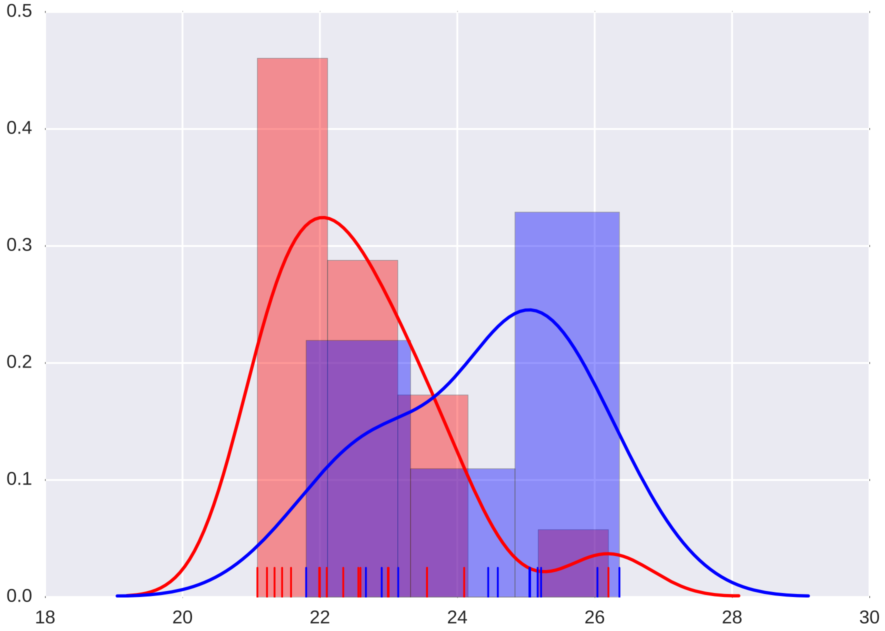

Figure 57: Significance Test Results for Glutathione peroxidase levels between the untreated group(GPx_U) and the group treated with *Centella asiatica*(GPx_C). As one of the sets was non-normal, we used a Mann-Whitney U Test. The p-value obtained for the test was 0.0032 and hence the difference between the data sets was considered statistically significant.

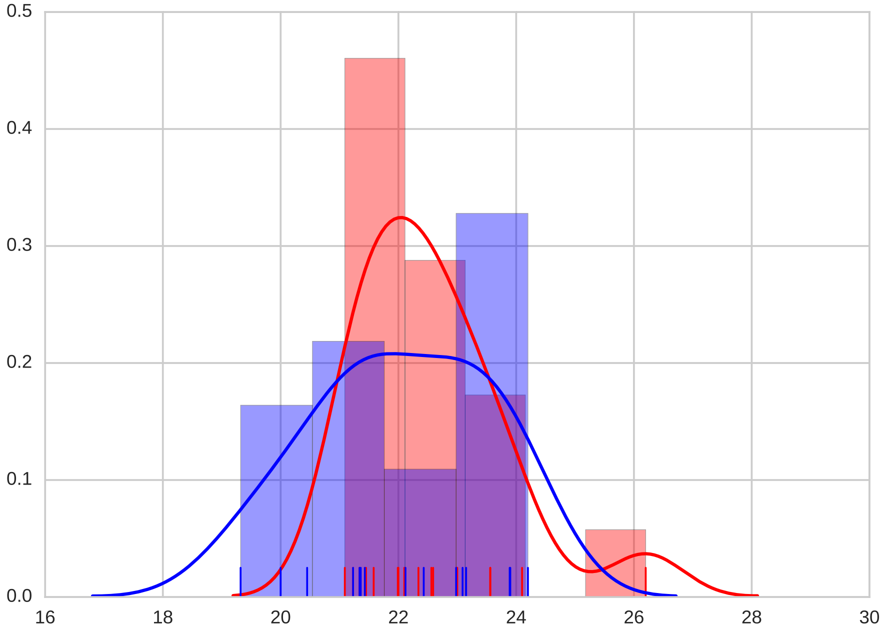

Figure 58: Significance Test Results for Glutathione peroxidase levels between the untreated group(GPx_U) and the group treated with *Withania somnifera*(GPx_W). As one of the sets was non-normal, we used a Mann-Whitney U Test. The p-value obtained for the test was 0.5208 and hence the difference between the data sets was considered statistically non-significant.

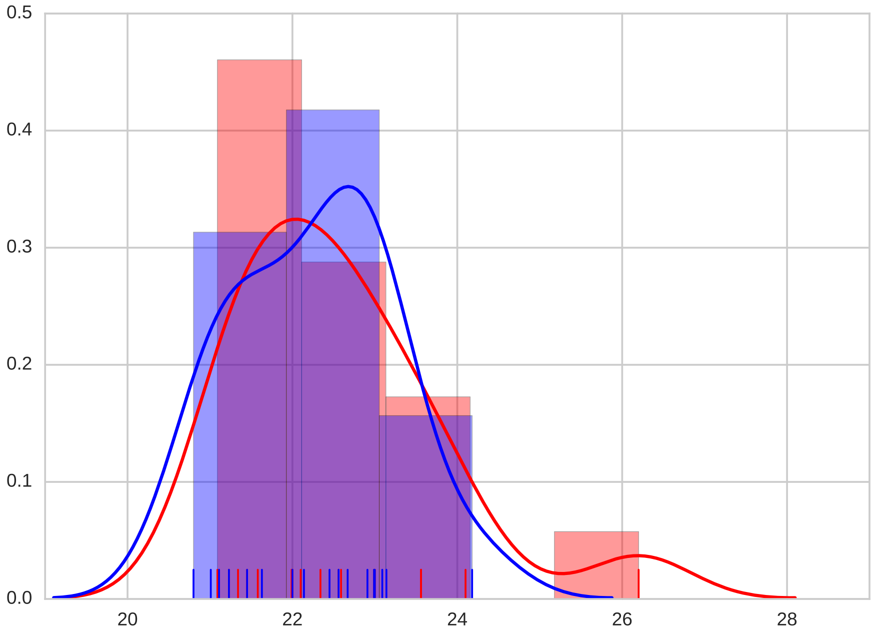

Figure 59: Significance Test Results for Glutathione peroxidase levels between the untreated group(GPx_U) and the group treated with both *Withania somnifera* and *Centella asiatica*(GPx_WC). As one of the sets was non-normal, we used a Mann-Whitney U Test. The p-value obtained for the test was 0.6792 and hence the difference between the data sets was considered statistically non-significant.

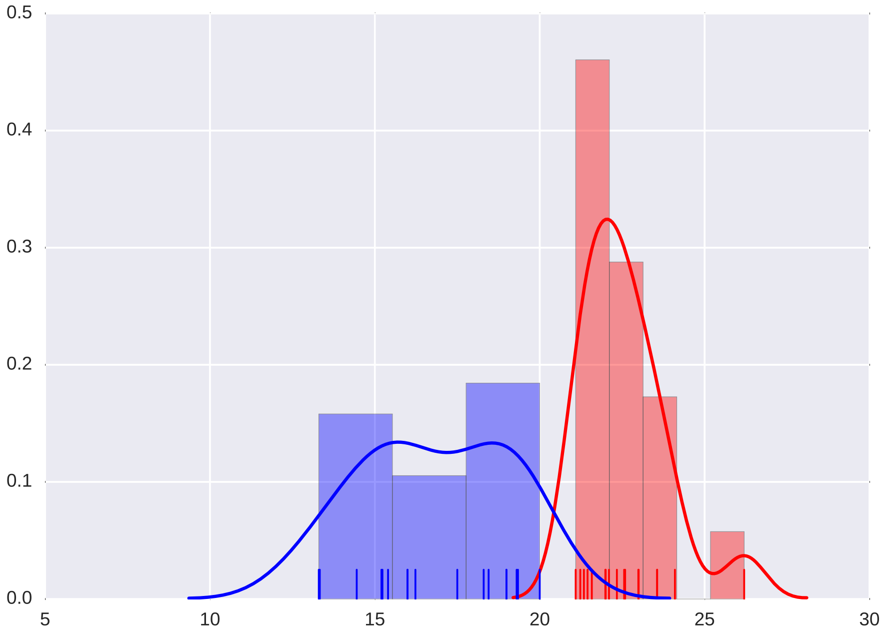

Figure 60: Significance Test Results for Glutathione peroxidase levels between the untreated group(GPx_U) and the MPTP disease induced and no treatment group(GPx_M). As one of the sets was non-normal but the variances were unequal, we used a Welch's T Test with ranked data. The p-value obtained for the test was 0.0000 and hence the difference between the data sets was considered statistically significant.

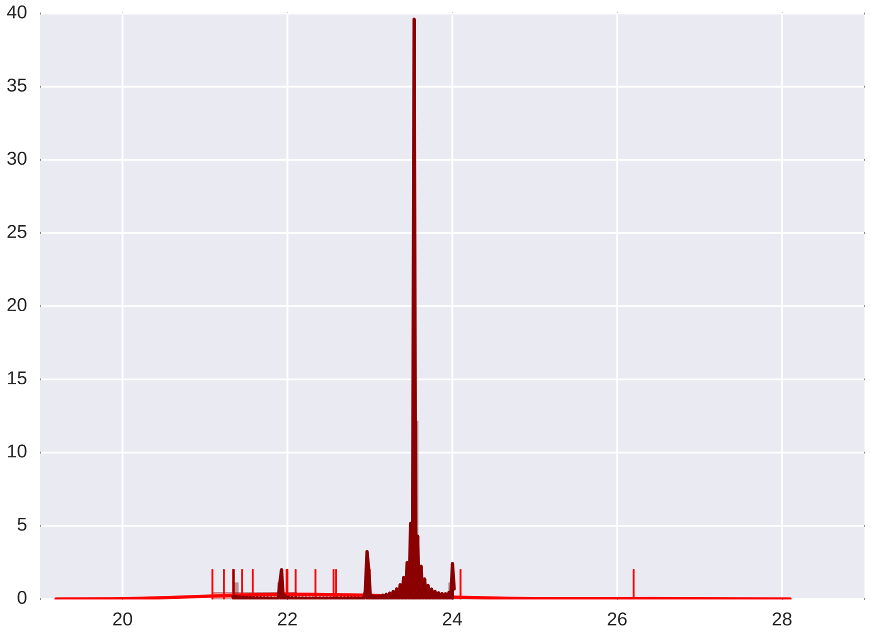

Figure 61: Significance Test Results for Glutathione peroxidase levels between the untreated group(GPx_U) and the MPTP disease induced and treated with *Centella asiatica* group(GPx_MC). As both the sets were non-normal but the variances were unequal, we used a Welch's T Test with ranked data. The p-value obtained for the test was 0.0474 and hence the difference between the data sets was considered statistically significant.

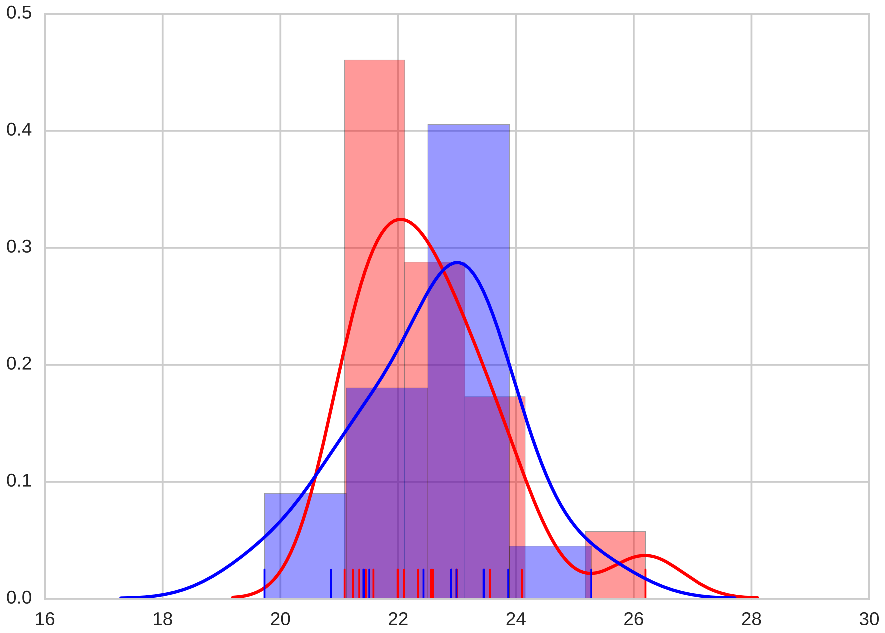

Figure 62: Significance Test Results for Glutathione peroxidase levels between the untreated group(GPx_U) and the MPTP disease induced and treated with *Withania somnifera* group(GPx_MW). As one of the sets was non-normal, we used a Mann-Whitney U Test. The p-value obtained for the test was 0.7730 and hence the difference between the data sets was considered statistically non-significant.

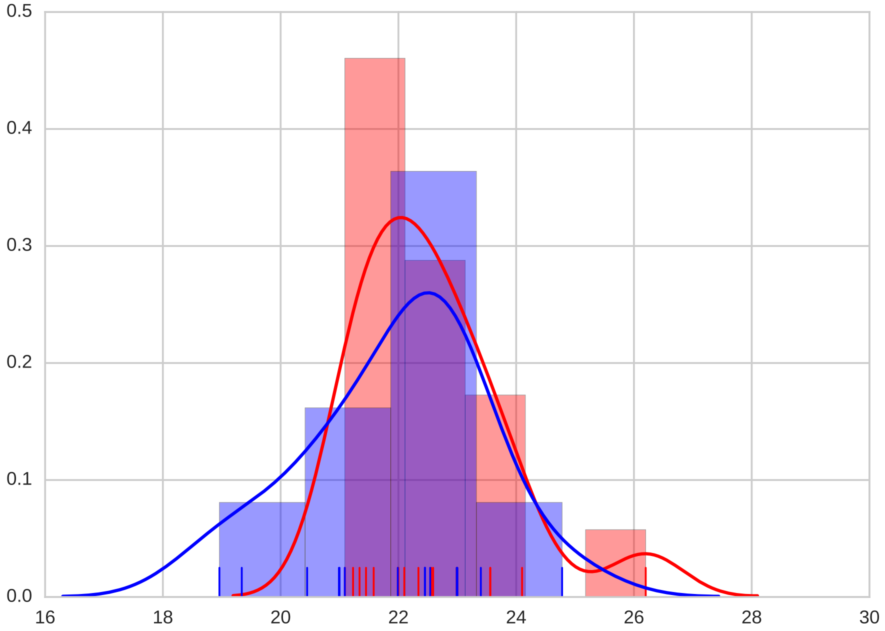

Figure 63: Significance Test Results for Glutathione peroxidase levels between the untreated group(GPx_U) and the MPTP disease induced and treated with both *Withania somnifera* and *Centella asiatica* group(GPx_MWC). As one of the sets was non-normal, we used a Mann-Whitney U Test. The p-value obtained for the test was 0.3429 and hence the difference between the data sets was considered statistically non-significant.

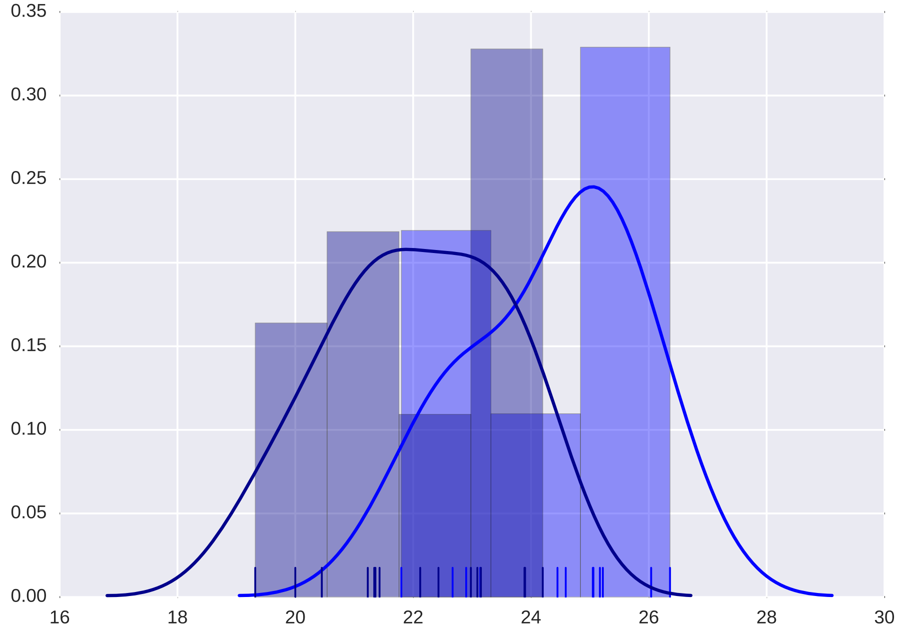

Figure 64: Significance Test Results for Glutathione peroxidase levels between the group treated with *Centella asiatica*(GPx_C) and the group treated with *Withania somnifera*(GPx_W). As both the sets were normal, a Student's T-test was used. The p-value obtained for the test was 0.0004 and hence the difference between the data sets was considered statistically significant.

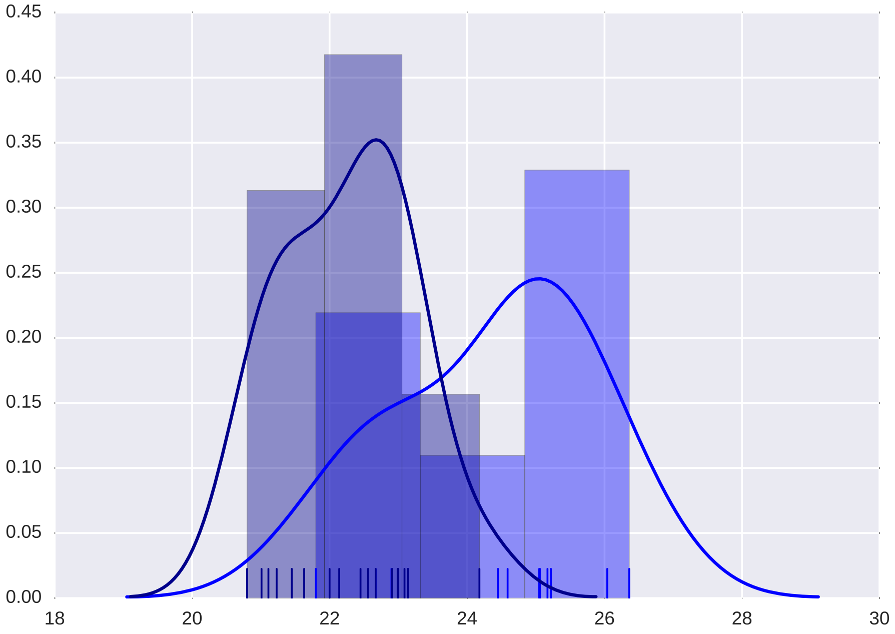

Figure 65: Significance Test Results for Glutathione peroxidase levels between the group treated with *Centella asiatica*(GPx_C) and the group treated with both *Withania somnifera* and *Centella asiatica*(GPx_WC). As both the sets were normal, a Student's T-test was used. The p-value obtained for the test was 0.0000 and hence the difference between the data sets was considered statistically significant.

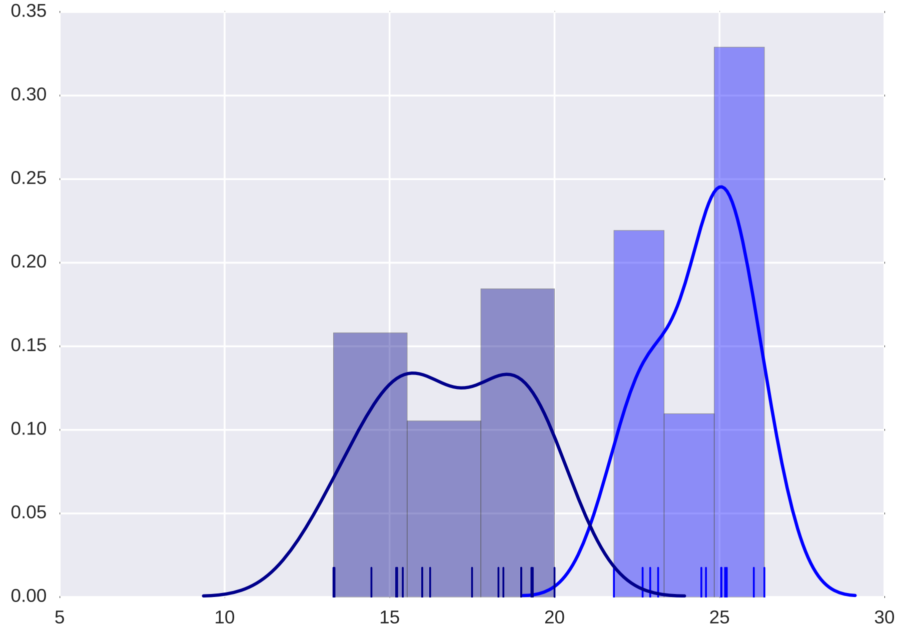

Figure 66: Significance Test Results for Glutathione peroxidase levels between the group treated with *Centella asiatica*(GPx_C) and the MPTP disease induced and no treatment group(GPx_M). As both the sets were normal, a Student's T-test was used. The p-value obtained for the test was 0.0000 and hence the difference between the data sets was considered statistically significant.

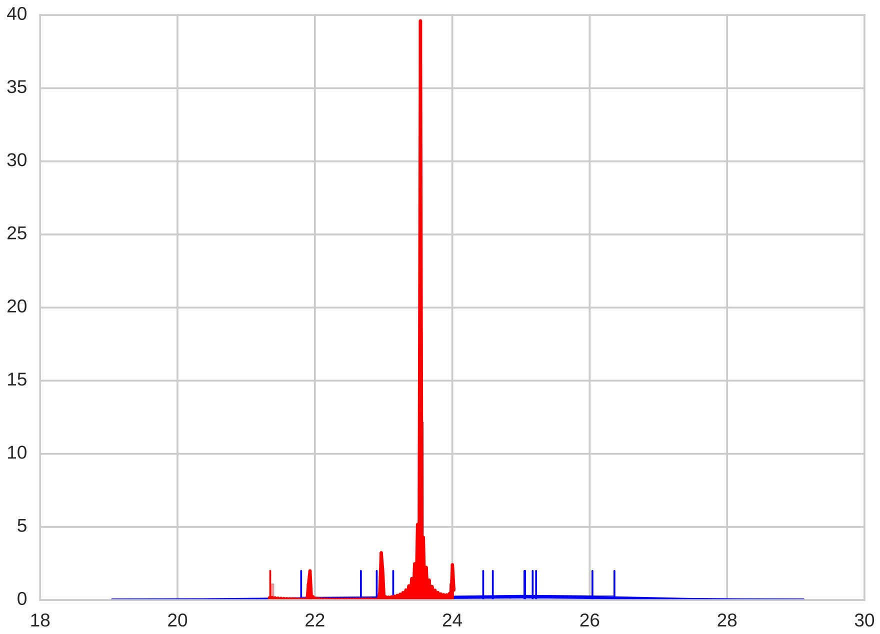

Figure 67: Significance Test Results for Glutathione peroxidase levels between the group treated with *Centella asiatica*(GPx_C) and the MPTP disease induced and treated with *Centella asiatica* group(GPx_MC). As one of the sets was non-normal but the variances were unequal, we used a Welch's T Test with ranked data. The p-value obtained for the test was 0.0827 and hence the difference between the data sets was considered statistically non-significant.

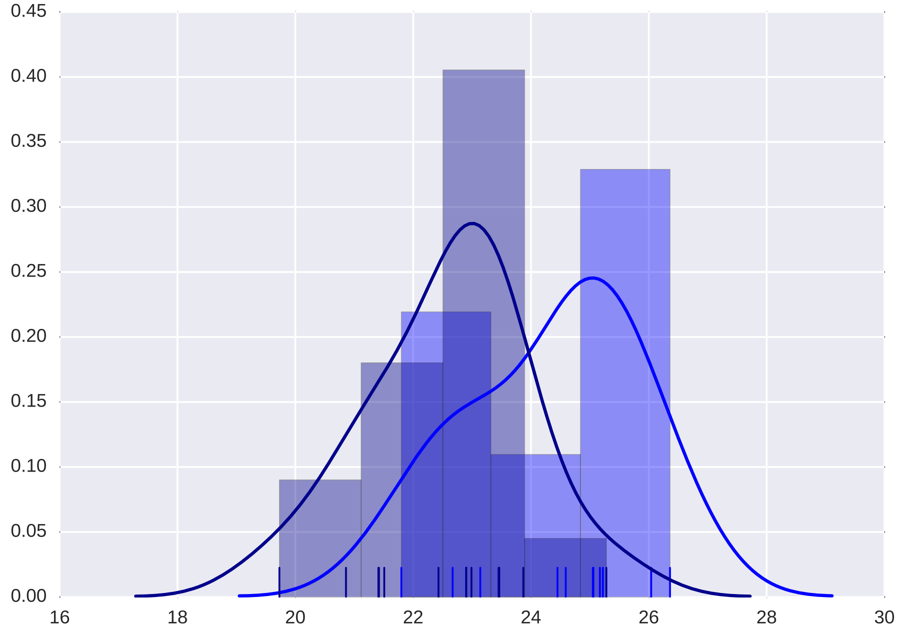

Figure 68: Significance Test Results for Glutathione peroxidase levels between the group treated with *Centella asiatica*(GPx_C) and the MPTP disease induced and treated with *Withania somnifera* group(GPx_MW). As both the sets were normal, a Student's T-test was used. The p-value obtained for the test was 0.0022 and hence the difference between the data sets was considered statistically significant.

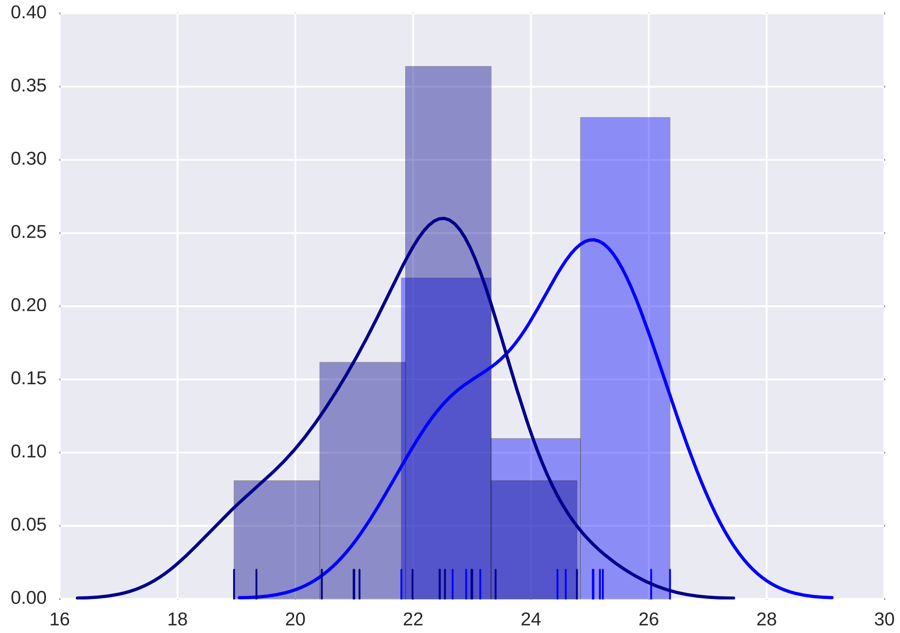

Figure 69: Significance Test Results for Glutathione peroxidase levels between the group treated with *Centella asiatica*(GPx_C) and the MPTP disease induced and treated with both *Withania somnifera* and *Centella asiatica* group(GPx_MWC). As both the sets were normal, a Student's T-test was used. The p-value obtained for the test was 0.0002 and hence the difference between the data sets was considered statistically significant.

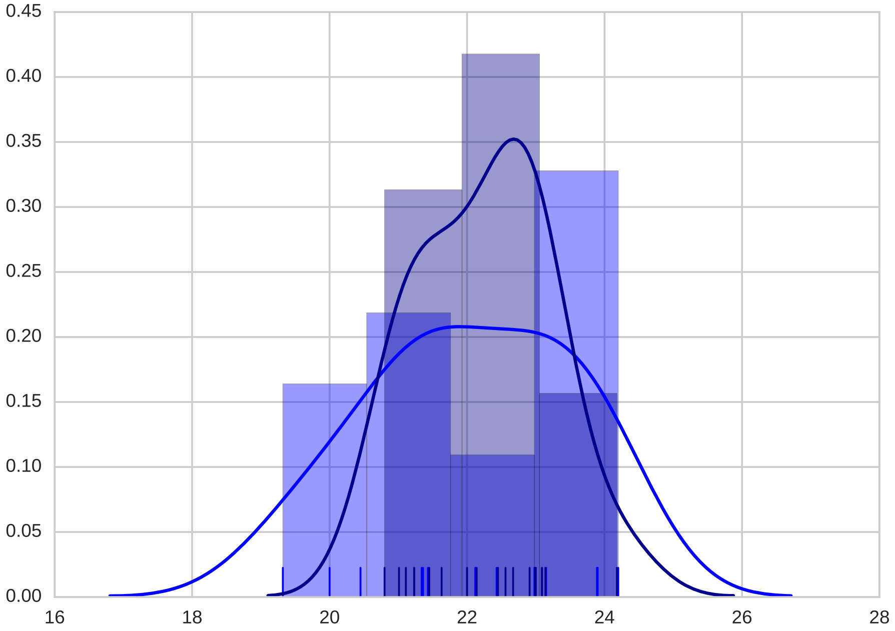

Figure 70: Significance Test Results for Glutathione peroxidase levels between the group treated with *Withania somnifera*(GPx_W) and the group treated with both *Withania somnifera* and *Centella asiatica*(GPx_WC). As both the sets were normal, a Student's T-test was used. The p-value obtained for the test was 0.6534 and hence the difference between the data sets was considered statistically non-significant.

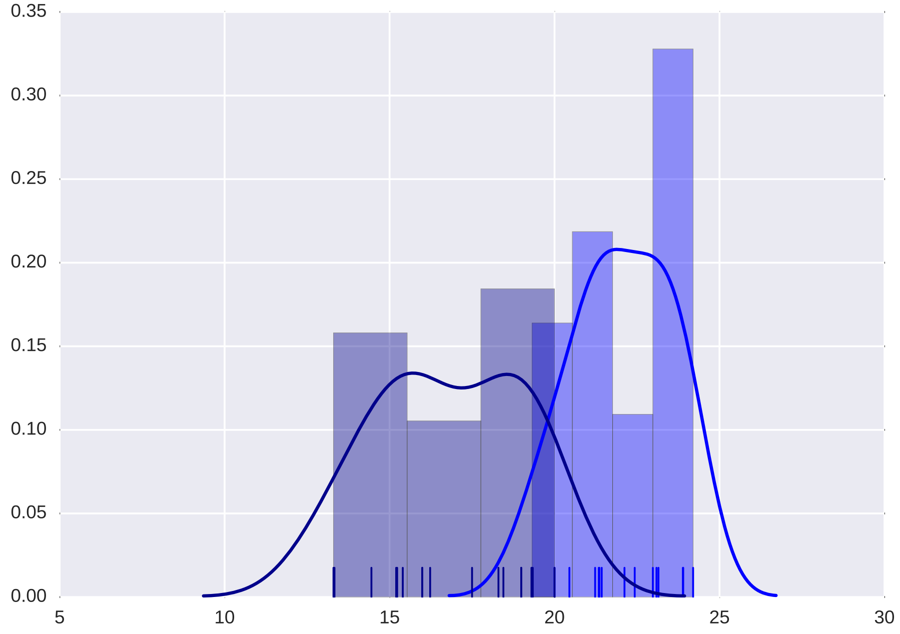

Figure 71: Significance Test Results for Glutathione peroxidase levels between the group treated with *Withania somnifera*(GPx_W) and the MPTP disease induced and no treatment group(GPx_M). As both the sets were normal, a Student's T-test was used. The p-value obtained for the test was 0.0000 and hence the difference between the data sets was considered statistically significant.

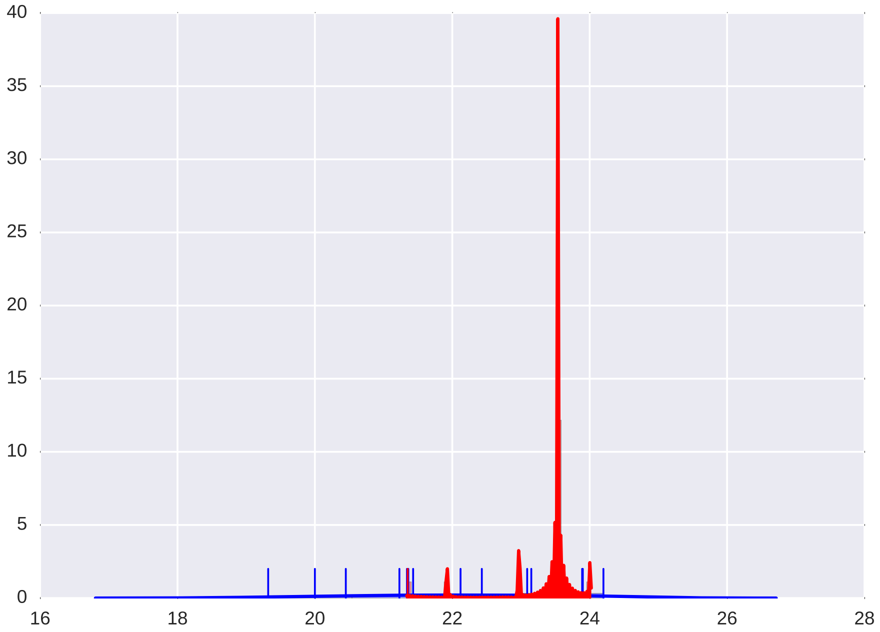

Figure 72: Significance Test Results for Glutathione peroxidase levels between the group treated with *Withania somnifera*(GPx_W) and the MPTP disease induced and treated with *Centella asiatica* group(GPx_MC). As one of the sets was non-normal but the variances were unequal, we used a Welch's T Test with ranked data. The p-value obtained for the test was 0.0210 and hence the difference between the data sets was considered statistically significant.

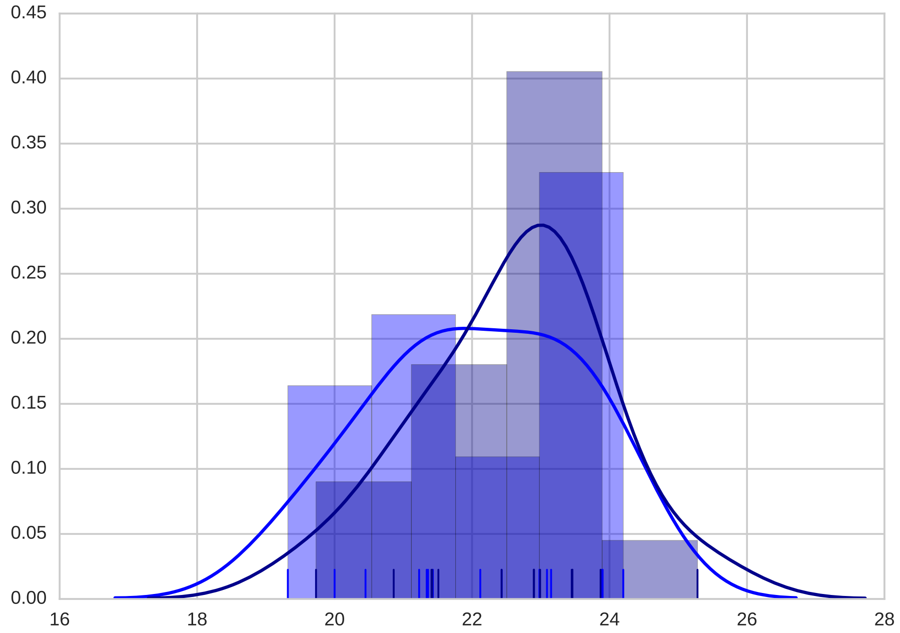

Figure 73: Significance Test Results for Glutathione peroxidase levels between the group treated with *Withania somnifera*(GPx_W) and the MPTP disease induced and treated with *Withania somnifera* group(GPx_MW). As both the sets were normal, a Student's T-test was used. The p-value obtained for the test was 0.3025 and hence the difference between the data sets was considered statistically non-significant.

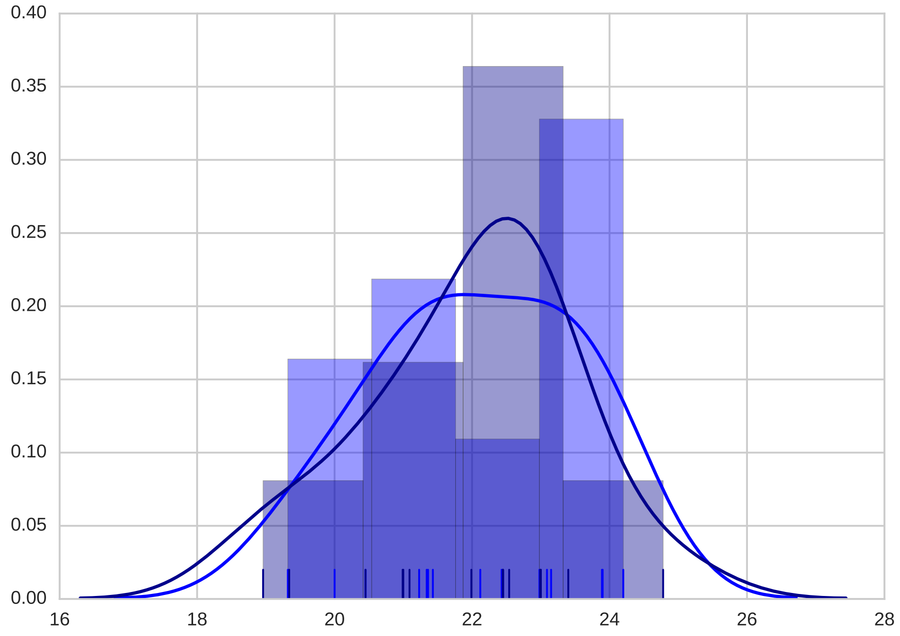

Figure 74: Significance Test Results for Glutathione peroxidase levels between the group treated with *Withania somnifera*(GPx_W) and the MPTP disease induced and treated with both *Withania somnifera* and *Centella asiatica* group(GPx_MWC). As both the sets were normal, a Student's T-test was used. The p-value obtained for the test was 0.8525 and hence the difference between the data sets was considered statistically non-significant.

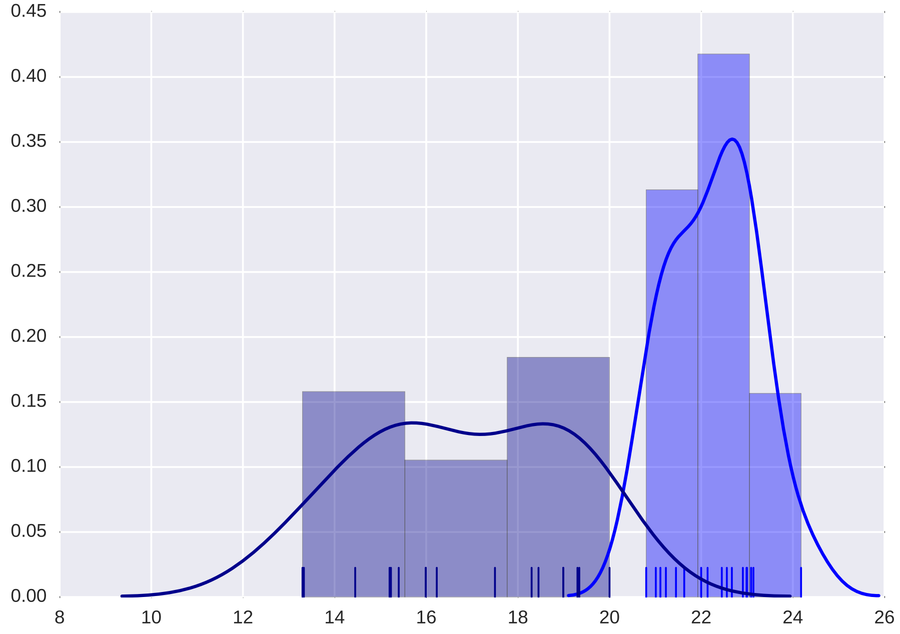

Figure 75: Significance Test Results for Glutathione peroxidase levels between the group treated with both *Withania somnifera* and *Centella asiatica*(GPx_WC) and the MPTP disease induced and no treatment group(GPx_M). As both the sets were normal but with unequal variances, a Welch's T-test was used. The p-value obtained for the test was 0.0000 and hence the difference between the data sets was considered statistically significant.

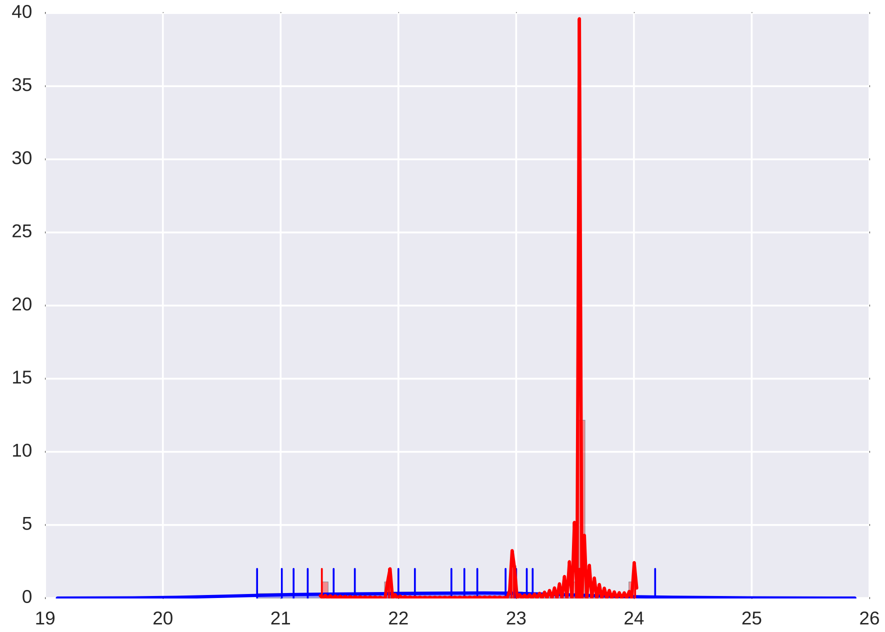

Figure 76: Significance Test Results for Glutathione peroxidase levels between the group treated with both *Withania somnifera* and *Centella asiatica*(GPx_WC) and the MPTP disease induced and treated with *Centella asiatica* group(GPx_MC). As one of the sets was non-normal but the variances were unequal, we used a Welch's T Test with ranked data. The p-value obtained for the test was 0.0002 and hence the difference between the data sets was considered statistically significant.

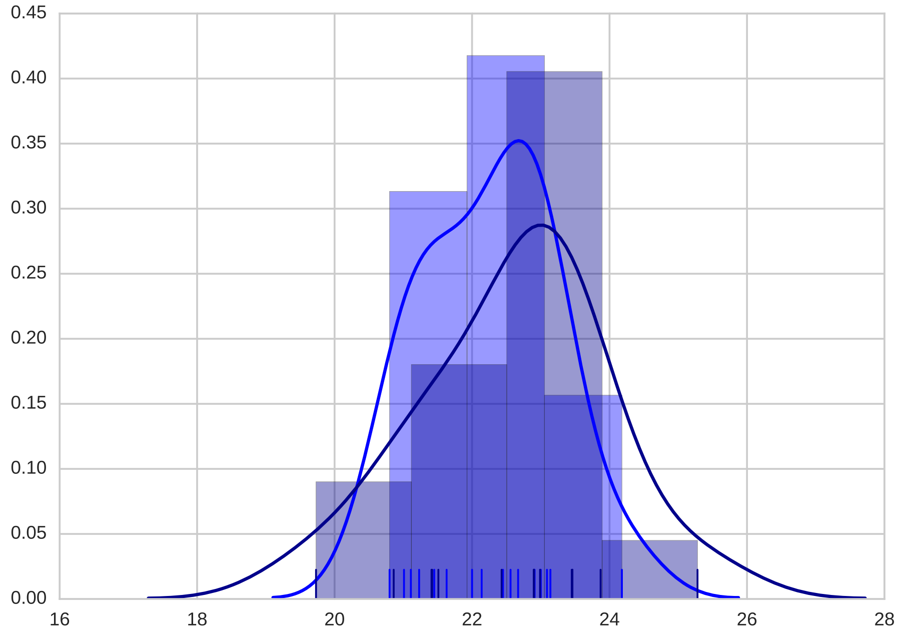

Figure 77: Significance Test Results for Glutathione peroxidase levels between the group treated with both *Withania somnifera* and *Centella asiatica*(GPx_WC) and the MPTP disease induced and treated with *Withania somnifera* group(GPx_MW). As both the sets were normal, a Student's T-test was used. The p-value obtained for the test was 0.4086 and hence the difference between the data sets was considered statistically non-significant.

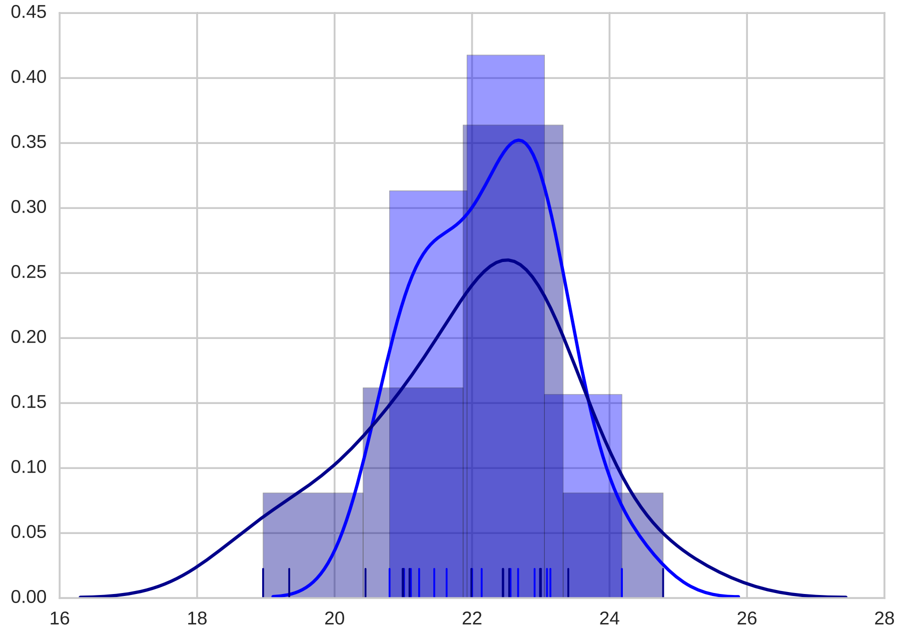

Figure 78: Significance Test Results for Glutathione peroxidase levels between the group treated with both *Withania somnifera* and *Centella asiatica*(GPx_WC) and the MPTP disease induced and treated with both *Withania somnifera* and *Centella asiatica* group(GPx_MWC). As both the sets were normal, a Student's T-test was used. The p-value obtained for the test was 0.4928 and hence the difference between the data sets was considered statistically non-significant.

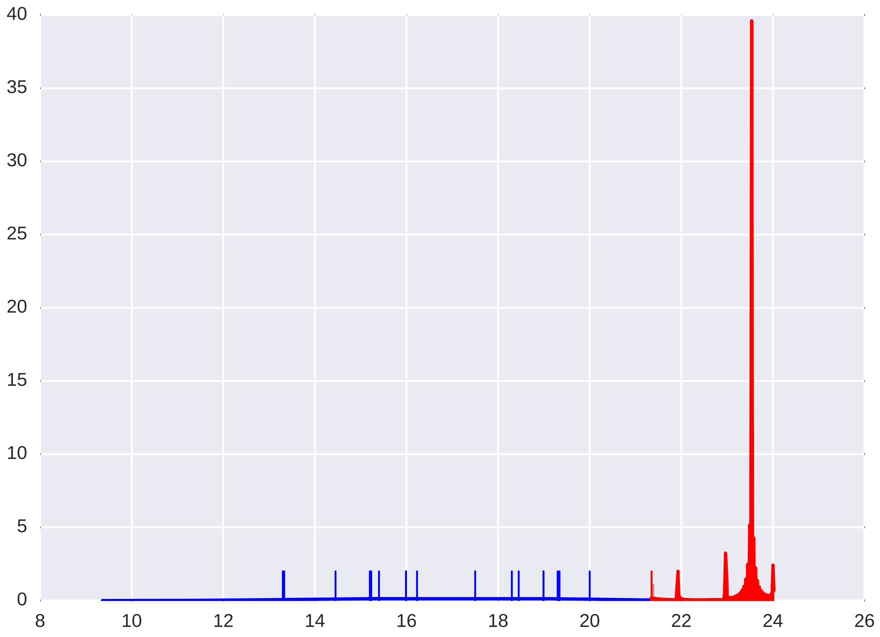

Figure 79: Significance Test Results for Glutathione peroxidase levels between the MPTP disease induced and no treatment group(GPx_M) and the MPTP disease induced and treated with *Centella asiatica* group(GPx_MC). As one of the sets was non-normal but the variances were unequal, we used a Welch's T Test with ranked data. The p-value obtained for the test was 0.0000 and hence the difference between the data sets was considered statistically significant.

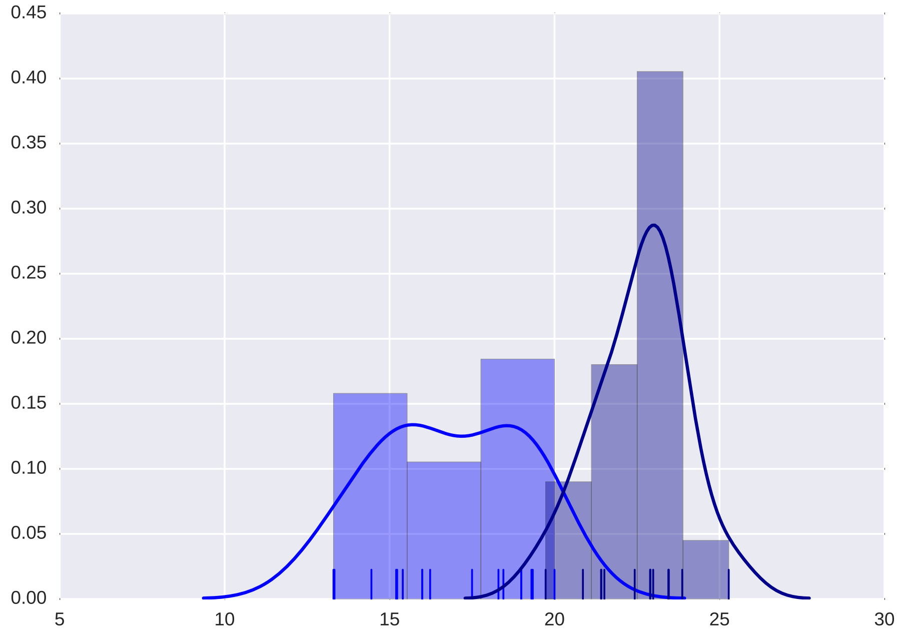

Figure 80: Significance Test Results for Glutathione peroxidase levels between the MPTP disease induced and no treatment group(GPx_M) and the MPTP disease induced and treated with *Withania somnifera* group(GPx_MW). As both the sets were normal but with unequal variances, a Welch's T-test was used. The p-value obtained for the test was 0.0000 and hence the difference between the data sets was considered statistically significant.

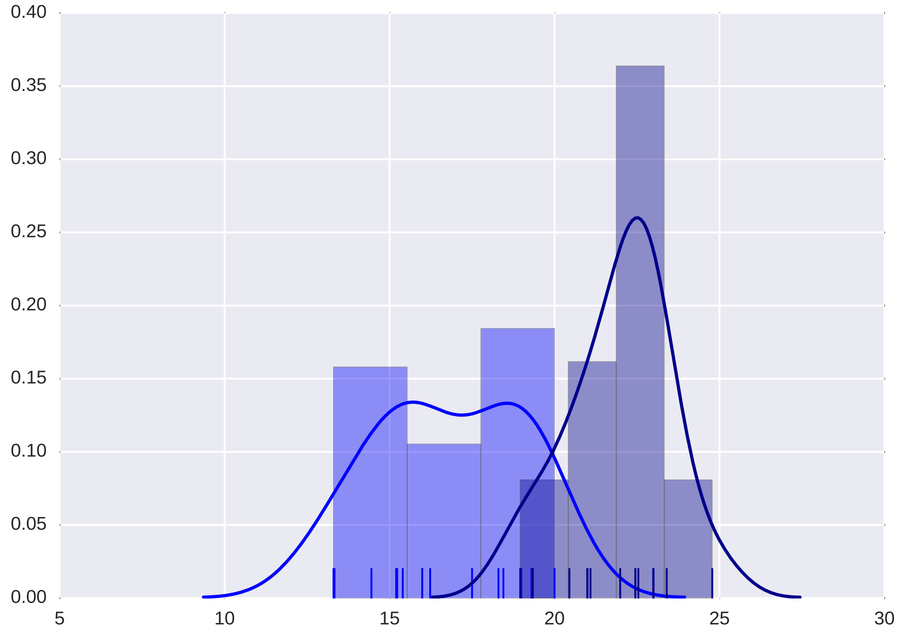

Figure 81: Significance Test Results for Glutathione peroxidase levels between the MPTP disease induced and no treatment group(GPx_M) and the MPTP disease induced and treated with both *Withania somnifera* and *Centella asiatica* group(GPx_MWC). As both the sets were normal but with unequal variances, a Welch's T-test was used. The p-value obtained for the test was 0.0000 and hence the difference between the data sets was considered statistically significant.

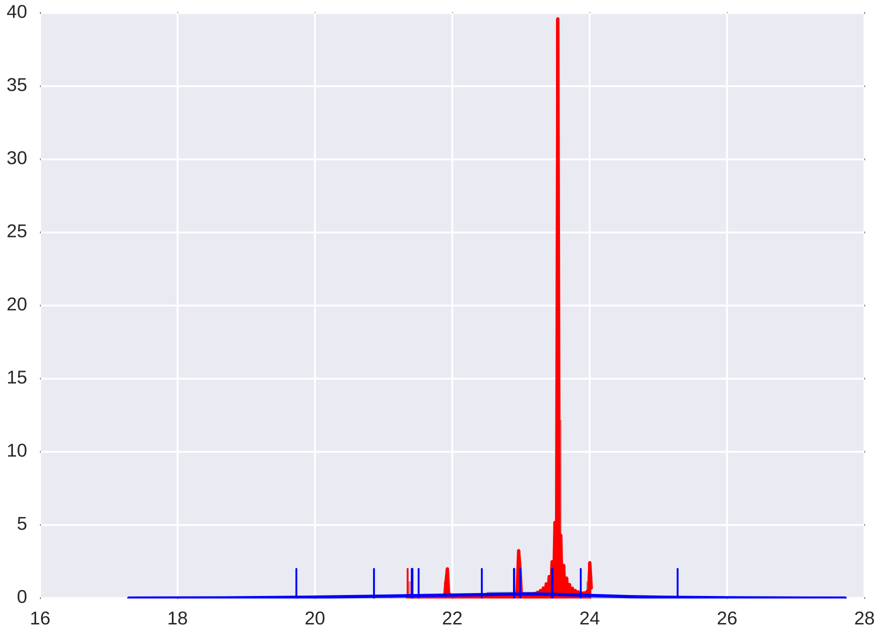

Figure 82: Significance Test Results for Glutathione peroxidase levels between the MPTP disease induced and treated with *Centella asiatica* group(GPx_MC) and the MPTP disease induced and treated with *Withania somnifera* group(GPx_MW). As one of the sets was non-normal but the variances were unequal, we used a Welch's T Test with ranked data. The p-value obtained for the test was 0.0050 and hence the difference between the data sets was considered statistically significant.

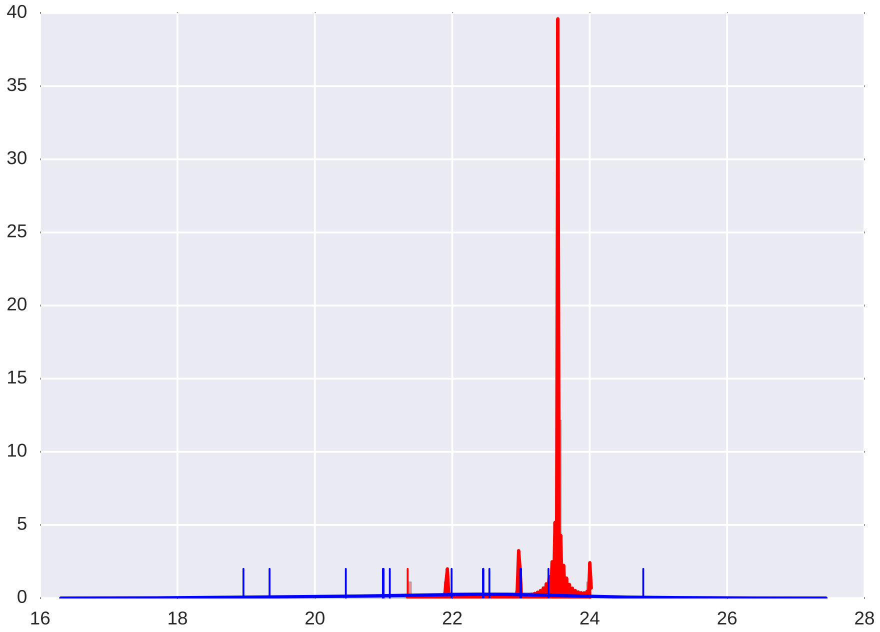

Figure 83: Significance Test Results for Glutathione peroxidase levels between the MPTP disease induced and treated with *Centella asiatica* group(GPx_MC) and the MPTP disease induced and treated with both *Withania somnifera* and *Centella asiatica* group(GPx_MWC). As one of the sets was non-normal but the variances were unequal, we used a Welch's T Test with ranked data. The p-value obtained for the test was 0.0001 and hence the difference between the data sets was considered statistically significant.

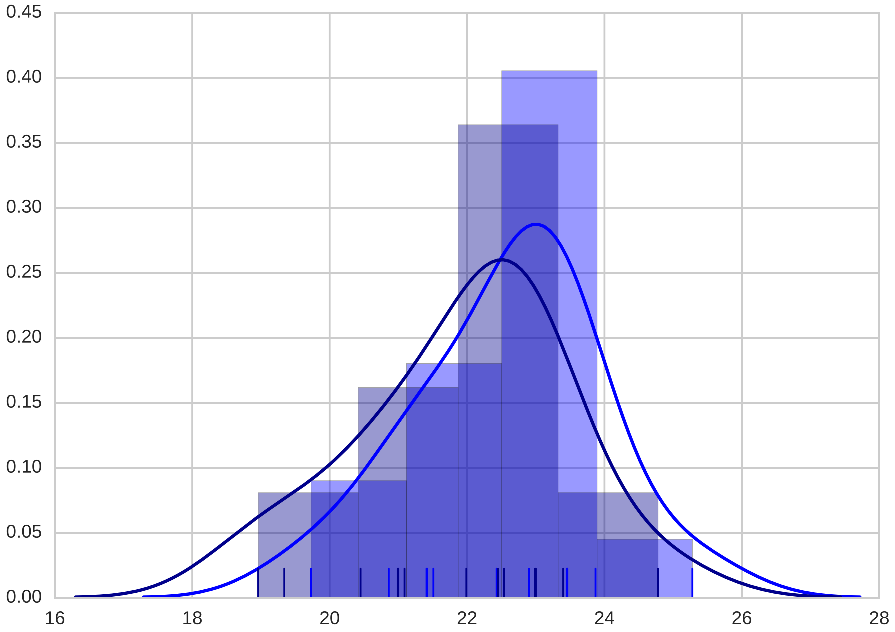

Figure 84: Significance Test Results for Glutathione peroxidase levels between the MPTP disease induced and treated with *Withania somnifera* group(GPx_MW) and the MPTP disease induced and treated with both *Withania somnifera* and *Centella asiatica* group(GPx_MWC). As both the sets were normal, a Student's T-test was used. The p-value obtained for the test was 0.2099 and hence the difference between the data sets was considered statistically non-significant.

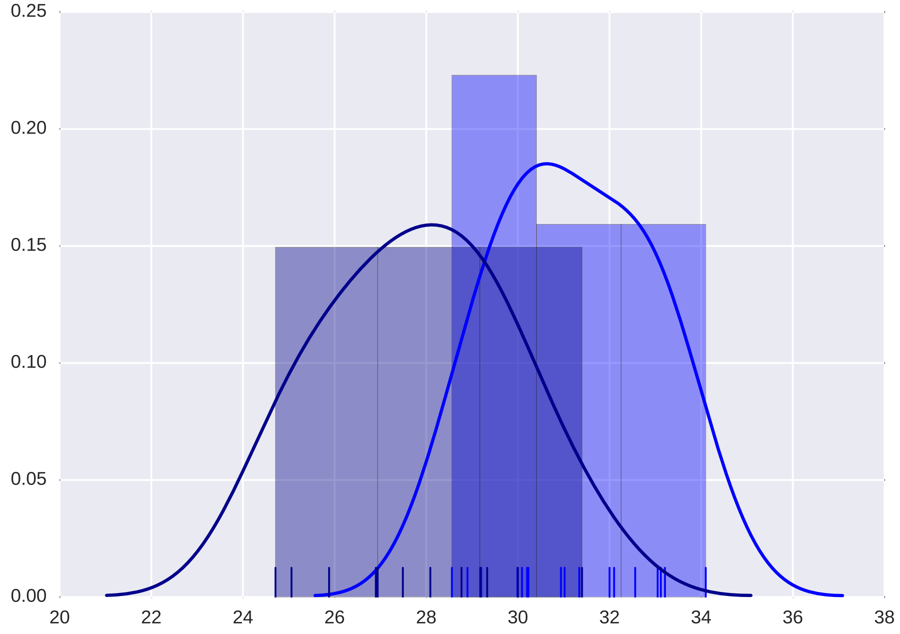

Figure 85: Significance Test Results for Reduced glutathione levels between the untreated group(GSH_U) and the group treated with *Centella asiatica*(GSH_C). As both the sets were normal, a Student's T-test was used. The p-value obtained for the test was 0.0000 and hence the difference between the data sets was considered statistically significant.

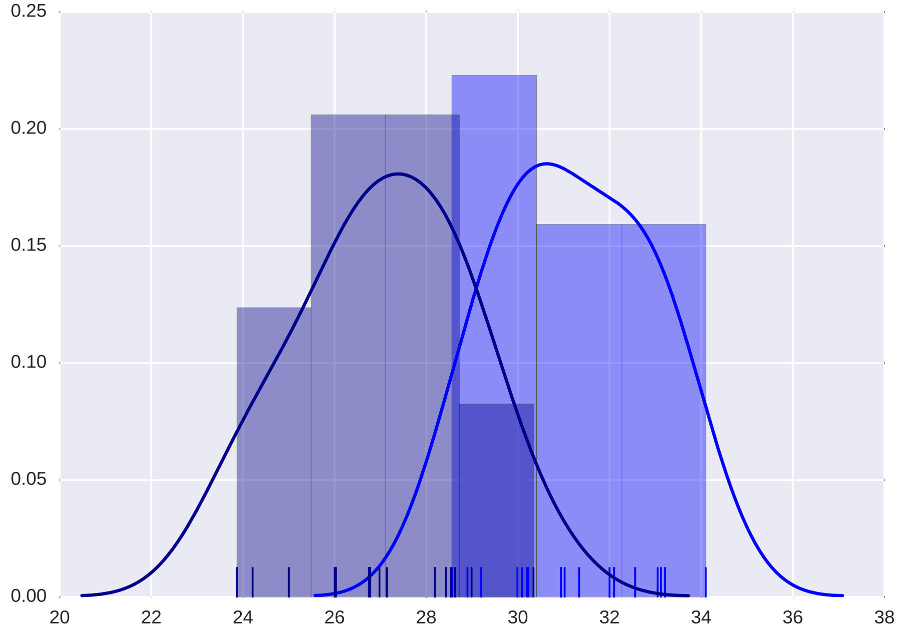

Figure 86: Significance Test Results for Reduced glutathione levels between the untreated group(GSH_U) and the group treated with *Withania somnifera*(GSH_W). As both the sets were normal, a Student's T-test was used. The p-value obtained for the test was 0.0000 and hence the difference between the data sets was considered statistically significant.

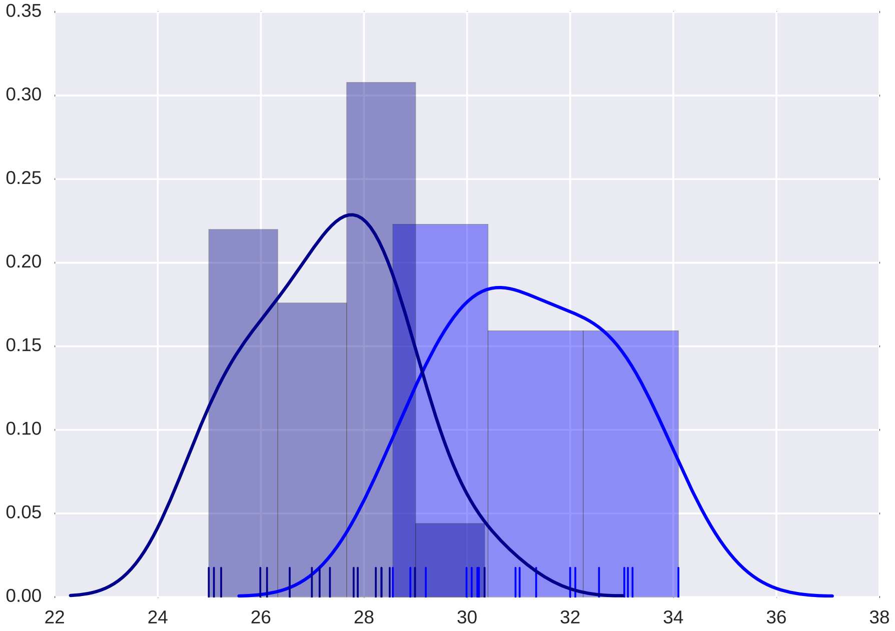

Figure 87: Significance Test Results for Reduced glutathione levels between the untreated group(GSH_U) and the group treated with both *Withania somnifera* and *Centella asiatica*(GSH_WC). As both the sets were normal, a Student's T-test was used. The p-value obtained for the test was 0.0000 and hence the difference between the data sets was considered statistically significant.

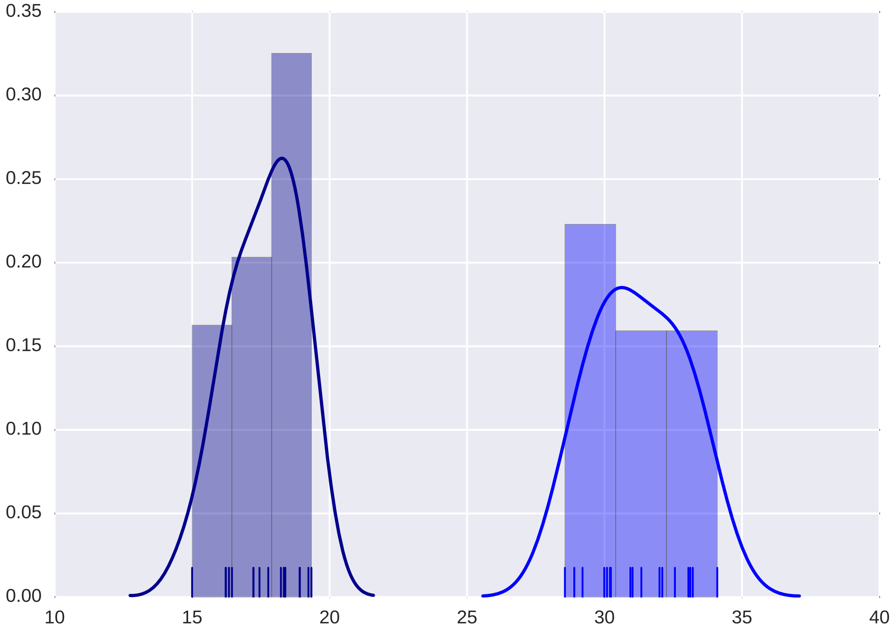

Figure 88: Significance Test Results for Reduced glutathione levels between the untreated group(GSH_U) and the MPTP disease induced and no treatment group(GSH_M). As both the sets were normal, a Student's T-test was used. The p-value obtained for the test was 0.0000 and hence the difference between the data sets was considered statistically significant.

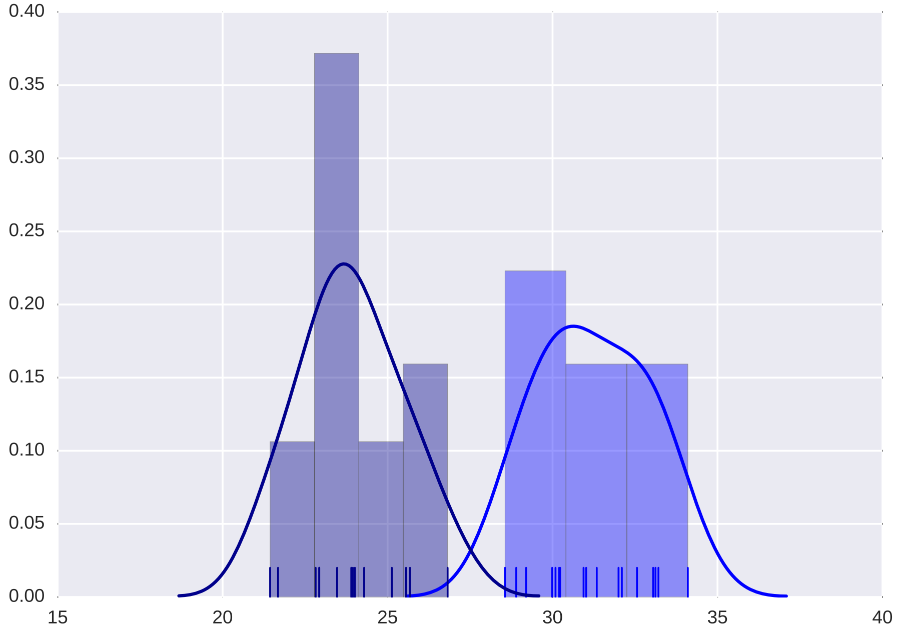

Figure 89: Significance Test Results for Reduced glutathione levels between the untreated group(GSH_U) and the MPTP disease induced and treated with *Centella asiatica* group(GSH_MC). As both the sets were normal, a Student's T-test was used. The p-value obtained for the test was 0.0000 and hence the difference between the data sets was considered statistically significant.

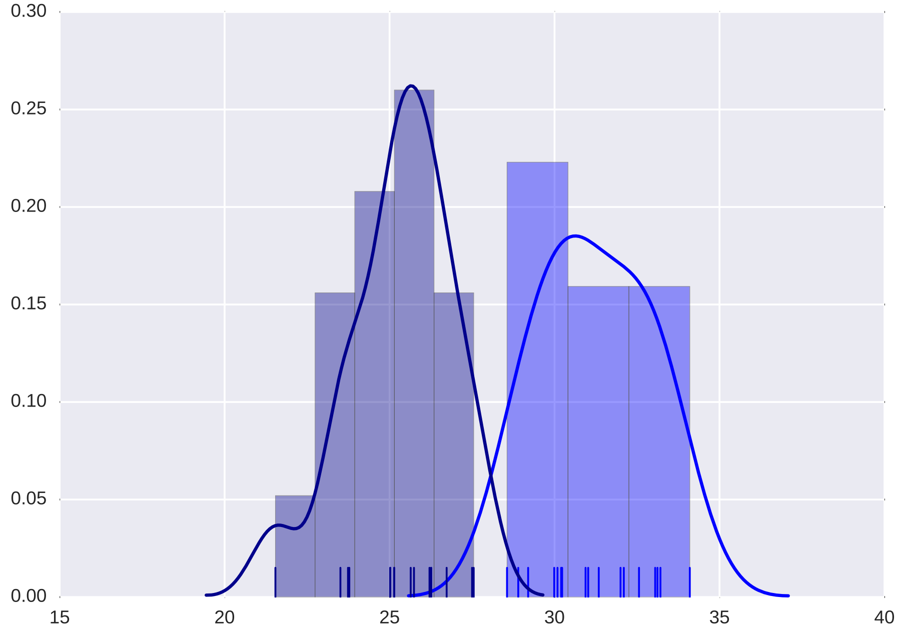

Figure 90: Significance Test Results for Reduced glutathione levels between the untreated group(GSH_U) and the MPTP disease induced and treated with *Withania somnifera* group(GSH_MW). As both the sets were normal, a Student's T-test was used. The p-value obtained for the test was 0.0000 and hence the difference between the data sets was considered statistically significant.

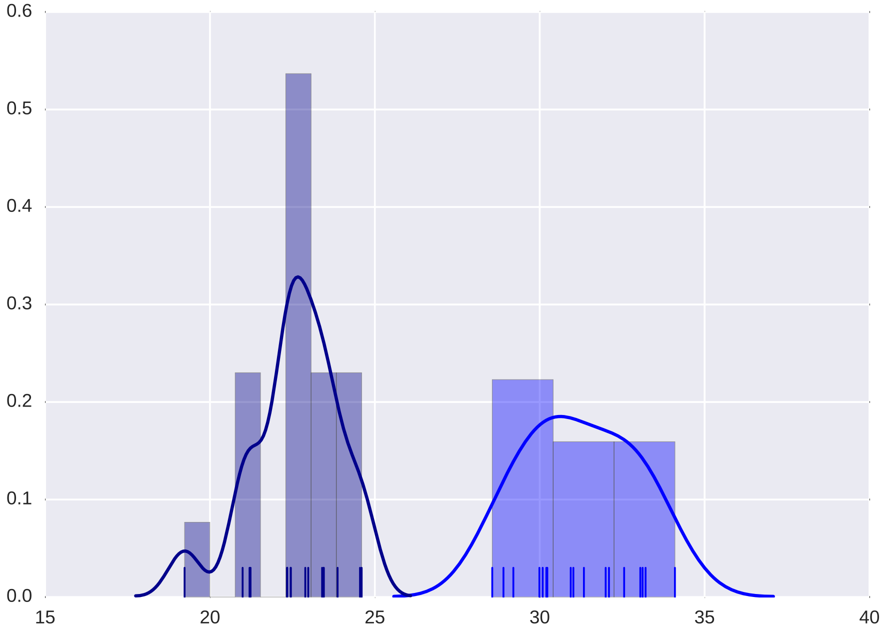

Figure 91: Significance Test Results for Reduced glutathione levels between the untreated group(GSH_U) and the MPTP disease induced and treated with both *Withania somnifera* and *Centella asiatica* group(GSH_MWC). As both the sets were normal, a Student's T-test was used. The p-value obtained for the test was 0.0000 and hence the difference between the data sets was considered statistically significant.

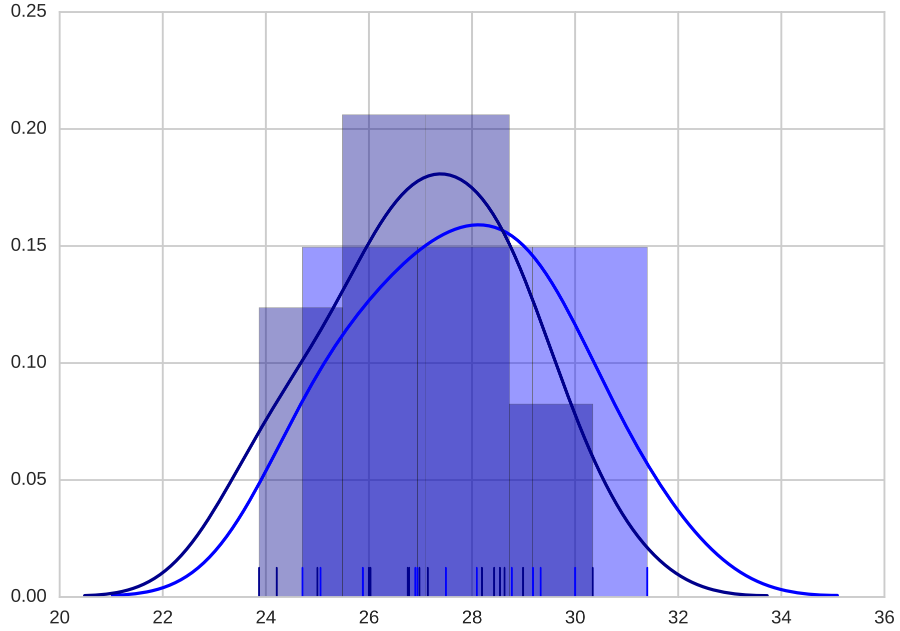

Figure 92: Significance Test Results for Reduced glutathione levels between the group treated with *Centella asiatica*(GSH_C) and the group treated with *Withania somnifera*(GSH_W). As both the sets were normal, a Student's T-test was used. The p-value obtained for the test was 0.3217 and hence the difference between the data sets was considered statistically non-significant.

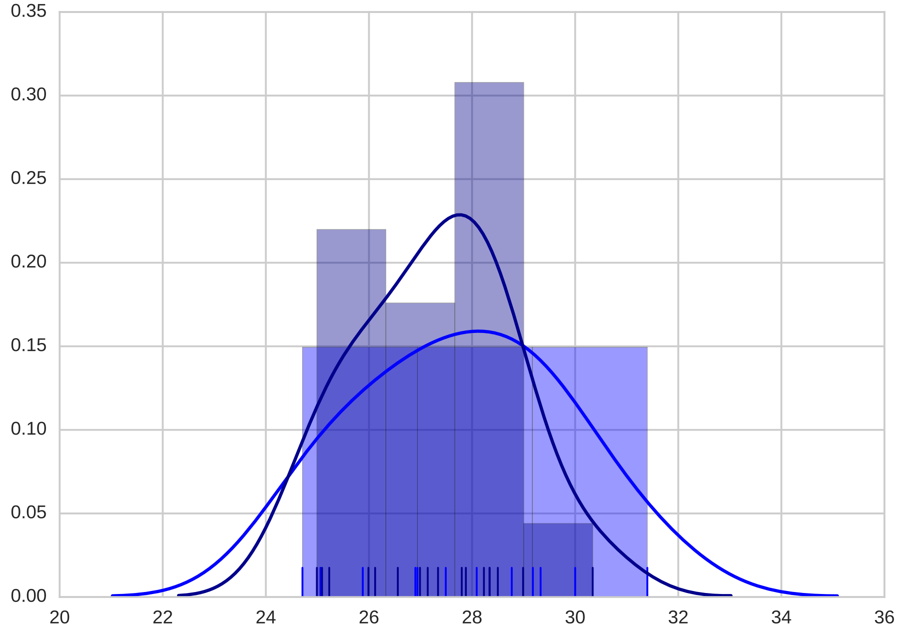

Figure 93: Significance Test Results for Reduced glutathione levels between the group treated with *Centella asiatica*(GSH_C) and the group treated with both *Withania somnifera* and *Centella asiatica*(GSH_WC). As both the sets were normal, a Student's T-test was used. The p-value obtained for the test was 0.4273 and hence the difference between the data sets was considered statistically non-significant.

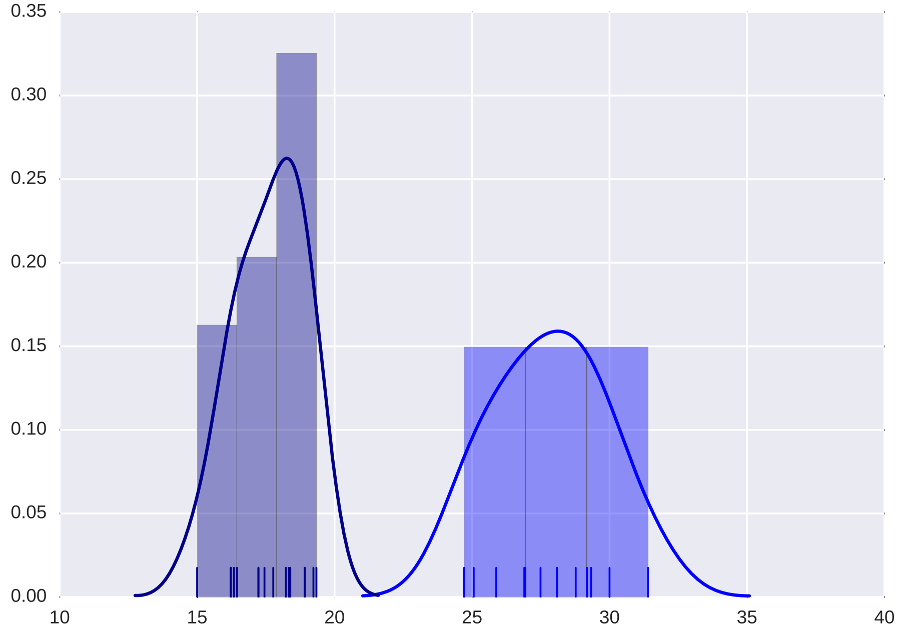

Figure 94: Significance Test Results for Reduced glutathione levels between the group treated with *Centella asiatica*(GSH_C) and the MPTP disease induced and no treatment group(GSH_M). As both the sets were normal, a Student's T-test was used. The p-value obtained for the test was 0.0000 and hence the difference between the data sets was considered statistically significant.

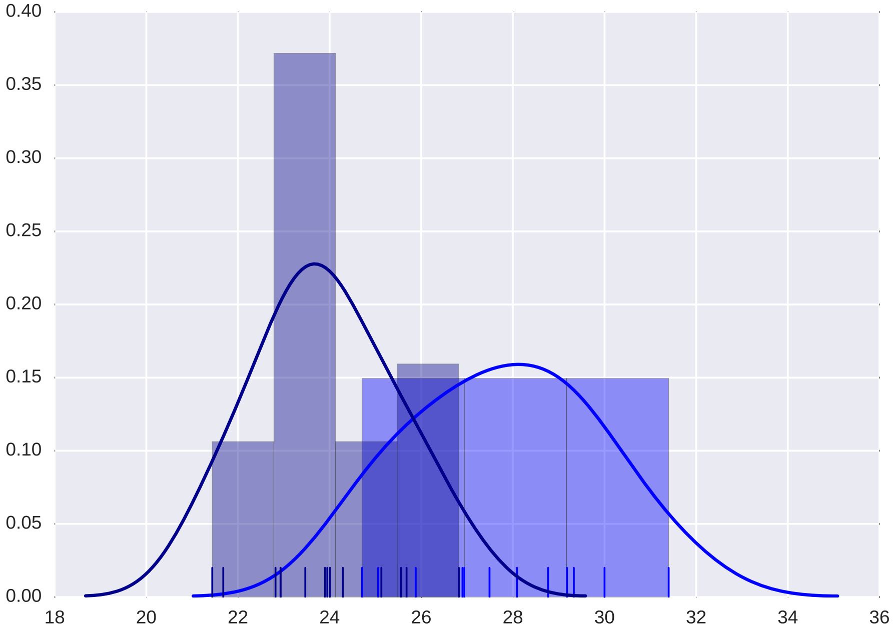

Figure 95: Significance Test Results for Reduced glutathione levels between the group treated with *Centella asiatica*(GSH_C) and the MPTP disease induced and treated with *Centella asiatica* group(GSH_MC). As both the sets were normal, a Student's T-test was used. The p-value obtained for the test was 0.0000 and hence the difference between the data sets was considered statistically significant.

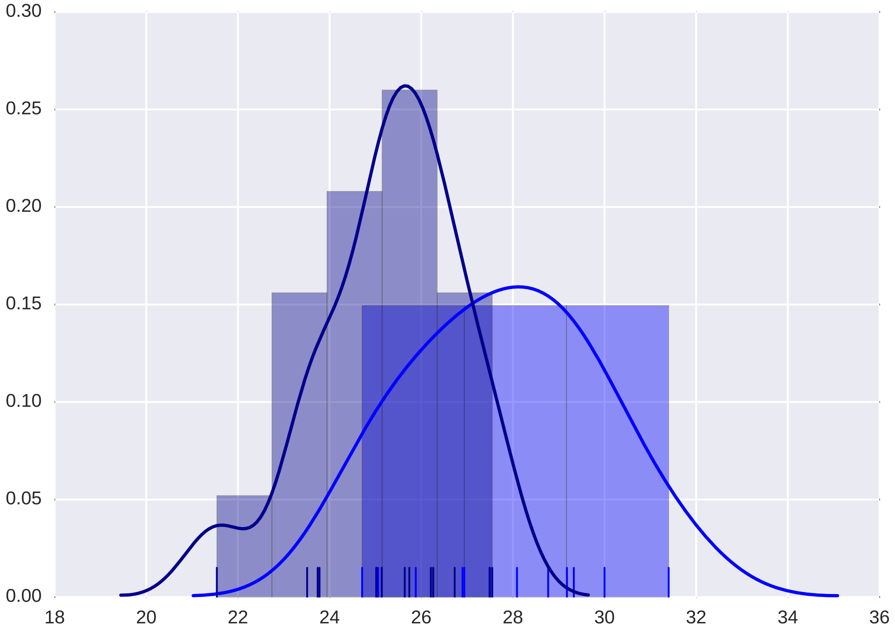

Figure 96: Significance Test Results for Reduced glutathione levels between the group treated with *Centella asiatica*(GSH_C) and the MPTP disease induced and treated with *Withania somnifera* group(GSH_MW). As both the sets were normal, a Student's T-test was used. The p-value obtained for the test was 0.0010 and hence the difference between the data sets was considered statistically significant.

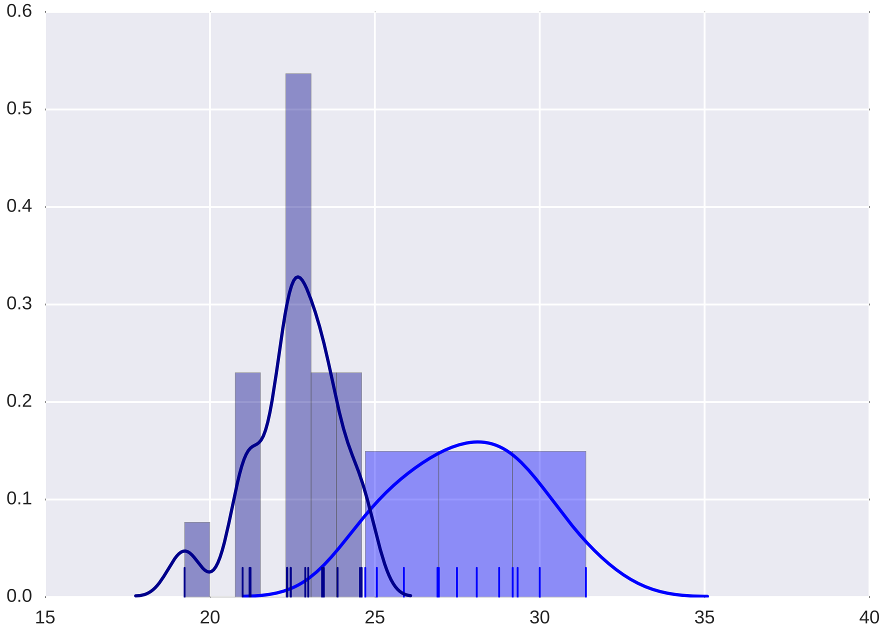

Figure 97: Significance Test Results for Reduced glutathione levels between the group treated with *Centella asiatica*(GSH_C) and the MPTP disease induced and treated with both *Withania somnifera* and *Centella asiatica* group(GSH_MWC). As both the sets were normal, a Student's T-test was used. The p-value obtained for the test was 0.0000 and hence the difference between the data sets was considered statistically significant.

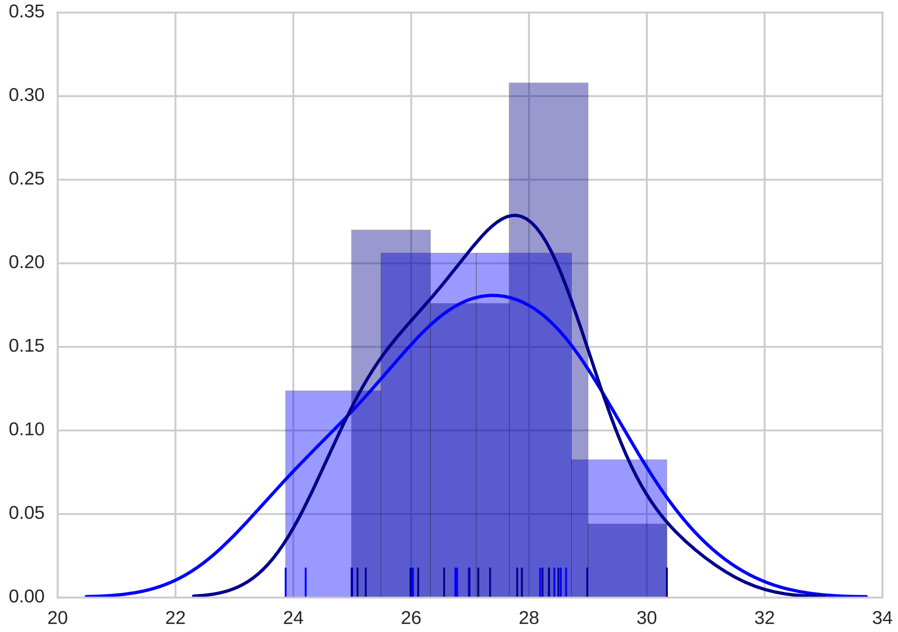

Figure 98: Significance Test Results for Reduced glutathione levels between the group treated with *Withania somnifera*(GSH_W) and the group treated with both *Withania somnifera* and *Centella asiatica*(GSH_WC). As both the sets were normal, a Student's T-test was used. The p-value obtained for the test was 0.7012 and hence the difference between the data sets was considered statistically non-significant.

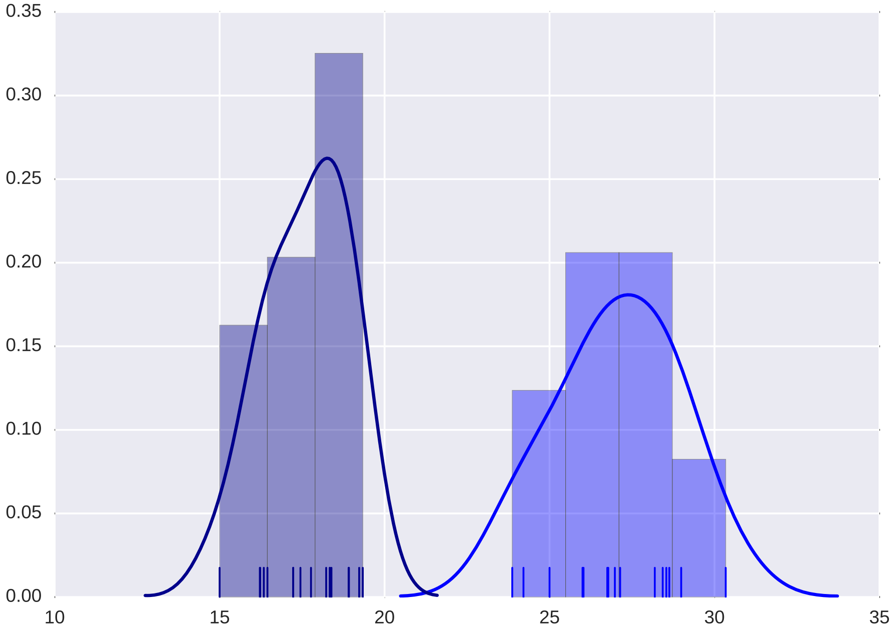

Figure 99: Significance Test Results for Reduced glutathione levels between the group treated with *Withania somnifera*(GSH_W) and the MPTP disease induced and no treatment group(GSH_M). As both the sets were normal, a Student's T-test was used. The p-value obtained for the test was 0.0000 and hence the difference between the data sets was considered statistically significant.

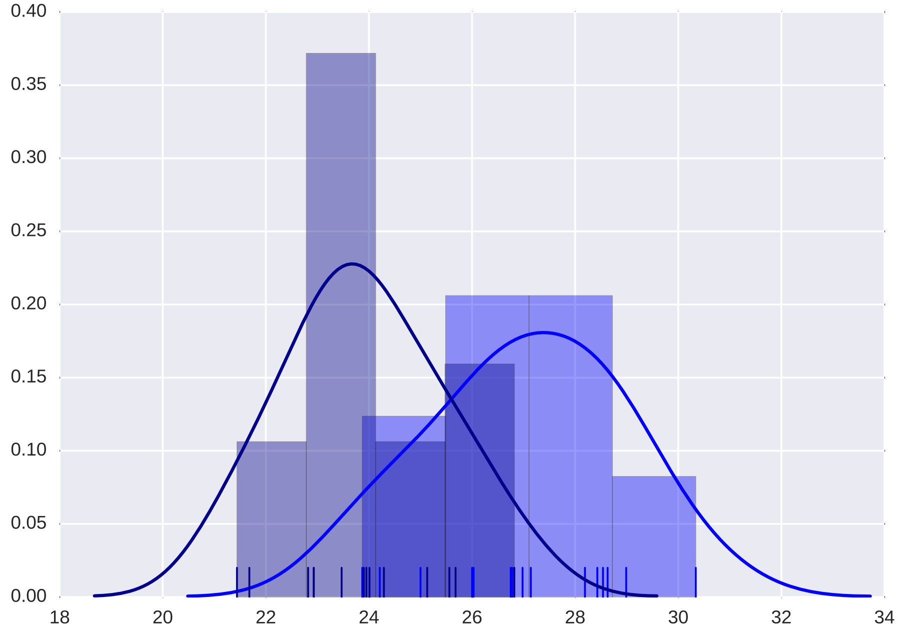

Figure 100: Significance Test Results for Reduced glutathione levels between the group treated with *Withania somnifera*(GSH_W) and the MPTP disease induced and treated with *Centella asiatica* group(GSH_MC). As both the sets were normal, a Student's T-test was used. The p-value obtained for the test was 0.0000 and hence the difference between the data sets was considered statistically significant.

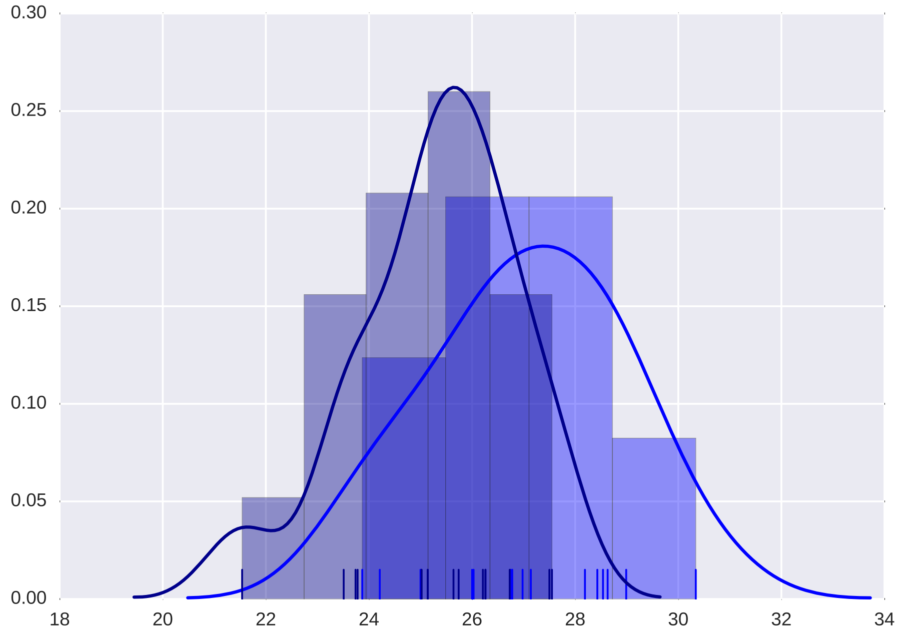

Figure 101: Significance Test Results for Reduced glutathione levels between the group treated with *Withania somnifera*(GSH_W) and the MPTP disease induced and treated with *Withania somnifera* group(GSH_MW). As both the sets were normal, a Student's T-test was used. The p-value obtained for the test was 0.0076 and hence the difference between the data sets was considered statistically significant.

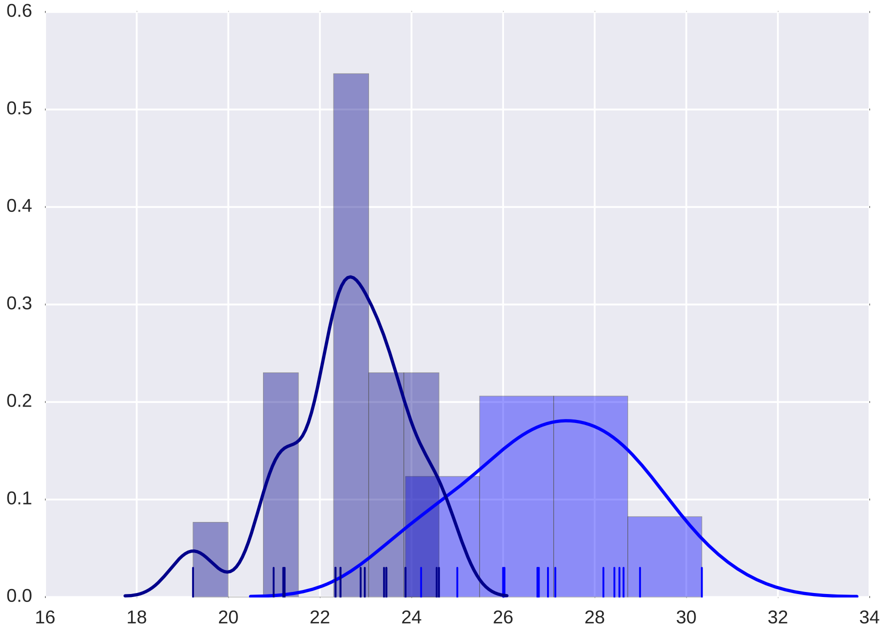

Figure 102: Significance Test Results for Reduced glutathione levels between the group treated with *Withania somnifera*(GSH_W) and the MPTP disease induced and treated with both *Withania somnifera* and *Centella asiatica* group(GSH_MWC). As both the sets were normal, a Student's T-test was used. The p-value obtained for the test was 0.0000 and hence the difference between the data sets was considered statistically significant.

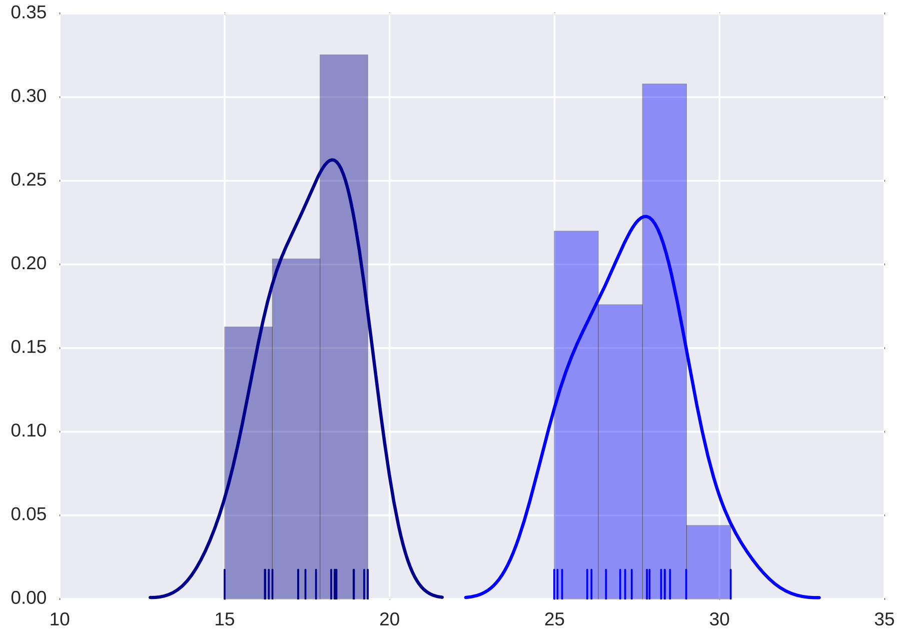

Figure 103: Significance Test Results for Reduced glutathione levels between the group treated with both *Withania somnifera* and *Centella asiatica*(GSH_WC) and the MPTP disease induced and no treatment group(GSH_M). As both the sets were normal, a Student's T-test was used. The p-value obtained for the test was 0.0000 and hence the difference between the data sets was considered statistically significant.

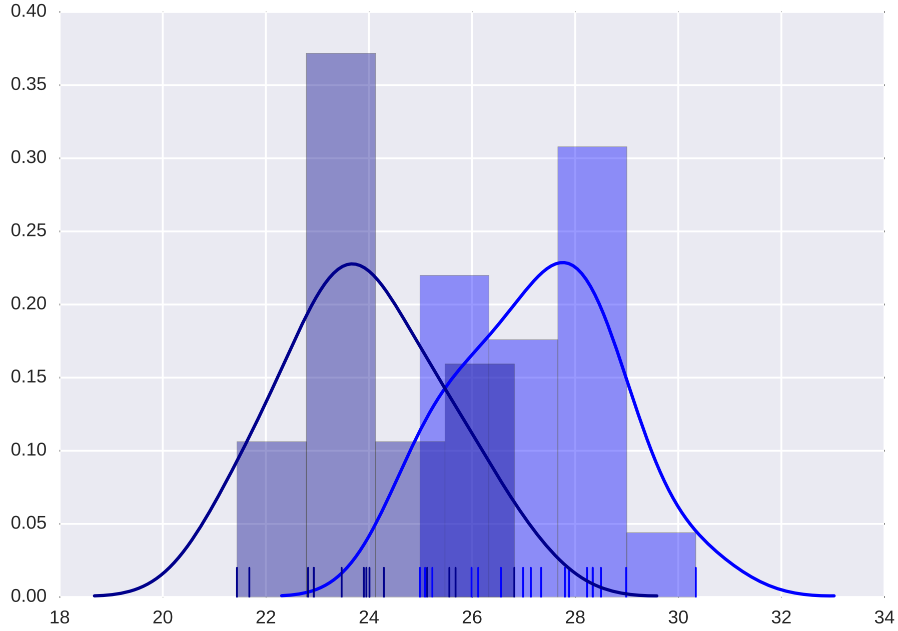

Figure 104: Significance Test Results for Reduced glutathione levels between the group treated with both *Withania somnifera* and *Centella asiatica*(GSH_WC) and the MPTP disease induced and treated with *Centella asiatica* group(GSH_MC). As both the sets were normal, a Student's T-test was used. The p-value obtained for the test was 0.0000 and hence the difference between the data sets was considered statistically significant.

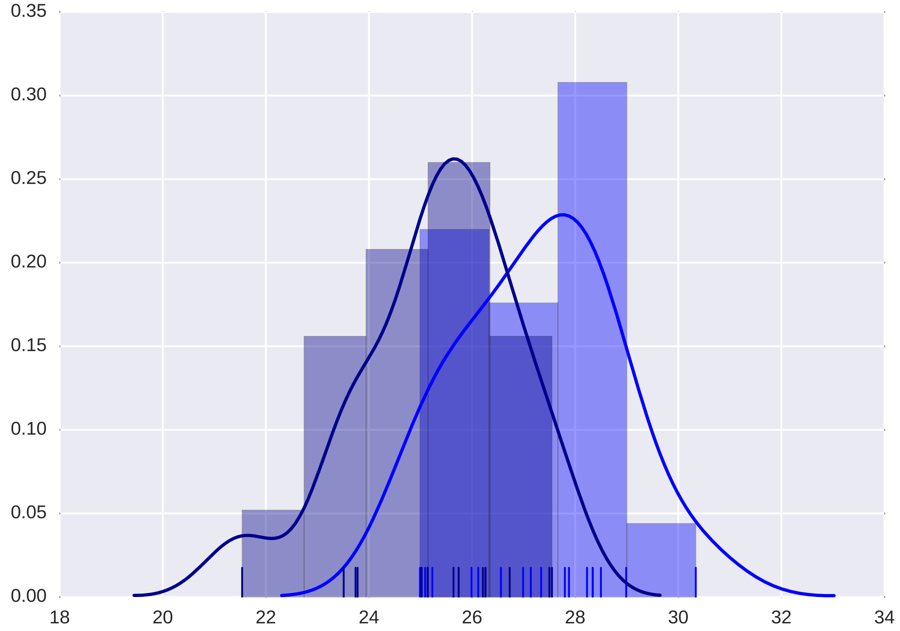

Figure 105: Significance Test Results for Reduced glutathione levels between the group treated with both *Withania somnifera* and *Centella asiatica*(GSH_WC) and the MPTP disease induced and treated with *Withania somnifera* group(GSH_MW). As both the sets were normal, a Student's T-test was used. The p-value obtained for the test was 0.0008 and hence the difference between the data sets was considered statistically significant.

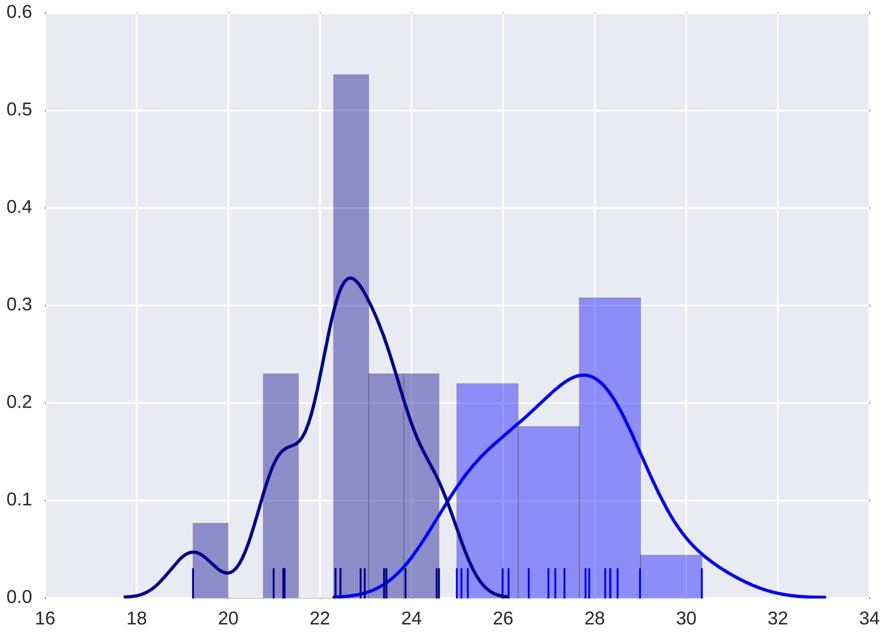

Figure 106: Significance Test Results for Reduced glutathione levels between the group treated with both *Withania somnifera* and *Centella asiatica*(GSH_WC) and the MPTP disease induced and treated with both *Withania somnifera* and *Centella asiatica* group(GSH_MWC). As both the sets were normal, a Student's T-test was used. The p-value obtained for the test was 0.0000 and hence the difference between the data sets was considered statistically significant.

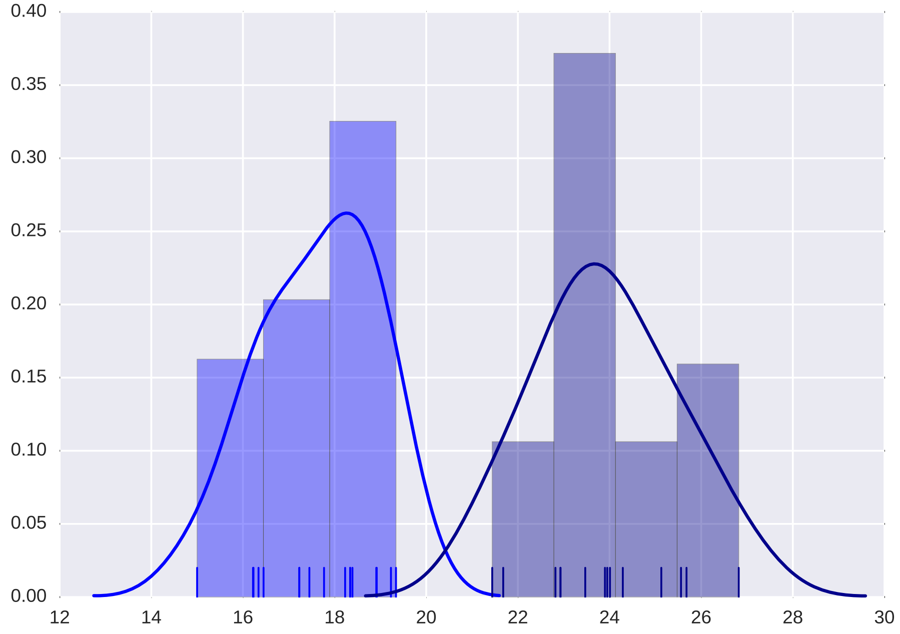

Figure 107: Significance Test Results for Reduced glutathione levels between the MPTP disease induced and no treatment group(GSH_M) and the MPTP disease induced and treated with *Centella asiatica* group(GSH_MC). As both the sets were normal, a Student's T-test was used. The p-value obtained for the test was 0.0000 and hence the difference between the data sets was considered statistically significant.

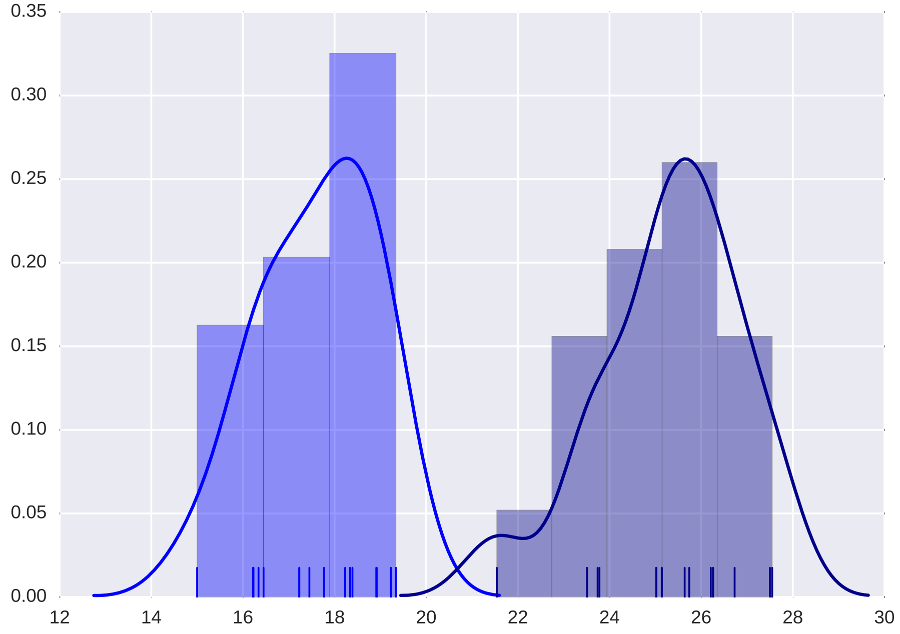

Figure 108: Significance Test Results for Reduced glutathione levels between the MPTP disease induced and no treatment group(GSH_M) and the MPTP disease induced and treated with *Withania somnifera* group(GSH_MW). As both the sets were normal, a Student's T-test was used. The p-value obtained for the test was 0.0000 and hence the difference between the data sets was considered statistically significant.

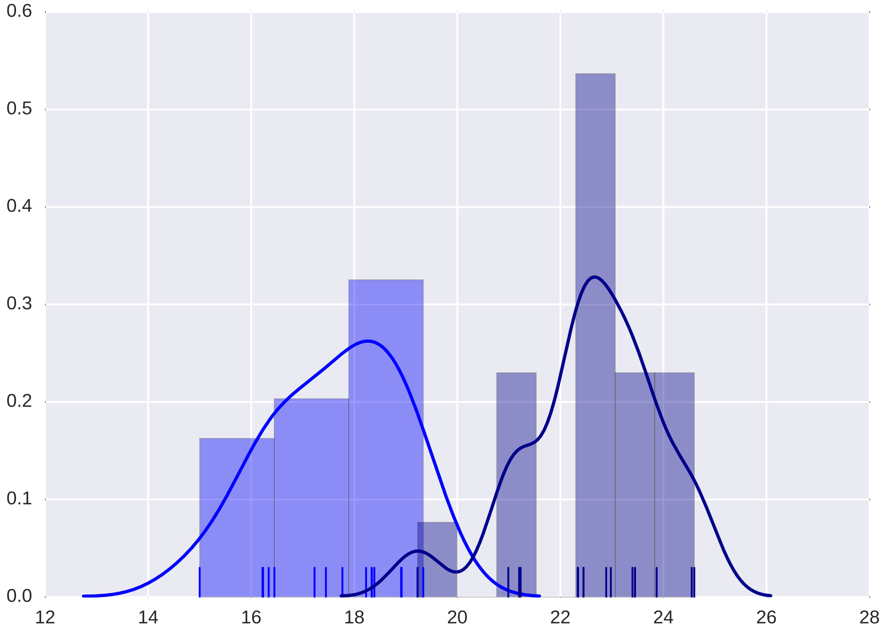

Figure 109: Significance Test Results for Reduced glutathione levels between the MPTP disease induced and no treatment group(GSH_M) and the MPTP disease induced and treated with both *Withania somnifera* and *Centella asiatica* group(GSH_MWC). As both the sets were normal, a Student's T-test was used. The p-value obtained for the test was 0.0000 and hence the difference between the data sets was considered statistically significant.

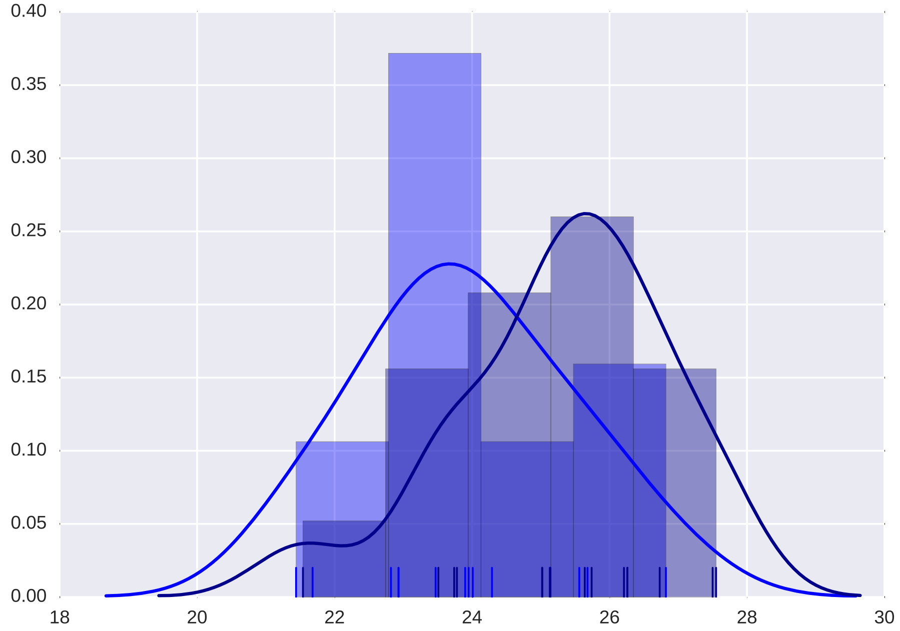

Figure 110: Significance Test Results for Reduced glutathione levels between the MPTP disease induced and treated with *Centella asiatica* group(GSH_MC) and the MPTP disease induced and treated with *Withania somnifera* group(GSH_MW). As both the sets were normal, a Student's T-test was used. The p-value obtained for the test was 0.0204 and hence the difference between the data sets was considered statistically significant.

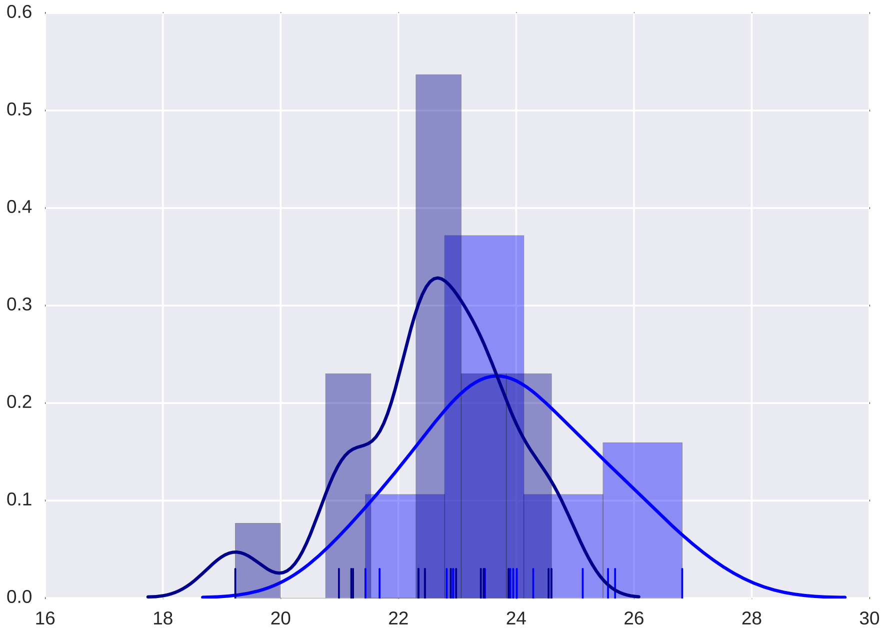

Figure 111: Significance Test Results for Reduced glutathione levels between the MPTP disease induced and treated with *Centella asiatica* group(GSH_MC) and the MPTP disease induced and treated with both *Withania somnifera* and *Centella asiatica* group(GSH_MWC). As both the sets were normal, a Student's T-test was used. The p-value obtained for the test was 0.0162 and hence the difference between the data sets was considered statistically significant.

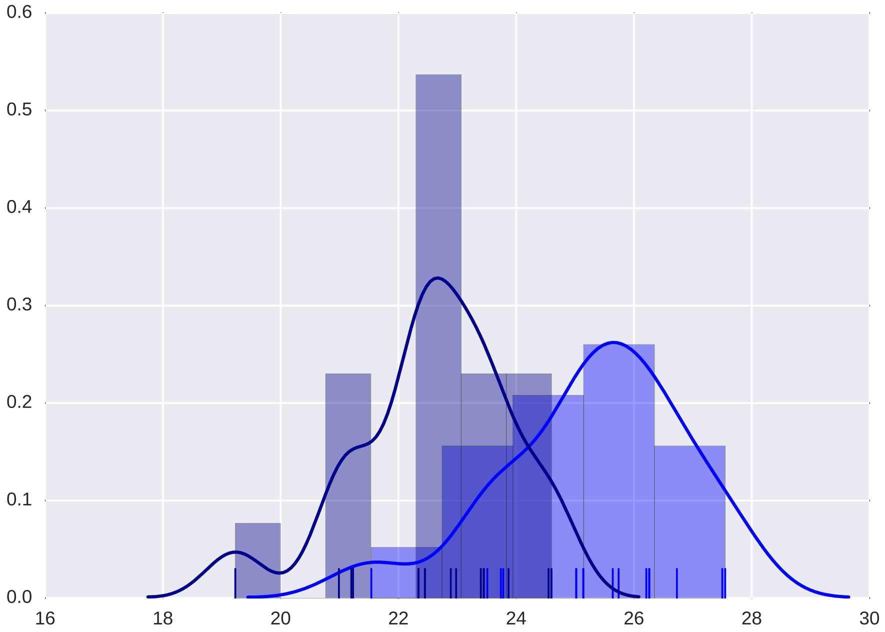

Figure 112: Significance Test Results for Reduced glutathione levels between the MPTP disease induced and treated with *Withania somnifera* group(GSH_MW) and the MPTP disease induced and treated with both *Withania somnifera* and *Centella asiatica* group(GSH_MWC). As both the sets were normal, a Student's T-test was used. The p-value obtained for the test was 0.0000 and hence the difference between the data sets was considered statistically significant.

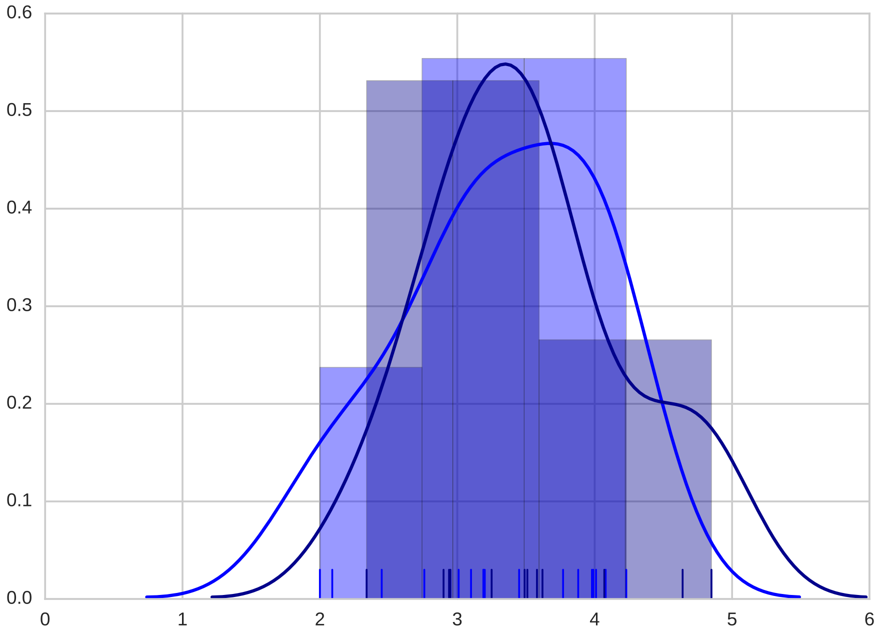

Figure 113: Significance Test Results for Lipid peroxidation levels between the untreated group(LPO_U) and the group treated with *Centella asiatica*(LPO_C). As both the sets were normal, a Student's T-test was used. The p-value obtained for the test was 0.4731 and hence the difference between the data sets was considered statistically non-significant.

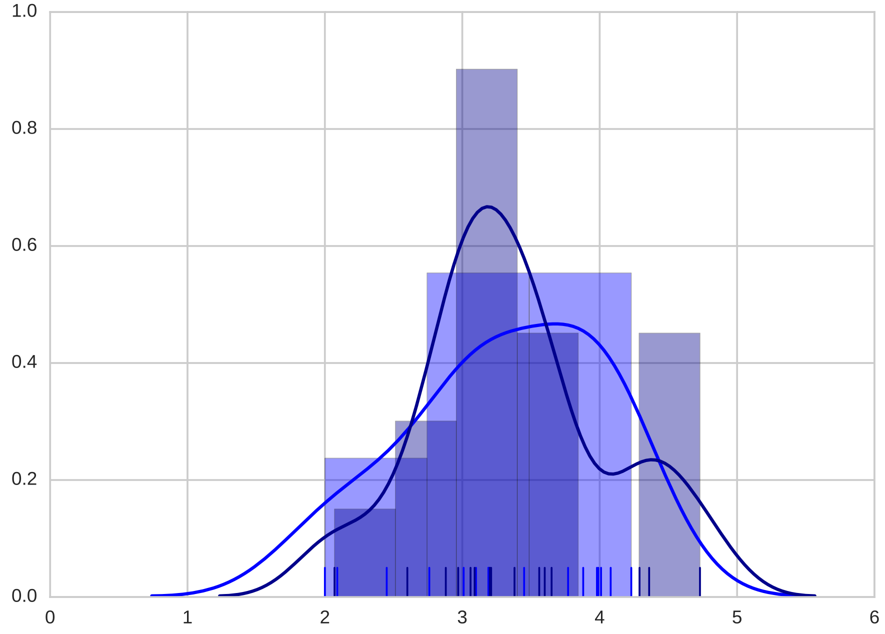

Figure 114: Significance Test Results for Lipid peroxidation levels between the untreated group(LPO_U) and the group treated with *Withania somnifera*(LPO_W). As both the sets were normal, a Student's T-test was used. The p-value obtained for the test was 0.8090 and hence the difference between the data sets was considered statistically non-significant.

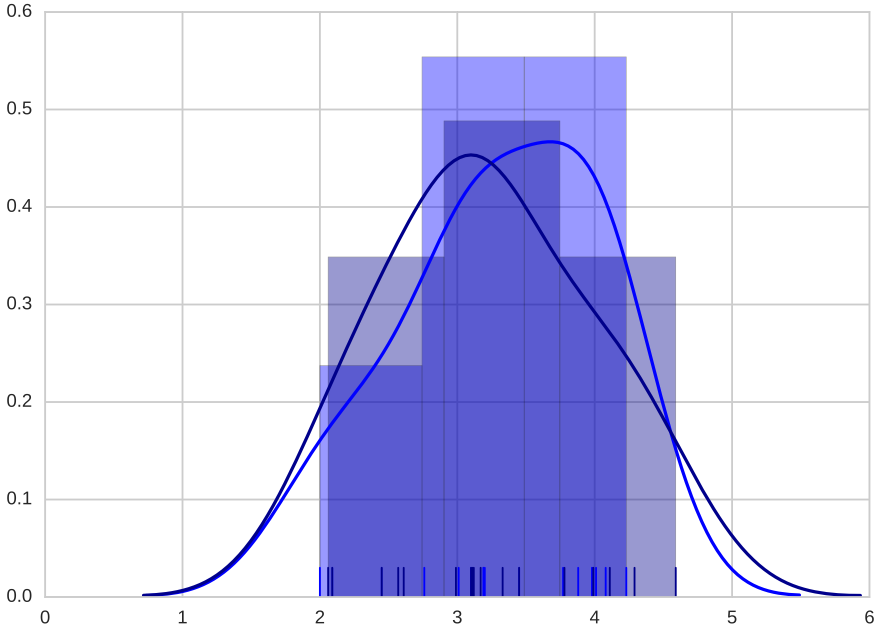

Figure 115: Significance Test Results for Lipid peroxidation levels between the untreated group(LPO_U) and the group treated with both *Withania somnifera* and *Centella asiatica*(LPO_WC). As both the sets were normal, a Student's T-test was used. The p-value obtained for the test was 0.7119 and hence the difference between the data sets was considered statistically non-significant.

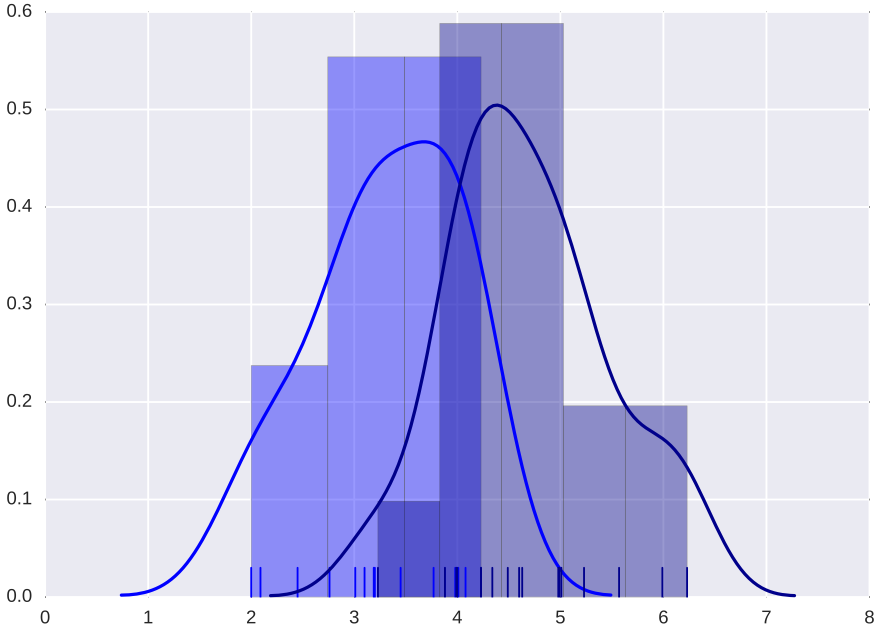

Figure 116: Significance Test Results for Lipid peroxidation levels between the untreated group(LPO_U) and the MPTP disease induced and no treatment group(LPO_M). As both the sets were normal, a Student's T-test was used. The p-value obtained for the test was 0.0000 and hence the difference between the data sets was considered statistically significant.

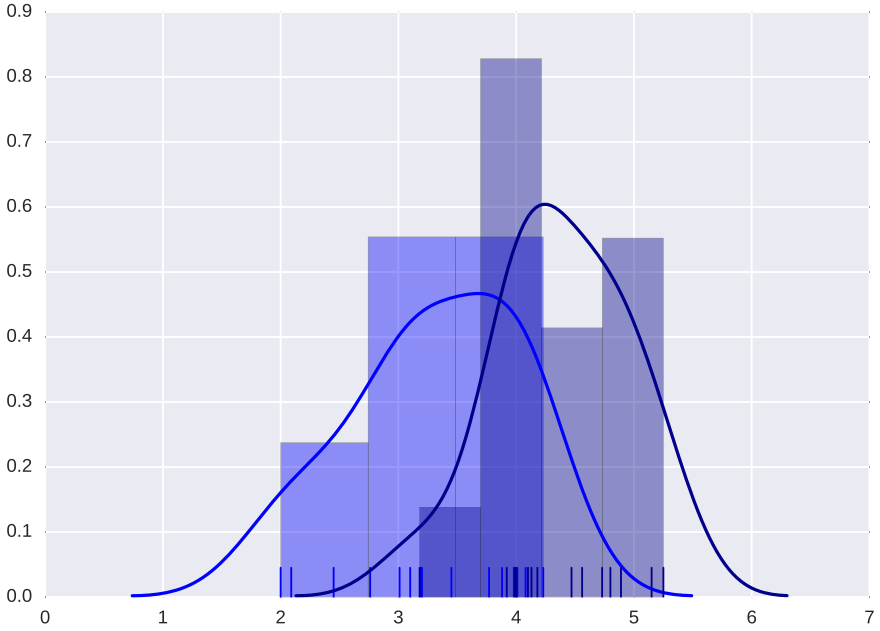

Figure 117: Significance Test Results for Lipid peroxidation levels between the untreated group(LPO_U) and the MPTP disease induced and treated with *Centella asiatica* group(LPO_MC). As both the sets were normal, a Student's T-test was used. The p-value obtained for the test was 0.0001 and hence the difference between the data sets was considered statistically significant.

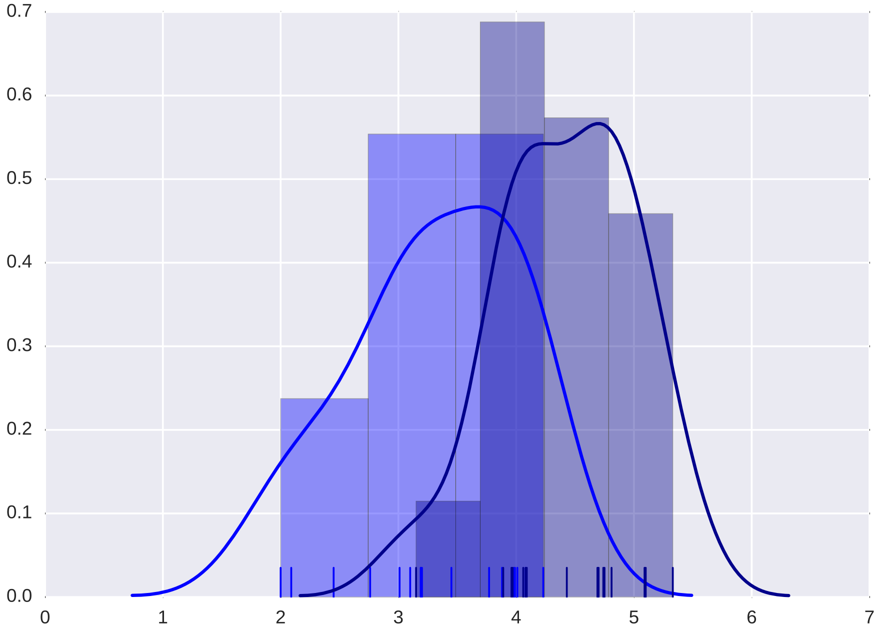

Figure 118: Significance Test Results for Lipid peroxidation levels between the untreated group(LPO_U) and the MPTP disease induced and treated with *Withania somnifera* group(LPO_MW). As both the sets were normal, a Student's T-test was used. The p-value obtained for the test was 0.0000 and hence the difference between the data sets was considered statistically significant.

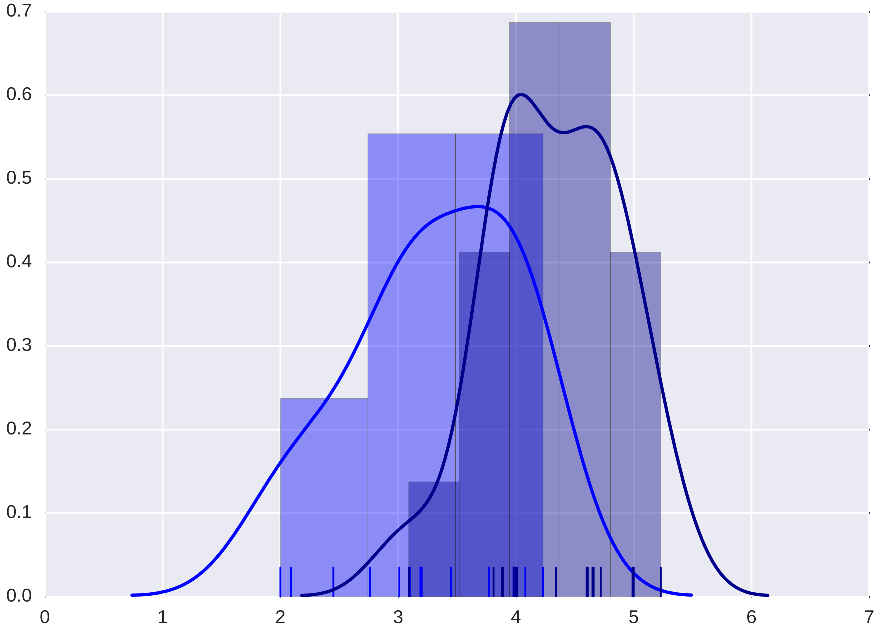

Figure 119: Significance Test Results for Lipid peroxidation levels between the untreated group(LPO_U) and the MPTP disease induced and treated with both *Withania somnifera* and *Centella asiatica* group(LPO_MWC). As both the sets were normal, a Student's T-test was used. The p-value obtained for the test was 0.0001 and hence the difference between the data sets was considered statistically significant.

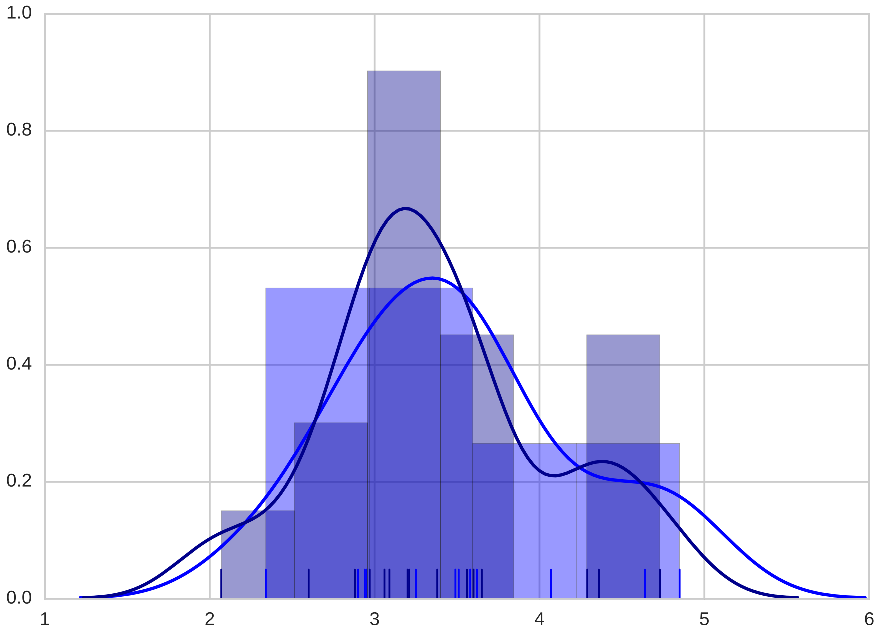

Figure 120: Significance Test Results for Lipid peroxidation levels between the group treated with *Centella asiatica*(LPO_C) and the group treated with *Withania somnifera*(LPO_W). As both the sets were normal, a Student's T-test was used. The p-value obtained for the test was 0.6278 and hence the difference between the data sets was considered statistically non-significant.

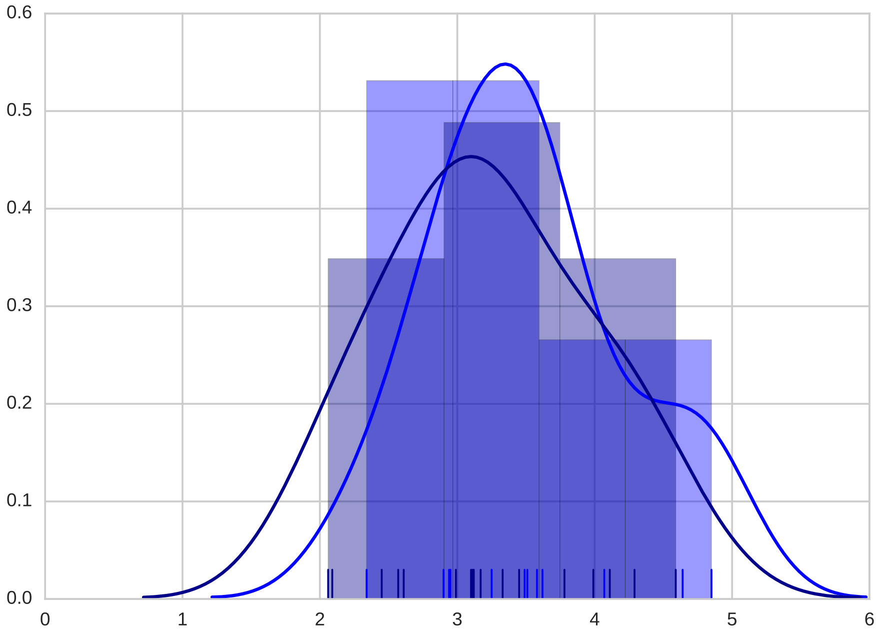

Figure 121: Significance Test Results for Lipid peroxidation levels between the group treated with *Centella asiatica*(LPO_C) and the group treated with both *Withania somnifera* and *Centella asiatica*(LPO_WC). As both the sets were normal, a Student's T-test was used. The p-value obtained for the test was 0.3111 and hence the difference between the data sets was considered statistically non-significant.

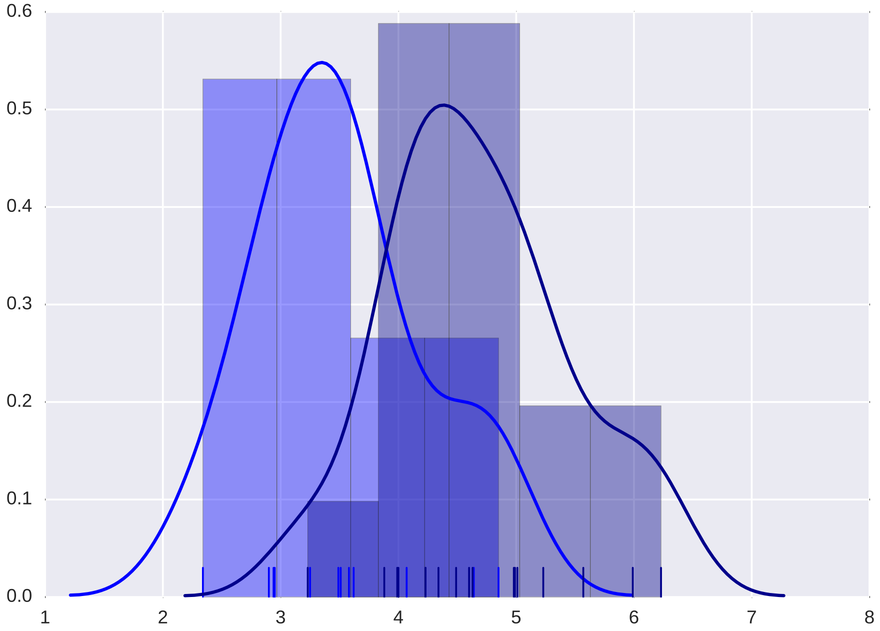

Figure 122: Significance Test Results for Lipid peroxidation levels between the group treated with *Centella asiatica*(LPO_C) and the MPTP disease induced and no treatment group(LPO_M). As both the sets were normal, a Student's T-test was used. The p-value obtained for the test was 0.0004 and hence the difference between the data sets was considered statistically significant.

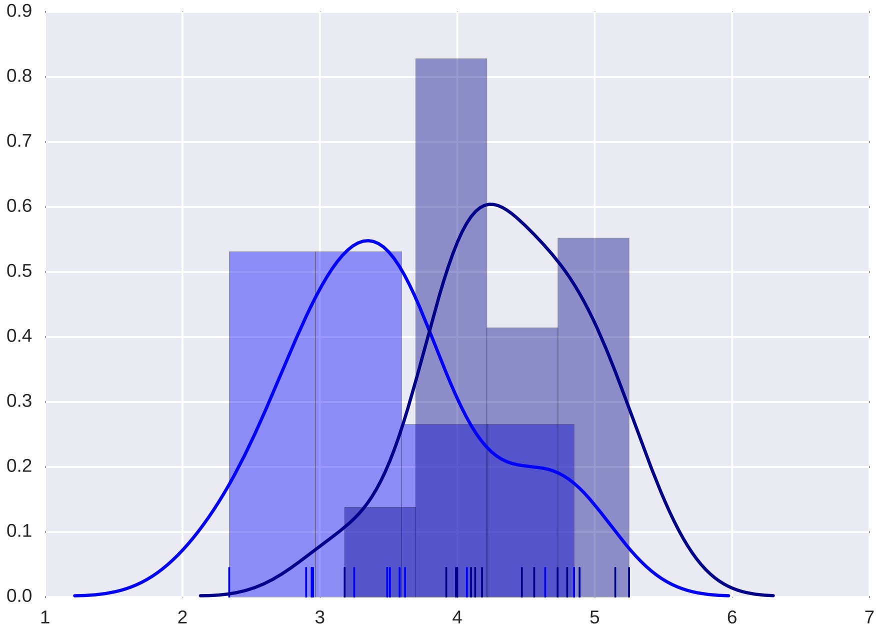

Figure 123: Significance Test Results for Lipid peroxidation levels between the group treated with *Centella asiatica*(LPO_C) and the MPTP disease induced and treated with *Centella asiatica* group(LPO_MC). As both the sets were normal, a Student's T-test was used. The p-value obtained for the test was 0.0021 and hence the difference between the data sets was considered statistically significant.

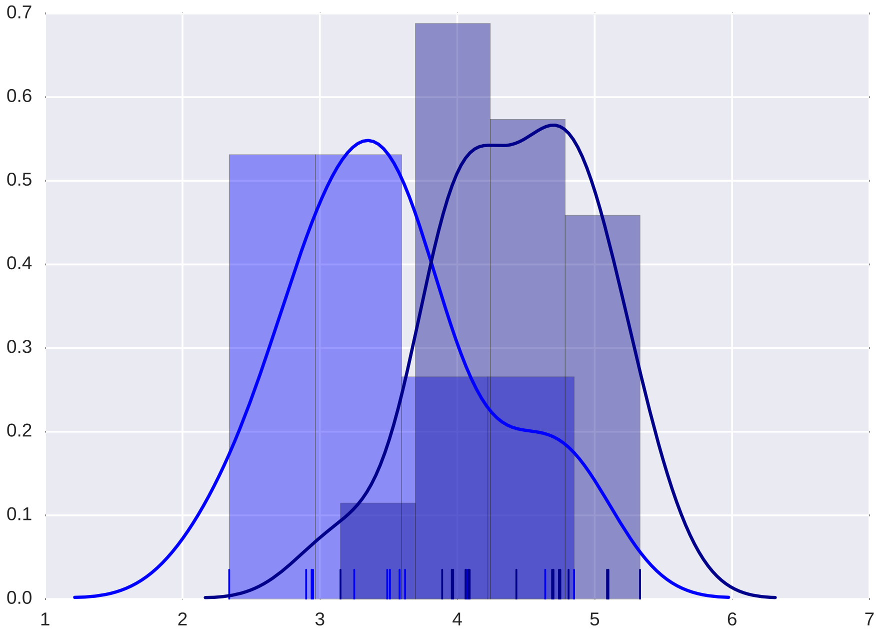

Figure 124: Significance Test Results for Lipid peroxidation levels between the group treated with *Centella asiatica*(LPO_C) and the MPTP disease induced and treated with *Withania somnifera* group(LPO_MW). As both the sets were normal, a Student's T-test was used. The p-value obtained for the test was 0.0009 and hence the difference between the data sets was considered statistically significant.

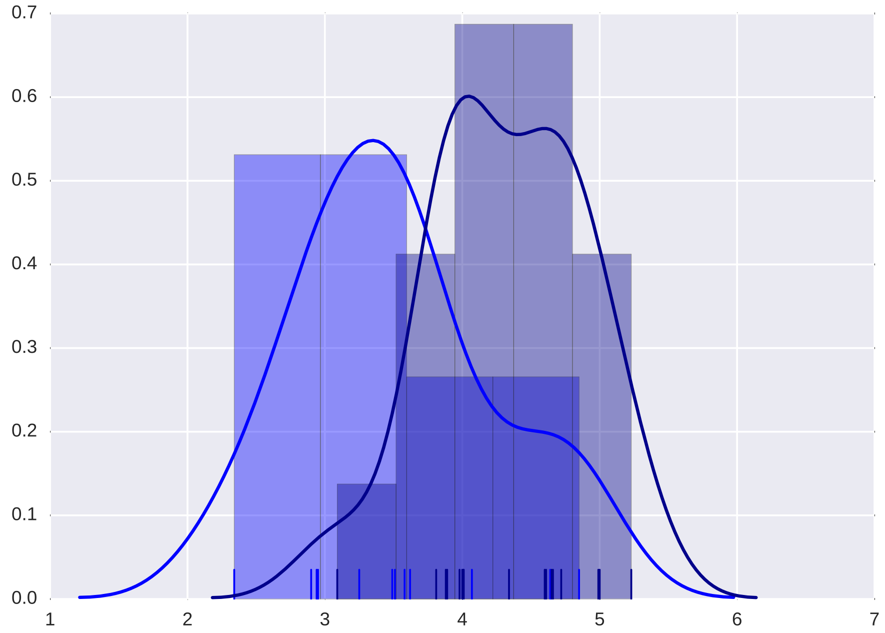

Figure 125: Significance Test Results for Lipid peroxidation levels between the group treated with *Centella asiatica*(LPO_C) and the MPTP disease induced and treated with both *Withania somnifera* and *Centella asiatica* group(LPO_MWC). As both the sets were normal, a Student's T-test was used. The p-value obtained for the test was 0.0020 and hence the difference between the data sets was considered statistically significant.

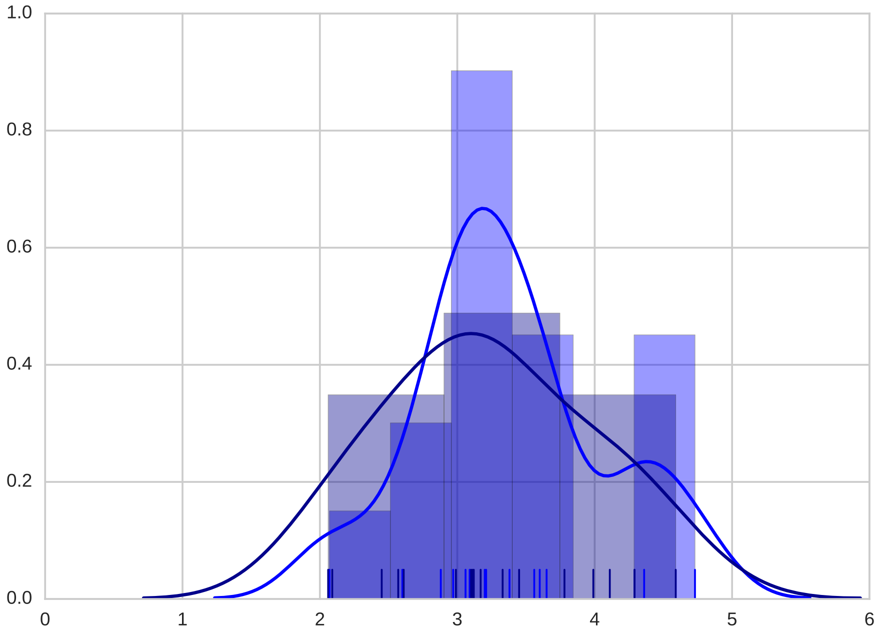

Figure 126: Significance Test Results for Lipid peroxidation levels between the group treated with *Withania somnifera*(LPO_W) and the group treated with both *Withania somnifera* and *Centella asiatica*(LPO_WC). As both the sets were normal, a Student's T-test was used. The p-value obtained for the test was 0.5554 and hence the difference between the data sets was considered statistically non-significant.

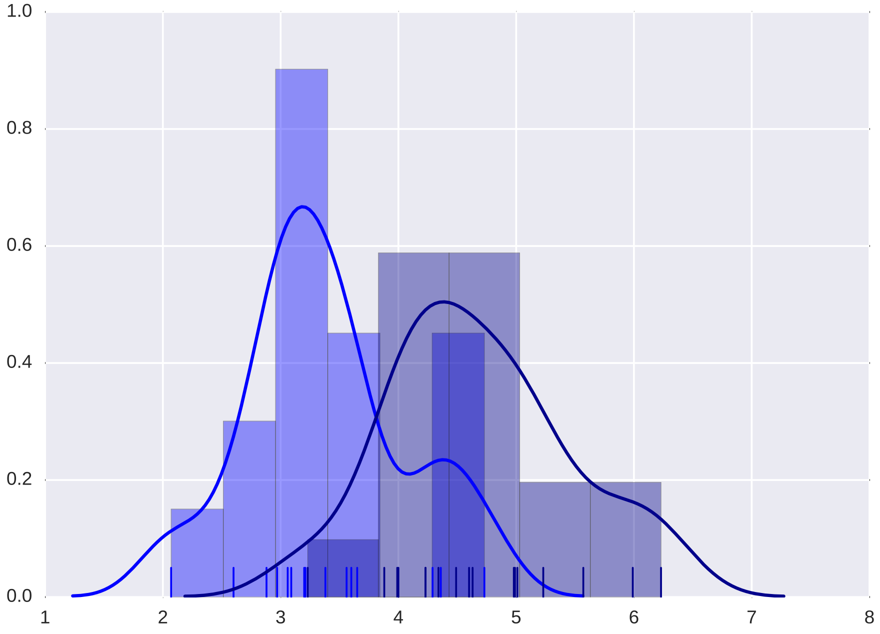

Figure 127: Significance Test Results for Lipid peroxidation levels between the group treated with *Withania somnifera*(LPO_W) and the MPTP disease induced and no treatment group(LPO_M). As both the sets were normal, a Student's T-test was used. The p-value obtained for the test was 0.0000 and hence the difference between the data sets was considered statistically significant.

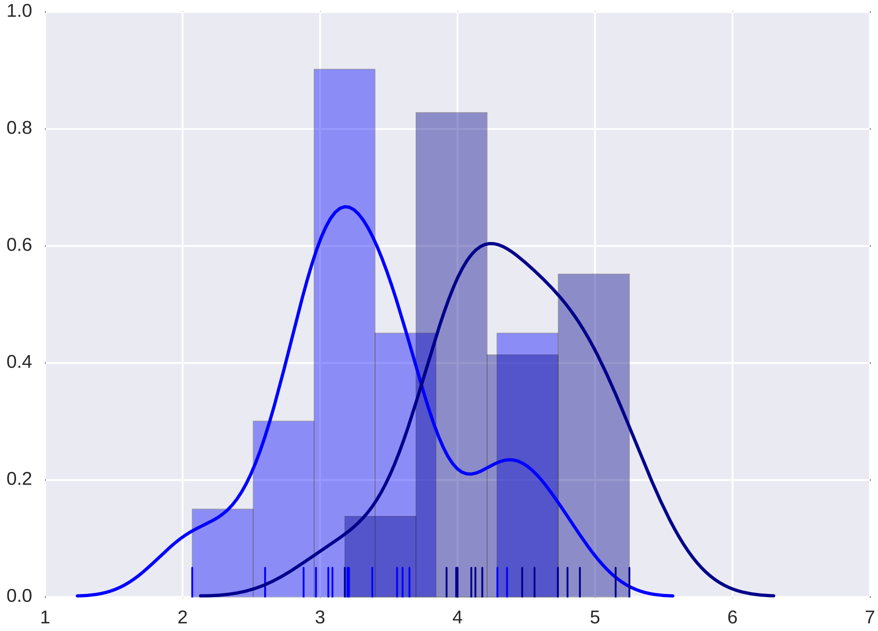

Figure 128: Significance Test Results for Lipid peroxidation levels between the group treated with *Withania somnifera*(LPO_W) and the MPTP disease induced and treated with *Centella asiatica* group(LPO_MC). As both the sets were normal, a Student's T-test was used. The p-value obtained for the test was 0.0002 and hence the difference between the data sets was considered statistically significant.

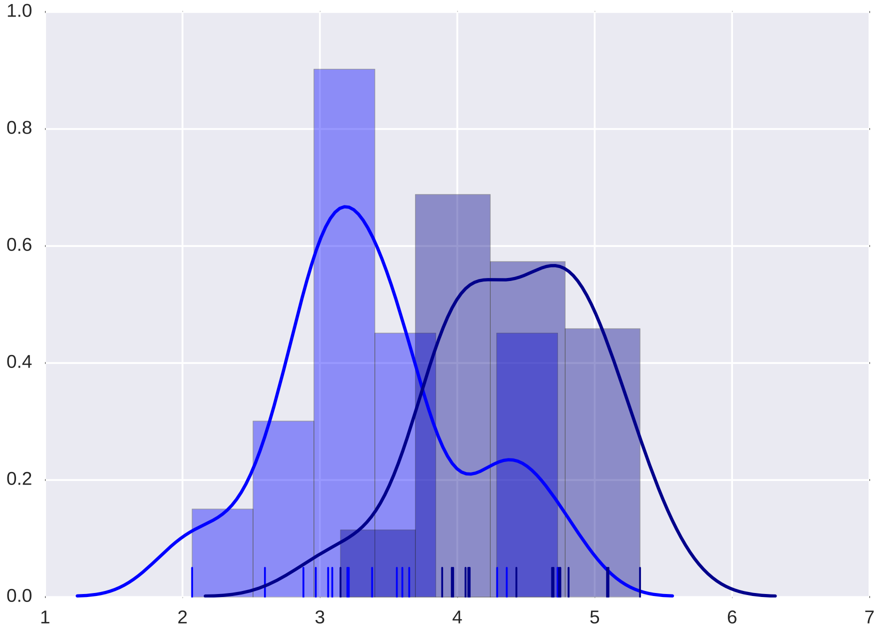

Figure 129: Significance Test Results for Lipid peroxidation levels between the group treated with *Withania somnifera*(LPO_W) and the MPTP disease induced and treated with *Withania somnifera* group(LPO_MW). As both the sets were normal, a Student's T-test was used. The p-value obtained for the test was 0.0001 and hence the difference between the data sets was considered statistically significant.

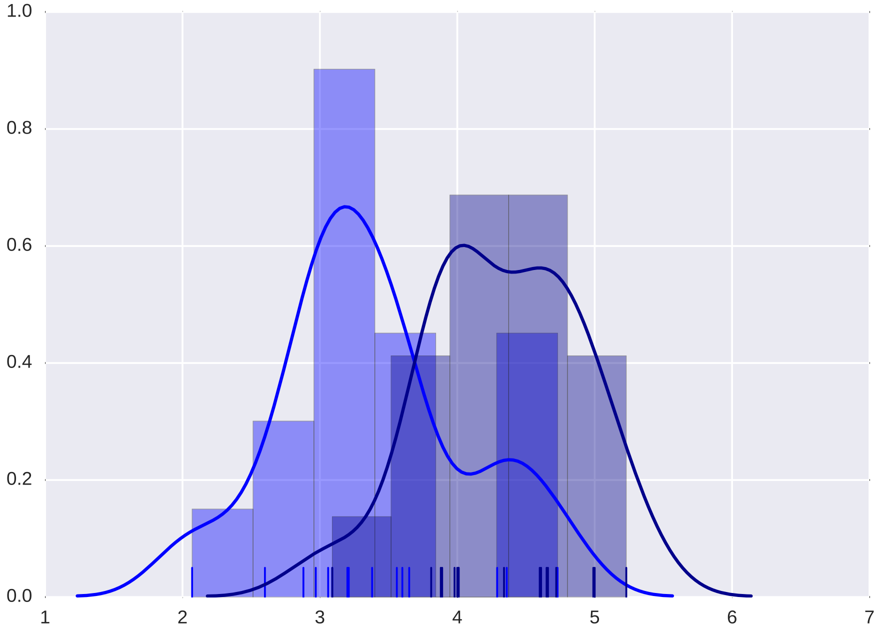

Figure 130: Significance Test Results for Lipid peroxidation levels between the group treated with *Withania somnifera*(LPO_W) and the MPTP disease induced and treated with both *Withania somnifera* and *Centella asiatica* group(LPO_MWC). As both the sets were normal, a Student's T-test was used. The p-value obtained for the test was 0.0002 and hence the difference between the data sets was considered statistically significant.

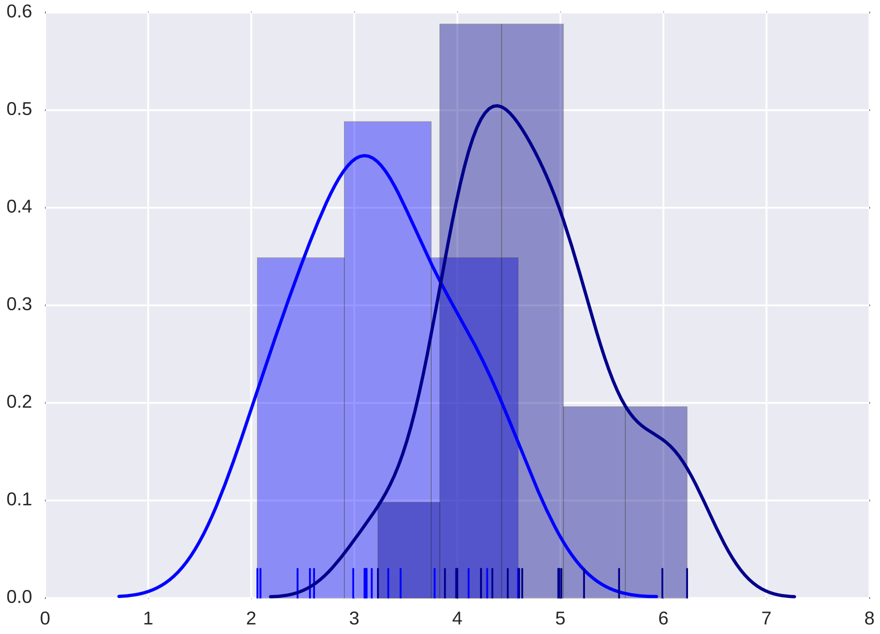

Figure 131: Significance Test Results for Lipid peroxidation levels between the group treated with both *Withania somnifera* and *Centella asiatica*(LPO_WC) and the MPTP disease induced and no treatment group(LPO_M). As both the sets were normal, a Student's T-test was used. The p-value obtained for the test was 0.0000 and hence the difference between the data sets was considered statistically significant.

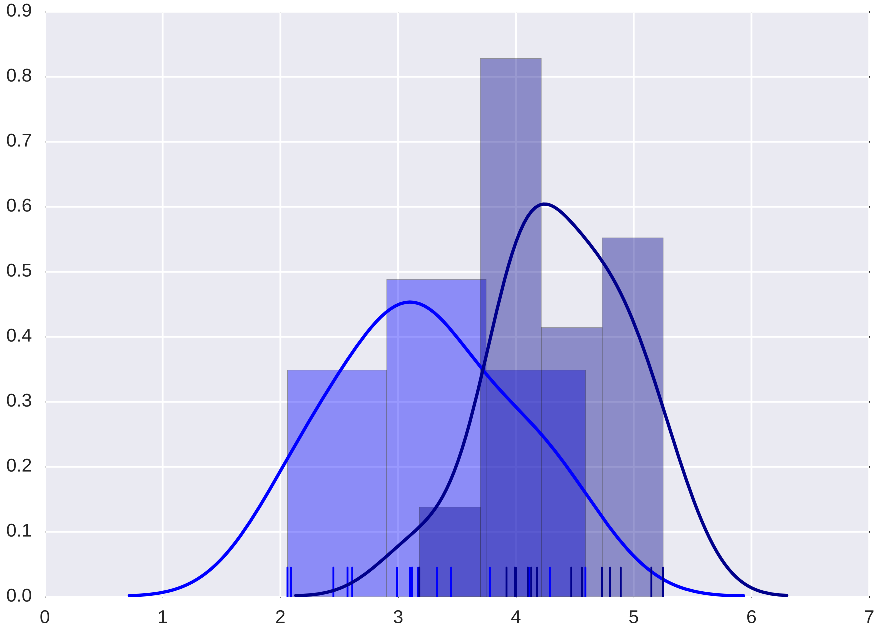

Figure 132: Significance Test Results for Lipid peroxidation levels between the group treated with both *Withania somnifera* and *Centella asiatica*(LPO_WC) and the MPTP disease induced and treated with *Centella asiatica* group(LPO_MC). As both the sets were normal, a Student's T-test was used. The p-value obtained for the test was 0.0000 and hence the difference between the data sets was considered statistically significant.

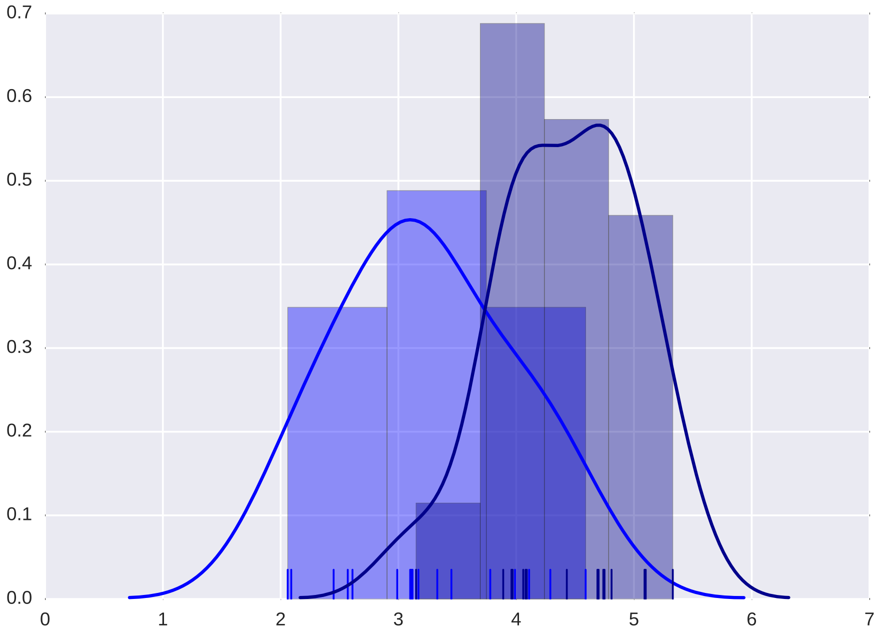

Figure 133: Significance Test Results for Lipid peroxidation levels between the group treated with both *Withania somnifera* and *Centella asiatica*(LPO_WC) and the MPTP disease induced and treated with *Withania somnifera* group(LPO_MW). As both the sets were normal, a Student's T-test was used. The p-value obtained for the test was 0.0000 and hence the difference between the data sets was considered statistically significant.

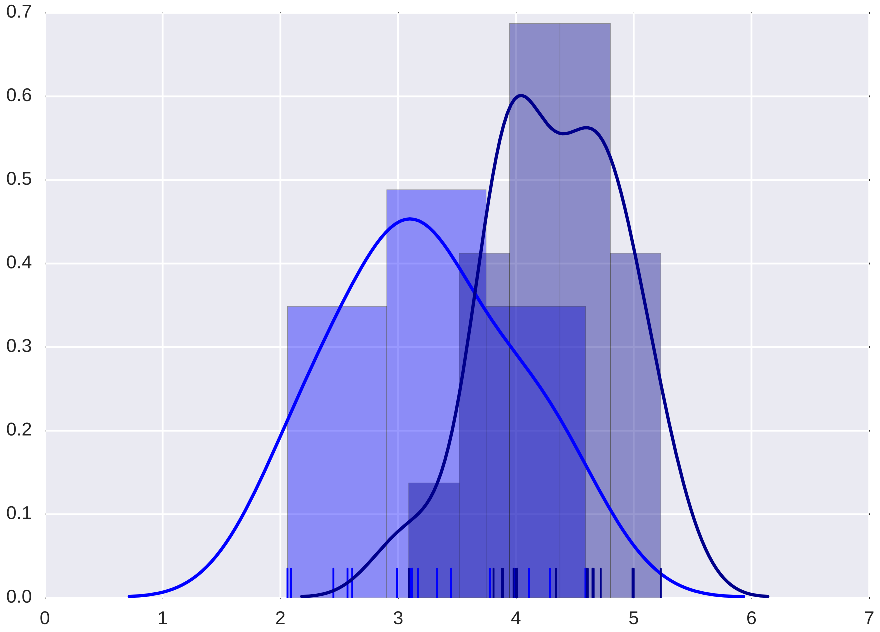

Figure 134: Significance Test Results for Lipid peroxidation levels between the group treated with both *Withania somnifera* and *Centella asiatica*(LPO_WC) and the MPTP disease induced and treated with both *Withania somnifera* and *Centella asiatica* group(LPO_MWC). As both the sets were normal, a Student's T-test was used. The p-value obtained for the test was 0.0000 and hence the difference between the data sets was considered statistically significant.

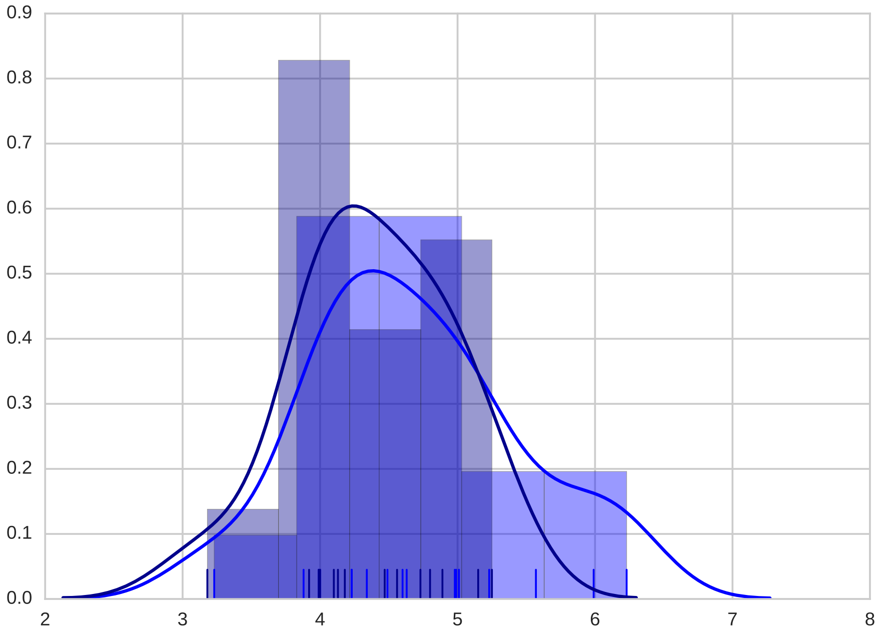

Figure 135: Significance Test Results for Lipid peroxidation levels between the MPTP disease induced and no treatment group(LPO_M) and the MPTP disease induced and treated with *Centella asiatica* group(LPO_MC). As both the sets were normal, a Student's T-test was used. The p-value obtained for the test was 0.2364 and hence the difference between the data sets was considered statistically non-significant.

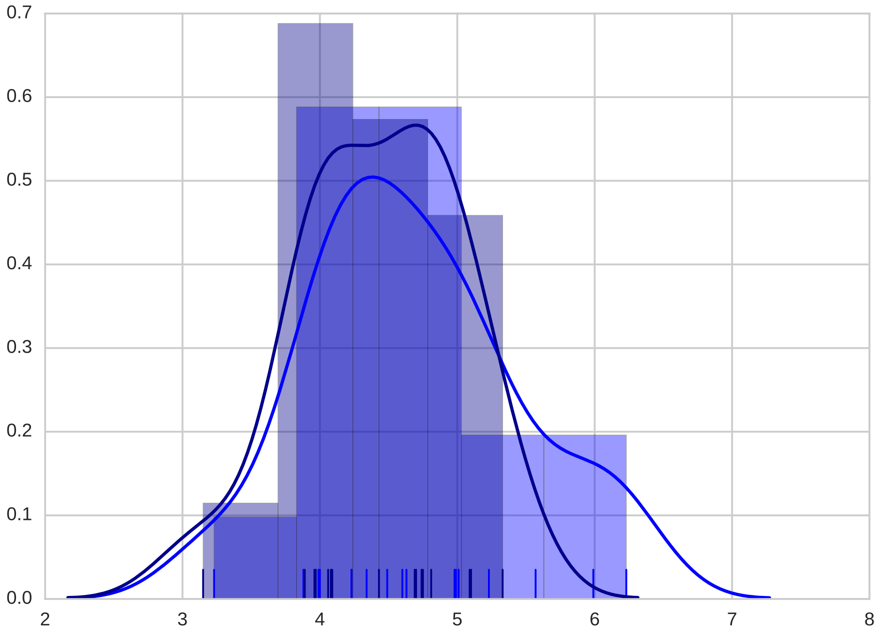

Figure 136: Significance Test Results for Lipid peroxidation levels between the MPTP disease induced and no treatment group(LPO_M) and the MPTP disease induced and treated with *Withania somnifera* group(LPO_MW). As both the sets were normal, a Student's T-test was used. The p-value obtained for the test was 0.2935 and hence the difference between the data sets was considered statistically non-significant.

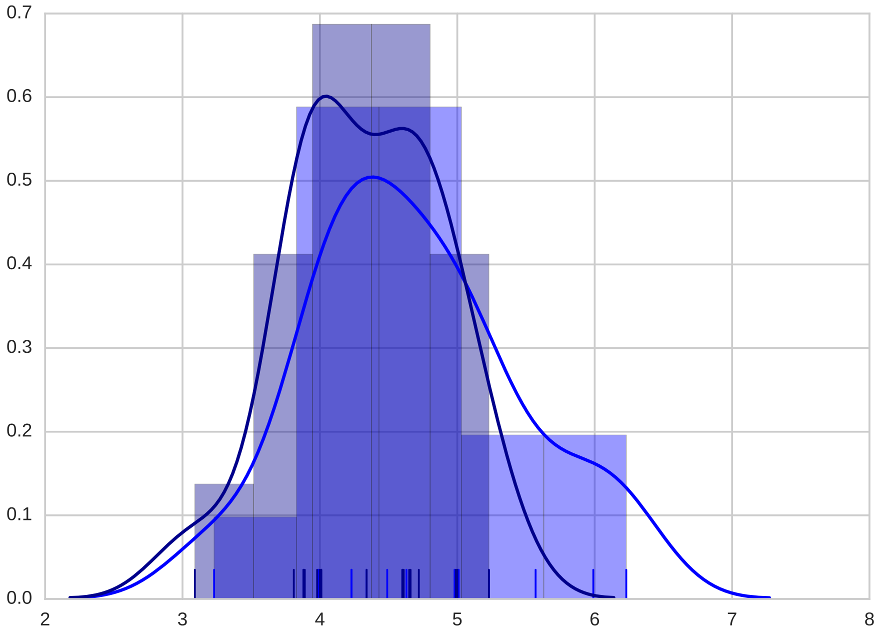

Figure 137: Significance Test Results for Lipid peroxidation levels between the MPTP disease induced and no treatment group(LPO_M) and the MPTP disease induced and treated with both *Withania somnifera* and *Centella asiatica* group(LPO_MWC). As both the sets were normal, a Student's T-test was used. The p-value obtained for the test was 0.1283 and hence the difference between the data sets was considered statistically non-significant.

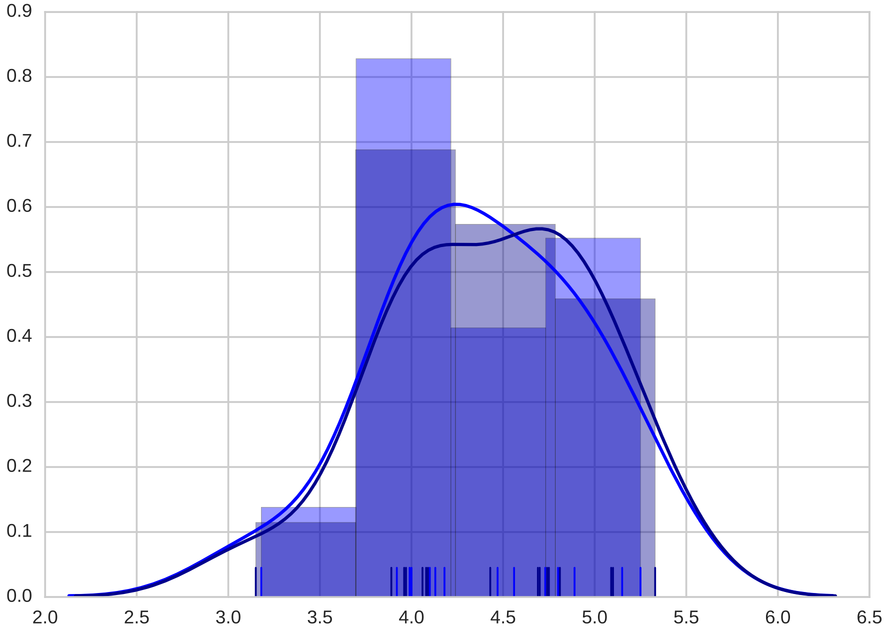

Figure 138: Significance Test Results for Lipid peroxidation levels between the MPTP disease induced and treated with *Centella asiatica* group(LPO_MC) and the MPTP disease induced and treated with *Withania somnifera* group(LPO_MW). As both the sets were normal, a Student's T-test was used. The p-value obtained for the test was 0.8285 and hence the difference between the data sets was considered statistically non-significant.

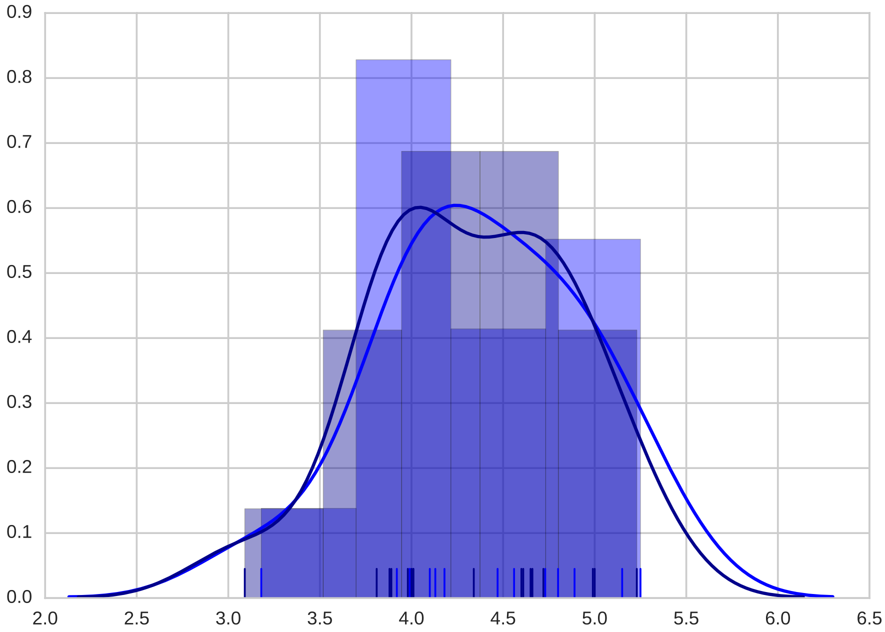

Figure 139: Significance Test Results for Lipid peroxidation levels between the MPTP disease induced and treated with *Centella asiatica* group(LPO_MC) and the MPTP disease induced and treated with both *Withania somnifera* and *Centella asiatica* group(LPO_MWC). As both the sets were normal, a Student's T-test was used. The p-value obtained for the test was 0.7652 and hence the difference between the data sets was considered statistically non-significant.

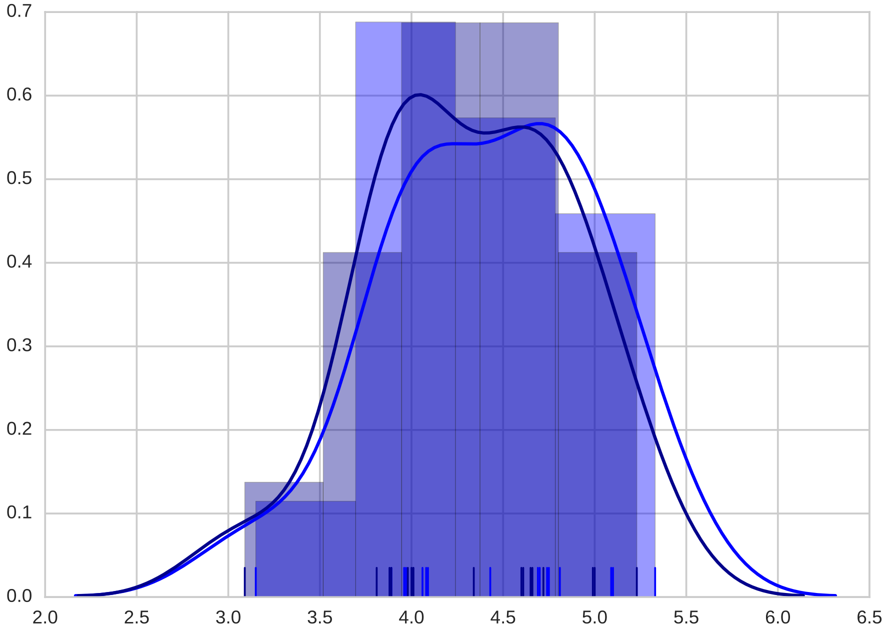

Figure 140: Significance Test Results for Lipid peroxidation levels between the MPTP disease induced and treated with *Withania somnifera* group(LPO_MW) and the MPTP disease induced and treated with both *Withania somnifera* and *Centella asiatica* group(LPO_MWC). As both the sets were normal, a Student's T-test was used. The p-value obtained for the test was 0.5924 and hence the difference between the data sets was considered statistically non-significant.

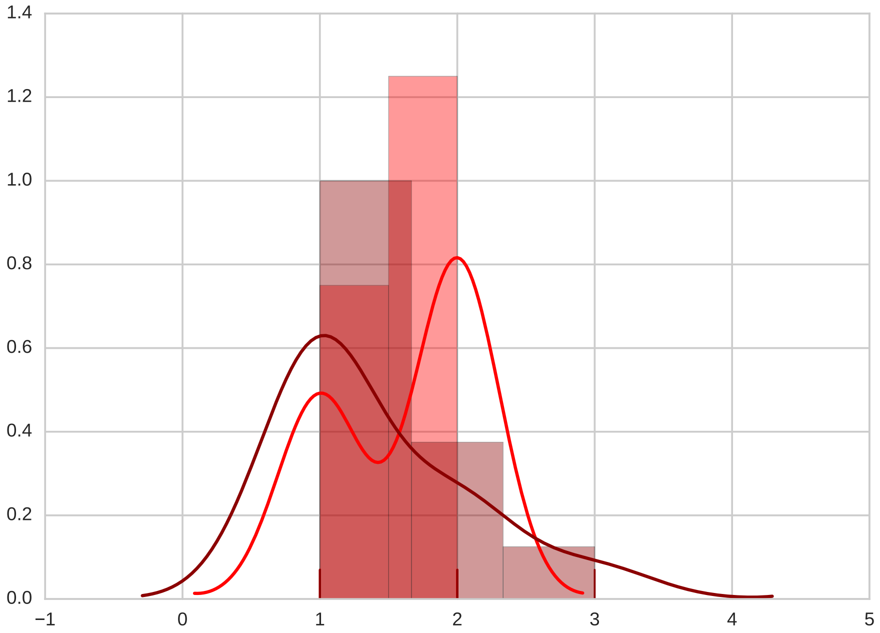

Figure 141: Significance Test Results for Akinesia results between the untreated group(AU) and the group treated with *Centella asiatica*(AC). As both the sets were non-normal, we used a Mann-Whitney U Test. The p-value obtained for the test was 0.2357 and hence the difference between the data sets was considered statistically non-significant.

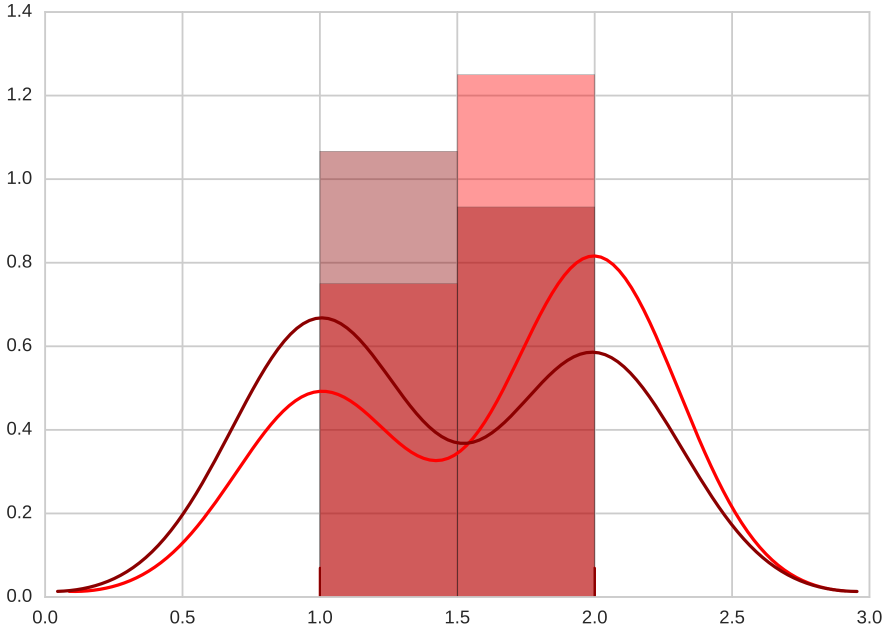

Figure 142: Significance Test Results for Akinesia results between the untreated group(AU) and the group treated with *Withania somnifera*(AW). As both the sets were non-normal, we used a Mann-Whitney U Test. The p-value obtained for the test was 0.3965 and hence the difference between the data sets was considered statistically non-significant.

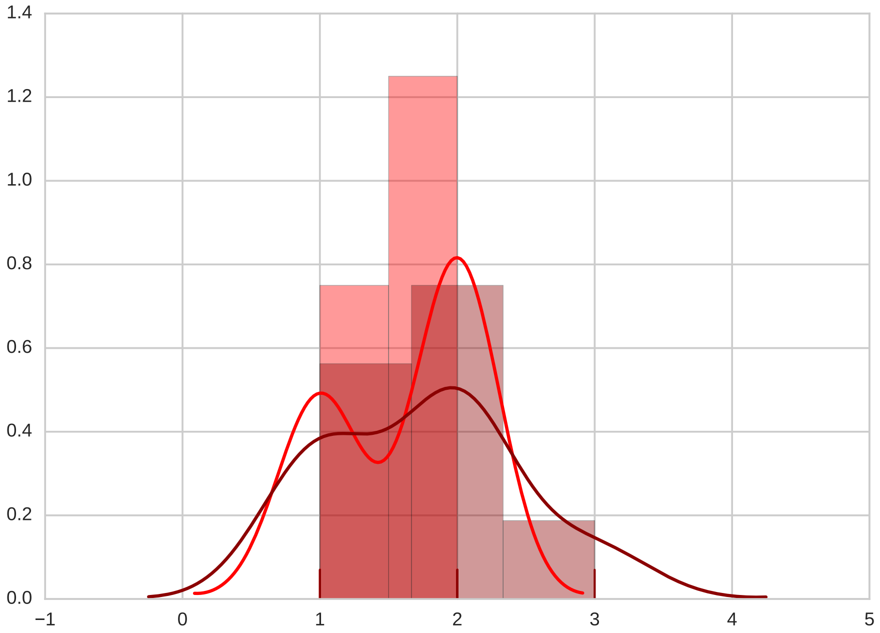

Figure 143: Significance Test Results for Akinesia results between the untreated group(AU) and the group treated with both *Withania somnifera* and *Centella asiatica*(AWC). As both the sets were non-normal, we used a Mann-Whitney U Test. The p-value obtained for the test was 0.6832 and hence the difference between the data sets was considered statistically non-significant.

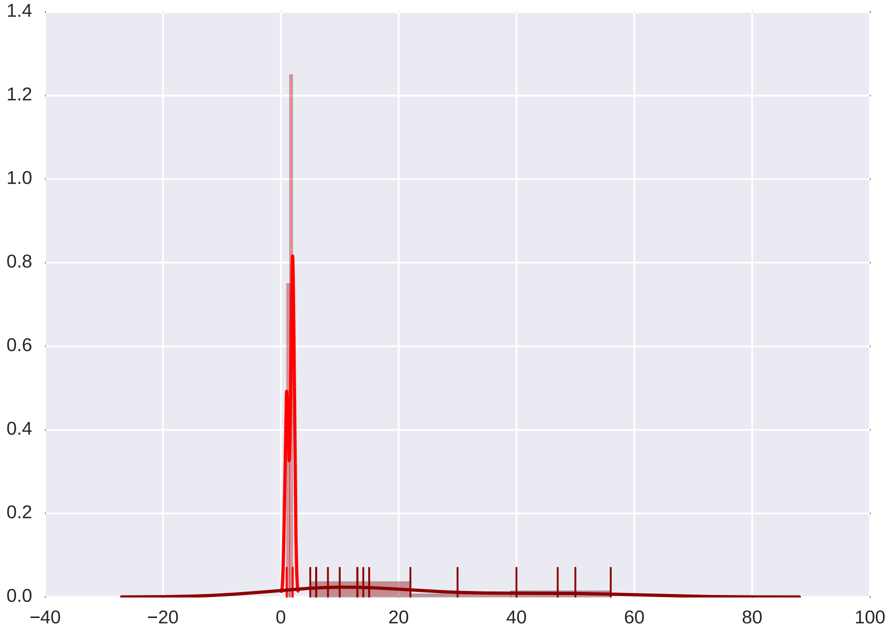

Figure 144: Significance Test Results for Akinesia results between the untreated group(AU) and the MPTP disease induced and no treatment group(AM). As both the sets were non-normal but the variances were unequal, we used a Welch's T Test with ranked data. The p-value obtained for the test was 0.0000 and hence the difference between the data sets was considered statistically significant.

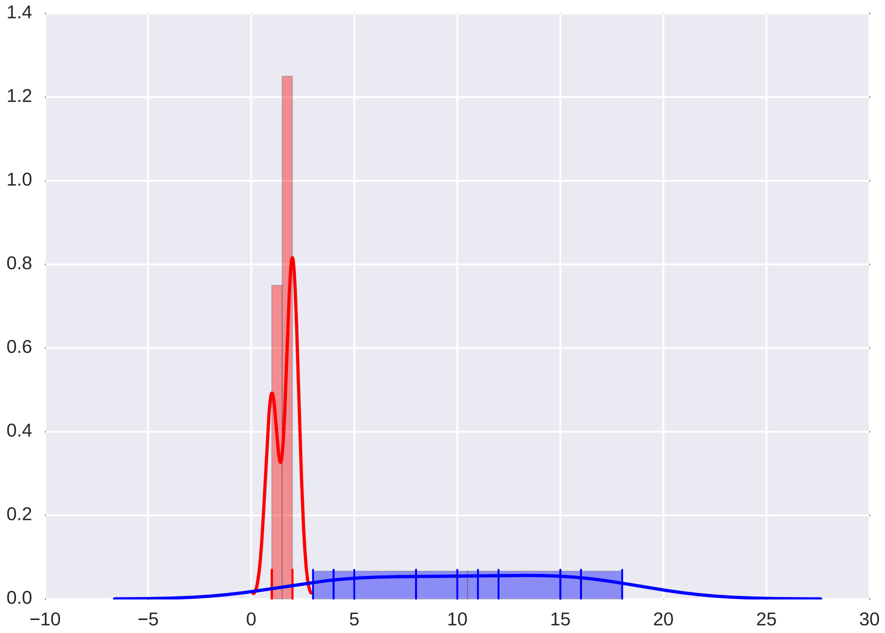

Figure 145: Significance Test Results for Akinesia results between the untreated group(AU) and the MPTP disease induced and treated with *Centella asiatica* group(AMC). As one of the sets was non-normal but the variances were unequal, we used a Welch's T Test with ranked data. The p-value obtained for the test was 0.0000 and hence the difference between the data sets was considered statistically significant.

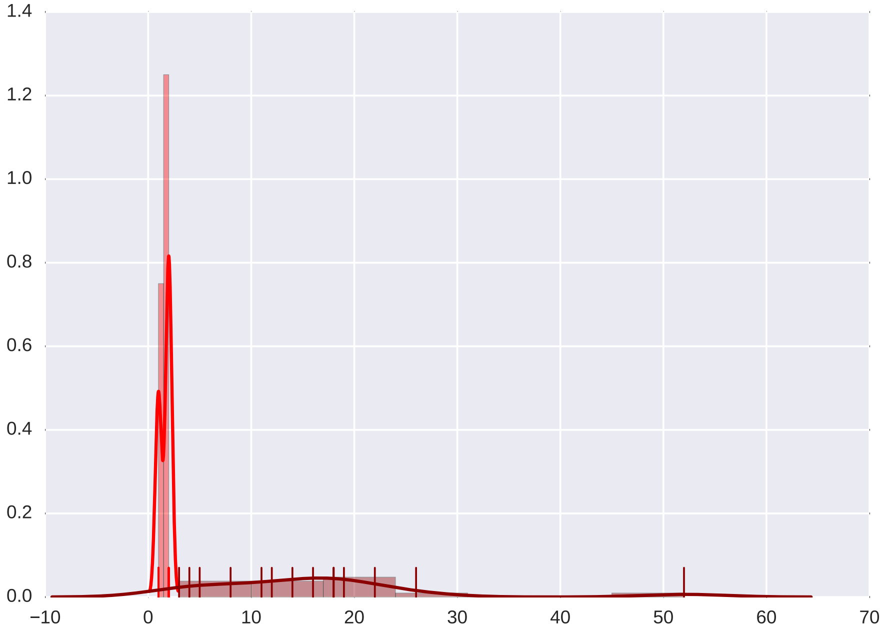

Figure 146: Significance Test Results for Akinesia results between the untreated group(AU) and the MPTP disease induced and treated with *Withania somnifera* group(AMW). As both the sets were non-normal but the variances were unequal, we used a Welch's T Test with ranked data. The p-value obtained for the test was 0.0000 and hence the difference between the data sets was considered statistically significant.

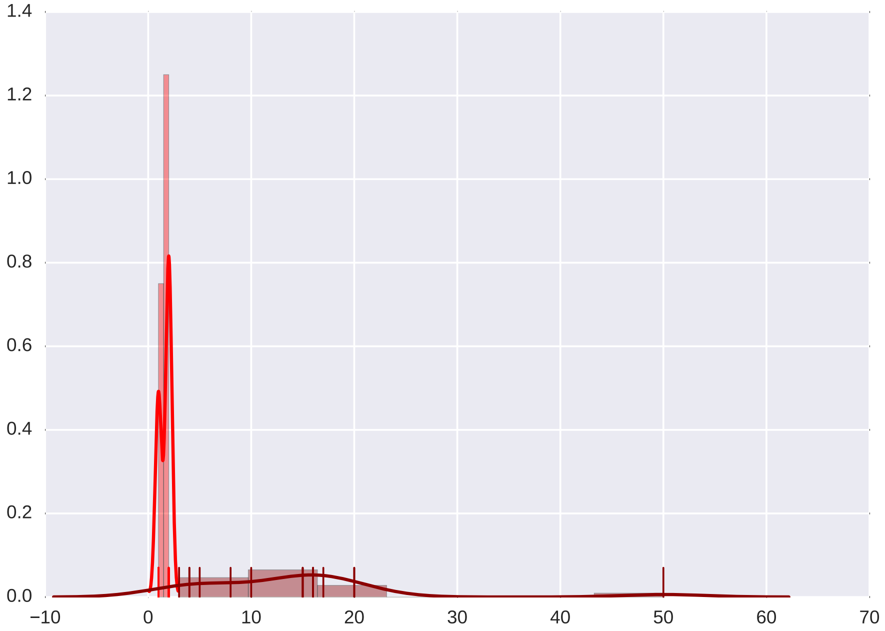

Figure 147: Significance Test Results for Akinesia results between the untreated group(AU) and the MPTP disease induced and treated with both *Withania somnifera* and *Centella asiatica* group(AMWC). As both the sets were non-normal but the variances were unequal, we used a Welch's T Test with ranked data. The p-value obtained for the test was 0.0000 and hence the difference between the data sets was considered statistically significant.

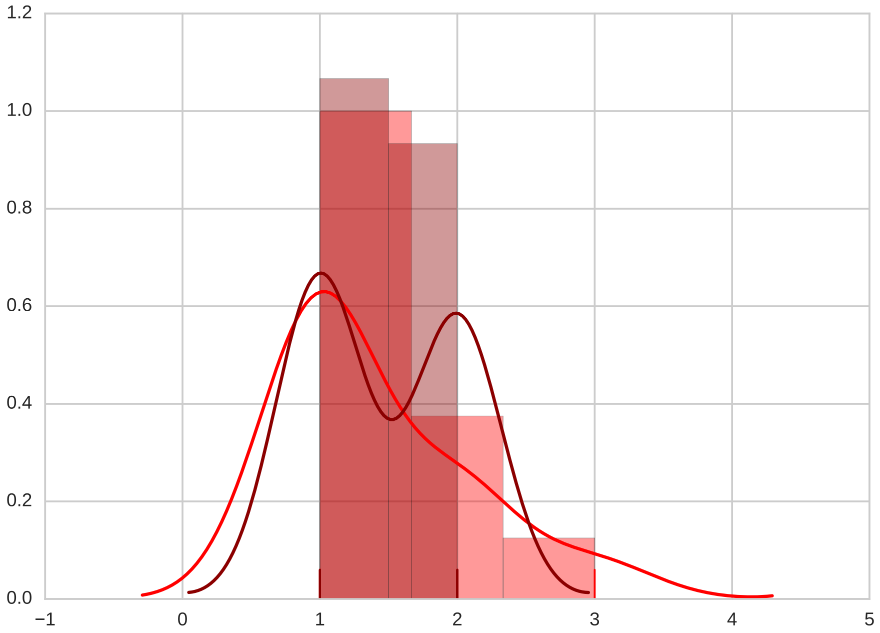

Figure 148: Significance Test Results for Akinesia results between the group treated with *Centella asiatica*(AC) and the group treated with *Withania somnifera*(AW). As both the sets were non-normal, we used a Mann-Whitney U Test. The p-value obtained for the test was 0.6504 and hence the difference between the data sets was considered statistically non-significant.

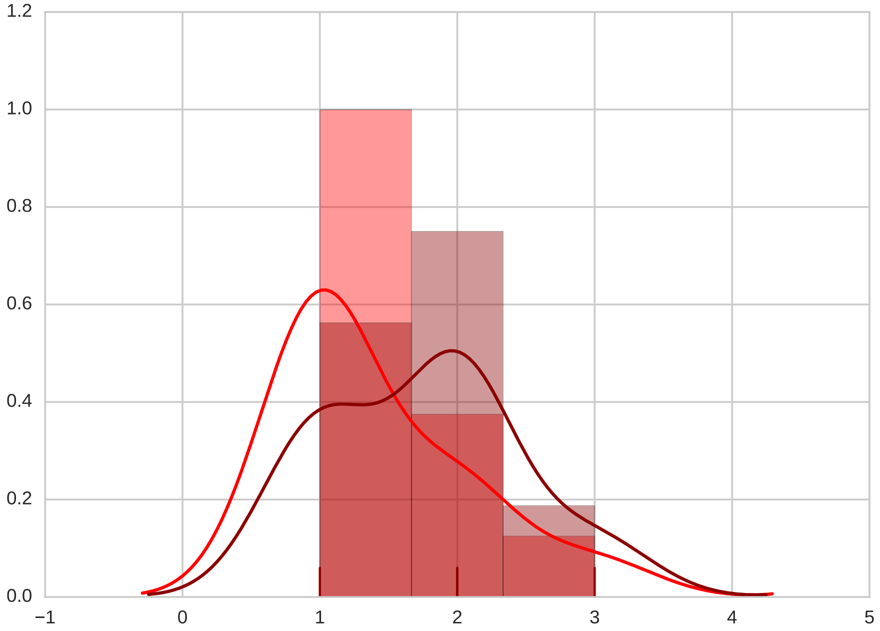

Figure 149: Significance Test Results for Akinesia results between the group treated with *Centella asiatica*(AC) and the group treated with both *Withania somnifera* and *Centella asiatica*(AWC). As both the sets were non-normal, we used a Mann-Whitney U Test. The p-value obtained for the test was 0.1728 and hence the difference between the data sets was considered statistically non-significant.

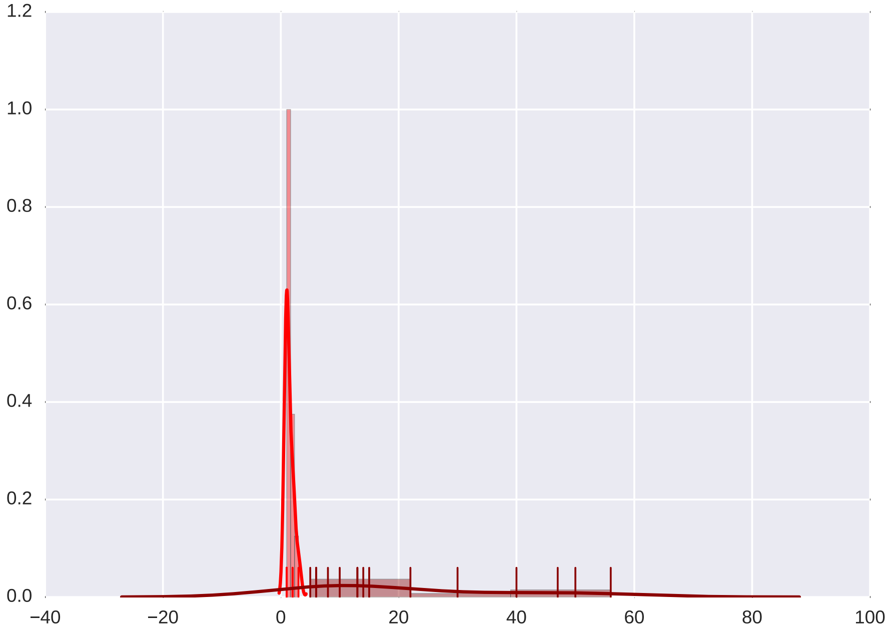

Figure 150: Significance Test Results for Akinesia results between the group treated with *Centella asiatica*(AC) and the MPTP disease induced and no treatment group(AM). As both the sets were non-normal but the variances were unequal, we used a Welch's T Test with ranked data. The p-value obtained for the test was 0.0000 and hence the difference between the data sets was considered statistically significant.

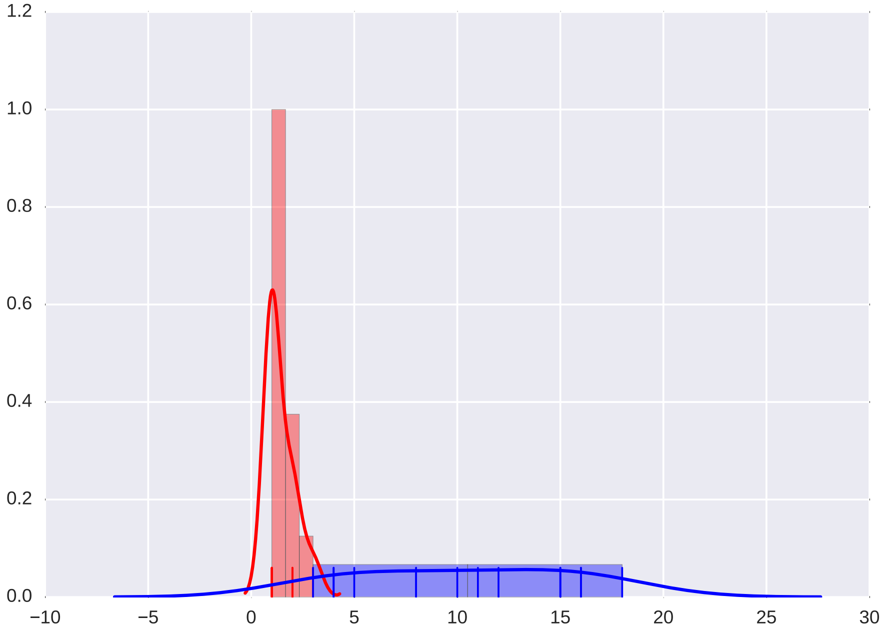

Figure 151: Significance Test Results for Akinesia results between the group treated with *Centella asiatica*(AC) and the MPTP disease induced and treated with *Centella asiatica* group(AMC). As one of the sets was non-normal but the variances were unequal, we used a Welch's T Test with ranked data. The p-value obtained for the test was 0.0000 and hence the difference between the data sets was considered statistically significant.

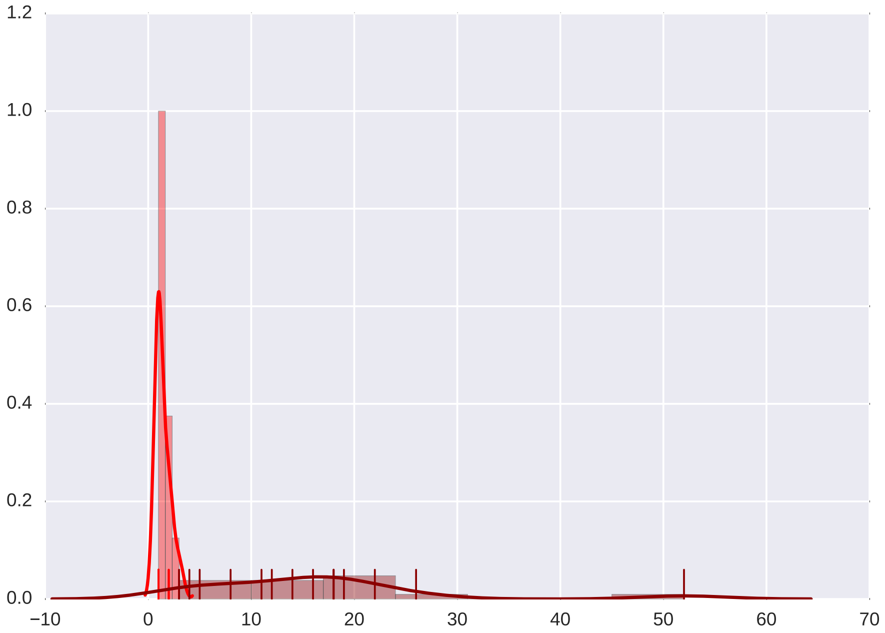

Figure 152: Significance Test Results for Akinesia results between the group treated with *Centella asiatica*(AC) and the MPTP disease induced and treated with *Withania somnifera* group(AMW). As both the sets were non-normal but the variances were unequal, we used a Welch's T Test with ranked data. The p-value obtained for the test was 0.0000 and hence the difference between the data sets was considered statistically significant.

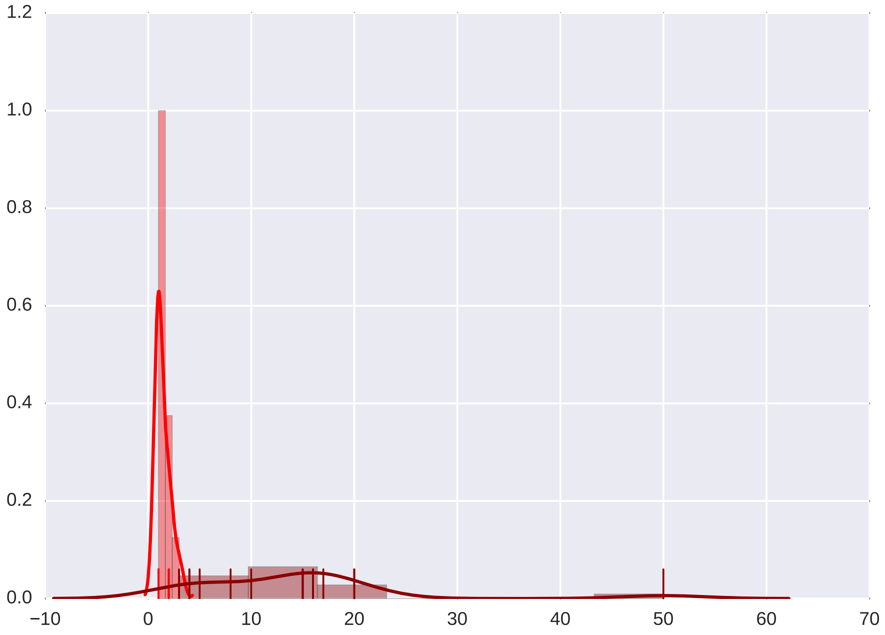

Figure 153: Significance Test Results for Akinesia results between the group treated with *Centella asiatica*(AC) and the MPTP disease induced and treated with both *Withania somnifera* and *Centella asiatica* group(AMWC). As both the sets were non-normal but the variances were unequal, we used a Welch's T Test with ranked data. The p-value obtained for the test was 0.0000 and hence the difference between the data sets was considered statistically significant.

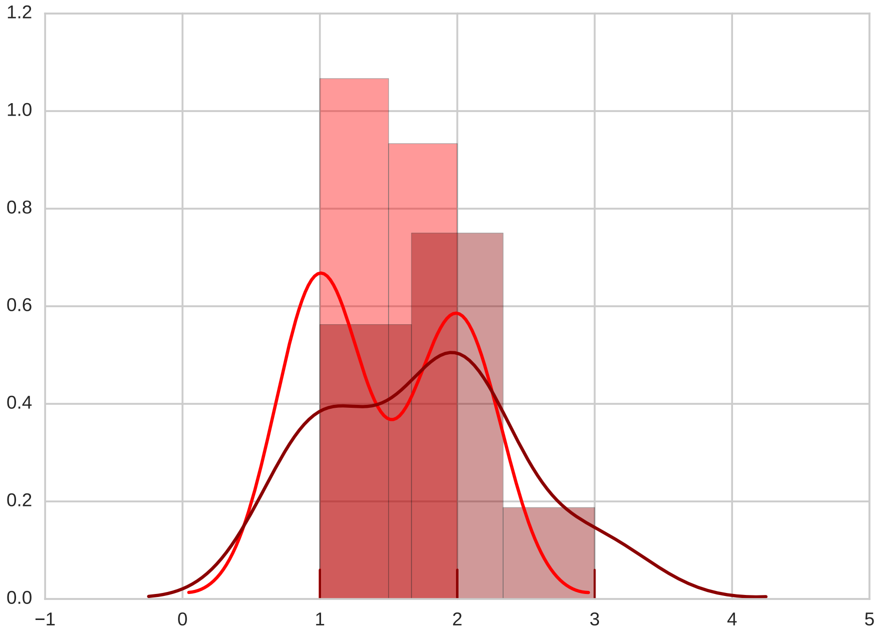

Figure 154: Significance Test Results for Akinesia results between the group treated with *Withania somnifera*(AW) and the group treated with both *Withania somnifera* and *Centella asiatica*(AWC). As both the sets were non-normal, we used a Mann-Whitney U Test. The p-value obtained for the test was 0.2583 and hence the difference between the data sets was considered statistically non-significant.

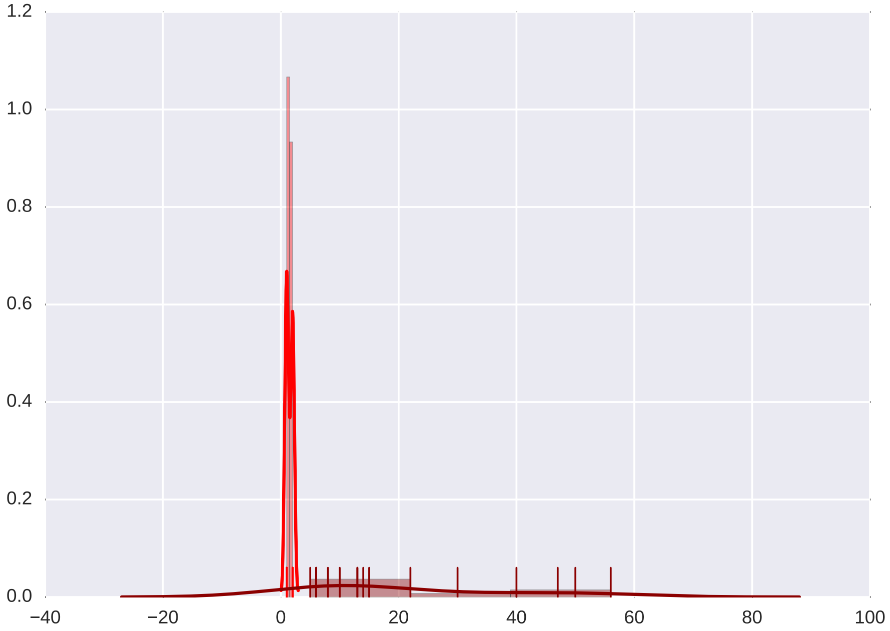

Figure 155: Significance Test Results for Akinesia results between the group treated with *Withania somnifera*(AW) and the MPTP disease induced and no treatment group(AM). As both the sets were non-normal but the variances were unequal, we used a Welch's T Test with ranked data. The p-value obtained for the test was 0.0000 and hence the difference between the data sets was considered statistically significant.

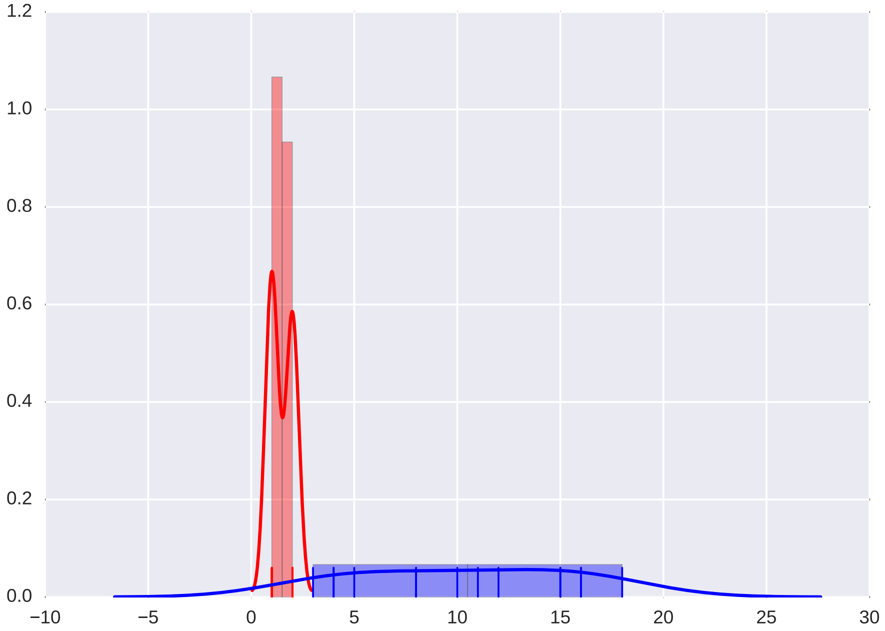

Figure 156: Significance Test Results for Akinesia results between the group treated with *Withania somnifera*(AW) and the MPTP disease induced and treated with *Centella asiatica* group(AMC). As one of the sets was non-normal but the variances were unequal, we used a Welch's T Test with ranked data. The p-value obtained for the test was 0.0000 and hence the difference between the data sets was considered statistically significant.

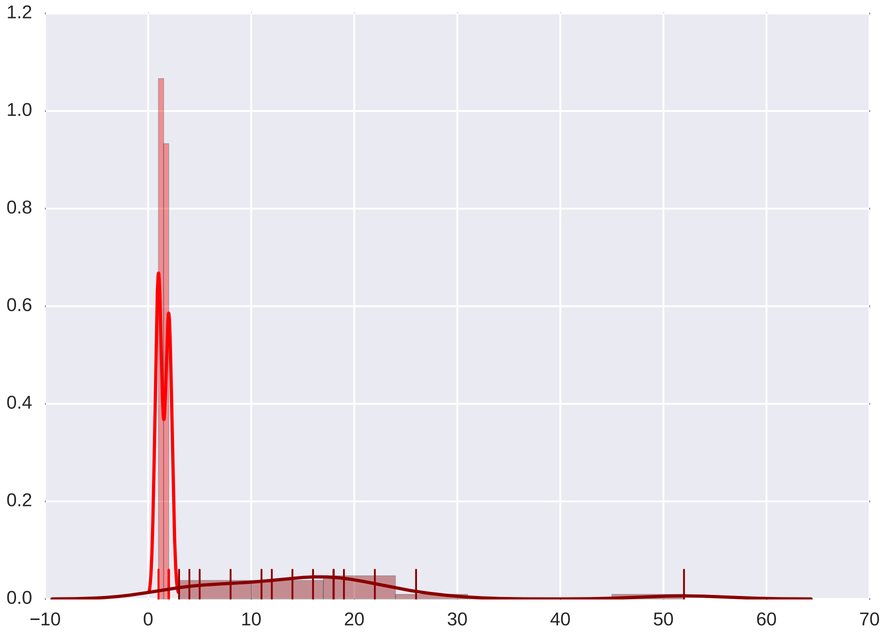

Figure 157: Significance Test Results for Akinesia results between the group treated with *Withania somnifera*(AW) and the MPTP disease induced and treated with *Withania somnifera* group(AMW). As both the sets were non-normal but the variances were unequal, we used a Welch's T Test with ranked data. The p-value obtained for the test was 0.0000 and hence the difference between the data sets was considered statistically significant.

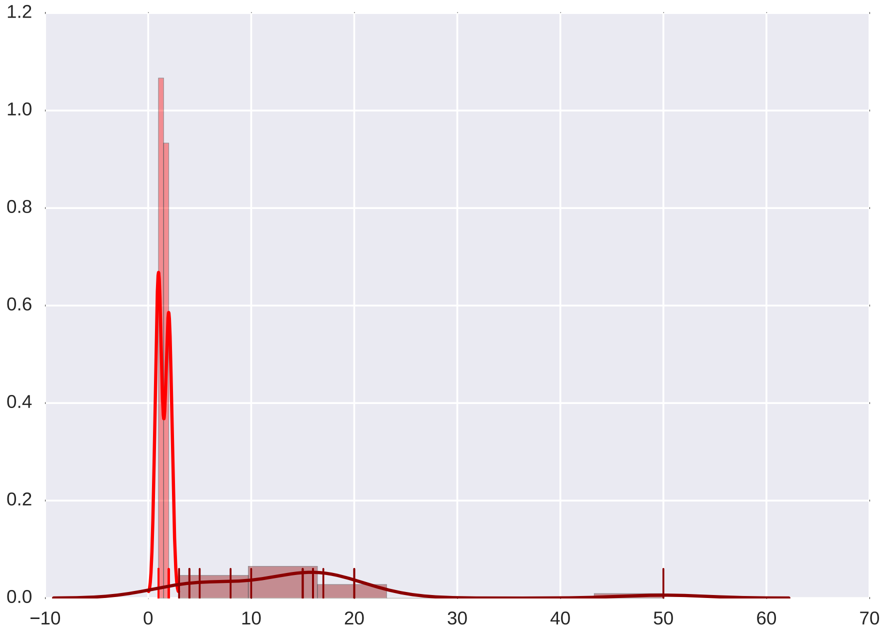

Figure 158: Significance Test Results for Akinesia results between the group treated with *Withania somnifera*(AW) and the MPTP disease induced and treated with both *Withania somnifera* and *Centella asiatica* group(AMWC). As both the sets were non-normal but the variances were unequal, we used a Welch's T Test with ranked data. The p-value obtained for the test was 0.0000 and hence the difference between the data sets was considered statistically significant.

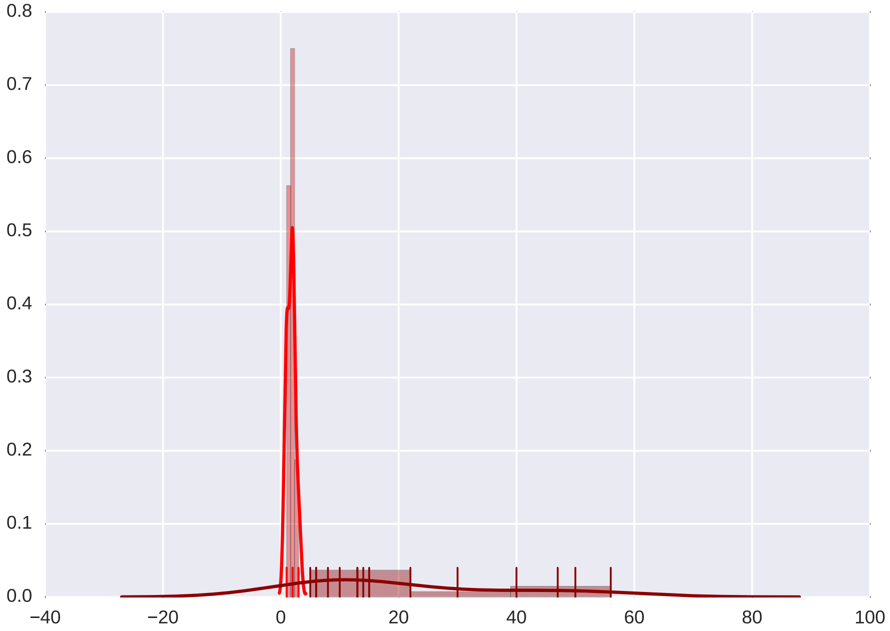

Figure 159: Significance Test Results for Akinesia results between the group treated with both *Withania somnifera* and *Centella asiatica*(AWC) and the MPTP disease induced and no treatment group(AM). As both the sets were non-normal but the variances were unequal, we used a Welch's T Test with ranked data. The p-value obtained for the test was 0.0000 and hence the difference between the data sets was considered statistically significant.

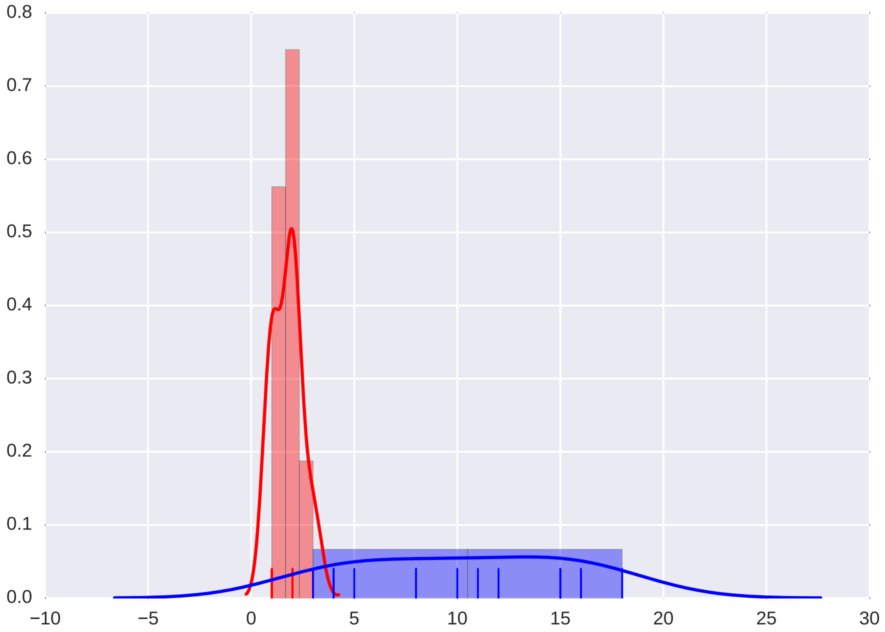

Figure 160: Significance Test Results for Akinesia results between the group treated with both *Withania somnifera* and *Centella asiatica*(AWC) and the MPTP disease induced and treated with *Centella asiatica* group(AMC). As one of the sets was non-normal but the variances were unequal, we used a Welch's T Test with ranked data. The p-value obtained for the test was 0.0000 and hence the difference between the data sets was considered statistically significant.

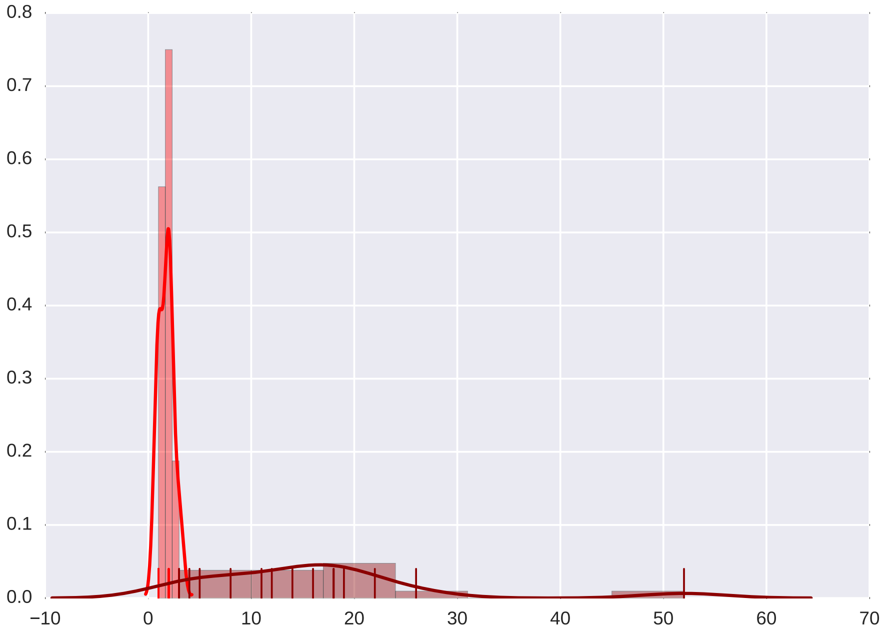

Figure 161: Significance Test Results for Akinesia results between the group treated with both *Withania somnifera* and *Centella asiatica*(AWC) and the MPTP disease induced and treated with *Withania somnifera* group(AMW). As both the sets were non-normal but the variances were unequal, we used a Welch's T Test with ranked data. The p-value obtained for the test was 0.0000 and hence the difference between the data sets was considered statistically significant.

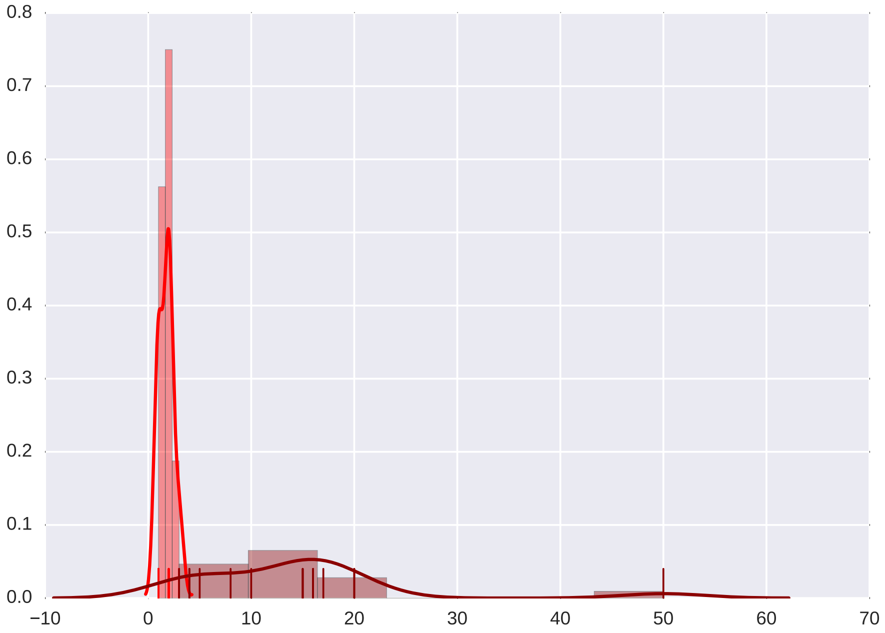

Figure 162: Significance Test Results for Akinesia results between the group treated with both *Withania somnifera* and *Centella asiatica*(AWC) and the MPTP disease induced and treated with both *Withania somnifera* and *Centella asiatica* group(AMWC). As both the sets were non-normal but the variances were unequal, we used a Welch's T Test with ranked data. The p-value obtained for the test was 0.0000 and hence the difference between the data sets was considered statistically significant.

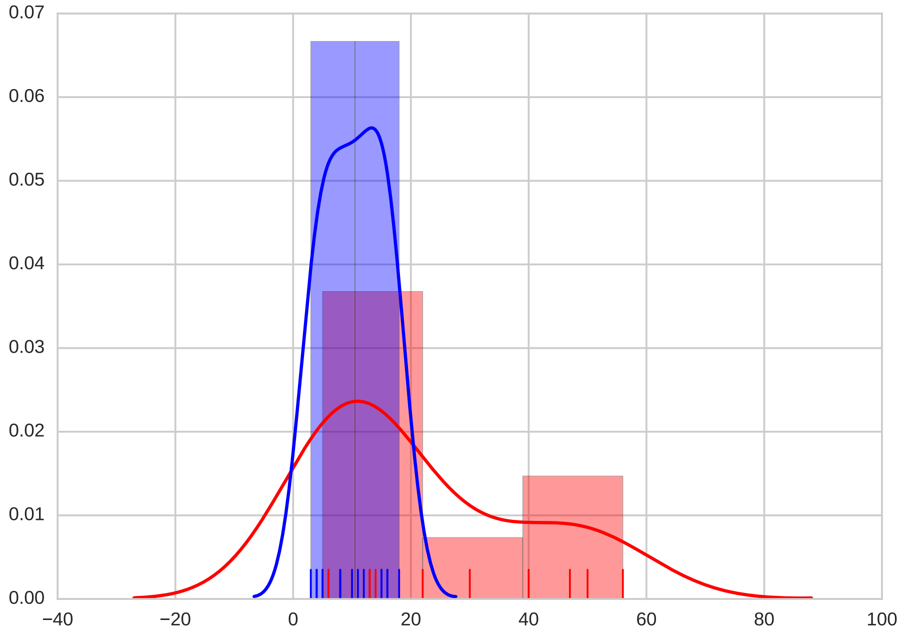

Figure 163: Significance Test Results for Akinesia results between the MPTP disease induced and no treatment group(AM) and the MPTP disease induced and treated with *Centella asiatica* group(AMC). As one of the sets was non-normal but the variances were unequal, we used a Welch's T Test with ranked data. The p-value obtained for the test was 0.1338 and hence the difference between the data sets was considered statistically non-significant.

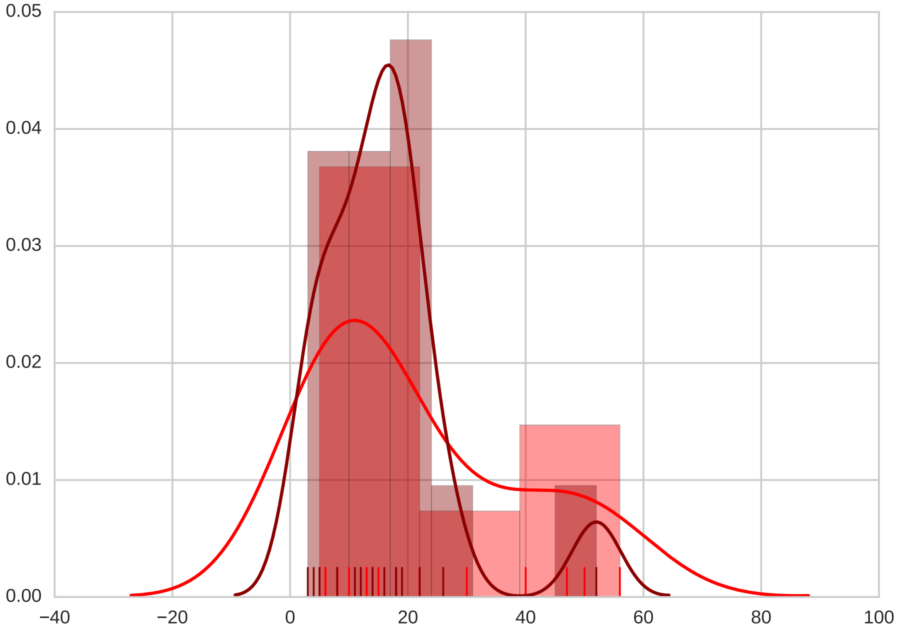

Figure 164: Significance Test Results for Akinesia results between the MPTP disease induced and no treatment group(AM) and the MPTP disease induced and treated with *Withania somnifera* group(AMW). As both the sets were non-normal, we used a Mann-Whitney U Test. The p-value obtained for the test was 0.7666 and hence the difference between the data sets was considered statistically non-significant.

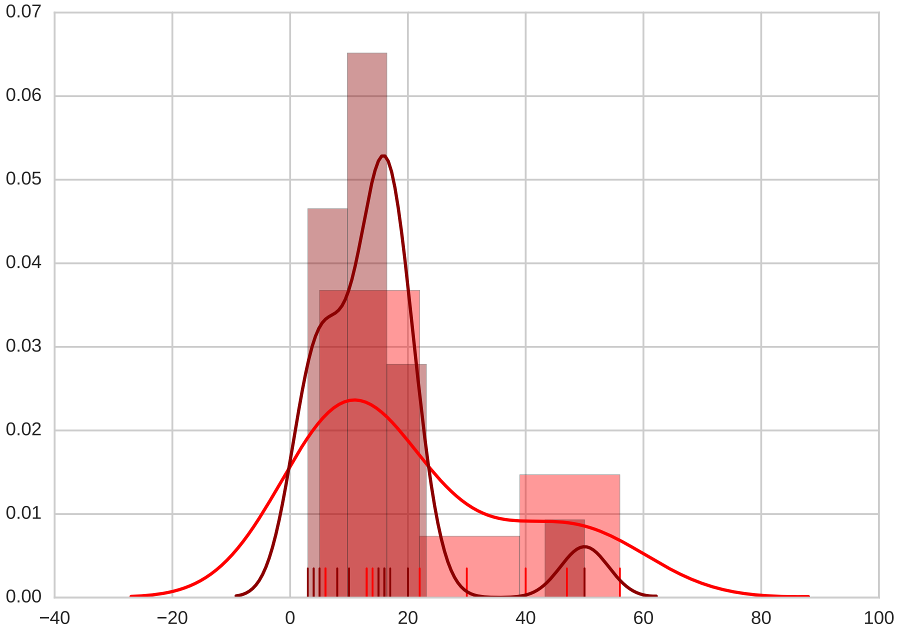

Figure 165: Significance Test Results for Akinesia results between the MPTP disease induced and no treatment group(AM) and the MPTP disease induced and treated with both *Withania somnifera* and *Centella asiatica* group(AMWC). As both the sets were non-normal, we used a Mann-Whitney U Test. The p-value obtained for the test was 0.5969 and hence the difference between the data sets was considered statistically non-significant.

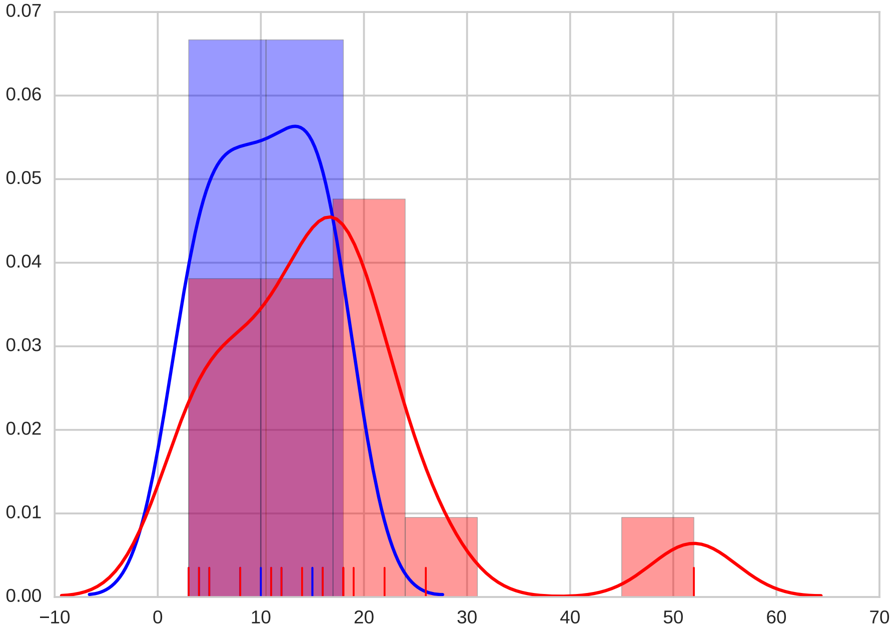

Figure 166: Significance Test Results for Akinesia results between the MPTP disease induced and treated with *Centella asiatica* group(AMC) and the MPTP disease induced and treated with *Withania somnifera* group(AMW). As one of the sets was non-normal, we used a Mann-Whitney U Test. The p-value obtained for the test was 0.0877 and hence the difference between the data sets was considered statistically non-significant.

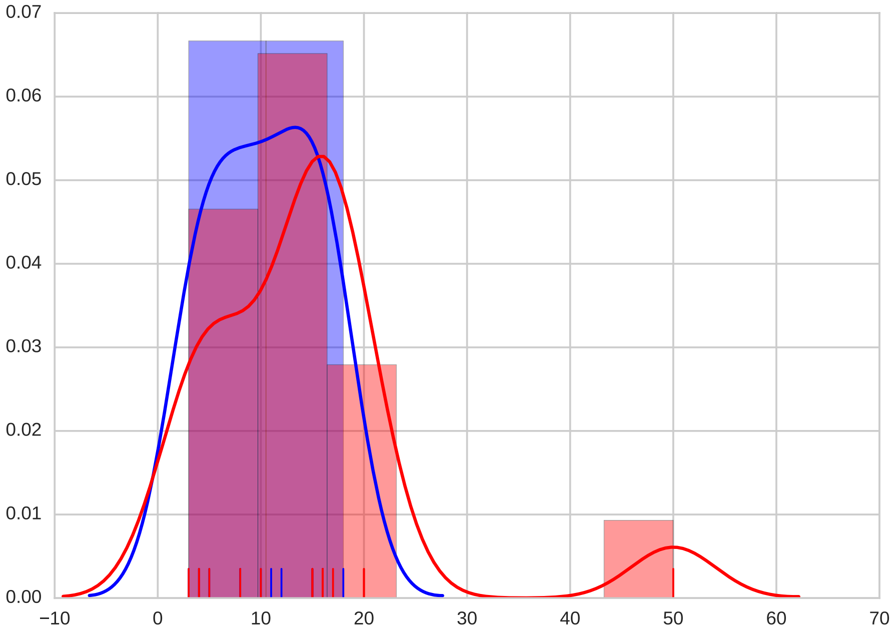

Figure 167: Significance Test Results for Akinesia results between the MPTP disease induced and treated with *Centella asiatica* group(AMC) and the MPTP disease induced and treated with both *Withania somnifera* and *Centella asiatica* group(AMWC). As one of the sets was non-normal, we used a Mann-Whitney U Test. The p-value obtained for the test was 0.2414 and hence the difference between the data sets was considered statistically non-significant.

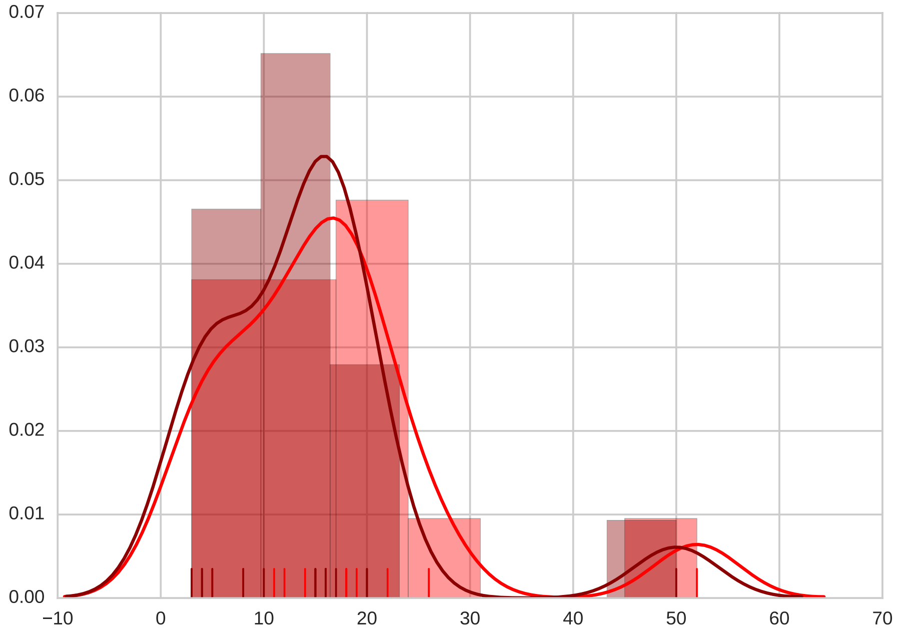

Figure 168: Significance Test Results for Akinesia results between the MPTP disease induced and treated with *Withania somnifera* group(AMW) and the MPTP disease induced and treated with both *Withania somnifera* and *Centella asiatica* group(AMWC). As both the sets were non-normal, we used a Mann-Whitney U Test. The p-value obtained for the test was 0.5132 and hence the difference between the data sets was considered statistically non-significant.

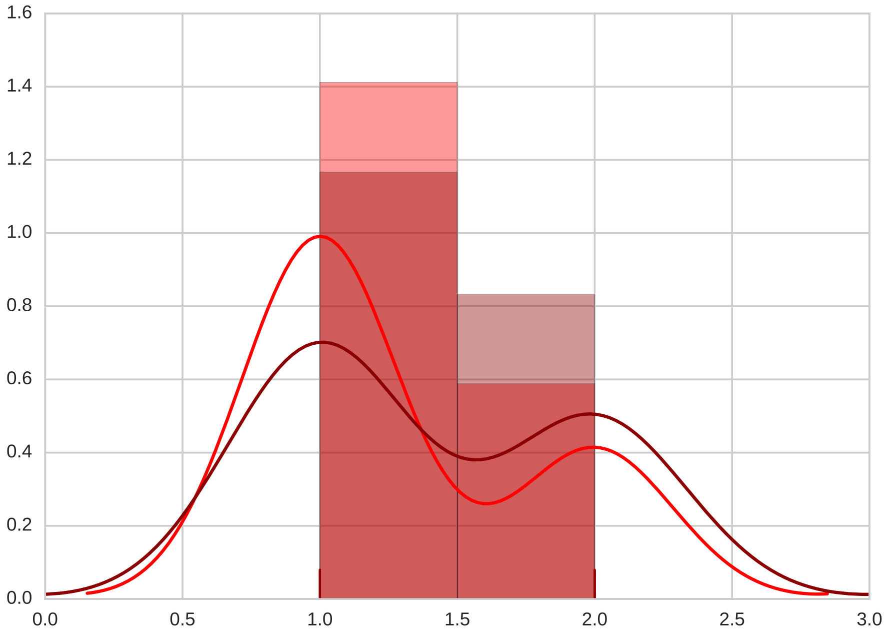

Figure 169: Significance Test Results for Catalepsy results between the untreated group(CU) and the group treated with *Centella asiatica*(CC). As both the sets were non-normal, we used a Mann-Whitney U Test. The p-value obtained for the test was 0.5189 and hence the difference between the data sets was considered statistically non-significant.

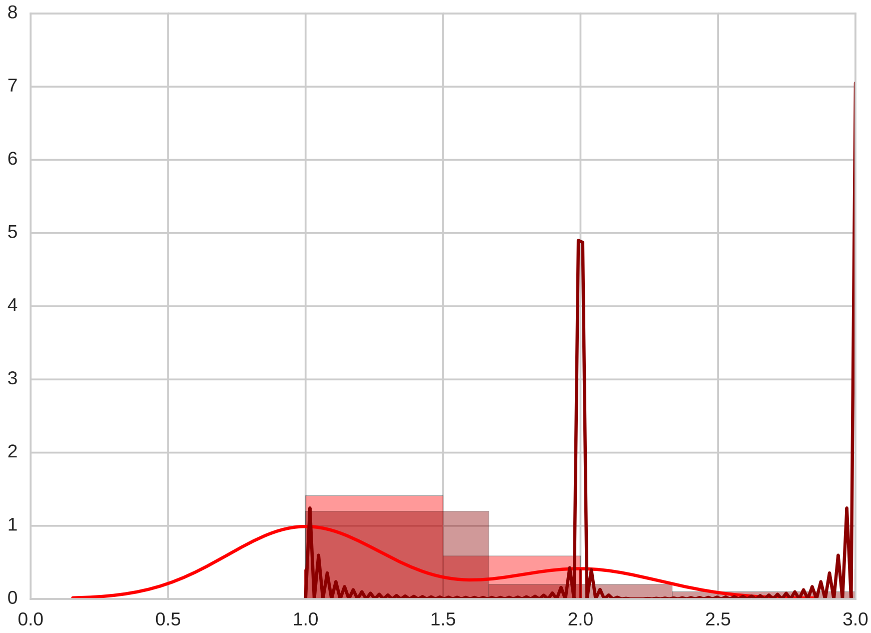

Figure 170: Significance Test Results for Catalepsy results between the untreated group(CU) and the group treated with *Withania somnifera*(CW). As both the sets were non-normal, we used a Mann-Whitney U Test. The p-value obtained for the test was 0.6521 and hence the difference between the data sets was considered statistically non-significant.

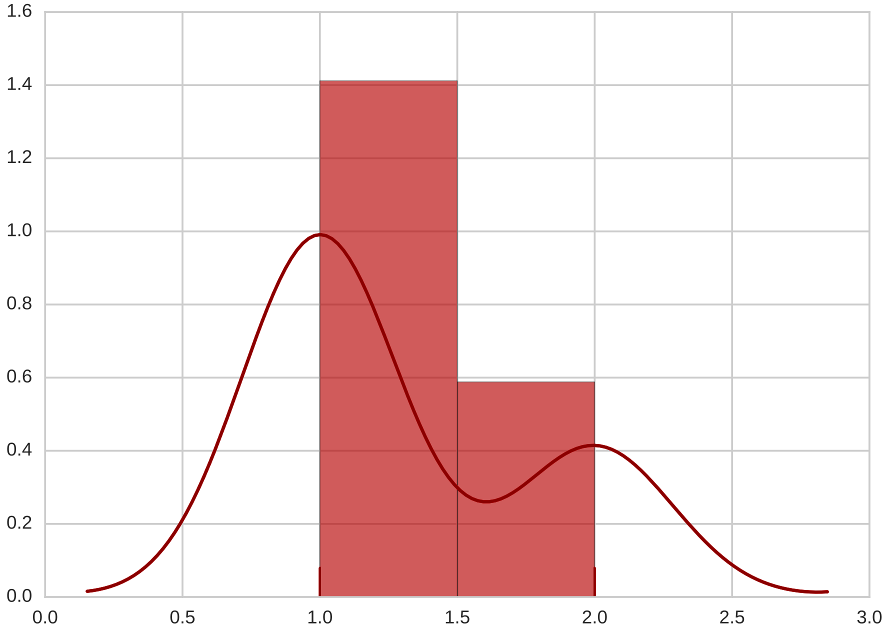

Figure 171: Significance Test Results for Catalepsy results between the untreated group(CU) and the group treated with both *Withania somnifera* and *Centella asiatica*(CWC). As both the sets were non-normal, we used a Mann-Whitney U Test. The p-value obtained for the test was 0.9826 and hence the difference between the data sets was considered statistically non-significant.

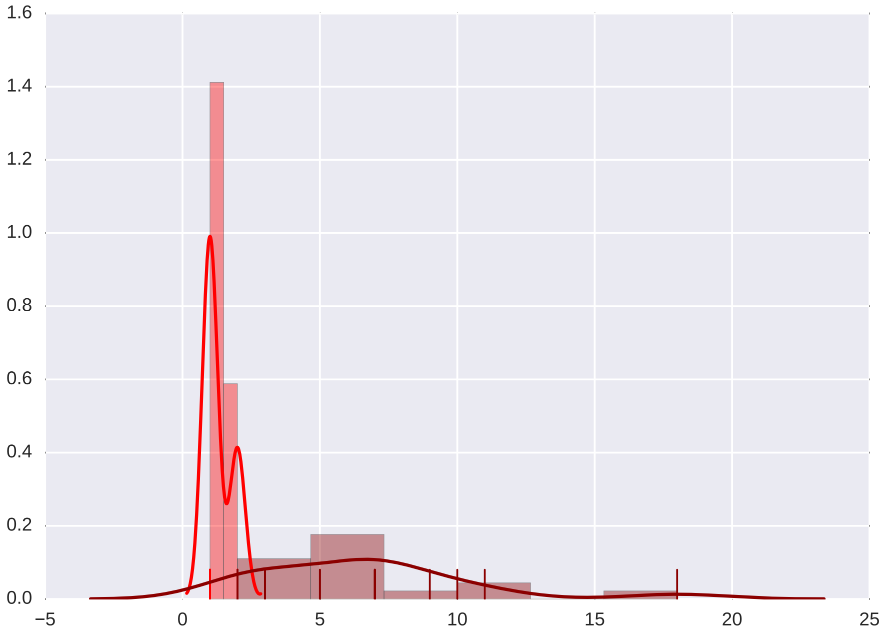

Figure 172: Significance Test Results for Catalepsy results between the untreated group(CU) and the MPTP disease induced and no treatment group(CM). As both the sets were non-normal but the variances were unequal, we used a Welch's T Test with ranked data. The p-value obtained for the test was 0.0000 and hence the difference between the data sets was considered statistically significant.

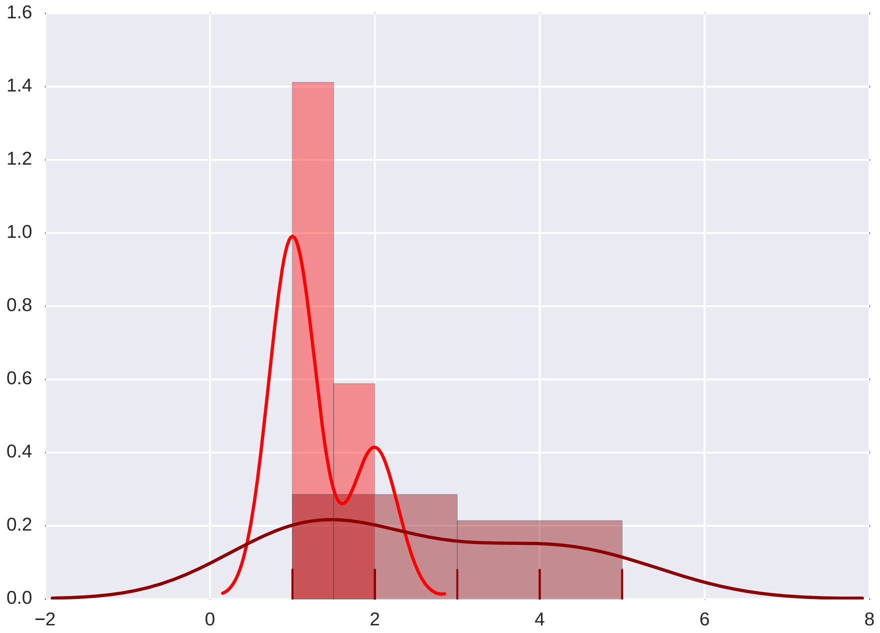

Figure 173: Significance Test Results for Catalepsy results between the untreated group(CU) and the MPTP disease induced and treated with *Centella asiatica* group(CMC). As both the sets were non-normal but the variances were unequal, we used a Welch's T Test with ranked data. The p-value obtained for the test was 0.0156 and hence the difference between the data sets was considered statistically significant.

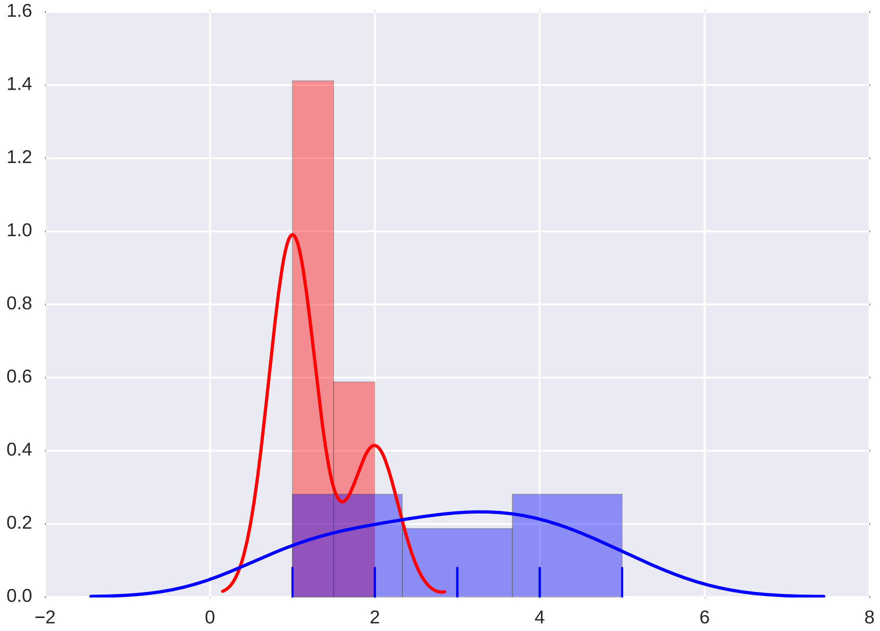

Figure 174: Significance Test Results for Catalepsy results between the untreated group(CU) and the MPTP disease induced and treated with *Withania somnifera* group(CMW). As one of the sets was non-normal but the variances were unequal, we used a Welch's T Test with ranked data. The p-value obtained for the test was 0.0001 and hence the difference between the data sets was considered statistically significant.

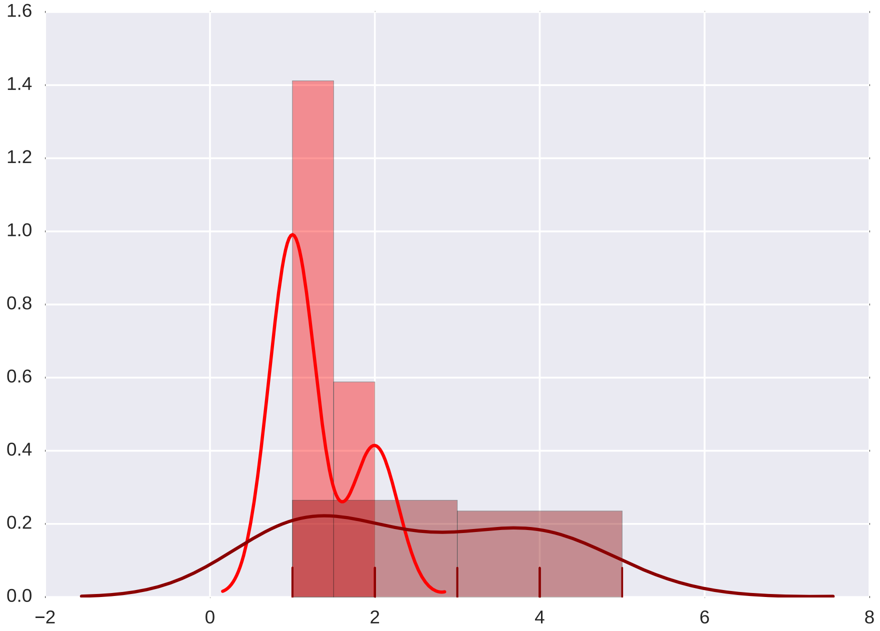

Figure 175: Significance Test Results for Catalepsy results between the untreated group(CU) and the MPTP disease induced and treated with both *Withania somnifera* and *Centella asiatica* group(CMWC). As both the sets were non-normal but the variances were unequal, we used a Welch's T Test with ranked data. The p-value obtained for the test was 0.0061 and hence the difference between the data sets was considered statistically significant.

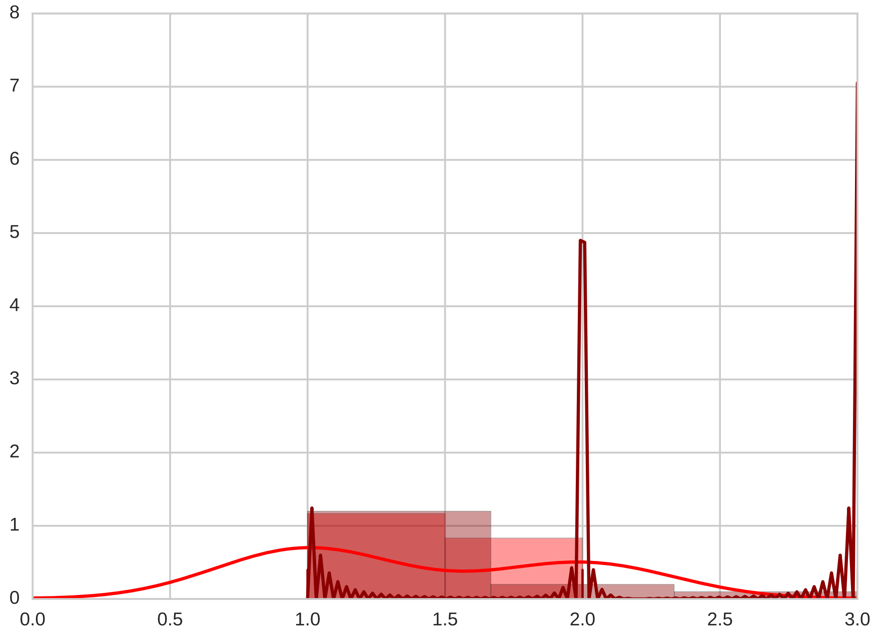

Figure 176: Significance Test Results for Catalepsy results between the group treated with *Centella asiatica*(CC) and the group treated with *Withania somnifera*(CW). As both the sets were non-normal, we used a Mann-Whitney U Test. The p-value obtained for the test was 0.3123 and hence the difference between the data sets was considered statistically non-significant.

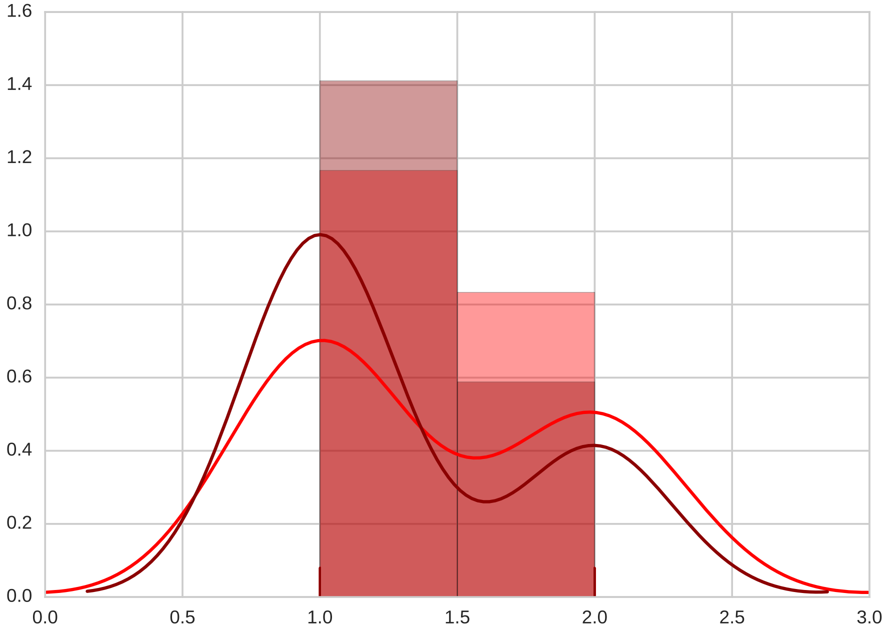

Figure 177: Significance Test Results for Catalepsy results between the group treated with *Centella asiatica*(CC) and the group treated with both *Withania somnifera* and *Centella asiatica*(CWC). As both the sets were non-normal, we used a Mann-Whitney U Test. The p-value obtained for the test was 0.5189 and hence the difference between the data sets was considered statistically non-significant.

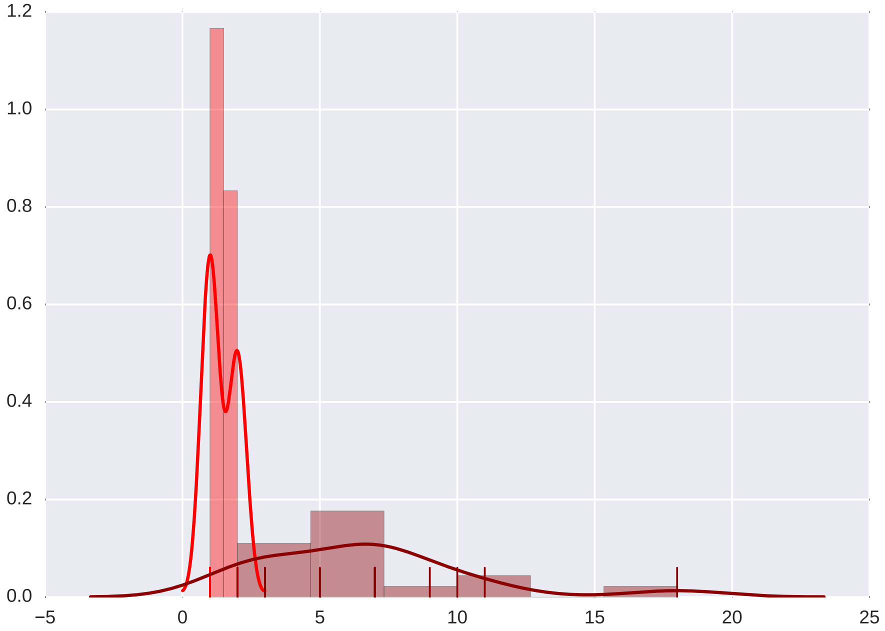

Figure 178: Significance Test Results for Catalepsy results between the group treated with *Centella asiatica*(CC) and the MPTP disease induced and no treatment group(CM). As both the sets were non-normal but the variances were unequal, we used a Welch's T Test with ranked data. The p-value obtained for the test was 0.0000 and hence the difference between the data sets was considered statistically significant.

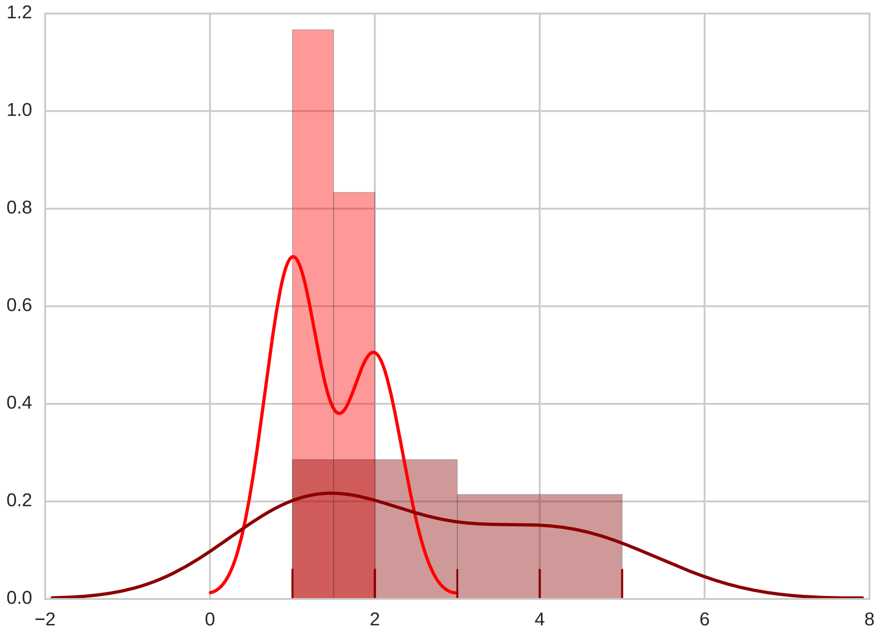

Figure 179: Significance Test Results for Catalepsy results between the group treated with *Centella asiatica*(CC) and the MPTP disease induced and treated with *Centella asiatica* group(CMC). As both the sets were non-normal but the variances were unequal, we used a Welch's T Test with ranked data. The p-value obtained for the test was 0.0528 and hence the difference between the data sets was considered statistically non-significant.

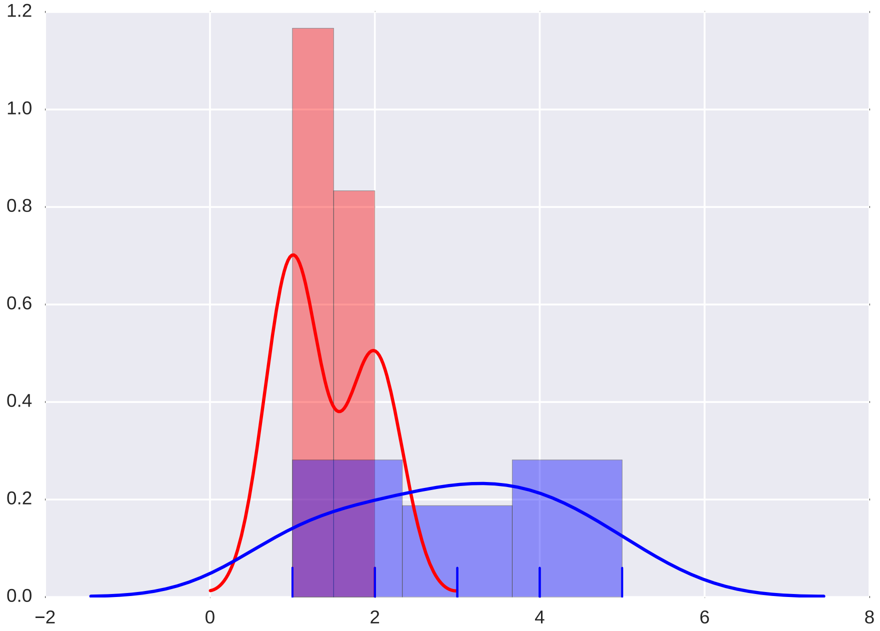

Figure 180: Significance Test Results for Catalepsy results between the group treated with *Centella asiatica*(CC) and the MPTP disease induced and treated with *Withania somnifera* group(CMW). As one of the sets was non-normal but the variances were unequal, we used a Welch's T Test with ranked data. The p-value obtained for the test was 0.0006 and hence the difference between the data sets was considered statistically significant.

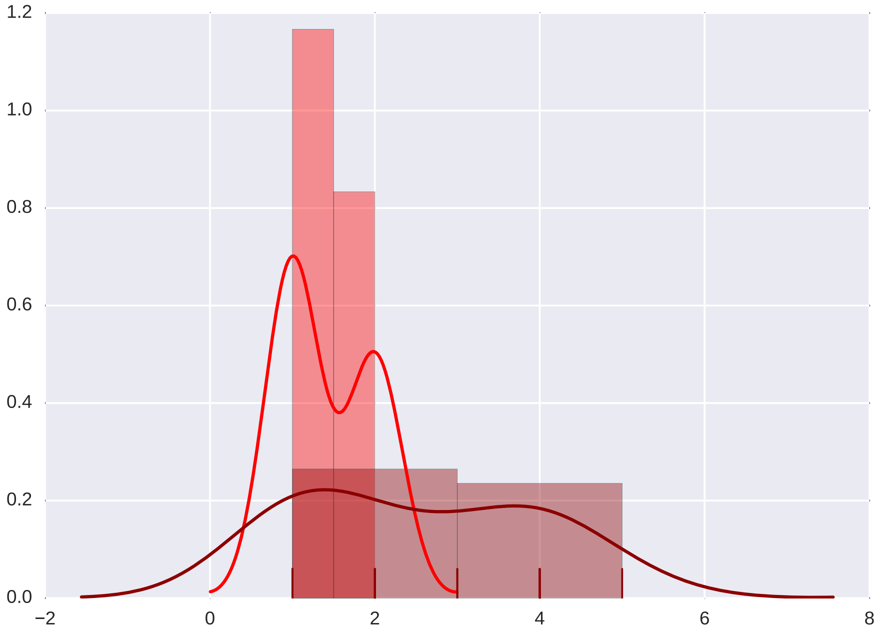

Figure 181: Significance Test Results for Catalepsy results between the group treated with *Centella asiatica*(CC) and the MPTP disease induced and treated with both *Withania somnifera* and *Centella asiatica* group(CMWC). As both the sets were non-normal but the variances were unequal, we used a Welch's T Test with ranked data. The p-value obtained for the test was 0.0258 and hence the difference between the data sets was considered statistically significant.

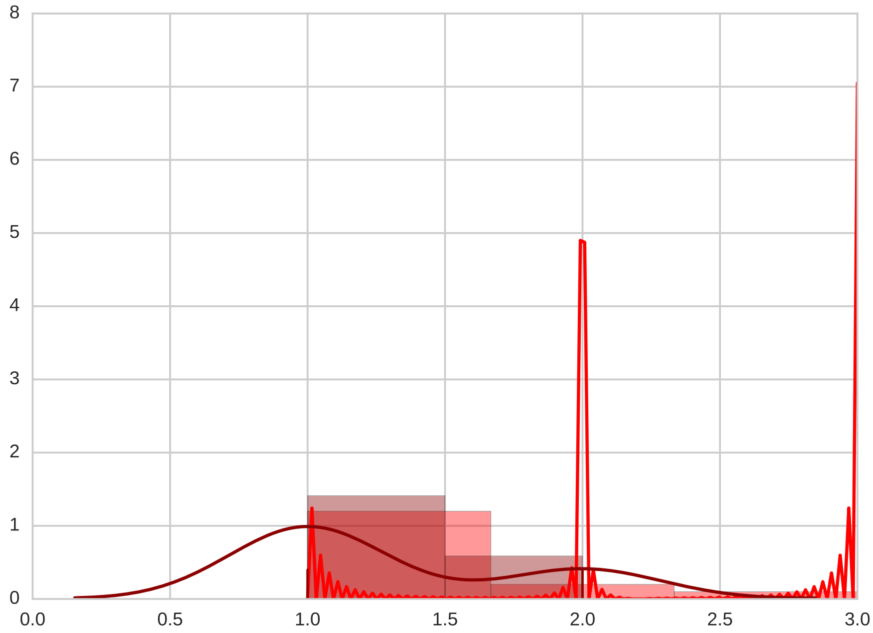

Figure 182: Significance Test Results for Catalepsy results between the group treated with *Withania somnifera*(CW) and the group treated with both *Withania somnifera* and *Centella asiatica*(CWC). As both the sets were non-normal, we used a Mann-Whitney U Test. The p-value obtained for the test was 0.6521 and hence the difference between the data sets was considered statistically non-significant.

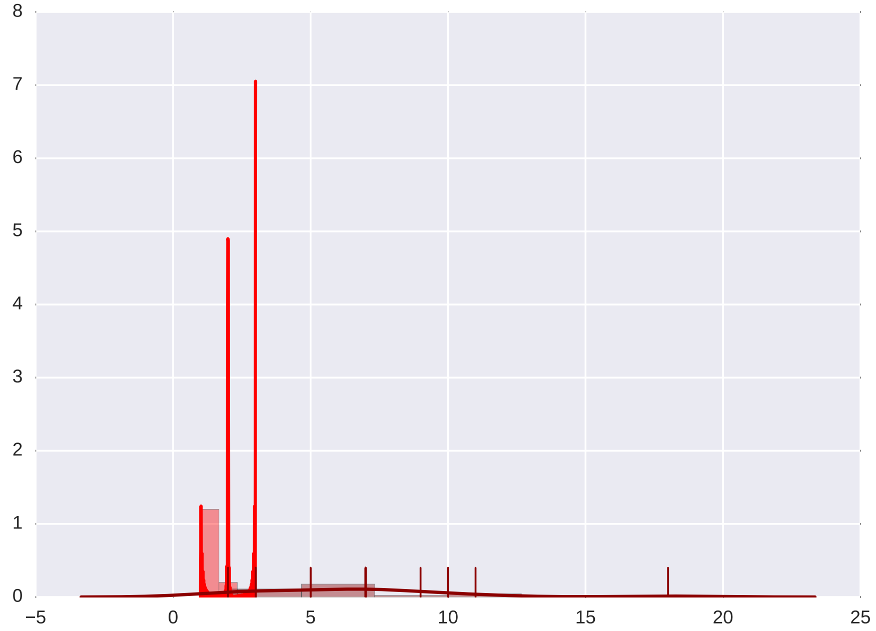

Figure 183: Significance Test Results for Catalepsy results between the group treated with *Withania somnifera*(CW) and the MPTP disease induced and no treatment group(CM). As both the sets were non-normal but the variances were unequal, we used a Welch's T Test with ranked data. The p-value obtained for the test was 0.0000 and hence the difference between the data sets was considered statistically significant.

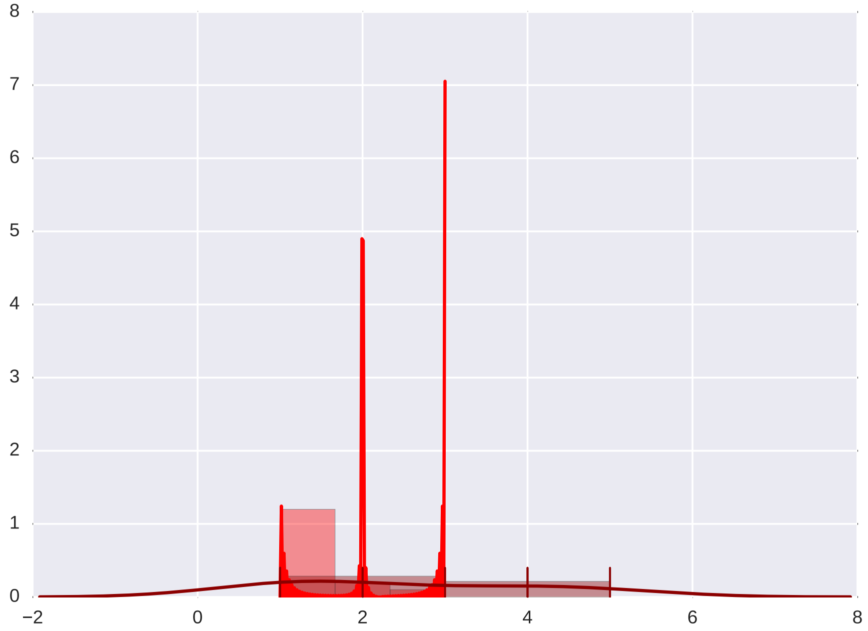

Figure 184: Significance Test Results for Catalepsy results between the group treated with *Withania somnifera*(CW) and the MPTP disease induced and treated with *Centella asiatica* group(CMC). As both the sets were non-normal but the variances were unequal, we used a Welch's T Test with ranked data. The p-value obtained for the test was 0.0080 and hence the difference between the data sets was considered statistically significant.

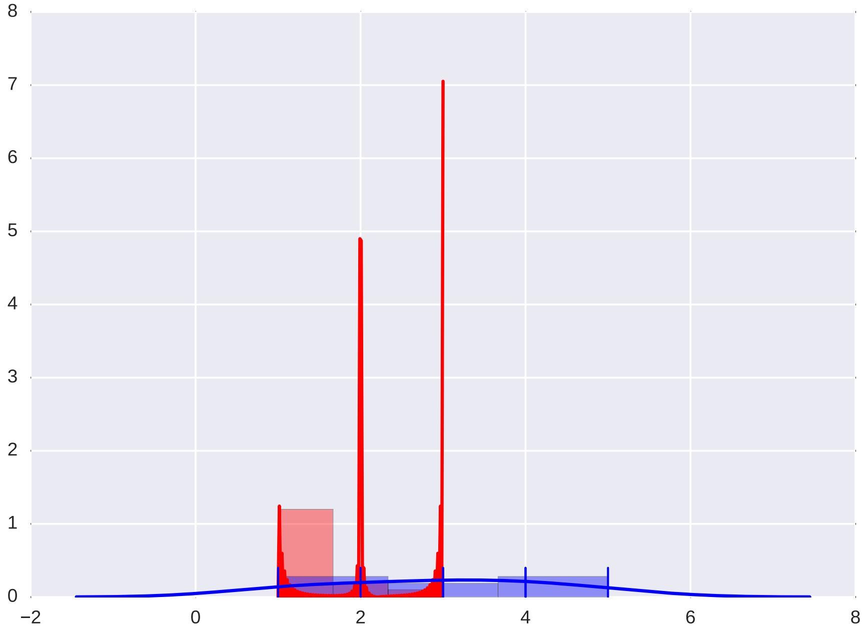

Figure 185: Significance Test Results for Catalepsy results between the group treated with *Withania somnifera*(CW) and the MPTP disease induced and treated with *Withania somnifera* group(CMW). As one of the sets was non-normal but the variances were unequal, we used a Welch's T Test with ranked data. The p-value obtained for the test was 0.0001 and hence the difference between the data sets was considered statistically significant.

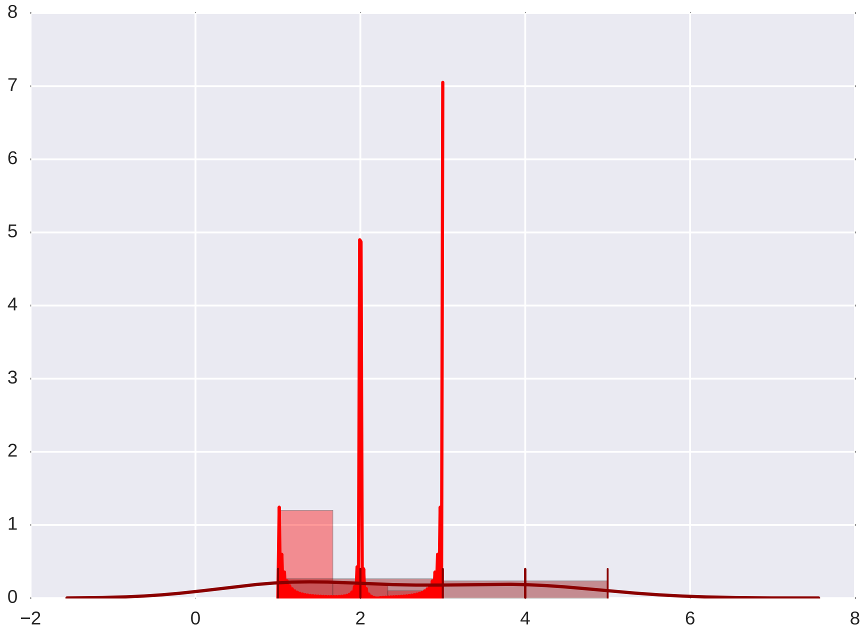

Figure 186: Significance Test Results for Catalepsy results between the group treated with *Withania somnifera*(CW) and the MPTP disease induced and treated with both *Withania somnifera* and *Centella asiatica* group(CMWC). As both the sets were non-normal but the variances were unequal, we used a Welch's T Test with ranked data. The p-value obtained for the test was 0.0031 and hence the difference between the data sets was considered statistically significant.

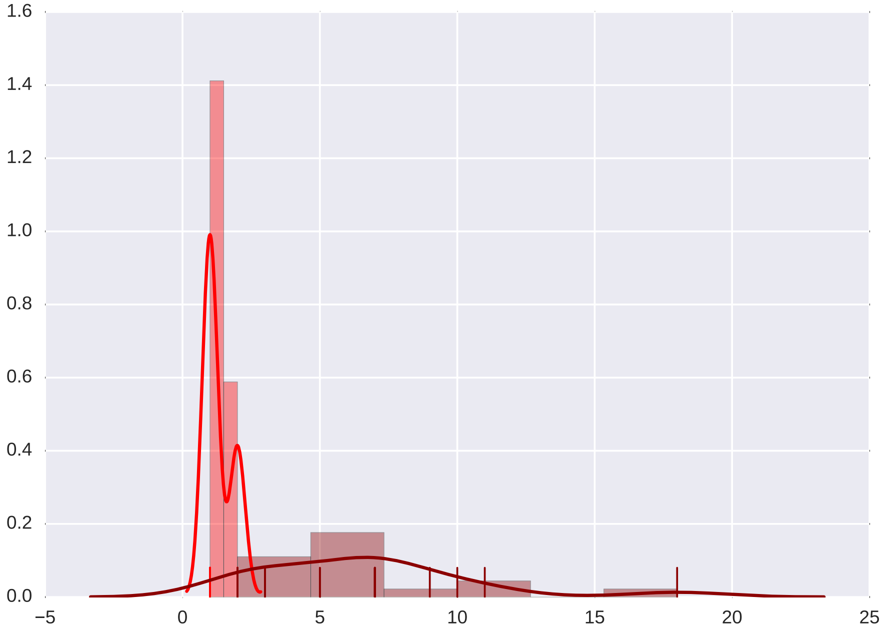

Figure 187: Significance Test Results for Catalepsy results between the group treated with both *Withania somnifera* and *Centella asiatica*(CWC) and the MPTP disease induced and no treatment group(CM). As both the sets were non-normal but the variances were unequal, we used a Welch's T Test with ranked data. The p-value obtained for the test was 0.0000 and hence the difference between the data sets was considered statistically significant.

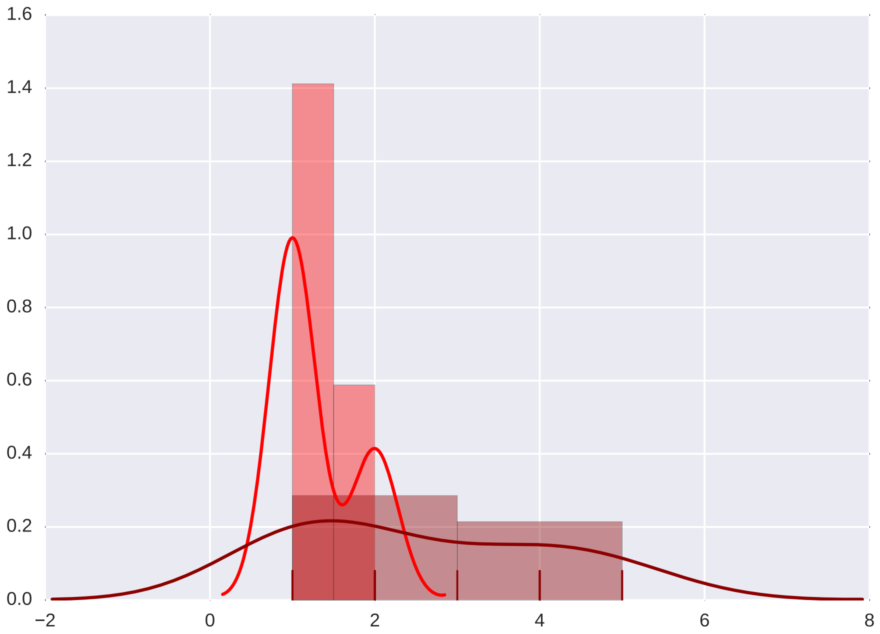

Figure 188: Significance Test Results for Catalepsy results between the group treated with both *Withania somnifera* and *Centella asiatica*(CWC) and the MPTP disease induced and treated with *Centella asiatica* group(CMC). As both the sets were non-normal but the variances were unequal, we used a Welch's T Test with ranked data. The p-value obtained for the test was 0.0156 and hence the difference between the data sets was considered statistically significant.

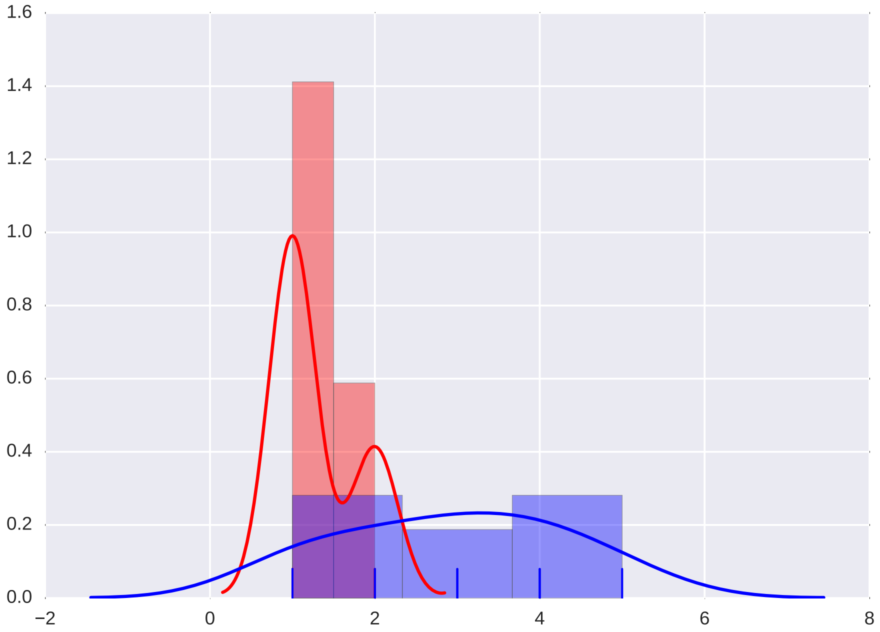

Figure 189: Significance Test Results for Catalepsy results between the group treated with both *Withania somnifera* and *Centella asiatica*(CWC) and the MPTP disease induced and treated with *Withania somnifera* group(CMW). As one of the sets was non-normal but the variances were unequal, we used a Welch's T Test with ranked data. The p-value obtained for the test was 0.0001 and hence the difference between the data sets was considered statistically significant.

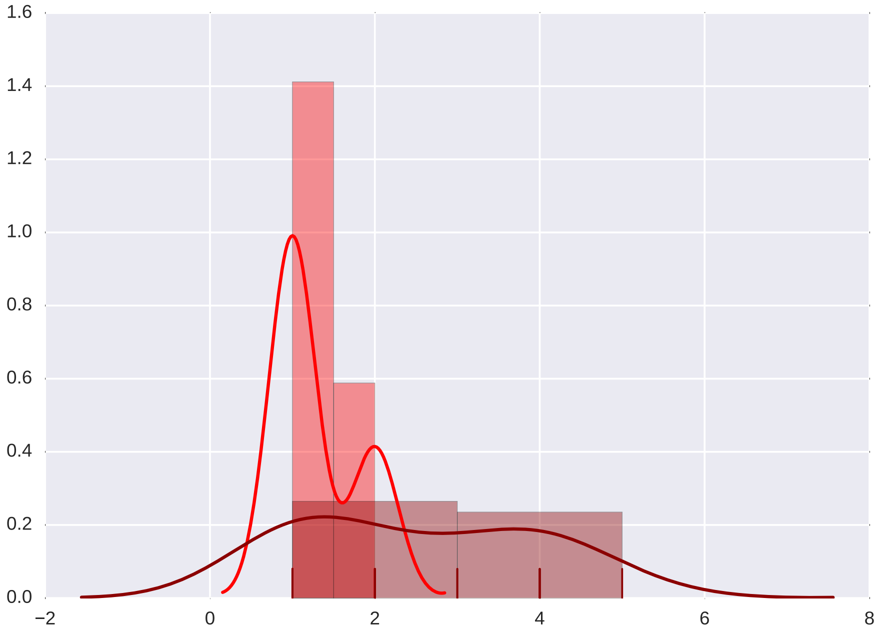

Figure 190: Significance Test Results for Catalepsy results between the group treated with both *Withania somnifera* and *Centella asiatica*(CWC) and the MPTP disease induced and treated with both *Withania somnifera* and *Centella asiatica* group(CMWC). As both the sets were non-normal but the variances were unequal, we used a Welch's T Test with ranked data. The p-value obtained for the test was 0.0061 and hence the difference between the data sets was considered statistically significant.

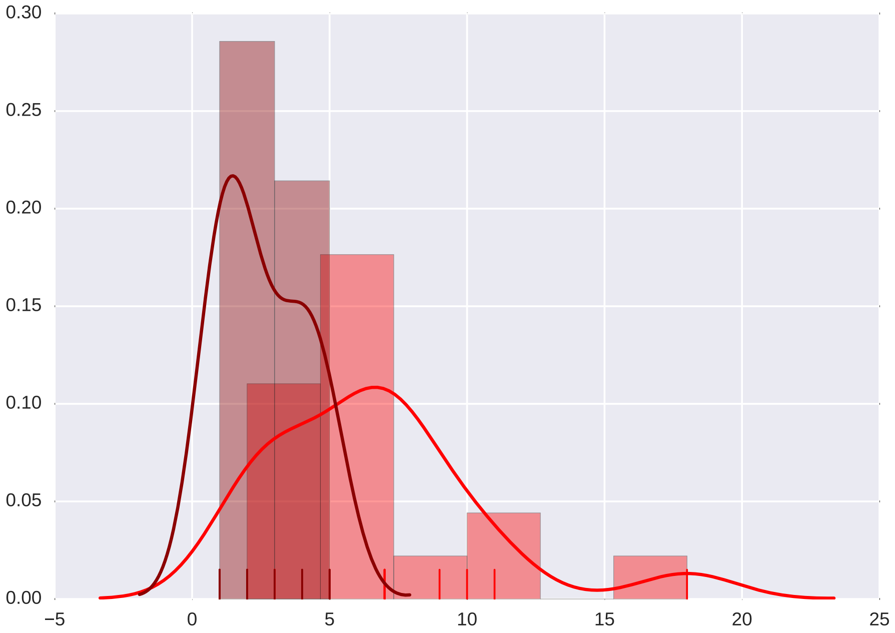

Figure 191: Significance Test Results for Catalepsy results between the MPTP disease induced and no treatment group(CM) and the MPTP disease induced and treated with *Centella asiatica* group(CMC). As both the sets were non-normal, we used a Mann-Whitney U Test. The p-value obtained for the test was 0.0007 and hence the difference between the data sets was considered statistically significant.

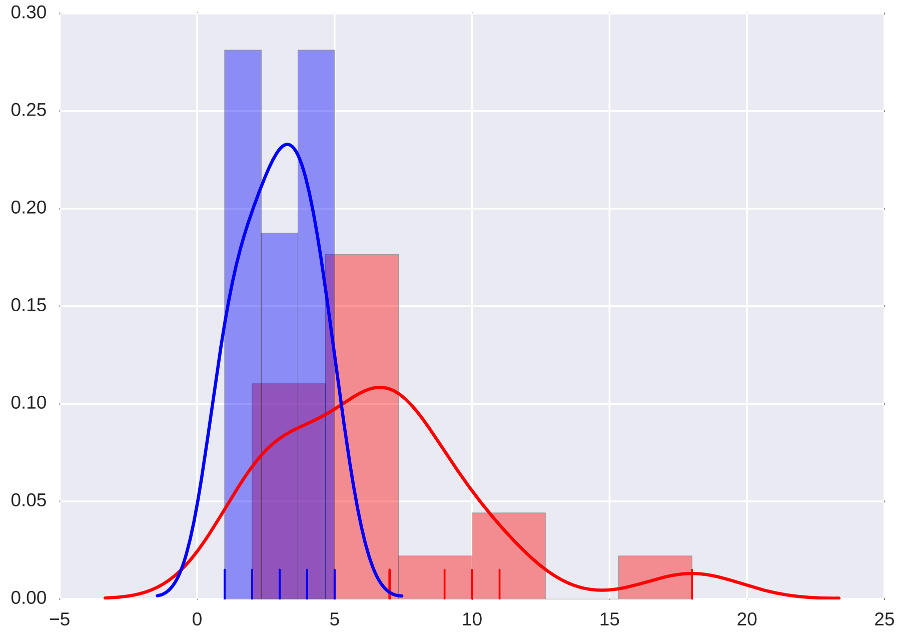

Figure 192: Significance Test Results for Catalepsy results between the MPTP disease induced and no treatment group(CM) and the MPTP disease induced and treated with *Withania somnifera* group(CMW). As one of the sets was non-normal but the variances were unequal, we used a Welch's T Test with ranked data. The p-value obtained for the test was 0.0007 and hence the difference between the data sets was considered statistically significant.

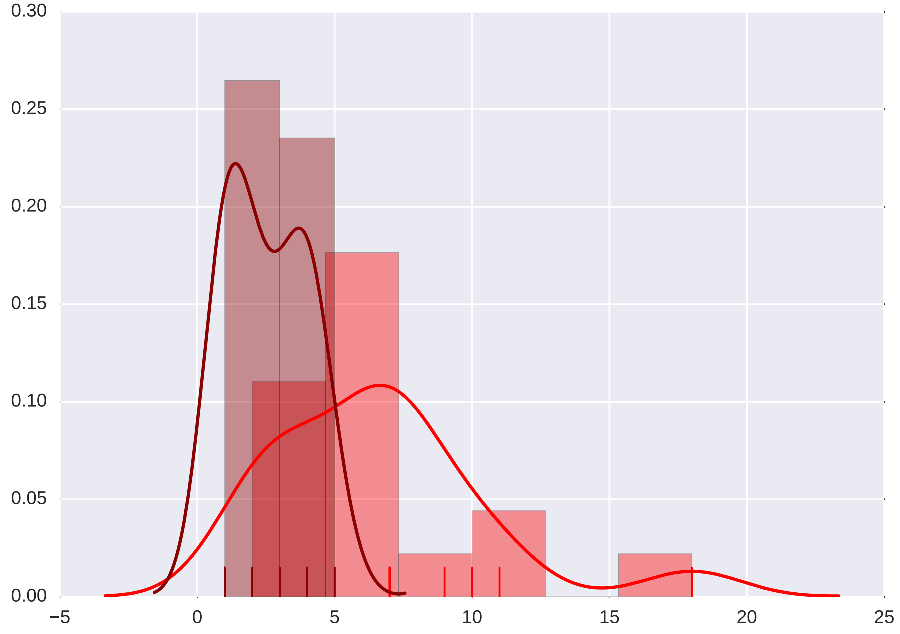

Figure 193: Significance Test Results for Catalepsy results between the MPTP disease induced and no treatment group(CM) and the MPTP disease induced and treated with both *Withania somnifera* and *Centella asiatica* group(CMWC). As both the sets were non-normal, we used a Mann-Whitney U Test. The p-value obtained for the test was 0.0003 and hence the difference between the data sets was considered statistically significant.

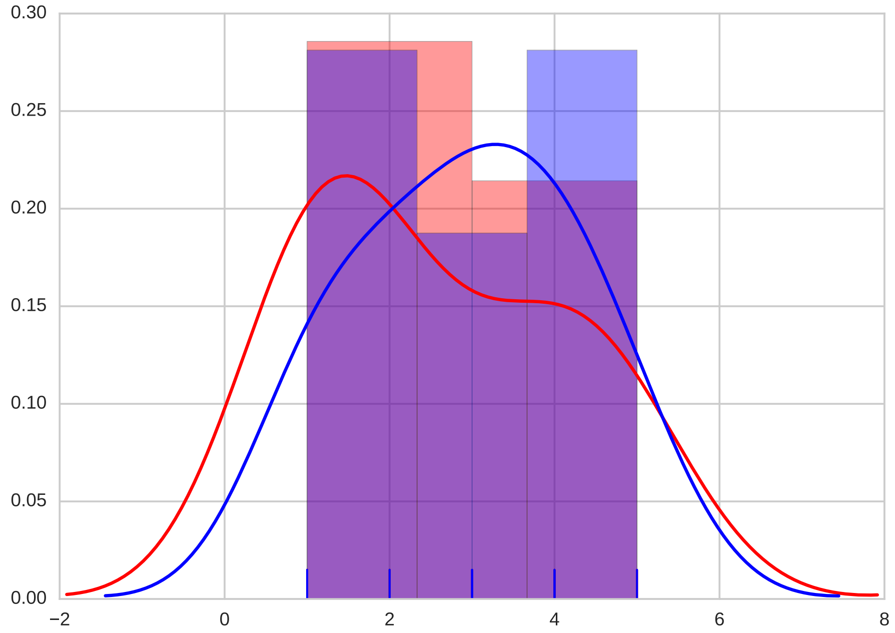

Figure 194: Significance Test Results for Catalepsy results between the MPTP disease induced and treated with *Centella asiatica* group(CMC) and the MPTP disease induced and treated with *Withania somnifera* group(CMW). As one of the sets was non-normal, we used a Mann-Whitney U Test. The p-value obtained for the test was 0.4826 and hence the difference between the data sets was considered statistically non-significant.

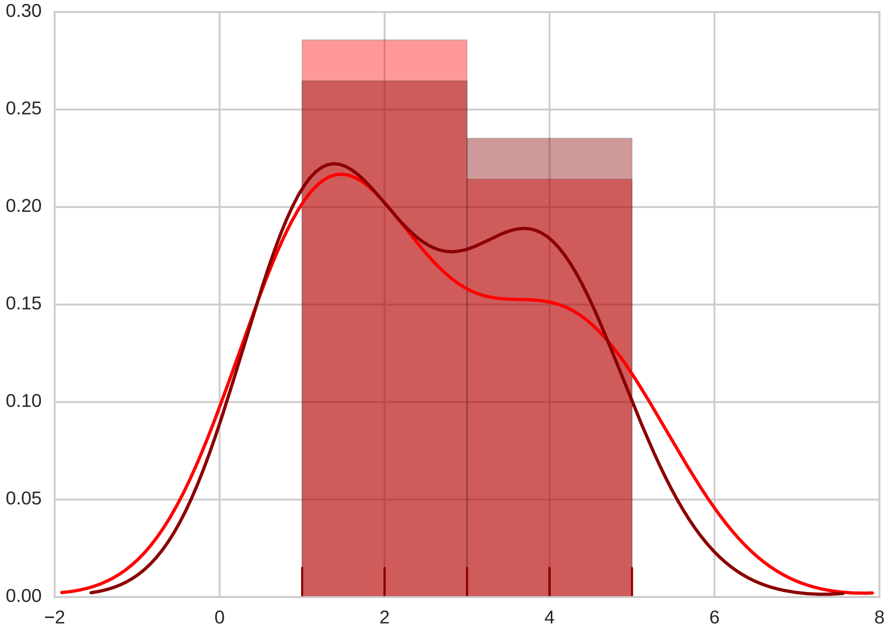

Figure 195: Significance Test Results for Catalepsy results between the MPTP disease induced and treated with *Centella asiatica* group(CMC) and the MPTP disease induced and treated with both *Withania somnifera* and *Centella asiatica* group(CMWC). As both the sets were non-normal, we used a Mann-Whitney U Test. The p-value obtained for the test was 0.9508 and hence the difference between the data sets was considered statistically non-significant.

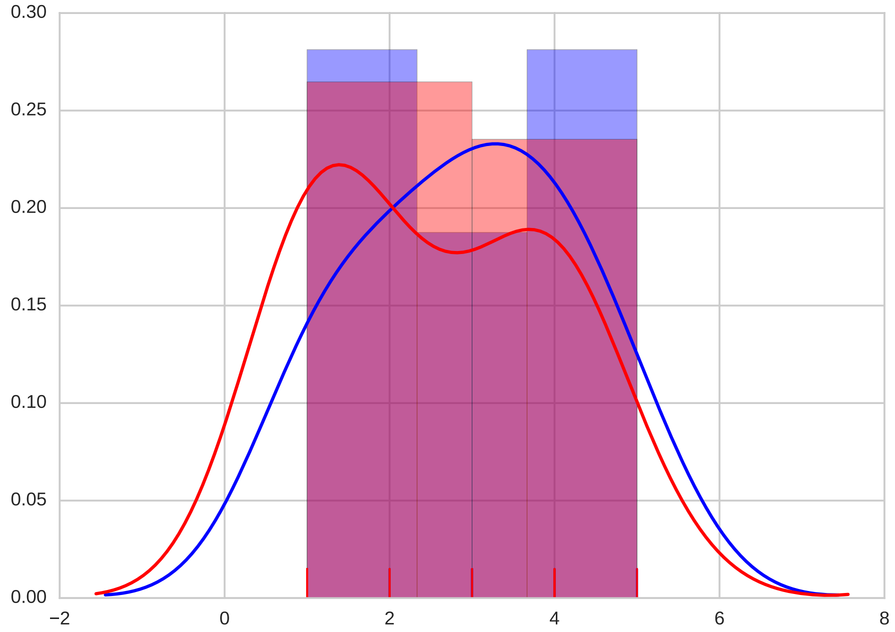

Figure 196: Significance Test Results for Catalepsy results between the MPTP disease induced and treated with *Withania somnifera* group(CMW) and the MPTP disease induced and treated with both *Withania somnifera* and *Centella asiatica* group(CMWC). As one of the sets was non-normal, we used a Mann-Whitney U Test. The p-value obtained for the test was 0.4156 and hence the difference between the data sets was considered statistically non-significant.

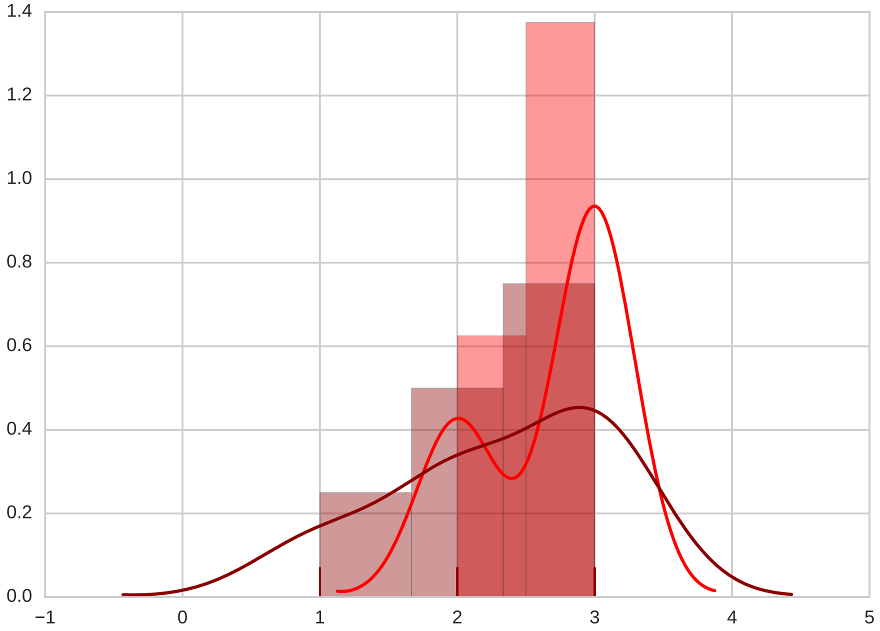

Figure 197: Significance Test Results for Swim-test results between the untreated group(SU) and the group treated with *Centella asiatica*(SC). As both the sets were non-normal but the variances were unequal, we used a Welch's T Test with ranked data. The p-value obtained for the test was 0.2415 and hence the difference between the data sets was considered statistically non-significant.

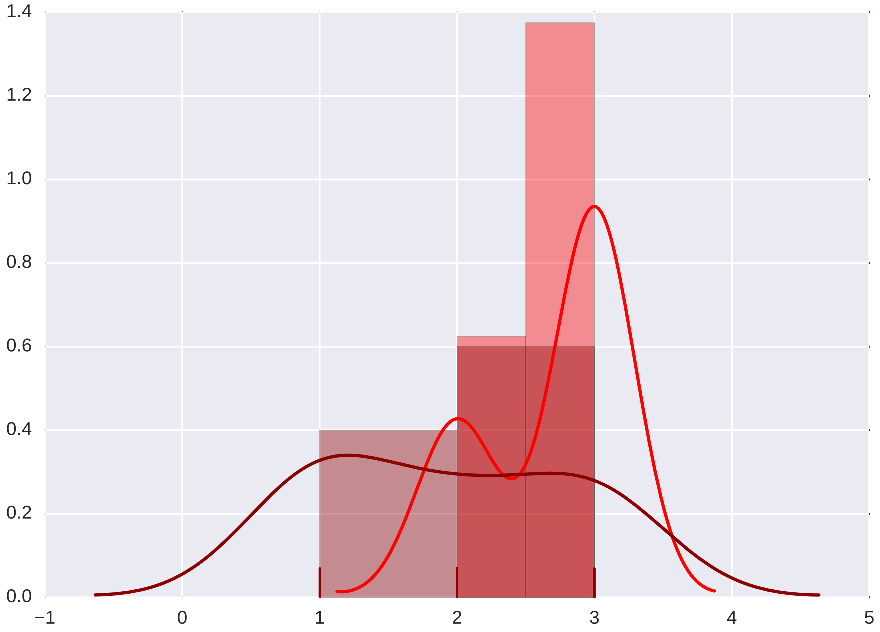

Figure 198: Significance Test Results for Swim-test results between the untreated group(SU) and the group treated with *Withania somnifera*(SW). As both the sets were non-normal but the variances were unequal, we used a Welch's T Test with ranked data. The p-value obtained for the test was 0.0119 and hence the difference between the data sets was considered statistically significant.

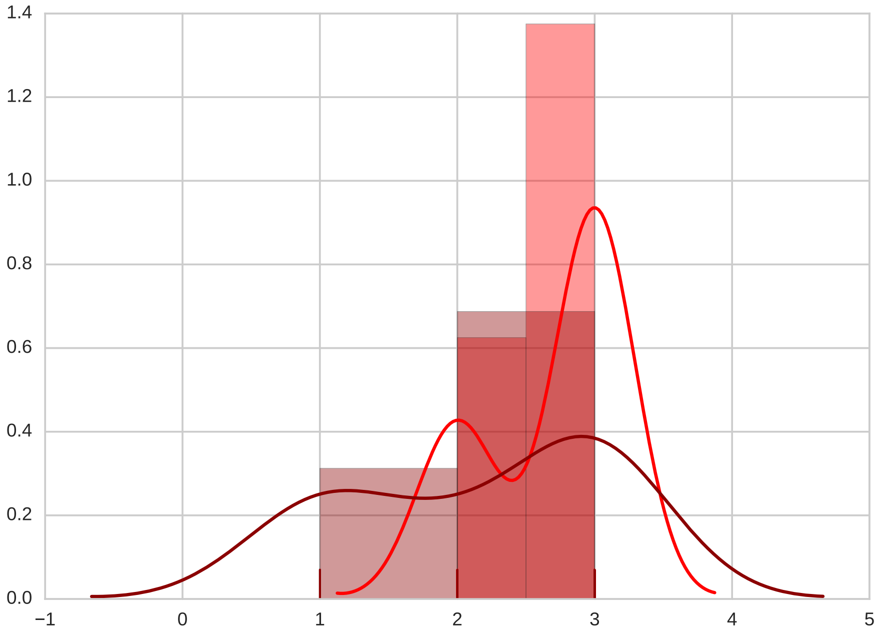

Figure 199: Significance Test Results for Swim-test results between the untreated group(SU) and the group treated with both *Withania somnifera* and *Centella asiatica*(SWC). As both the sets were non-normal but the variances were unequal, we used a Welch's T Test with ranked data. The p-value obtained for the test was 0.1203 and hence the difference between the data sets was considered statistically non-significant.

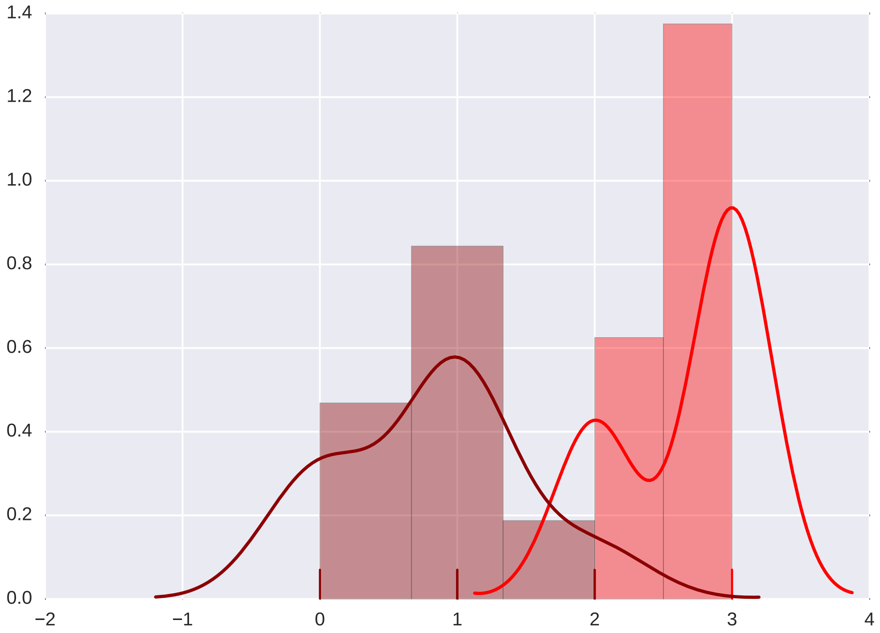

Figure 200: Significance Test Results for Swim-test results between the untreated group(SU) and the MPTP disease induced and no treatment group(SM). As both the sets were non-normal, we used a Mann-Whitney U Test. The p-value obtained for the test was 0.0000 and hence the difference between the data sets was considered statistically significant.

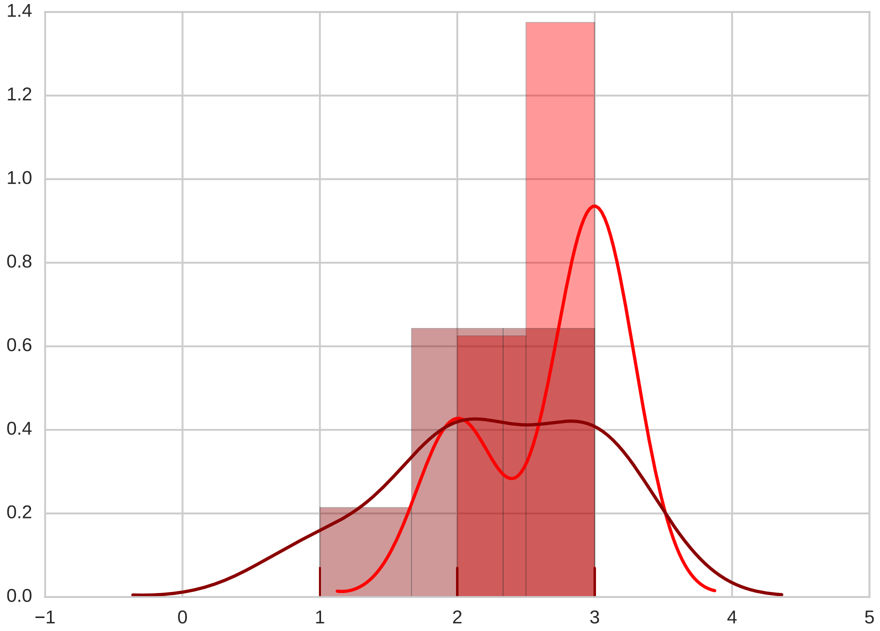

Figure 201: Significance Test Results for Swim-test results between the untreated group(SU) and the MPTP disease induced and treated with *Centella asiatica* group(SMC). As both the sets were non-normal, we used a Mann-Whitney U Test. The p-value obtained for the test was 0.1123 and hence the difference between the data sets was considered statistically non-significant.

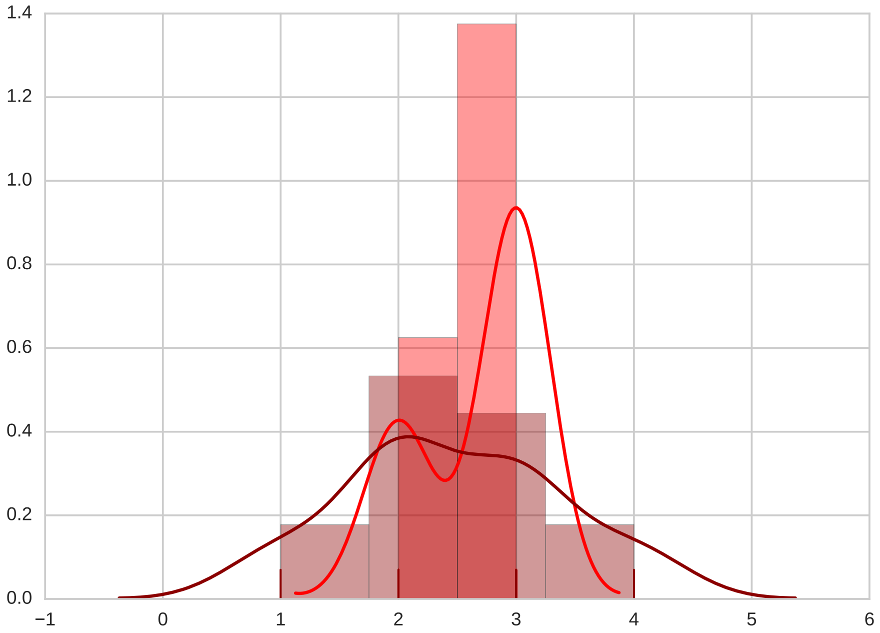

Figure 202: Significance Test Results for Swim-test results between the untreated group(SU) and the MPTP disease induced and treated with *Withania somnifera* group(SMW). As both the sets were non-normal, we used a Mann-Whitney U Test. The p-value obtained for the test was 0.3821 and hence the difference between the data sets was considered statistically non-significant.

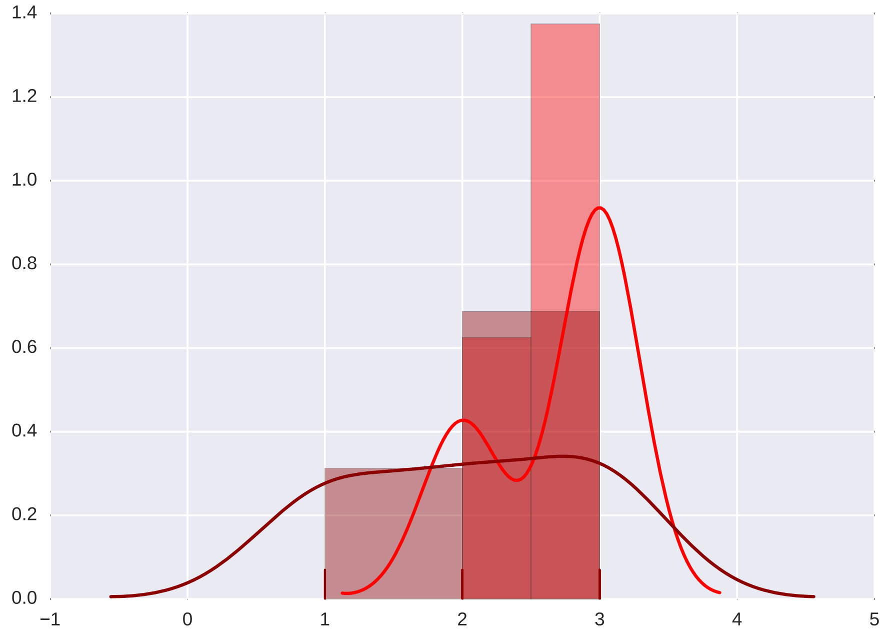

Figure 203: Significance Test Results for Swim-test results between the untreated group(SU) and the MPTP disease induced and treated with both *Withania somnifera* and *Centella asiatica* group(SMWC). As both the sets were non-normal but the variances were unequal, we used a Welch's T Test with ranked data. The p-value obtained for the test was 0.0268 and hence the difference between the data sets was considered statistically significant.

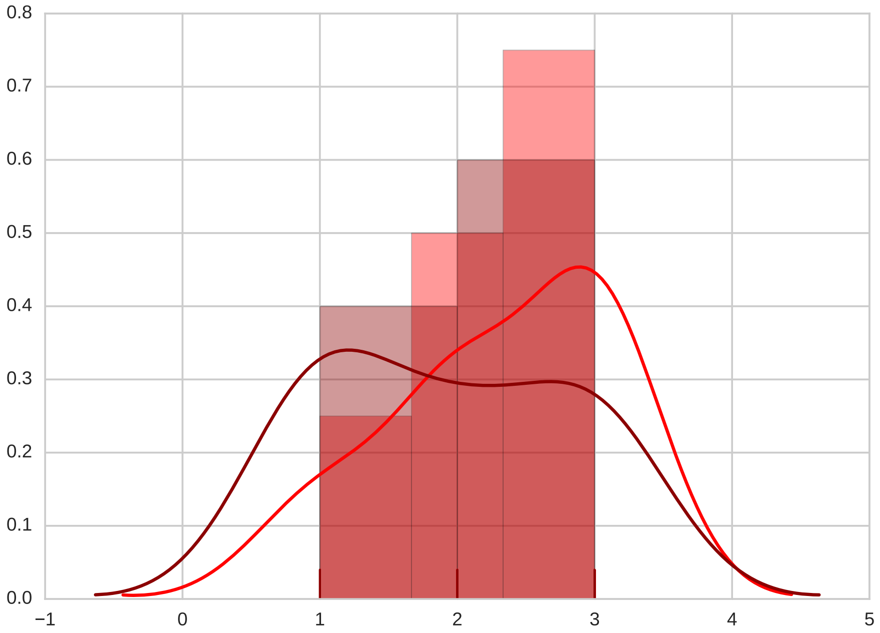

Figure 204: Significance Test Results for Swim-test results between the group treated with *Centella asiatica*(SC) and the group treated with *Withania somnifera*(SW). As both the sets were non-normal, we used a Mann-Whitney U Test. The p-value obtained for the test was 0.2423 and hence the difference between the data sets was considered statistically non-significant.

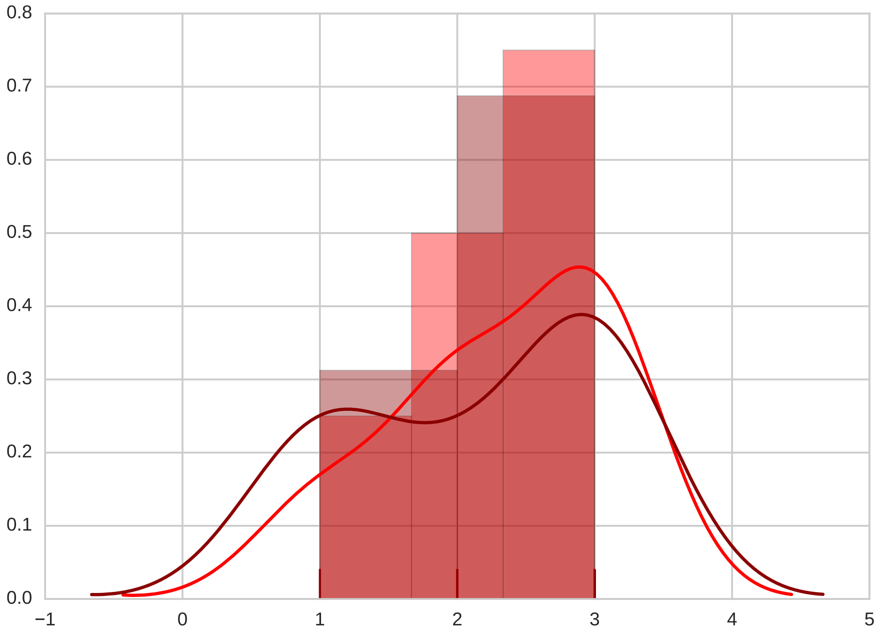

Figure 205: Significance Test Results for Swim-test results between the group treated with *Centella asiatica*(SC) and the group treated with both *Withania somnifera* and *Centella asiatica*(SWC). As both the sets were non-normal, we used a Mann-Whitney U Test. The p-value obtained for the test was 0.7427 and hence the difference between the data sets was considered statistically non-significant.

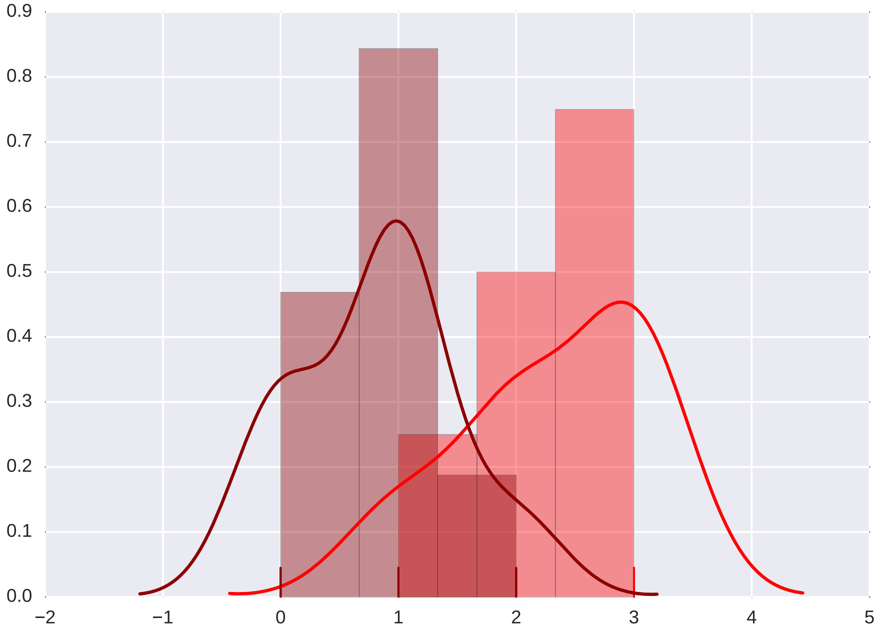

Figure 206: Significance Test Results for Swim-test results between the group treated with *Centella asiatica*(SC) and the MPTP disease induced and no treatment group(SM). As both the sets were non-normal, we used a Mann-Whitney U Test. The p-value obtained for the test was 0.0001 and hence the difference between the data sets was considered statistically significant.

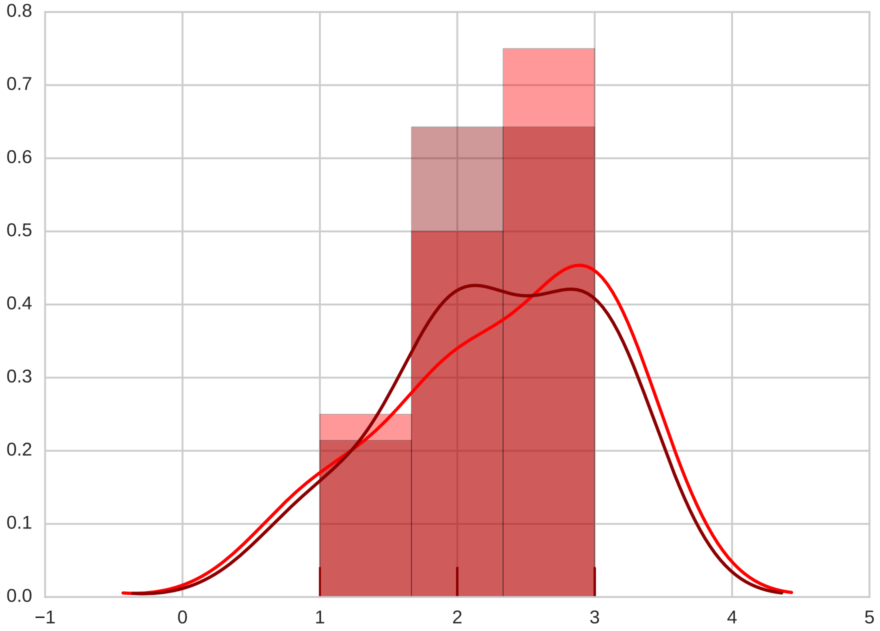

Figure 207: Significance Test Results for Swim-test results between the group treated with *Centella asiatica*(SC) and the MPTP disease induced and treated with *Centella asiatica* group(SMC). As both the sets were non-normal, we used a Mann-Whitney U Test. The p-value obtained for the test was 0.8445 and hence the difference between the data sets was considered statistically non-significant.

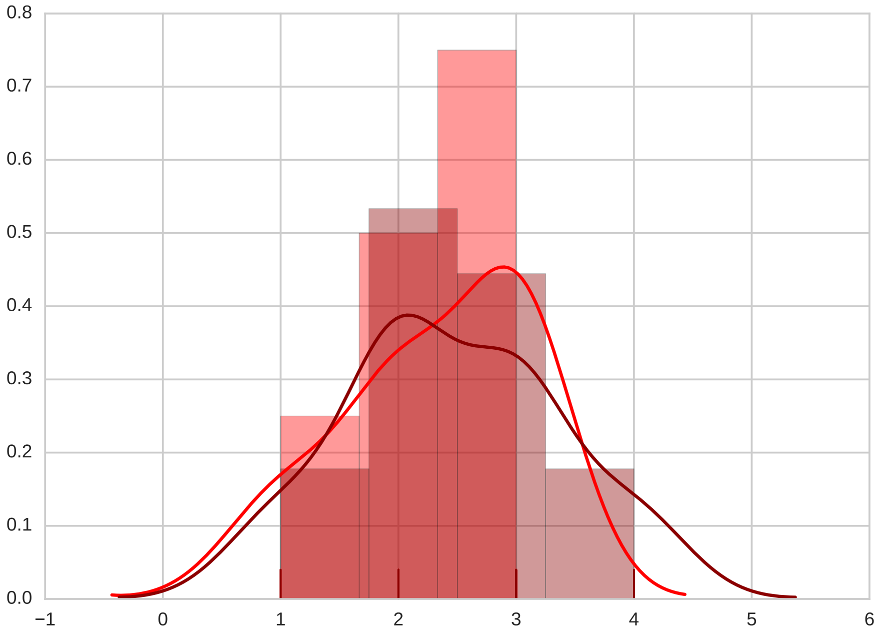

Figure 208: Significance Test Results for Swim-test results between the group treated with *Centella asiatica*(SC) and the MPTP disease induced and treated with *Withania somnifera* group(SMW). As both the sets were non-normal, we used a Mann-Whitney U Test. The p-value obtained for the test was 0.8148 and hence the difference between the data sets was considered statistically non-significant.

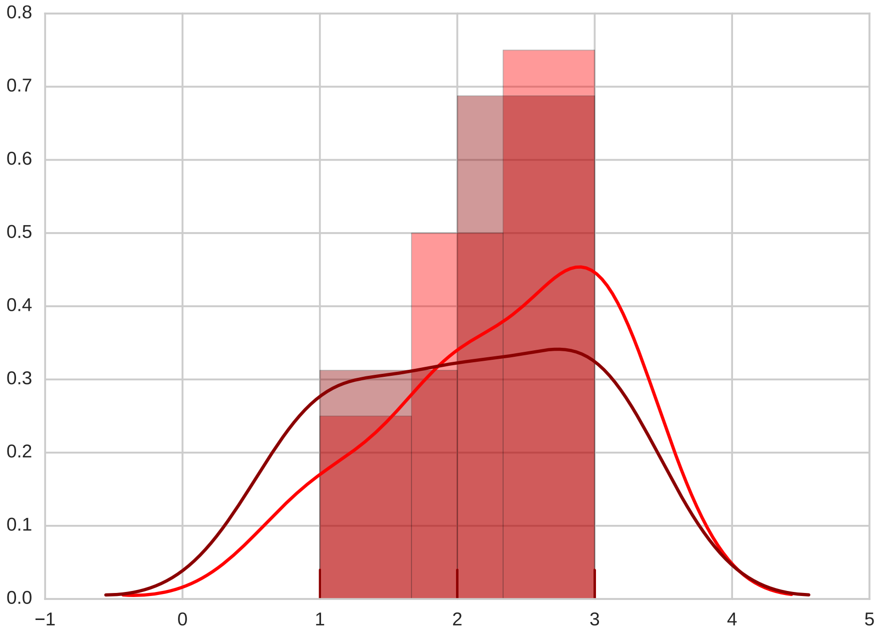

Figure 209: Significance Test Results for Swim-test results between the group treated with *Centella asiatica*(SC) and the MPTP disease induced and treated with both *Withania somnifera* and *Centella asiatica* group(SMWC). As both the sets were non-normal, we used a Mann-Whitney U Test. The p-value obtained for the test was 0.4125 and hence the difference between the data sets was considered statistically non-significant.

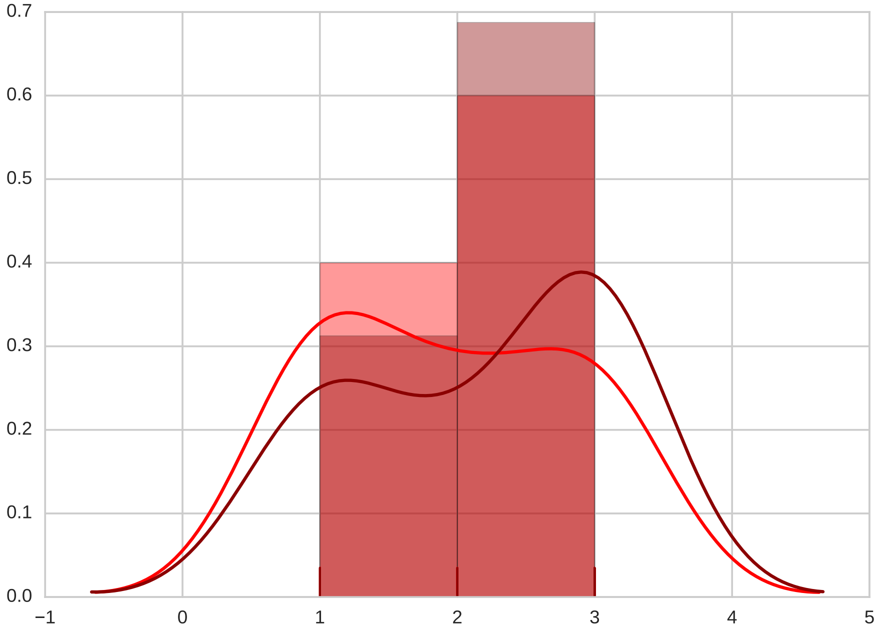

Figure 210: Significance Test Results for Swim-test results between the group treated with *Withania somnifera*(SW) and the group treated with both *Withania somnifera* and *Centella asiatica*(SWC). As both the sets were non-normal, we used a Mann-Whitney U Test. The p-value obtained for the test was 0.4333 and hence the difference between the data sets was considered statistically non-significant.

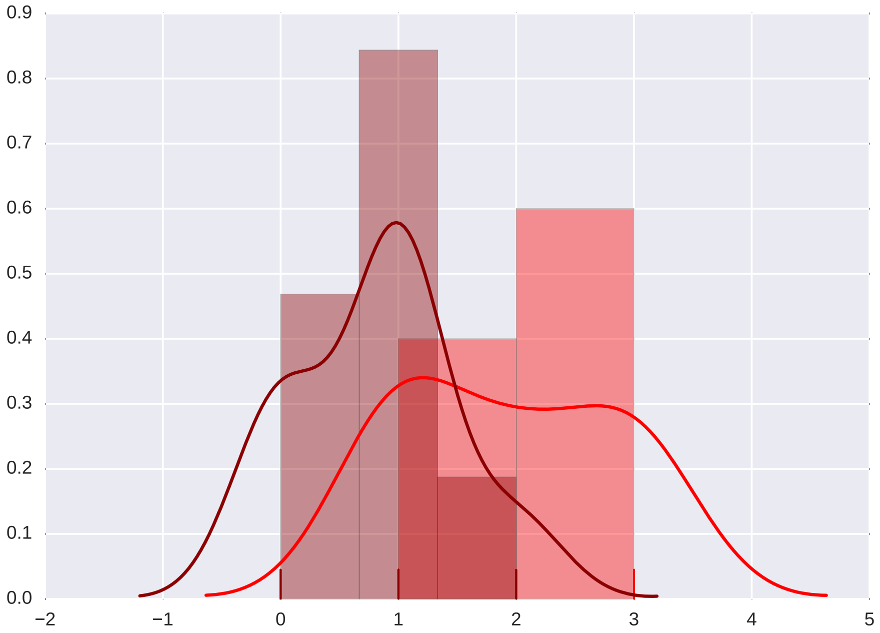

Figure 211: Significance Test Results for Swim-test results between the group treated with *Withania somnifera*(SW) and the MPTP disease induced and no treatment group(SM). As both the sets were non-normal, we used a Mann-Whitney U Test. The p-value obtained for the test was 0.0012 and hence the difference between the data sets was considered statistically significant.

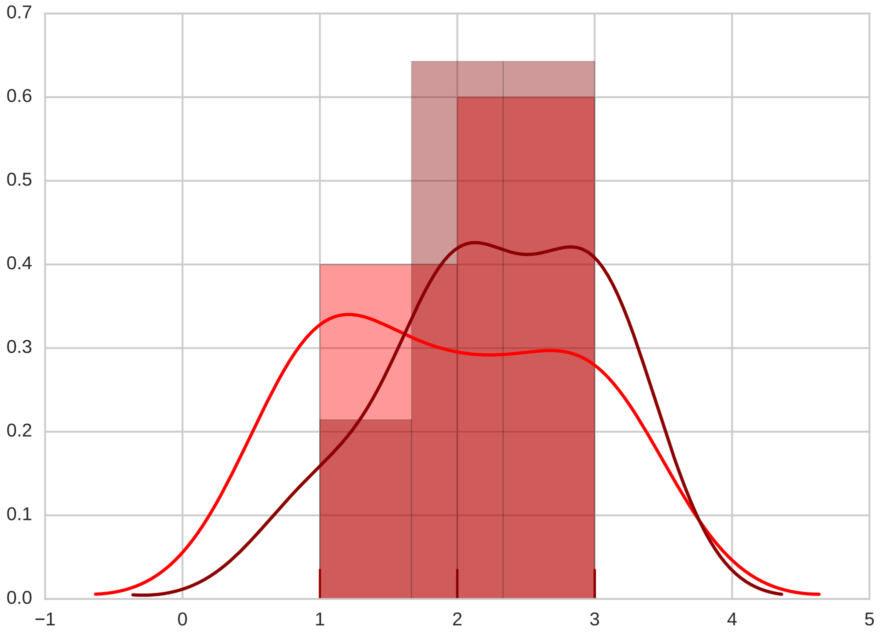

Figure 212: Significance Test Results for Swim-test results between the group treated with *Withania somnifera*(SW) and the MPTP disease induced and treated with *Centella asiatica* group(SMC). As both the sets were non-normal, we used a Mann-Whitney U Test. The p-value obtained for the test was 0.2755 and hence the difference between the data sets was considered statistically non-significant.

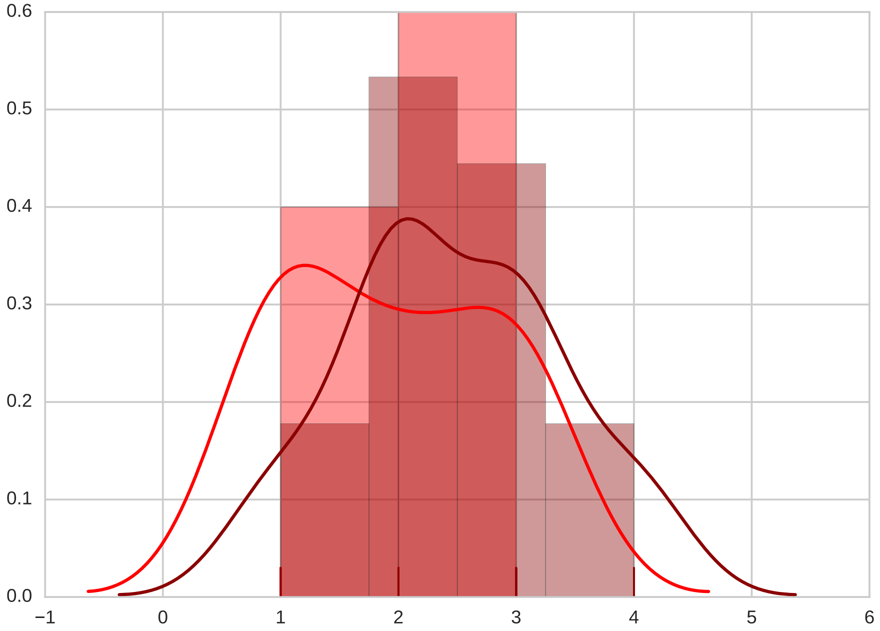

Figure 213: Significance Test Results for Swim-test results between the group treated with *Withania somnifera*(SW) and the MPTP disease induced and treated with *Withania somnifera* group(SMW). As both the sets were non-normal, we used a Mann-Whitney U Test. The p-value obtained for the test was 0.1447 and hence the difference between the data sets was considered statistically non-significant.

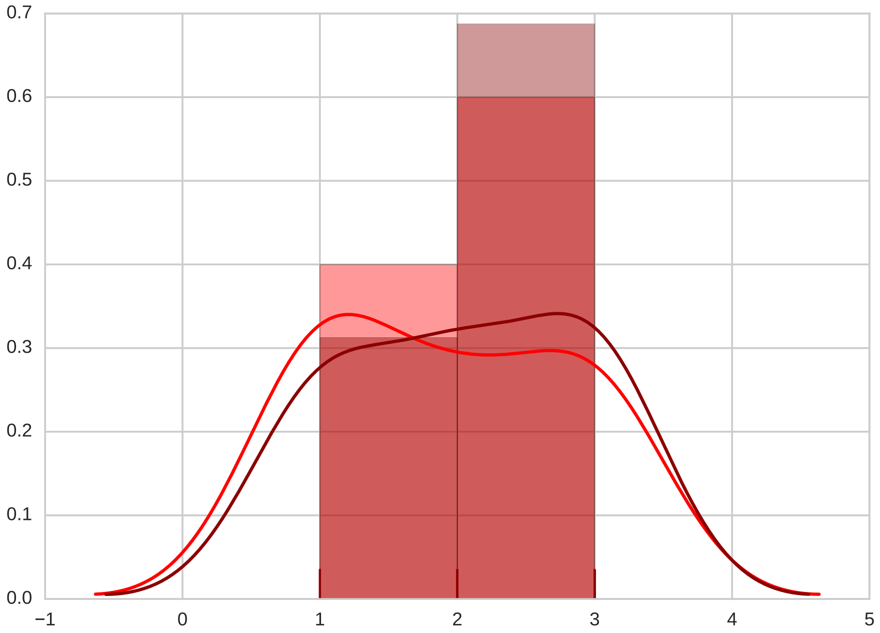

Figure 214: Significance Test Results for Swim-test results between the group treated with *Withania somnifera*(SW) and the MPTP disease induced and treated with both *Withania somnifera* and *Centella asiatica* group(SMWC). As both the sets were non-normal, we used a Mann-Whitney U Test. The p-value obtained for the test was 0.6901 and hence the difference between the data sets was considered statistically non-significant.

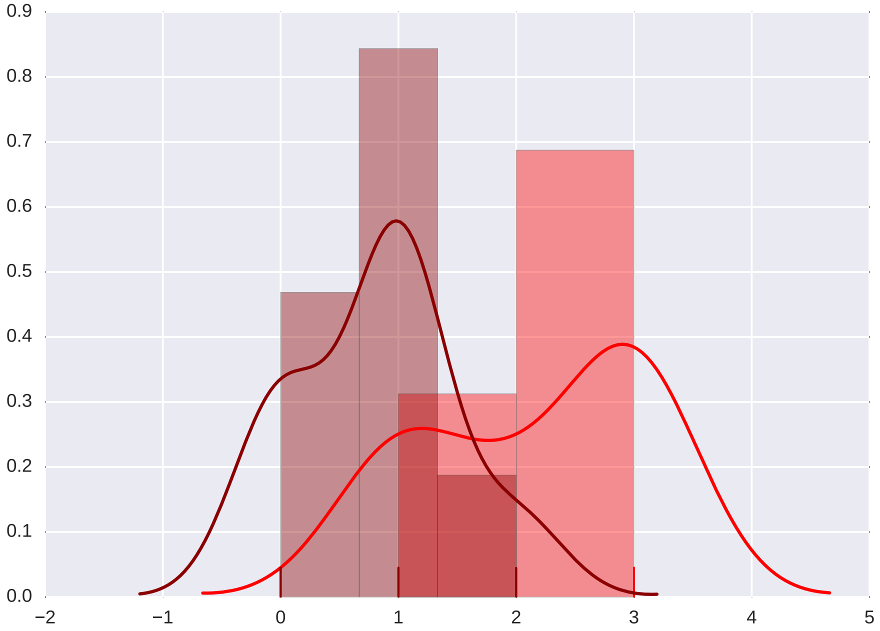

Figure 215: Significance Test Results for Swim-test results between the group treated with both *Withania somnifera* and *Centella asiatica*(SWC) and the MPTP disease induced and no treatment group(SM). As both the sets were non-normal but the variances were unequal, we used a Welch's T Test with ranked data. The p-value obtained for the test was 0.0000 and hence the difference between the data sets was considered statistically significant.

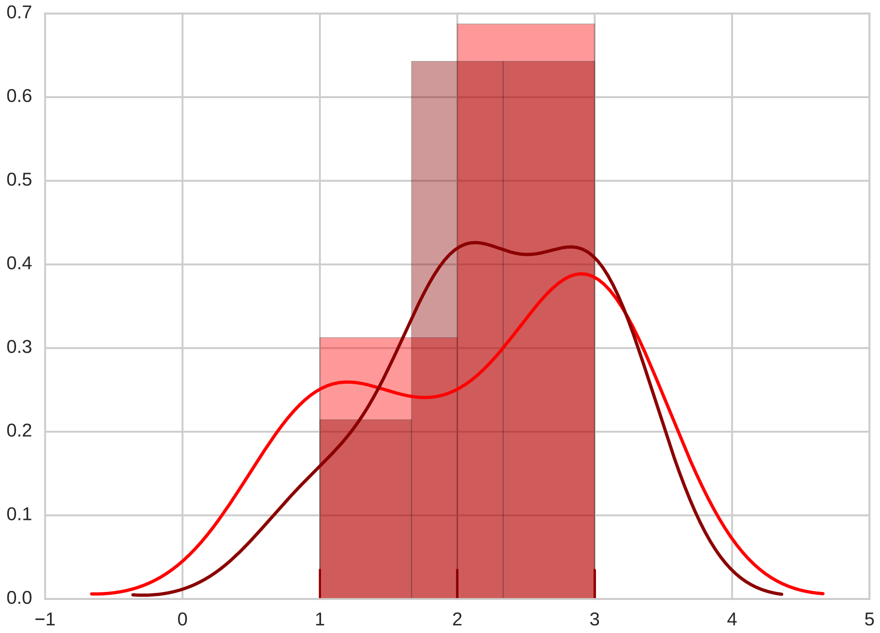

Figure 216: Significance Test Results for Swim-test results between the group treated with both *Withania somnifera* and *Centella asiatica*(SWC) and the MPTP disease induced and treated with *Centella asiatica* group(SMC). As both the sets were non-normal, we used a Mann-Whitney U Test. The p-value obtained for the test was 0.8753 and hence the difference between the data sets was considered statistically non-significant.

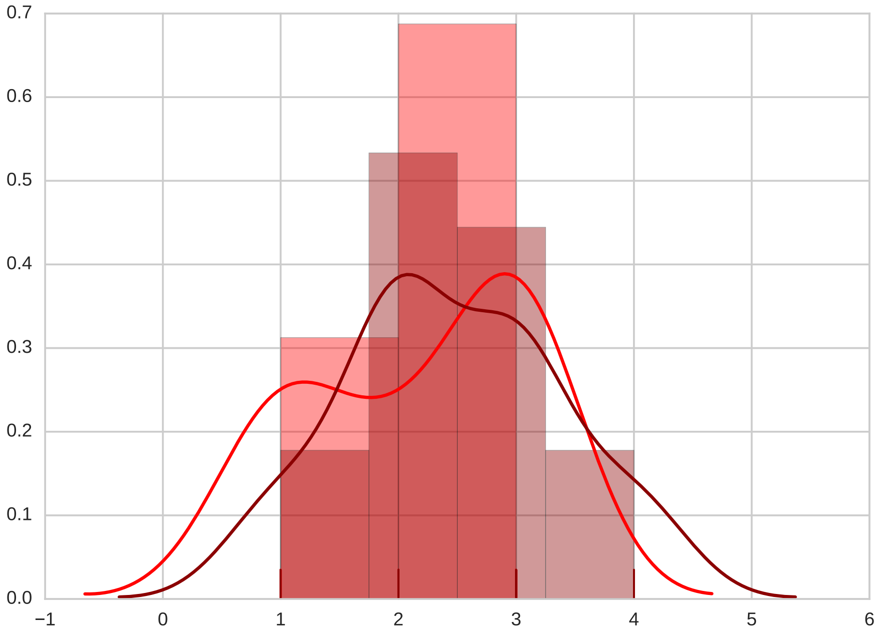

Figure 217: Significance Test Results for Swim-test results between the group treated with both *Withania somnifera* and *Centella asiatica*(SWC) and the MPTP disease induced and treated with *Withania somnifera* group(SMW). As both the sets were non-normal, we used a Mann-Whitney U Test. The p-value obtained for the test was 0.5163 and hence the difference between the data sets was considered statistically non-significant.

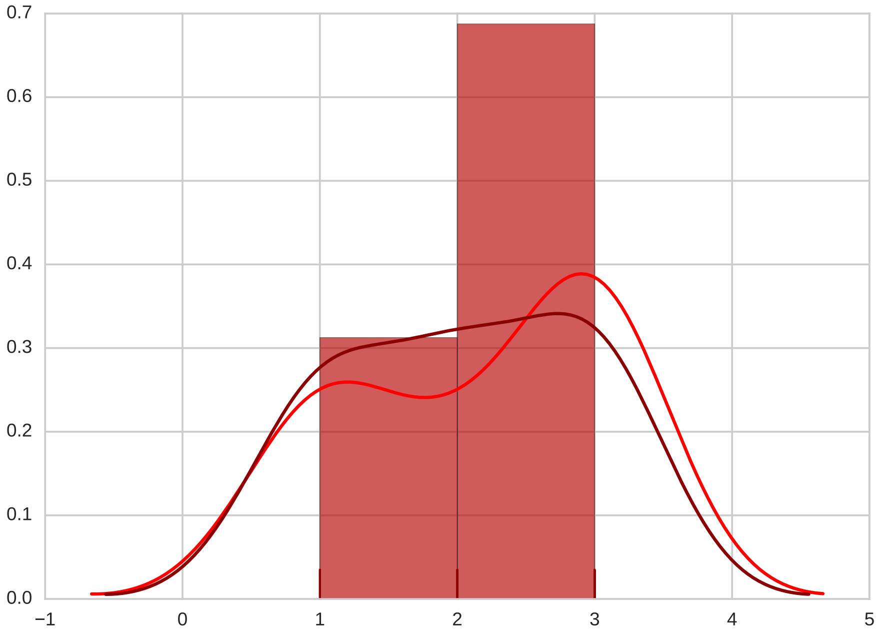

Figure 218: Significance Test Results for Swim-test results between the group treated with both *Withania somnifera* and *Centella asiatica*(SWC) and the MPTP disease induced and treated with both *Withania somnifera* and *Centella asiatica* group(SMWC). As both the sets were non-normal, we used a Mann-Whitney U Test. The p-value obtained for the test was 0.6715 and hence the difference between the data sets was considered statistically non-significant.

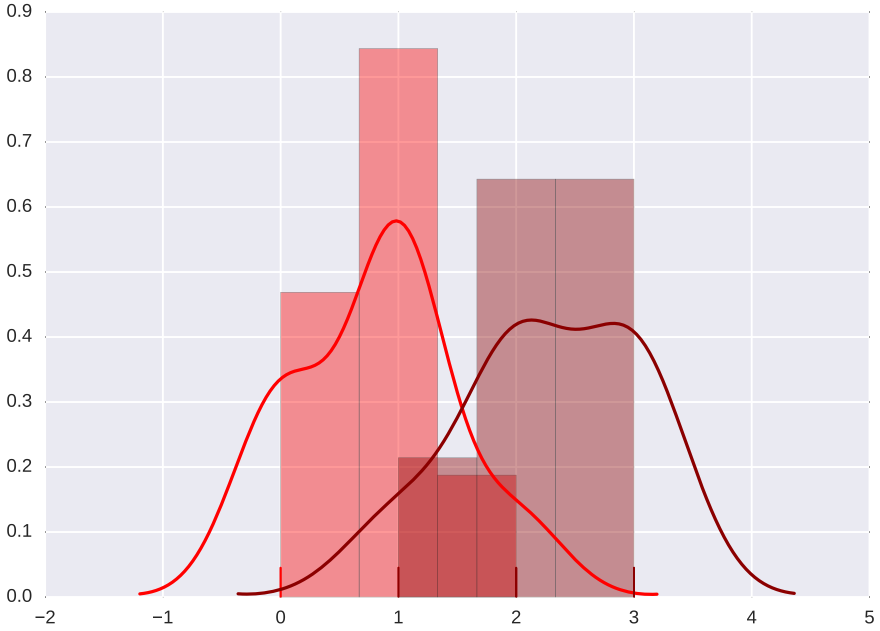

Figure 219: Significance Test Results for Swim-test results between the MPTP disease induced and no treatment group(SM) and the MPTP disease induced and treated with *Centella asiatica* group(SMC). As both the sets were non-normal, we used a Mann-Whitney U Test. The p-value obtained for the test was 0.0001 and hence the difference between the data sets was considered statistically significant.

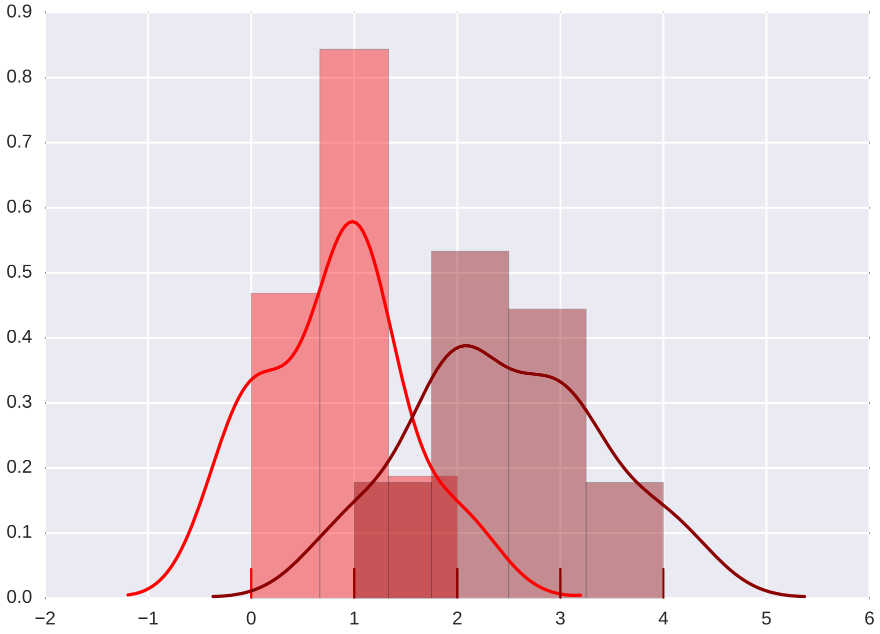

Figure 220: Significance Test Results for Swim-test results between the MPTP disease induced and no treatment group(SM) and the MPTP disease induced and treated with *Withania somnifera* group(SMW). As both the sets were non-normal, we used a Mann-Whitney U Test. The p-value obtained for the test was 0.0000 and hence the difference between the data sets was considered statistically significant.

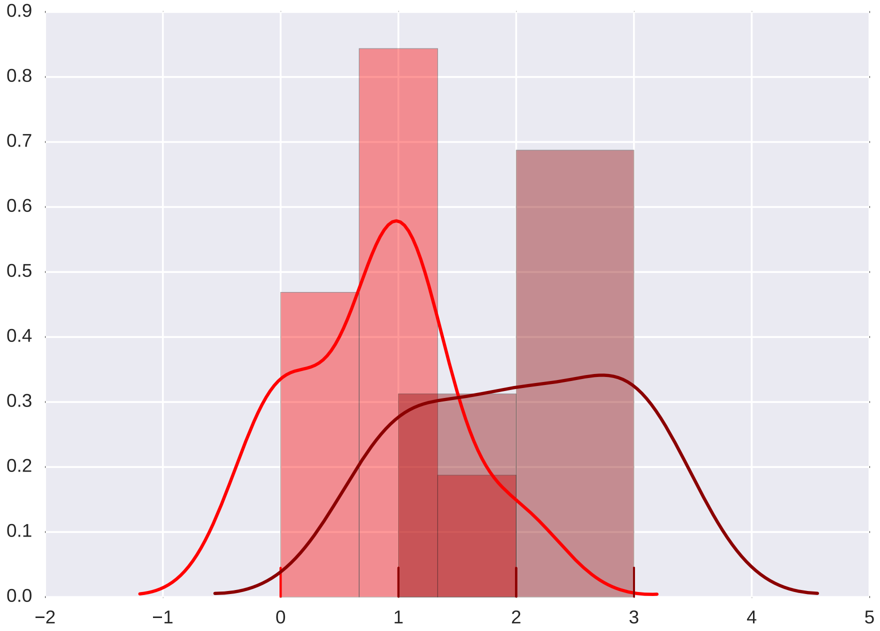

Figure 221: Significance Test Results for Swim-test results between the MPTP disease induced and no treatment group(SM) and the MPTP disease induced and treated with both *Withania somnifera* and *Centella asiatica* group(SMWC). As both the sets were non-normal, we used a Mann-Whitney U Test. The p-value obtained for the test was 0.0003 and hence the difference between the data sets was considered statistically significant.

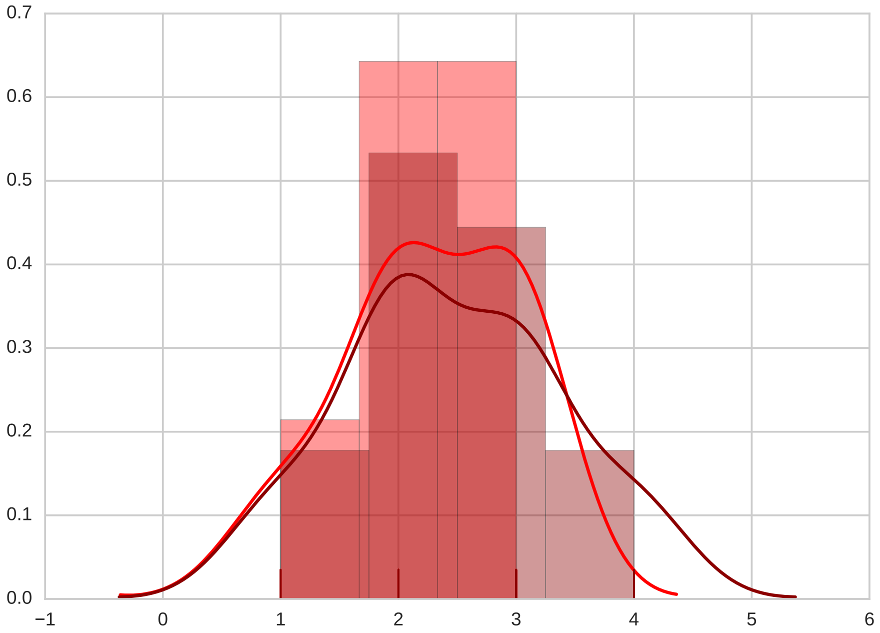

Figure 222: Significance Test Results for Swim-test results between the MPTP disease induced and treated with *Centella asiatica* group(SMC) and the MPTP disease induced and treated with *Withania somnifera* group(SMW). As both the sets were non-normal, we used a Mann-Whitney U Test. The p-value obtained for the test was 0.6572 and hence the difference between the data sets was considered statistically non-significant.

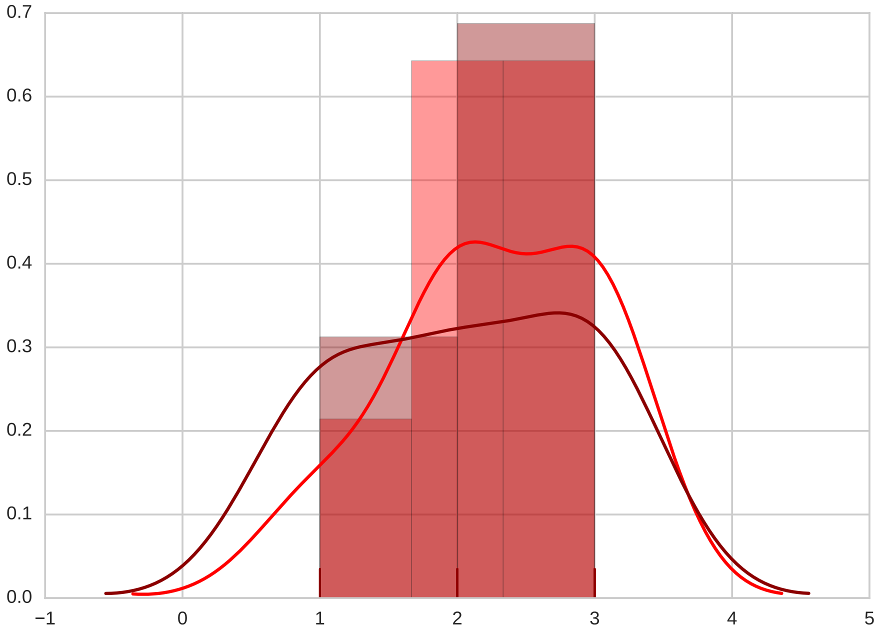

Figure 223: Significance Test Results for Swim-test results between the MPTP disease induced and treated with *Centella asiatica* group(SMC) and the MPTP disease induced and treated with both *Withania somnifera* and *Centella asiatica* group(SMWC). As both the sets were non-normal, we used a Mann-Whitney U Test. The p-value obtained for the test was 0.4909 and hence the difference between the data sets was considered statistically non-significant.

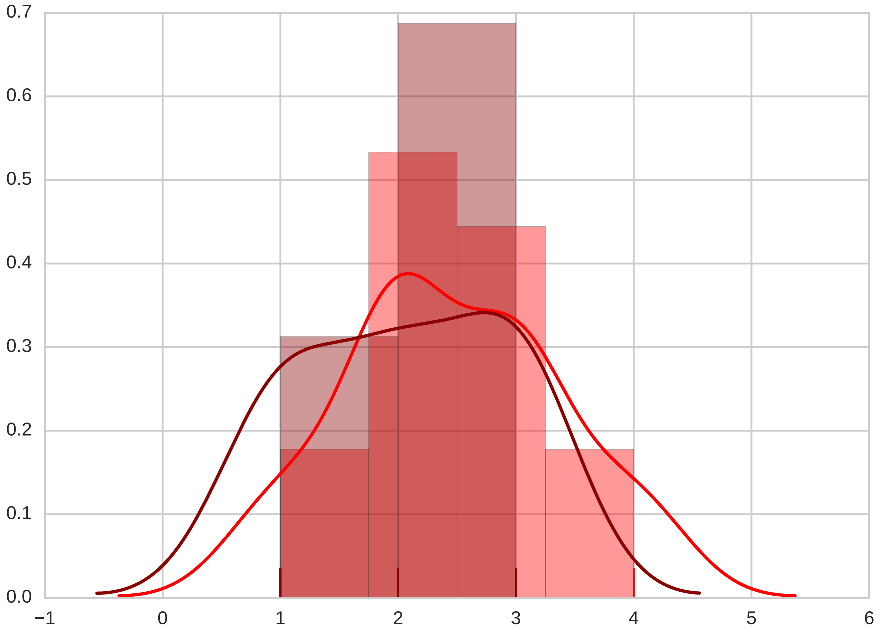

Figure 224: Significance Test Results for Swim-test results between the MPTP disease induced and treated with *Withania somnifera* group(SMW) and the MPTP disease induced and treated with both *Withania somnifera* and *Centella asiatica* group(SMWC). As both the sets were non-normal, we used a Mann-Whitney U Test. The p-value obtained for the test was 0.2695 and hence the difference between the data sets was considered statistically non-significant.

DataSet 1	DataSet 2	Size 1	Size 2	Levene's Test Statistic	Levene's Test P Value
SOD_U	SOD_C	17	12	1.4311	0.2420
SOD_U	SOD_W	17	15	0.5163	0.4780
SOD_U	SOD_WC	17	17	0.6963	0.4102
SOD_U	SOD_M	17	17	2.4386	0.1282
SOD_U	SOD_MC	17	14	0.0347	0.8536
SOD_U	SOD_MW	17	16	4.2921	0.0467
SOD_U	SOD_MWC	17	17	6.9655	0.0127
SOD_C	SOD_W	12	15	0.2055	0.6543
SOD_C	SOD_WC	12	17	0.1089	0.7440
SOD_C	SOD_M	12	17	5.1282	0.0318
SOD_C	SOD_MC	12	14	1.1651	0.2911
SOD_C	SOD_MW	12	16	6.4507	0.0174
SOD_C	SOD_MWC	12	17	8.6584	0.0066
SOD_W	SOD_WC	15	17	0.0162	0.8994
SOD_W	SOD_M	15	17	3.5655	0.0687
SOD_W	SOD_MC	15	14	0.5140	0.4796
SOD_W	SOD_MW	15	16	4.9960	0.0333
SOD_W	SOD_MWC	15	17	7.0477	0.0126
SOD_WC	SOD_M	17	17	3.6951	0.0635
SOD_WC	SOD_MC	17	14	0.6753	0.4179
SOD_WC	SOD_MW	17	16	5.0550	0.0318
SOD_WC	SOD_MWC	17	17	6.9336	0.0129
SOD_M	SOD_MC	17	14	0.8356	0.3682
SOD_M	SOD_MW	17	16	0.4932	0.4878
SOD_M	SOD_MWC	17	17	1.5329	0.2247
SOD_MC	SOD_MW	14	16	1.7102	0.2016
SOD_MC	SOD_MWC	14	17	2.8001	0.1050
SOD_MW	SOD_MWC	16	17	0.1853	0.6698
CAT_U	CAT_C	17	12	8.9814	0.0058
CAT_U	CAT_W	17	15	2.3028	0.1396
CAT_U	CAT_WC	17	17	1.5035	0.2291
CAT_U	CAT_M	17	17	0.3450	0.5611
CAT_U	CAT_MC	17	14	0.1655	0.6871
CAT_U	CAT_MW	17	16	1.8218	0.1869
CAT_U	CAT_MWC	17	17	2.0075	0.1662
CAT_C	CAT_W	12	15	1.4065	0.2468
CAT_C	CAT_WC	12	17	1.6605	0.2085
CAT_C	CAT_M	12	17	6.0992	0.0201
CAT_C	CAT_MC	12	14	7.4626	0.0116
CAT_C	CAT_MW	12	16	4.7874	0.0379
CAT_C	CAT_MWC	12	17	4.8984	0.0355
CAT_W	CAT_WC	15	17	0.0275	0.8694
CAT_W	CAT_M	15	17	1.0592	0.3116
CAT_W	CAT_MC	15	14	1.4560	0.2380
CAT_W	CAT_MW	15	16	0.3520	0.5576
CAT_W	CAT_MWC	15	17	0.3371	0.5659
CAT_WC	CAT_M	17	17	0.6026	0.4433
CAT_WC	CAT_MC	17	14	0.8405	0.3668
CAT_WC	CAT_MW	17	16	0.1184	0.7331
CAT_WC	CAT_MWC	17	17	0.1073	0.7453

CAT_M	CAT_MC	17	14	0.0472	0.8295
CAT_M	CAT_MW	17	16	0.4356	0.5141
CAT_M	CAT_MWC	17	17	0.5058	0.4821
CAT_MC	CAT_MW	14	16	1.0188	0.3215
CAT_MC	CAT_MWC	14	17	1.1406	0.2943
CAT_MW	CAT_MWC	16	17	0.0018	0.9663
GPx_U	GPx_C	17	12	0.2854	0.5976
GPx_U	GPx_W	17	15	1.0665	0.3100
GPx_U	GPx_WC	17	17	0.3696	0.5475
GPx_U	GPx_M	17	17	7.0582	0.0122
GPx_U	GPx_MC	17	17	5.2205	0.0291
GPx_U	GPx_MW	17	16	0.0033	0.9544
GPx_U	GPx_MWC	17	17	0.1906	0.6653
GPx_C	GPx_W	12	15	0.1364	0.7150
GPx_C	GPx_WC	12	17	1.5013	0.2311
GPx_C	GPx_M	12	17	3.4980	0.0723
GPx_C	GPx_MC	12	17	7.2845	0.0118
GPx_C	GPx_MW	12	16	0.2054	0.6542
GPx_C	GPx_MWC	12	17	0.0073	0.9325
GPx_W	GPx_WC	15	17	3.9416	0.0563
GPx_W	GPx_M	15	17	3.2893	0.0797
GPx_W	GPx_MC	15	17	13.5495	0.0009
GPx_W	GPx_MW	15	16	0.8348	0.3684
GPx_W	GPx_MWC	15	17	0.2071	0.6523
GPx_WC	GPx_M	17	17	12.4104	0.0013
GPx_WC	GPx_MC	17	17	5.0568	0.0315
GPx_WC	GPx_MW	17	16	0.4080	0.5277
GPx_WC	GPx_MWC	17	17	1.0399	0.3155
GPx_M	GPx_MC	17	17	23.0387	0.0000
GPx_M	GPx_MW	17	16	6.1595	0.0187
GPx_M	GPx_MWC	17	17	4.1854	0.0491
GPx_MC	GPx_MW	17	16	4.9003	0.0343
GPx_MC	GPx_MWC	17	17	5.9975	0.0200
GPx_MW	GPx_MWC	16	17	0.1325	0.7183
GSH_U	GSH_C	17	12	0.5678	0.4577
GSH_U	GSH_W	17	15	0.0372	0.8483
GSH_U	GSH_WC	17	17	0.3735	0.5454
GSH_U	GSH_M	17	17	1.6231	0.2118
GSH_U	GSH_MC	17	14	0.4224	0.5209
GSH_U	GSH_MW	17	16	0.3296	0.5700
GSH_U	GSH_MWC	17	17	1.4796	0.2327
GSH_C	GSH_W	12	15	0.2486	0.6224
GSH_C	GSH_WC	12	17	1.6315	0.2124
GSH_C	GSH_M	12	17	3.5965	0.0686
GSH_C	GSH_MC	12	14	1.5157	0.2302
GSH_C	GSH_MW	12	16	1.3759	0.2514
GSH_C	GSH_MWC	12	17	3.0752	0.0908
GSH_W	GSH_WC	15	17	0.5316	0.4716
GSH_W	GSH_M	15	17	1.7085	0.2011
GSH_W	GSH_MC	15	14	0.5526	0.4637
GSH_W	GSH_MW	15	16	0.4677	0.4995
GSH_W	GSH_MWC	15	17	1.5752	0.2191
GSH_WC	GSH_M	17	17	0.3879	0.5378

GSH_WC	GSH_MC	17	14	0.0104	0.9194
GSH_WC	GSH_MW	17	16	0.0005	0.9821
GSH_WC	GSH_MWC	17	17	0.4101	0.5265
GSH_M	GSH_MC	17	14	0.2005	0.6577
GSH_M	GSH_MW	17	16	0.2788	0.6012
GSH_M	GSH_MWC	17	17	0.0108	0.9180
GSH_MC	GSH_MW	14	16	0.0051	0.9434
GSH_MC	GSH_MWC	14	17	0.2287	0.6361
GSH_MW	GSH_MWC	16	17	0.3106	0.5813
LPO_U	LPO_C	17	12	0.0369	0.8491
LPO_U	LPO_W	17	15	0.1079	0.7448
LPO_U	LPO_WC	17	17	0.0026	0.9597
LPO_U	LPO_M	17	17	0.0609	0.8067
LPO_U	LPO_MC	17	14	0.7104	0.4062
LPO_U	LPO_MW	17	16	0.4995	0.4850
LPO_U	LPO_MWC	17	17	0.7883	0.3812
LPO_C	LPO_W	12	15	0.0118	0.9144
LPO_C	LPO_WC	12	17	0.0487	0.8270
LPO_C	LPO_M	12	17	0.1473	0.7042
LPO_C	LPO_MC	12	14	0.2679	0.6095
LPO_C	LPO_MW	12	16	0.1565	0.6956
LPO_C	LPO_MWC	12	17	0.2877	0.5961
LPO_W	LPO_WC	15	17	0.1239	0.7273
LPO_W	LPO_M	15	17	0.2736	0.6048
LPO_W	LPO_MC	15	14	0.1565	0.6955
LPO_W	LPO_MW	15	16	0.0729	0.7890
LPO_W	LPO_MWC	15	17	0.1612	0.6909
LPO_WC	LPO_M	17	17	0.0340	0.8549
LPO_WC	LPO_MC	17	14	0.6619	0.4225
LPO_WC	LPO_MW	17	16	0.4864	0.4907
LPO_WC	LPO_MWC	17	17	0.7400	0.3961
LPO_M	LPO_MC	17	14	0.9959	0.3266
LPO_M	LPO_MW	17	16	0.7958	0.3792
LPO_M	LPO_MWC	17	17	1.1322	0.2953
LPO_MC	LPO_MW	14	16	0.0299	0.8640
LPO_MC	LPO_MWC	14	17	0.0019	0.9654
LPO_MW	LPO_MWC	16	17	0.0213	0.8850
AU	AC	16	12	0.0357	0.8516
AU	AW	16	15	0.2521	0.6194
AU	AWC	16	16	0.4839	0.4920
AU	AM	16	16	12.9726	0.0011
AU	AMC	16	14	40.6960	0.0000
AU	AMW	16	15	11.0244	0.0024
AU	AMWC	16	16	8.1985	0.0076
AC	AW	12	15	0.0482	0.8281
AC	AWC	12	16	0.1389	0.7124
AC	AM	12	16	9.5691	0.0047
AC	AMC	12	14	29.1408	0.0000
AC	AMW	12	15	8.0829	0.0088
AC	AMWC	12	16	6.0033	0.0213
AW	AWC	15	16	0.0323	0.8587
AW	AM	15	16	11.9593	0.0017
AW	AMC	15	14	36.3339	0.0000

AW	AMW	15	15	10.0565	0.0037
AW	AMWC	15	16	7.4464	0.0107
AWC	AM	16	16	12.7146	0.0012
AWC	AMC	16	14	38.0643	0.0000
AWC	AMW	16	15	10.6506	0.0028
AWC	AMWC	16	16	7.8721	0.0087
AM	AMC	16	14	5.1416	0.0313
AM	AMW	16	15	1.5145	0.2283
AM	AMWC	16	16	2.3580	0.1351
AMC	AMW	14	15	1.8941	0.1801
AMC	AMWC	14	16	0.8832	0.3554
AMW	AMWC	15	16	0.1232	0.7281
CU	CC	17	12	0.4425	0.5116
CU	CW	17	15	0.0213	0.8850
CU	CWC	17	17	0.0000	1.0000
CU	CM	17	17	11.7143	0.0017
CU	CMC	17	14	13.3597	0.0010
CU	CMW	17	16	12.1008	0.0015
CU	CMWC	17	17	16.5161	0.0003
CC	CW	12	15	0.4777	0.4958
CC	CWC	12	17	0.4425	0.5116
CC	CM	12	17	7.4032	0.0113
CC	CMC	12	14	7.4250	0.0118
CC	CMW	12	16	6.2732	0.0189
CC	CMWC	12	17	9.1075	0.0055
CW	CWC	15	17	0.0213	0.8850
CW	CM	15	17	10.4231	0.0030
CW	CMC	15	14	11.4126	0.0022
CW	CMW	15	16	10.2515	0.0033
CW	CMWC	15	17	14.0255	0.0008
CWC	CM	17	17	11.7143	0.0017
CWC	CMC	17	14	13.3597	0.0010
CWC	CMW	17	16	12.1008	0.0015
CWC	CMWC	17	17	16.5161	0.0003
CM	CMC	17	14	3.1100	0.0883
CM	CMW	17	16	4.9127	0.0341
CM	CMWC	17	17	4.1254	0.0506
CMC	CMW	14	16	0.4779	0.4950
CMC	CMWC	14	17	0.0237	0.8788
CMW	CMWC	16	17	0.3815	0.5413
SU	SC	16	12	4.3813	0.0462
SU	SW	16	15	6.2412	0.0184
SU	SWC	16	16	8.7273	0.0060
SU	SM	16	16	0.5085	0.4813
SU	SMC	16	14	2.0414	0.1641
SU	SMW	16	15	3.8340	0.0599
SU	SMWC	16	16	4.9091	0.0344
SC	SW	12	15	0.1610	0.6916
SC	SWC	12	16	0.7429	0.3966
SC	SM	12	16	1.6707	0.2075
SC	SMC	12	14	0.2761	0.6041
SC	SMW	12	15	0.0861	0.7716
SC	SMWC	12	16	0.0152	0.9030

SW	SWC	15	16	0.2209	0.6419
SW	SM	15	16	2.8598	0.1015
SW	SMC	15	14	0.8056	0.3774
SW	SMW	15	15	0.0000	1.0000
SW	SMWC	15	16	0.0740	0.7875
SWC	SM	16	16	4.5763	0.0407
SWC	SMC	16	14	1.7696	0.1942
SWC	SMW	16	15	0.1357	0.7153
SWC	SMWC	16	16	0.5455	0.4659
SM	SMC	16	14	0.5091	0.4814
SM	SMW	16	15	1.8075	0.1892
SM	SMWC	16	16	2.0339	0.1641
SMC	SMW	14	15	0.4946	0.4879
SMC	SMWC	14	16	0.4102	0.5271
SMW	SMWC	15	16	0.0455	0.8326

*Red cells show the combinations with unequal variances

DataSet 1	DataSet 2	Size 1	Size 2	Test Statistic	P Value
SOD_U	SOD_C	17	12	-3.9168	0.0006
SOD_U	SOD_W	17	15	-5.3786	0.0000
SOD_U	SOD_WC	17	17	-5.8040	0.0000
SOD_U	SOD_M	17	17	14.4183	0.0000
SOD_U	SOD_MC	17	14	38.0000	0.0014
SOD_U	SOD_MW	17	16	*-3.7995*	*0.0007*
SOD_U	SOD_MWC	17	17	*2.4436*	*0.0220*
SOD_C	SOD_W	12	15	-0.7084	0.4852
SOD_C	SOD_WC	12	17	-0.9771	0.3372
SOD_C	SOD_M	12	17	*12.8688*	*0.0000*
SOD_C	SOD_MC	12	14	95.0000	0.5890
SOD_C	SOD_MW	12	16	*1.5815*	*0.1365*
SOD_C	SOD_MWC	12	17	*5.4383*	*0.0001*
SOD_W	SOD_WC	15	17	-0.2813	0.7804
SOD_W	SOD_M	15	17	17.4966	0.0000
SOD_W	SOD_MC	15	14	142.0000	0.1110
SOD_W	SOD_MW	15	16	*2.9881*	*0.0074*
SOD_W	SOD_MWC	15	17	*7.8382*	*0.0000*
SOD_WC	SOD_M	17	17	17.9782	0.0000
SOD_WC	SOD_MC	17	14	169.0000	0.0494
SOD_WC	SOD_MW	17	16	*3.4957*	*0.0020*
SOD_WC	SOD_MWC	17	17	*8.5982*	*0.0000*
SOD_M	SOD_MC	17	14	0.0000	0.0000
SOD_M	SOD_MW	17	16	-23.1910	0.0000
SOD_M	SOD_MWC	17	17	-16.6415	0.0000
SOD_MC	SOD_MW	14	16	117.5000	0.8353
SOD_MC	SOD_MWC	14	17	227.0000	0.0000
SOD_MW	SOD_MWC	16	17	8.6505	0.0000
CAT_U	CAT_C	17	12	*-4.4087*	*0.0005*
CAT_U	CAT_W	17	15	-5.4258	0.0000
CAT_U	CAT_WC	17	17	34.5000	0.0002
CAT_U	CAT_M	17	17	289.0000	0.0000
CAT_U	CAT_MC	17	14	2.0652	0.0479
CAT_U	CAT_MW	17	16	6.0279	0.0000
CAT_U	CAT_MWC	17	17	7.0935	0.0000
CAT_C	CAT_W	12	15	0.4032	0.6902
CAT_C	CAT_WC	12	17	118.0000	0.4924
CAT_C	CAT_M	12	17	*9.0215*	*0.0000*
CAT_C	CAT_MC	12	14	*5.5090*	*0.0001*
CAT_C	CAT_MW	12	16	*7.7191*	*0.0000*
CAT_C	CAT_MWC	12	17	*8.3240*	*0.0000*
CAT_W	CAT_WC	15	17	128.0000	1.0000
CAT_W	CAT_M	15	17	255.0000	0.0000
CAT_W	CAT_MC	15	14	6.5882	0.0000
CAT_W	CAT_MW	15	16	9.5857	0.0000
CAT_W	CAT_MWC	15	17	10.5186	0.0000
CAT_WC	CAT_M	17	17	289.0000	0.0000
CAT_WC	CAT_MC	17	14	221.0000	0.0001
CAT_WC	CAT_MW	17	16	266.0000	0.0000
CAT_WC	CAT_MWC	17	17	284.0000	0.0000
CAT_M	CAT_MC	17	14	0.0000	0.0000
CAT_M	CAT_MW	17	16	0.0000	0.0000

CAT_M	CAT_MWC	17	17	0.0000	0.0000
CAT_MC	CAT_MW	14	16	3.9311	0.0005
CAT_MC	CAT_MWC	14	17	4.9230	0.0000
CAT_MW	CAT_MWC	16	17	0.9162	0.3667
GPx_U	GPx_C	17	12	35.0000	0.0032
GPx_U	GPx_W	17	15	145.0000	0.5208
GPx_U	GPx_WC	17	17	157.0000	0.6792
GPx_U	GPx_M	17	17	*9.8240*	*0.0000*
GPx_U	GPx_MC	17	17	*-2.0768*	*0.0474*
GPx_U	GPx_MW	17	16	127.5000	0.7730
GPx_U	GPx_MWC	17	17	172.5000	0.3429
GPx_C	GPx_W	12	15	4.0890	0.0004
GPx_C	GPx_WC	12	17	4.8276	0.0000
GPx_C	GPx_M	12	17	10.4706	0.0000
GPx_C	GPx_MC	12	17	*1.8595*	*0.0827*
GPx_C	GPx_MW	12	16	3.3909	0.0022
GPx_C	GPx_MWC	12	17	4.3773	0.0002
GPx_W	GPx_WC	15	17	-0.4536	0.6534
GPx_W	GPx_M	15	17	7.8121	0.0000
GPx_W	GPx_MC	15	17	*-2.4793*	*0.0210*
GPx_W	GPx_MW	15	16	-1.0497	0.3025
GPx_W	GPx_MWC	15	17	0.1875	0.8525
GPx_WC	GPx_M	17	17	*9.4155*	*0.0000*
GPx_WC	GPx_MC	17	17	*-4.1842*	*0.0002*
GPx_WC	GPx_MW	17	16	-0.8377	0.4086
GPx_WC	GPx_MWC	17	17	0.6938	0.4928
GPx_M	GPx_MC	17	17	*-10.5594*	*0.0000*
GPx_M	GPx_MW	17	16	*-9.2068*	*0.0000*
GPx_M	GPx_MWC	17	17	*-8.0160*	*0.0000*
GPx_MC	GPx_MW	17	16	*3.0333*	*0.0050*
GPx_MC	GPx_MWC	17	17	*4.3253*	*0.0001*
GPx_MW	GPx_MWC	16	17	1.2804	0.2099
GSH_U	GSH_C	17	12	4.9575	0.0000
GSH_U	GSH_W	17	15	6.7295	0.0000
GSH_U	GSH_WC	17	17	7.2706	0.0000
GSH_U	GSH_M	17	17	26.9977	0.0000
GSH_U	GSH_MC	17	14	12.6641	0.0000
GSH_U	GSH_MW	17	16	10.4970	0.0000
GSH_U	GSH_MWC	17	17	16.6036	0.0000
GSH_C	GSH_W	12	15	1.0110	0.3217
GSH_C	GSH_WC	12	17	0.8060	0.4273
GSH_C	GSH_M	12	17	16.7240	0.0000
GSH_C	GSH_MC	12	14	5.5952	0.0000
GSH_C	GSH_MW	12	16	3.6914	0.0010
GSH_C	GSH_MWC	12	17	8.3246	0.0000
GSH_W	GSH_WC	15	17	-0.3875	0.7012
GSH_W	GSH_M	15	17	17.1627	0.0000
GSH_W	GSH_MC	15	14	5.0143	0.0000
GSH_W	GSH_MW	15	16	2.8699	0.0076
GSH_W	GSH_MWC	15	17	7.9040	0.0000
GSH_WC	GSH_M	17	17	20.4932	0.0000
GSH_WC	GSH_MC	17	14	6.2291	0.0000
GSH_WC	GSH_MW	17	16	3.7276	0.0008

GSH_WC	GSH_MWC	17	17	9.6261	0.0000
GSH_M	GSH_MC	17	14	-12.5852	0.0000
GSH_M	GSH_MW	17	16	-15.5480	0.0000
GSH_M	GSH_MWC	17	17	-11.0291	0.0000
GSH_MC	GSH_MW	14	16	-2.4592	0.0204
GSH_MC	GSH_MWC	14	17	2.5535	0.0162
GSH_MW	GSH_MWC	16	17	5.3219	0.0000
LPO_U	LPO_C	17	12	-0.7276	0.4731
LPO_U	LPO_W	17	15	-0.2438	0.8090
LPO_U	LPO_WC	17	17	0.3727	0.7119
LPO_U	LPO_M	17	17	-5.3785	0.0000
LPO_U	LPO_MC	17	14	-4.6103	0.0001
LPO_U	LPO_MW	17	16	-4.9770	0.0000
LPO_U	LPO_MWC	17	17	-4.6586	0.0001
LPO_C	LPO_W	12	15	0.4909	0.6278
LPO_C	LPO_WC	12	17	1.0323	0.3111
LPO_C	LPO_M	12	17	-4.0883	0.0004
LPO_C	LPO_MC	12	14	-3.4402	0.0021
LPO_C	LPO_MW	12	16	-3.7269	0.0009
LPO_C	LPO_MWC	12	17	-3.4132	0.0020
LPO_W	LPO_WC	15	17	0.5964	0.5554
LPO_W	LPO_M	15	17	-4.9730	0.0000
LPO_W	LPO_MC	15	14	-4.2716	0.0002
LPO_W	LPO_MW	15	16	-4.6091	0.0001
LPO_W	LPO_MWC	15	17	-4.2898	0.0002
LPO_WC	LPO_M	17	17	-5.5747	0.0000
LPO_WC	LPO_MC	17	14	-4.7988	0.0000
LPO_WC	LPO_MW	17	16	-5.1777	0.0000
LPO_WC	LPO_MWC	17	17	-4.8846	0.0000
LPO_M	LPO_MC	17	14	1.2091	0.2364
LPO_M	LPO_MW	17	16	1.0685	0.2935
LPO_M	LPO_MWC	17	17	1.5613	0.1283
LPO_MC	LPO_MW	14	16	-0.2187	0.8285
LPO_MC	LPO_MWC	14	17	0.3015	0.7652
LPO_MW	LPO_MWC	16	17	0.5409	0.5924
AU	AC	16	12	119.0000	0.2357
AU	AW	16	15	139.0000	0.3965
AU	AWC	16	16	118.0000	0.6832
AU	AM	16	16	*-10.3145*	*0.0000*
AU	AMC	16	14	*-10.0245*	*0.0000*
AU	AMW	16	15	*-10.1679*	*0.0000*
AU	AMWC	16	16	*-10.3369*	*0.0000*
AC	AW	12	15	81.5000	0.6504
AC	AWC	12	16	69.0000	0.1728
AC	AM	12	16	*-9.5341*	*0.0000*
AC	AMC	12	14	*-8.9831*	*0.0000*
AC	AMW	12	15	*-9.1522*	*0.0000*
AC	AMWC	12	16	*-9.3442*	*0.0000*
AW	AWC	15	16	94.0000	0.2583
AW	AM	15	16	*-9.9922*	*0.0000*
AW	AMC	15	14	*-9.6904*	*0.0000*
AW	AMW	15	15	*-9.8399*	*0.0000*
AW	AMWC	15	16	*-10.0139*	*0.0000*

AWC	AM	16	16	-9.9749	0.0000
AWC	AMC	16	14	-9.3235	0.0000
AWC	AMW	16	15	-9.4930	0.0000
AWC	AMWC	16	16	-9.6808	0.0000
AM	AMC	16	14	1.5443	0.1338
AM	AMW	16	15	128.0000	0.7666
AM	AMWC	16	16	142.5000	0.5969
AMC	AMW	14	15	65.5000	0.0877
AMC	AMWC	14	16	83.5000	0.2414
AMW	AMWC	15	16	137.0000	0.5132
CU	CC	17	12	89.5000	0.5189
CU	CW	17	15	137.0000	0.6521
CU	CWC	17	17	144.5000	0.9826
CU	CM	17	17	-9.6175	0.0000
CU	CMC	17	14	-2.6261	0.0156
CU	CMW	17	16	-4.6375	0.0001
CU	CMWC	17	17	-2.9763	0.0061
CC	CW	12	15	107.0000	0.3123
CC	CWC	12	17	114.5000	0.5189
CC	CM	12	17	-8.2061	0.0000
CC	CMC	12	14	-2.0436	0.0528
CC	CMW	12	16	-3.9378	0.0006
CC	CMWC	12	17	-2.3600	0.0258
CW	CWC	15	17	118.0000	0.6521
CW	CM	15	17	-9.1949	0.0000
CW	CMC	15	14	-2.9198	0.0080
CW	CMW	15	16	-4.7351	0.0001
CW	CMWC	15	17	-3.2563	0.0031
CWC	CM	17	17	-9.6175	0.0000
CWC	CMC	17	14	-2.6261	0.0156
CWC	CMW	17	16	-4.6375	0.0001
CWC	CMWC	17	17	-2.9763	0.0061
CM	CMC	17	14	204.5000	0.0007
CM	CMW	17	16	3.7984	0.0007
CM	CMWC	17	17	248.0000	0.0003
CMC	CMW	14	16	95.0000	0.4826
CMC	CMWC	14	17	121.0000	0.9508
CMW	CMWC	16	17	158.5000	0.4156
SU	SC	16	12	1.2067	0.2415
SU	SW	16	15	2.7178	0.0119
SU	SWC	16	16	1.6050	0.1203
SU	SM	16	16	251.0000	0.0000
SU	SMC	16	14	146.0000	0.1123
SU	SMW	16	15	140.5000	0.3821
SU	SMWC	16	16	2.3429	0.0268
SC	SW	12	15	113.0000	0.2423
SC	SWC	12	16	103.0000	0.7427
SC	SM	12	16	175.0000	0.0001
SC	SMC	12	14	88.0000	0.8445
SC	SMW	12	15	85.0000	0.8148
SC	SMWC	12	16	113.0000	0.4125
SW	SWC	15	16	101.0000	0.4333
SW	SM	15	16	197.0000	0.0012

SW	SMC	15	14	81.0000	0.2755
SW	SMW	15	15	78.5000	0.1447
SW	SMWC	15	16	110.0000	0.6901
SWC	SM	16	16	*4.8413*	*0.0000*
SWC	SMC	16	14	108.0000	0.8753
SWC	SMW	16	15	104.0000	0.5163
SWC	SMWC	16	16	139.0000	0.6715
SM	SMC	16	14	19.0000	0.0001
SM	SMW	16	15	19.0000	0.0000
SM	SMWC	16	16	37.5000	0.0003
SMC	SMW	14	15	95.0000	0.6572
SMC	SMWC	14	16	128.0000	0.4909
SMW	SMWC	15	16	147.0000	0.2695

*Red cells show that the difference is significant among the groups.
*Underlined values show that atleast one of the groups was non-normal
*Italicized values show that the variance of the groups was unequal
