# Supplementary material for: Complete Comparison Display (CCD) evaluation of ethanol extracts of Centella asiatica and Withania somnifera shows that they can non-synergistically ameliorate biochemical and behavioural damages in MPTP induced Parkinson's model of mice
Source: PLoS One. 2017 May 16;12(5):e0177254. doi: 10.1371/journal.pone.0177254 (PMC5433711; doi:10.1371/journal.pone.0177254)
Supplement: S1 Table — This pdf has tables that present value of each single biochemical and behavioural measurement. (PDF) [file pone.0177254.s003.pdf]

Group	Score
AU	1
AU	2
AU	1
AU	2
AU	1
AU	2
AU	2
AU	2
AU	2
AU	1
AU	2
AU	1
AU	2
AU	2
AU	2
AU	1
AC	1
AC	1
AC	1
AC	1
AC	1
AC	1
AC	1
AC	2
AC	2
AC	2
AC	3
AC	1
AW	1
AW	1
AW	1
AW	1
AW	1
AW	1
AW	2
AW	2
AW	1
AW	2
AW	2
AW	2
AW	2
AW	1
AW	2
AWC	2
AWC	1
AWC	1
AWC	1
AWC	2
AWC	1

AWC	2
AWC	2
AWC	1
AWC	2
AWC	2
AWC	2
AWC	3
AWC	1
AWC	2
AWC	3
AM	5
AM	6
AM	8
AM	6
AM	13
AM	15
AM	14
AM	47
AM	40
AM	30
AM	22
AM	10
AM	56
AM	6
AM	50
AM	13
AMC	11
AMC	12
AMC	18
AMC	8
AMC	16
AMC	3
AMC	5
AMC	4
AMC	4
AMC	10
AMC	15
AMC	15
AMC	16
AMC	8
AMW	5
AMW	14
AMW	3
AMW	4
AMW	11
AMW	12
AMW	18
AMW	8
AMW	16
AMW	52

AMW	18
AMW	26
AMW	19
AMW	22
AMW	18
AMWC	3
AMWC	5
AMWC	4
AMWC	4
AMWC	10
AMWC	15
AMWC	15
AMWC	16
AMWC	8
AMWC	16
AMWC	50
AMWC	15
AMWC	20
AMWC	17
AMWC	20
AMWC	16

Group	Score
CU	1
CU	2
CU	2
CU	1
CU	1
CU	2
CU	1
CU	1
CU	1
CU	2
CU	1
CU	1
CU	1
CU	1
CU	1
CU	2
CU	1
CC	1
CC	1
CC	1
CC	1
CC	2
CC	2
CC	1
CC	2
CC	2
CC	2
CC	1
CC	1
CW	1
CW	1
CW	1
CW	1
CW	2
CW	2
CW	1
CW	1
CW	1
CW	1
CW	1
CW	1
CW	1
CW	3
CW	1
CWC	1
CWC	1
CWC	1
CWC	1
CWC	1

CWC	2
CWC	1
CWC	1
CWC	1
CWC	2
CWC	2
CWC	2
CWC	1
CWC	1
CWC	1
CWC	2
CWC	1
CM	3
CM	3
CM	3
CM	2
CM	5
CM	11
CM	10
CM	7
CM	18
CM	7
CM	9
CM	7
CM	7
CM	2
CM	5
CM	7
CM	7
CMC	3
CMC	5
CMC	4
CMC	2
CMC	1
CMC	4
CMC	2
CMC	1
CMC	1
CMC	5
CMC	4
CMC	2
CMC	1
CMC	1
CMW	4
CMW	2
CMW	1
CMW	4
CMW	3
CMW	5
CMW	4

CMW	2
CMW	1
CMW	1
CMW	4
CMW	3
CMW	3
CMW	3
CMW	5
CMW	2
CMWC	4
CMWC	2
CMWC	1
CMWC	1
CMWC	5
CMWC	4
CMWC	2
CMWC	1
CMWC	1
CMWC	1
CMWC	1
CMWC	4
CMWC	4
CMWC	3
CMWC	2
CMWC	4
CMWC	3

Group	Score
SU	3
SU	3
SU	2
SU	3
SU	3
SU	3
SU	3
SU	2
SU	3
SU	3
SU	2
SU	2
SU	3
SU	3
SU	3
SU	2
SC	3
SC	3
SC	2
SC	2
SC	2
SC	3
SC	3
SC	2
SC	1
SC	1
SC	3
SC	3
SW	3
SW	3
SW	2
SW	2
SW	2
SW	3
SW	3
SW	1
SW	1
SW	1
SW	1
SW	3
SW	2
SW	1
SW	1
SWC	3
SWC	3
SWC	2
SWC	2
SWC	3
SWC	3

SWC	3
SWC	1
SWC	3
SWC	2
SWC	1
SWC	1
SWC	3
SWC	3
SWC	1
SWC	1
SM	0
SM	1
SM	1
SM	0
SM	1
SM	0
SM	1
SM	1
SM	2
SM	0
SM	1
SM	0
SM	2
SM	1
SM	1
SM	1
SMC	3
SMC	2
SMC	2
SMC	3
SMC	2
SMC	3
SMC	2
SMC	2
SMC	2
SMC	3
SMC	1
SMC	3
SMC	3
SMC	1
SMW	4
SMW	3
SMW	3
SMW	2
SMW	3
SMW	2
SMW	2
SMW	3
SMW	2
SMW	2

SMW	3
SMW	1
SMW	4
SMW	2
SMW	1
SMWC	3
SMWC	2
SMWC	2
SMWC	2
SMWC	3
SMWC	1
SMWC	3
SMWC	3
SMWC	1
SMWC	2
SMWC	1
SMWC	3
SMWC	2
SMWC	3
SMWC	1
SMWC	1

Group	Score
SOD_U	18.2
SOD_U	17
SOD_U	18.9
SOD_U	17.3
SOD_U	20.2
SOD_U	18.56
SOD_U	17.49
SOD_U	17.9
SOD_U	18.67
SOD_U	16.99
SOD_U	17.32
SOD_U	18.99
SOD_U	19.56
SOD_U	19.2
SOD_U	18.89
SOD_U	18.1
SOD_U	19.88
SOD_C	23.03
SOD_C	18.47
SOD_C	20.45
SOD_C	20.58
SOD_C	21.02
SOD_C	22.02
SOD_C	18.01
SOD_C	18.26
SOD_C	19.36
SOD_C	20.01
SOD_C	20.58
SOD_C	21.25
SOD_W	23.01
SOD_W	18.45
SOD_W	20.43
SOD_W	20.56
SOD_W	21
SOD_W	22
SOD_W	21.89
SOD_W	18.9
SOD_W	18.36
SOD_W	21.09
SOD_W	20
SOD_W	20.56
SOD_W	20.34
SOD_W	21.07
SOD_W	22
SOD_WC	22.54
SOD_WC	20.45
SOD_WC	21.34
SOD_WC	21.56
SOD_WC	22

SOD_WC	21.99
SOD_WC	20.89
SOD_WC	19.68
SOD_WC	17.99
SOD_WC	18.24
SOD_WC	19.34
SOD_WC	19.99
SOD_WC	20.56
SOD_WC	21.23
SOD_WC	21.34
SOD_WC	22.09
SOD_WC	21.99
SOD_M	14.52
SOD_M	13.68
SOD_M	13.89
SOD_M	14.6
SOD_M	12.56
SOD_M	14.99
SOD_M	13.34
SOD_M	13.76
SOD_M	14.34
SOD_M	14.76
SOD_M	15.2
SOD_M	13.46
SOD_M	13.92
SOD_M	14.93
SOD_M	14.59
SOD_M	14.53
SOD_M	13.91
SOD_MC	21.04
SOD_MC	21.45
SOD_MC	21.64
SOD_MC	21.57
SOD_MC	19.59
SOD_MC	18.72
SOD_MC	19.63
SOD_MC	19.61
SOD_MC	19.84
SOD_MC	19.13
SOD_MC	19.5
SOD_MC	19.67
SOD_MC	19.61
SOD_MC	17.81
SOD_MW	18.89
SOD_MW	19.81
SOD_MW	19.79
SOD_MW	20.02
SOD_MW	19.31
SOD_MW	19.68
SOD_MW	19.86

SOD_MW	19.79
SOD_MW	17.97
SOD_MW	20.02
SOD_MW	19.29
SOD_MW	19.67
SOD_MW	20.17
SOD_MW	18.69
SOD_MW	18.95
SOD_MW	20.3
SOD_MWC	17.17
SOD_MWC	18.01
SOD_MWC	17.99
SOD_MWC	18.2
SOD_MWC	17.55
SOD_MWC	17.89
SOD_MWC	18.05
SOD_MWC	17.99
SOD_MWC	16.34
SOD_MWC	17.87
SOD_MWC	18.2
SOD_MWC	17.54
SOD_MWC	17.88
SOD_MWC	18.34
SOD_MWC	16.99
SOD_MWC	17.23
SOD_MWC	18.45

Group	Score
CAT_U	45.28
CAT_U	44.56
CAT_U	45.99
CAT_U	44
CAT_U	43.29
CAT_U	44.78
CAT_U	45.12
CAT_U	44.9
CAT_U	42.1
CAT_U	43.44
CAT_U	44.9
CAT_U	45.67
CAT_U	48.9
CAT_U	45.59
CAT_U	45.19
CAT_U	44.89
CAT_U	44.01
CAT_C	53.04
CAT_C	50.86
CAT_C	48.27
CAT_C	50.87
CAT_C	47.82
CAT_C	51.57
CAT_C	48.17
CAT_C	46.02
CAT_C	42.87
CAT_C	45.89
CAT_C	51.23
CAT_C	50.16
CAT_W	52
CAT_W	49.86
CAT_W	47.32
CAT_W	49.87
CAT_W	46.88
CAT_W	50.56
CAT_W	49.01
CAT_W	48.25
CAT_W	47.45
CAT_W	42.98
CAT_W	44.99
CAT_W	50.36
CAT_W	49.9
CAT_W	49.01
CAT_W	48.93
CAT_WC	51.12
CAT_WC	50.23
CAT_WC	49.99
CAT_WC	49.87
CAT_WC	48

CAT_WC	49.56
CAT_WC	49.01
CAT_WC	48.33
CAT_WC	47.23
CAT_WC	45.12
CAT_WC	42.03
CAT_WC	44.99
CAT_WC	50.23
CAT_WC	49.18
CAT_WC	49.3
CAT_WC	49.01
CAT_WC	48.45
CAT_M	32.09
CAT_M	33.12
CAT_M	31.99
CAT_M	33
CAT_M	31.09
CAT_M	33.45
CAT_M	29.99
CAT_M	30.87
CAT_M	31.34
CAT_M	32.98
CAT_M	33.56
CAT_M	33.17
CAT_M	32.66
CAT_M	31.23
CAT_M	37.45
CAT_M	32.29
CAT_M	33.55
CAT_MC	44.59
CAT_MC	45.4
CAT_MC	43.98
CAT_MC	44.91
CAT_MC	42.74
CAT_MC	40.71
CAT_MC	42.67
CAT_MC	45.65
CAT_MC	43.33
CAT_MC	44.15
CAT_MC	44.95
CAT_MC	43.54
CAT_MC	44.47
CAT_MC	42.31
CAT_MW	39.84
CAT_MW	41.76
CAT_MW	44.67
CAT_MW	42.41
CAT_MW	43.21
CAT_MW	44
CAT_MW	42.61

CAT_MW	43.52
CAT_MW	41.41
CAT_MW	39.38
CAT_MW	40.32
CAT_MW	40.44
CAT_MW	40.72
CAT_MW	39.42
CAT_MW	41.74
CAT_MW	40.39
CAT_MWC	39.45
CAT_MWC	41.35
CAT_MWC	44.23
CAT_MWC	41.99
CAT_MWC	42.78
CAT_MWC	43.56
CAT_MWC	42.19
CAT_MWC	43.09
CAT_MWC	41
CAT_MWC	39.19
CAT_MWC	38.99
CAT_MWC	39.92
CAT_MWC	40.04
CAT_MWC	40.32
CAT_MWC	39.03
CAT_MWC	41.33
CAT_MWC	39.99

Group	Score
LPO_U	3.19
LPO_U	2
LPO_U	3.45
LPO_U	3.99
LPO_U	2.09
LPO_U	3.1
LPO_U	3.98
LPO_U	2.45
LPO_U	4.08
LPO_U	3.2
LPO_U	3.88
LPO_U	3.01
LPO_U	2.76
LPO_U	3.19
LPO_U	3.77
LPO_U	4.01
LPO_U	4.23
LPO_C	2.34
LPO_C	3.49
LPO_C	2.94
LPO_C	3.62
LPO_C	4.07
LPO_C	3.25
LPO_C	3.58
LPO_C	2.95
LPO_C	3.51
LPO_C	4.85
LPO_C	4.64
LPO_C	2.9
LPO_W	2.07
LPO_W	3.09
LPO_W	2.6
LPO_W	3.2
LPO_W	3.6
LPO_W	2.88
LPO_W	4.36
LPO_W	3.06
LPO_W	3.21
LPO_W	3.56
LPO_W	4.29
LPO_W	4.73
LPO_W	2.97
LPO_W	3.65
LPO_W	3.38
LPO_WC	2.09
LPO_WC	3.99
LPO_WC	2.45

LPO_WC	3.45
LPO_WC	3.12
LPO_WC	2.99
LPO_WC	4.59
LPO_WC	2.06
LPO_WC	3.17
LPO_WC	2.61
LPO_WC	3.11
LPO_WC	4.29
LPO_WC	4.11
LPO_WC	2.57
LPO_WC	3.78
LPO_WC	3.33
LPO_WC	3.1
LPO_M	4
LPO_M	4.98
LPO_M	4.6
LPO_M	4.34
LPO_M	5.57
LPO_M	4.23
LPO_M	3.99
LPO_M	5.99
LPO_M	6.23
LPO_M	4.99
LPO_M	5.01
LPO_M	4.23
LPO_M	4.49
LPO_M	4.63
LPO_M	5.23
LPO_M	3.23
LPO_M	3.88
LPO_MC	4.89
LPO_MC	4.18
LPO_MC	4.56
LPO_MC	5.25
LPO_MC	4
LPO_MC	3.18
LPO_MC	4.13
LPO_MC	3.99
LPO_MC	4.73
LPO_MC	4.8
LPO_MC	4.1
LPO_MC	4.47
LPO_MC	5.15
LPO_MC	3.92
LPO_MW	3.15
LPO_MW	4.09
LPO_MW	3.96

LPO_MW	4.69
LPO_MW	4.75
LPO_MW	4.06
LPO_MW	4.43
LPO_MW	5.1
LPO_MW	3.89
LPO_MW	4.81
LPO_MW	4.74
LPO_MW	5.09
LPO_MW	3.97
LPO_MW	5.33
LPO_MW	4.08
LPO_MW	4.7
LPO_MW(	3.09
LPO_MW(	4.01
LPO_MW(	3.88
LPO_MW(	4.6
LPO_MW(	4.66
LPO_MW(	3.98
LPO_MW(	4.34
LPO_MW(	5
LPO_MW(	3.81
LPO_MW(	4.01
LPO_MW(	4.72
LPO_MW(	4.65
LPO_MW(	4.99
LPO_MW(	3.89
LPO_MW(	5.23
LPO_MW(	4
LPO_MW(	4.61

Group	Score
Gpx_U	21.58
Gpx_U	22
Gpx_U	22.56
Gpx_U	21.23
Gpx_U	21.45
Gpx_U	26.2
Gpx_U	23.56
Gpx_U	21.09
Gpx_U	23.56
Gpx_U	21.99
Gpx_U	22.59
Gpx_U	21.34
Gpx_U	22.99
Gpx_U	23
Gpx_U	24.1
Gpx_U	22.34
Gpx_U	22.1
Gpx_C	23.14
Gpx_C	25.05
Gpx_C	24.45
Gpx_C	26.04
Gpx_C	25.17
Gpx_C	21.8
Gpx_C	22.9
Gpx_C	24.59
Gpx_C	26.36
Gpx_C	25.22
Gpx_C	25.06
Gpx_C	22.67
Gpx_W	21.23
Gpx_W	22.98
Gpx_W	22.43
Gpx_W	23.89
Gpx_W	23.09
Gpx_W	20
Gpx_W	21.43
Gpx_W	23.9
Gpx_W	22.12
Gpx_W	23.15
Gpx_W	24.2
Gpx_W	21.34
Gpx_W	19.32
Gpx_W	20.45
Gpx_W	21.36
Gpx_WC	21.23
Gpx_WC	22
Gpx_WC	22.45

Gpx_WC	23
Gpx_WC	23.09
Gpx_WC	21.11
Gpx_WC	22.67
Gpx_WC	22.14
Gpx_WC	21.01
Gpx_WC	22.56
Gpx_WC	24.18
Gpx_WC	23.14
Gpx_WC	22.99
Gpx_WC	20.8
Gpx_WC	22.91
Gpx_WC	21.45
Gpx_WC	21.63
Gpx_M	15.99
Gpx_M	17.5
Gpx_M	18.3
Gpx_M	15.4
Gpx_M	13.33
Gpx_M	19.3
Gpx_M	15.2
Gpx_M	18.45
Gpx_M	18.99
Gpx_M	14.45
Gpx_M	16.23
Gpx_M	15.23
Gpx_M	18.99
Gpx_M	19.34
Gpx_M	20
Gpx_M	13.3
Gpx_M	15.99
Gpx_MC	23.53
Gpx_MC	22.96
Gpx_MC	21.92
Gpx_MC	24.01
Gpx_MC	21.35
Gpx_MC	22.98
Gpx_MC	23.54
Gpx_MC	23.54
Gpx_MC	23.54
Gpx_MC	23.54
Gpx_MC	23.54
Gpx_MC	23.54
Gpx_MC	23.54
Gpx_MC	23.54
Gpx_MC	23.54
Gpx_MC	23.54
Gpx_MC	23.54

Gpx_MW	22.9
Gpx_MW	23.46
Gpx_MW	25.28
Gpx_MW	22.9
Gpx_MW	22.99
Gpx_MW	22.43
Gpx_MW	21.42
Gpx_MW	23.46
Gpx_MW	20.86
Gpx_MW	19.73
Gpx_MW	23.45
Gpx_MW	22.9
Gpx_MW	21.51
Gpx_MW	21.41
Gpx_MW	23.87
Gpx_MW	22.9
Gpx_MW(	22.45
Gpx_MW(	23
Gpx_MW(	24.78
Gpx_MW(	22.45
Gpx_MW(	22.54
Gpx_MW(	21.99
Gpx_MW(	21
Gpx_MW(	23
Gpx_MW(	20.45
Gpx_MW(	18.96
Gpx_MW(	19.34
Gpx_MW(	22.99
Gpx_MW(	22.45
Gpx_MW(	21.09
Gpx_MW(	20.99
Gpx_MW(	23.4
Gpx_MW(	22.45

Group	Score
GSH_U	30.2
GSH_U	33.12
GSH_U	32.56
GSH_U	33.05
GSH_U	34.1
GSH_U	32
GSH_U	30.09
GSH_U	28.9
GSH_U	29.2
GSH_U	31.02
GSH_U	33.21
GSH_U	29.99
GSH_U	32.1
GSH_U	31.34
GSH_U	30.23
GSH_U	28.56
GSH_U	30.94
GSH_C	31.4
GSH_C	26.94
GSH_C	25.88
GSH_C	24.71
GSH_C	25.06
GSH_C	29.18
GSH_C	28.09
GSH_C	28.77
GSH_C	29.33
GSH_C	30
GSH_C	26.9
GSH_C	27.49
GSH_W	30.34
GSH_W	26.03
GSH_W	25
GSH_W	23.87
GSH_W	24.21
GSH_W	28.19
GSH_W	26.98
GSH_W	26.75
GSH_W	27.14
GSH_W	28.54
GSH_W	28.99
GSH_W	26
GSH_W	26.78
GSH_W	28.43
GSH_W	28.63
GSH_WC	25.23
GSH_WC	24.99
GSH_WC	26.12
GSH_WC	25.09
GSH_WC	28.34

GSH_WC	27.34
GSH_WC	26.99
GSH_WC	27.14
GSH_WC	27.8
GSH_WC	28.34
GSH_WC	28.99
GSH_WC	25.99
GSH_WC	26.56
GSH_WC	27.88
GSH_WC	28.23
GSH_WC	28.5
GSH_WC	30.34
GSH_M	17.23
GSH_M	18.34
GSH_M	19.34
GSH_M	16.45
GSH_M	17.77
GSH_M	18.91
GSH_M	17.23
GSH_M	16.22
GSH_M	19.23
GSH_M	16.23
GSH_M	18.34
GSH_M	15
GSH_M	18.23
GSH_M	18.39
GSH_M	17.45
GSH_M	18.92
GSH_M	16.34
GSH_MC	25.56
GSH_MC	24.29
GSH_MC	24.01
GSH_MC	25.68
GSH_MC	26.82
GSH_MC	22.93
GSH_MC	23.47
GSH_MC	25.13
GSH_MC	23.9
GSH_MC	22.82
GSH_MC	21.68
GSH_MC	21.44
GSH_MC	22.93
GSH_MC	23.95
GSH_MW	25.14
GSH_MW	25.74
GSH_MW	27.55
GSH_MW	26.21
GSH_MW	25.02
GSH_MW	23.78
GSH_MW	23.51

GSH_MW	25.14
GSH_MW	26.26
GSH_MW	25.64
GSH_MW	26.26
GSH_MW	27.5
GSH_MW	21.54
GSH_MW	23.74
GSH_MW	26.73
GSH_MW	25.02
GSH_MWC	22.45
GSH_MWC	22.98
GSH_MWC	24.6
GSH_MWC	23.4
GSH_MWC	22.34
GSH_MWC	21.23
GSH_MWC	20.99
GSH_MWC	22.45
GSH_MWC	23.45
GSH_MWC	22.34
GSH_MWC	22.89
GSH_MWC	23.45
GSH_MWC	24.55
GSH_MWC	19.23
GSH_MWC	21.2
GSH_MWC	23.87
GSH_MWC	22.34
